# On-oligonucleotide olefin metathesis in water

**DOI:** 10.1038/s41467-026-72995-4

**Published:** 2026-05-12

**Authors:** Chun Zhang, Christian O. Blanco, Anastasiya Khimich, Deryn E. Fogg, Andreas Brunschweiger

**Affiliations:** 1 Institute of Pharmacy and Food Chemistry, Julius-Maximilians-Universität Würzburg, Würzburg, Germany; 2 Center for Catalysis Research & Innovation, and Department of Chemistry and Biomolecular Sciences, University of Ottawa, Ottawa, ON Canada; 3 Department of Chemistry, University of Bergen, Bergen, Norway

**Keywords:** Combinatorial libraries, DNA, RNA, Synthetic chemistry methodology

## Abstract

Genetically-encoded libraries represent a breakthrough in the large-scale screening of chemical matter. Within such technologies, encoded libraries of synthetic macrocycles generated by ring-closing metathesis hold significant potential to address conventionally undruggable targets. To date, however, the mutual incompatibility of olefin metathesis catalysts and nucleic acids, exacerbated by short catalyst lifetimes in water, has limited advance. Described herein is the synthesis of nucleic acid-tagged macrocycles via ring-closing metathesis (RCM) in neat water, using an anionic, water-soluble ruthenium catalyst designed for this purpose. The ruthenium complex exhibits high compatibility with different oligonucleotide tags, including DNA and RNA oligomers and a chemically-stabilized DNA congener, furnishing the target macrocycles with useful conversions and recovery of the nucleic acid. This advance paves the way for uptake of ring-closing metathesis in water for encoded-library technologies, and for broader applications of nucleic acids, potentially including RNA-oligonucleotide drugs.

## Abstract (web summary)

Encoded libraries of synthetic macrocycles generated by ring-closing metathesis (RCM) hold significant potential to address conventionally undruggable targets, but the application of RCM to oligonucleotide-conjugated systems has been restricted. Here, authors report the synthesis of nucleic acid-tagged macrocycles via RCM in neat water, using an anionic, water-soluble ruthenium catalyst.

## Introduction

Genetically encoded library technologies, including RNA-display and DNA-encoded libraries (DEL), are accelerating the discovery of drug-like ligands with high affinity for protein targets^1–3^. Use of such platforms to synthesize and screen vast molecular collections is yielding increasing numbers of cell-permeable, target-specific small-molecule clinical candidates^1^. However, their chemical diversity is restricted by the limited range of transformations compatible with nucleic-acid tags, and with water as a reaction medium^1–3^. DEL researchers are increasingly seeking ways to access libraries of DNA-encoded macrocycles beyond those available via the established methodologies of head-to-tail lactam formation and Cu-promoted “click” chemistry (alkyne-azide cycloaddition)^4–7^. Other approaches, such as thioether formation, are slowly being adopted. In general, however, current synthetic strategies typically produce polar macrocycles that would require extensive optimization for bioavailability, a prerequisite to reach intracellular targets, for example^4^.

**Fig. 1:**
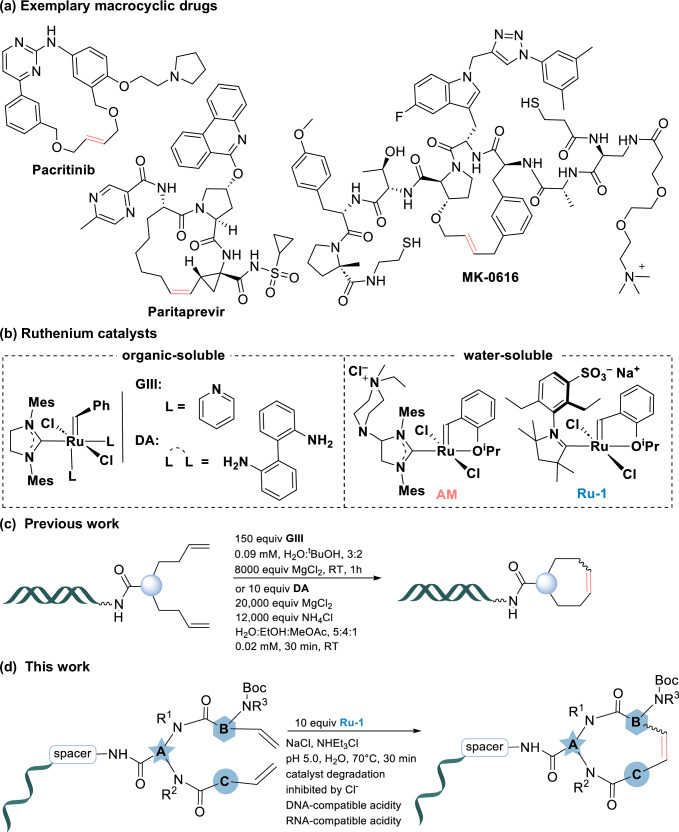
RCM tools for drug discovery. Olefinic linkages shown in red.** a** Selected macrocycles in clinical use or development accessed via RCM as a key step: pacritinib, a drug used to treat myelofibrosis, paritaprevir, a drug used to treat hepatitis C infection, MK-0616, a clinical candidate for the treatment of hypercholesterolemia^9,45–48^. **b** Metathesis catalysts discussed. **c** Prior work: current state-of-the-art conditions for on-DNA RCM. **d** This work: RCM of oligonucleotide-tagged olefins in neat water.

Ring-closing metathesis (RCM) is a leading means of introducing cyclic structural elements into linear oligoamides to furnish diverse artificial macrocycles^8^. RCM features prominently in discovery routes to clinical-stage and marketed macrocyclic drugs (Fig. 1a)^9^. Macrocycle preorganization via a hydrophobic olefin linkage can reduce the entropic costs of target binding, enhance metabolic stability, and improve membrane permeability^10^. The impact of these properties is illustrated by the recent report of sulanemadlin, the first stapled-peptide clinical candidate to reach clinical phase 1 studies, which was investigated for the treatment of acute myeloid leukemia and myelodysplastic syndrome^11^.

Despite this potential, the application of RCM to oligonucleotide-conjugates has been restricted by the chemical reactivity of nucleic acids, their poor compatibility with olefin metathesis catalysts, and the inefficiency of challenging RCM reactions in water^12^. Only two reports to date describe the metathesis of olefins appended to oligonucleotide barcodes^13,14^. Attempts at aqueous metathesis using a water-soluble catalyst were abandoned, following failed experiments^14^ with cationic AquaMet (**AM**), the leading commercial water-soluble catalyst for olefin metathesis^15^. Instead, both reports employed conventional, organic-soluble metathesis catalysts in organic-aqueous solvent mixtures. The pioneering 2017 study by Lu and co-workers at GlaxoSmithKline (GSK) described the first successful RCM for DEL applications, albeit the 150-fold excess of catalyst required (**GIII**; Fig. 1b, c), led to challenges with barcode degradation^9^. Young, Simmons, and co-workers were able to access DNA-tagged macrocycles at lower ruthenium loadings via dianiline complex **DA** (Fig. 1b), after painstaking optimization of the solvent system (Fig. 1c)^14^. However, ring-contraction reactions arising from catalyst degradation were evident by mass spectrometric analysis. In short, notwithstanding their groundbreaking nature, these reports demonstrate clear challenges of RCM methodologies with respect to barcode stability, library fidelity, and scalability^13,14^.

No further advances have been reported in the ensuing period. This absence underscores the limitations of the state of the art: in particular, the need for oligonucleotide-compatible catalysts that efficiently engage in aqueous RCM. Success in RCM of nucleic acid-tagged substrates in water would significantly expand the scope of encoded library technologies, potentially including in situ macrocyclization during DEL synthesis. Beyond production of conformationally-defined oligonucleotide conjugates, such capabilities may give access to a broader scope of structural modulations of aptamers, antisense agents, and other biologically-active oligonucleotides. Importantly, they also offer new opportunities to design structurally modulated RNA oligonucleotides via olefin linkages. Today, the chemistry toolbox for structural modulation of RNA oligomers is limited to head-to-tail cyclization via enzymatic ligation of the phosphate backbone, and Cu-promoted click chemistry^16,17^.

Here we describe a robust, oligonucleotide-compatible RCM strategy that delivers on these needs (Fig. 1d). Using a purpose-designed catalyst and building on our recent insights into the speciation of ruthenium metathesis catalysts in water^18^, we exploit high chloride concentrations and DNA-compatible acidity to inhibit catalyst degradation into metathesis-inactive ruthenium hydroxides^19^. Synthetic macrocycles tethered to DNA or RNA can then be generated with high yields in water, under conditions that preserve nucleic acid integrity. This advance paves the way to expanding the structural and functional diversity of nucleic acid-based discovery platforms.

## Results

### Catalyst design

In pursuing encoding technologies (DEL, RNA display), we employed our recently developed^20^ anionic metathesis catalyst **Ru-1** (Fig. 1b), which led to breakthrough performance in metathesis of unprotected nucleoside- and carbohydrate-tagged olefins in neat water^21^. An invaluable feature in **Ru-1** is the cyclic alkylamino carbene (CAAC) group on the metal, in place of the N-heterocyclic carbene (NHC) present in **AM** and **DA** (Fig. 1b). The CAAC ligand confers improved tolerance for water^20,21^, nucleophiles, Brønsted bases^22^, and the ethylene coproduct of RCM^23–25^. Importantly, it also ensures that the decomposed catalyst is essentially inactive in C=C migration^18,26^, an isomerization reaction prevalent for NHC catalysts^27^, which is particularly aggressive if water is present^18^. Such side-reactions represent a major hazard for macrocyclization, because isomerization prior to metathesis results in ring-contraction, as reported in early RCM efforts from pharma^27,28^, and evident from mass spectroscopic analysis in the prior DEL work^14^. That is, isomerization undermines the controlled specification of ring sizes essential for library fidelity in DEL applications. We deemed the capacity to preserve size-selectivity sufficiently valuable to offset the slower turnovers associated with the CAAC ligand, which necessitate the use of elevated temperatures to achieve acceptable rates of metathesis^20^ (see below).

A second key feature, unique to **Ru-1** among the hundreds of olefin metathesis catalysts reported to date, is the incorporation of an anionic sulfonate tag to confer solubility in water. A cationic ammonium group is routinely employed in water-soluble metathesis catalysts, including **AM**^29^. The sulfonate group improves the water-solubility of the catalyst^20^: it also remains anionic over a wide pH range, and exhibits minimal tendency to bind to ruthenium. As a potentially more fundamental asset in the present context, we anticipated that the negative charge on **Ru-1** would confer compatibility with oligonucleotides, via electrostatic repulsion with the polyanionic phosphate backbone (as compared with the electrostatic attraction posited^14^ for **AM**). No such electrostatic protection is present in prior ruthenium catalysts, all of which—even those which commence as neutral complexes—form cationic complexes in water upon aquation of the Ru–Cl bonds, and may thus compromise barcode integrity via coordinative degradation of oligonucleotides. Of note, the GSK study utilizing **GIII** (Fig. 1b, c) explicitly noted DNA decomposition^9^. In short, mutual tolerance between the barcode and the metal complex is critical to protect the structural integrity essential for barcode legibility and RCM activity, respectively. Promising from this perspective are precedents in which Pd-sulfonate catalysts were employed for Suzuki-Miyaura coupling of DNA-appended groups in water^30,31^.

### Assessing precatalyst stability to DNA and RNA oligonucleotides

**Fig. 2:**
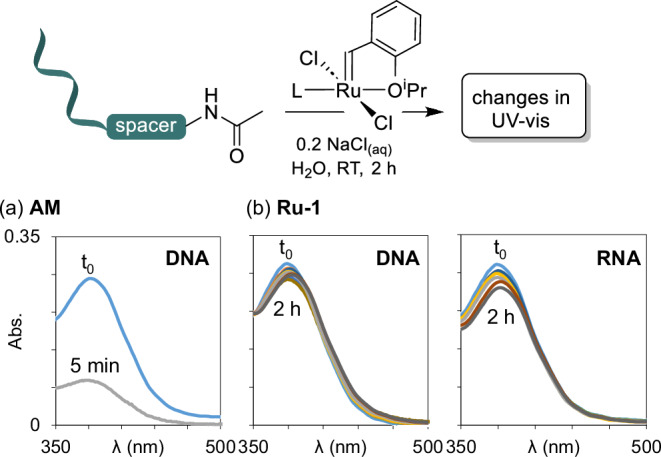
Probing precatalyst stability to oligonucleotides. **a** Fast degradation of **AM** after 5 min. **b** Relative stability of **Ru-1** incubated with ss-DNA or RNA after 2 h.

To test the hypothesis that the anionic charge on **Ru-1** protects it from reaction with oligonucleotides, whereas cationic **AM** is degraded, we incubated the precatalysts with single-stranded oligonucleotide sequences (ss-DNA, RNA) in 0.2 M NaCl_(aq)_, and monitored the ensuing changes in the UV–vis spectra. The results for both **AM** and **Ru-1** are presented in Fig. 2. The metal-to-ligand charge transfer band for **AM** exhibited a 75% drop in intensity within 5 min exposure to DNA (Fig. 2a), consistent with degradation of the precatalyst. In contrast, minor decreases in band intensity were observed over 2 h on incubating **Ru-1** with DNA or RNA (Fig. 2b).

### Stability of active catalysts to oligonucleotides

**Fig. 3:**
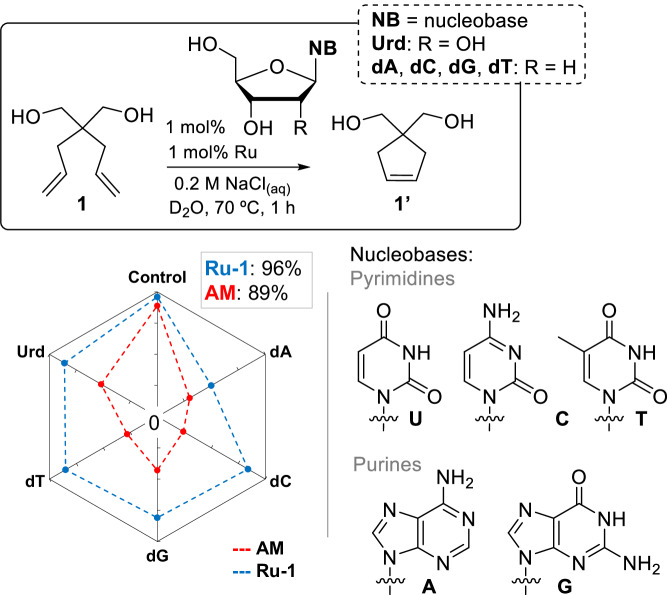
Impact of nucleosides on RCM productivity. Outer black line on radar plot = 100% RCM. For numerical values, see Supplementary Table S1.

The ruthenium species formed in the active cycle for olefin metathesis are significantly more susceptible to decomposition than the precatalysts. We previously established the susceptibility of Ru-NHC catalysts to decomposition by amine nucleophiles and Brønsted bases during catalysis^22,32^, and the superior robustness of Ru-CAAC complexes^22^. To assess the tolerance of the active catalysts for the nucleosides present in RNA and DNA, and to identify which nucleosides are of greatest concern, we examined the impact on RCM yields of added uridine (**Urd**), and the 2′-deoxy-nucleosides adenosine, cytidine, guanosine, and thymidine (**dA,** **dC,** **dG,** and **dT**, respectively; Fig. 3 and Supplementary Table S1). Each nucleoside was added in amounts equimolar with Ru, a proportion that was previously published for RCM on the oligonucleotide barcodes in DEL synthesis^13^. The water-soluble diene **1** was chosen as the RCM substrate for its ease of cyclization, which translates into a robust probe for problems.

The radar plot of Fig. 3 highlights the impact of individual nucleoside functionalities on catalyst activity. RCM was near-quantitative for both **AM** and **Ru-1** in the control experiment (vertical line), in which no nucleoside is present. For **Ru-1**, a minor decline in RCM yield (10–15%) was seen for all nucleosides, with the exception of **dA**, for which a 46% decrease was evident. In comparison, yields for **AM** dropped by >60% in the presence of **dA,** **dG** (Supplementary Table S1), or **dT**, and by 37–46% in the presence of **Urd** or **dC**. Clearly, **Ru-1** exhibits much superior compatibility with the constituents of a genetic barcode, although the purine nucleoside **dA** remains a hazard.

### Stability of nucleosides to active ruthenium catalysts

**Fig. 4:**
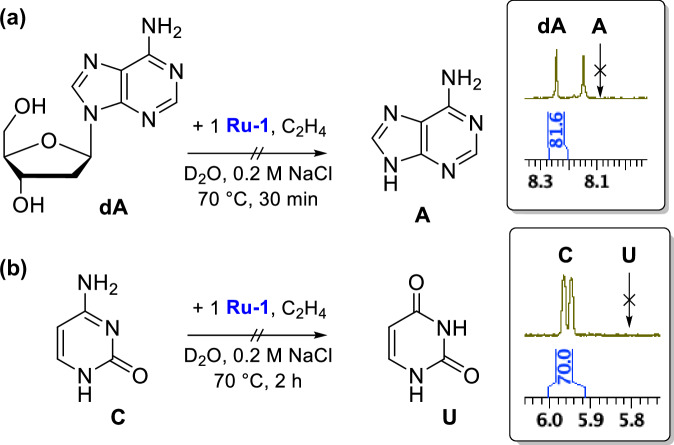
NMR assays of degradation by Ru-1 under RCM conditions. **a** Nucleoside stability to depurination (Supplementary Table S3). **b** Nucleobase stability to deamination (Supplementary Table S2).

We next examined the reciprocal issue: the impact of the ruthenium complexes on the genetic barcode. Barcode damage commonly results from depurination (for **A,** **G**) or hydrolytic deamination of the nucleobase (for **A,** **G,** **C**). To assess the tendency of **Ru-1** to induce these well-established degradation pathways, we undertook NMR experiments with deoxyadenosine **dA**, the most aggressive of the nucleosides examined above, and cytosine **C**, the nucleobase most susceptible to deamination^33,34^. As an aggressive test of stability (Fig. 2b), we conducted these experiments at 70 °C under ethylene. These conditions are chosen to maximize formation of the most accessible, most reactive ruthenium species generated during metathesis: that is, the [Ru]=CH_2_ intermediate and the unsubstituted metallacyclobutane. A much lower proportion of these species will be present in the on-DNA/on-RNA metathesis experiments below, in which the 10:1 stoichiometry of **Ru-1** vs diene limits the proportion of ethylene to <10% relative to Ru. Nevertheless, even under an atmosphere of ethylene, we observed neither cleavage of the riboside C–N bond in **dA** (Fig. 4a, Supplementary Figs. S5, S6, and Table S2), nor hydrolysis of the exocyclic amine in **C** (Fig. 4b, Supplementary Figs. S7, S8, and Table S3). The absence of deamination or depurination under forcing conditions implies that these side-reactions are irrelevant in the DEL experiments, in which ethylene concentrations are limited by both stoichiometry and the low solubility of ethylene in aqueous solvents at elevated temperatures^35^.

### Stability of RNA/DNA to ruthenium precatalysts

**Fig. 5:**
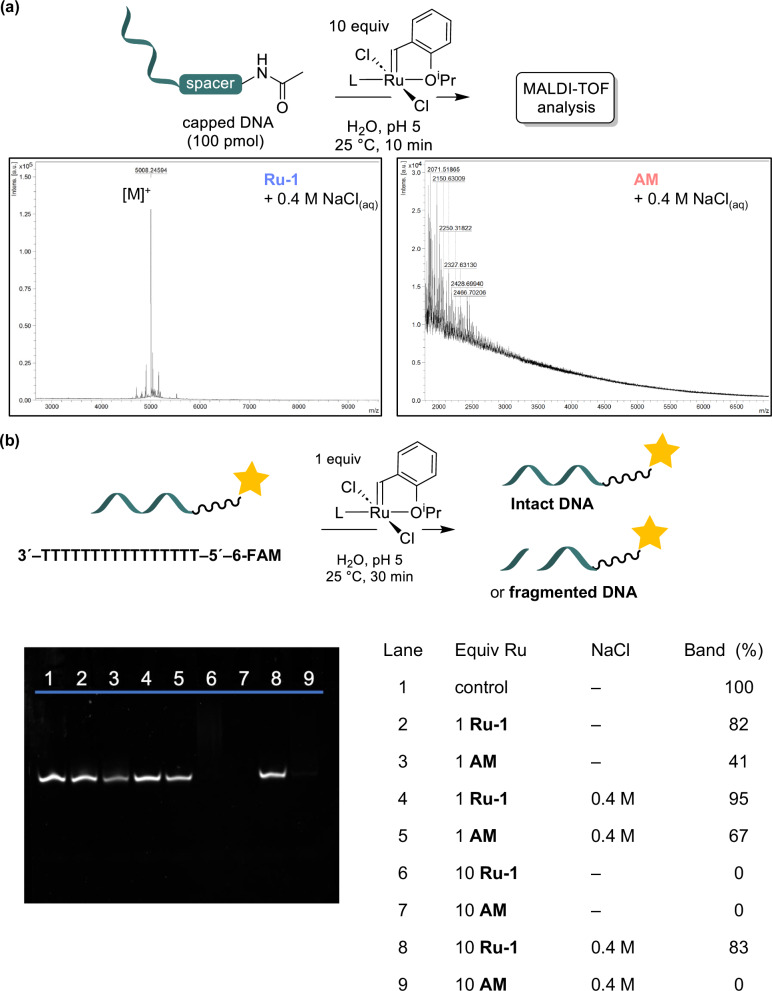
Oligonucleotide compatibility with Ru-1 and AM. **a** MALDI-TOF mass spectra. **b** Gel electrophoresis assay for backbone degradation of fluorescein (FAM)-labeled oligothymidine 16mer, following incubation of the oligothymidine with 1 or 10 equivalents of **Ru-1** or **AM**.

Degradation of the oligonucleotides during RCM is presumed to commence with binding to ruthenium. To probe for formation of Ru-oligonucleotide adducts, we incubated a 16mer single-stranded DNA with a tenfold excess of **Ru-1** or **AM** for 10 min at ambient temperature. MALDI-TOF mass spectra revealed little adduct formation for **Ru-1**, and binding could be suppressed completely by added NaCl (Fig. 5). In contrast, **AM** induced extensive degradation within 10 min even under these mild conditions, and the mass spectra were dominated by DNA fragments (Supplementary Fig. S9). The behavior of **AM** is consistent with the susceptibility of Ru-NHC catalysts to loss of the hydrocarbon ligands by nucleophiles and water^18,36^, and ensuing attack of low-coordinate ruthenium species on the oligonucleotide chains. The protective effect of NaCl (notwithstanding its limitations in the case of **AM**) has ample precedents from literature reports documenting the stabilizing effect of chloride salts on the ruthenium catalysts: specifically, inhibition of aquation by water, the first step in the decomposition cascade^18,37,38^. The large excess of NaCl may also shield DNA donor sites from binding by low-coordinate ruthenium degradation products, with negative impacts on oligonucleotide replication efficiency^39,40^.

### Localizing nucleic acid damage

To confirm cleavage of the oligonucleotide backbone, we carried out additional experiments with a fluorescein-labeled thymidine oligomer. Thymidine was chosen for its high chemical stability; as it cannot undergo depurination or deamination, and any damage provides strong evidence for backbone hydrolysis. A 16mer (16-T) was chosen to mimic the barcode DNA in length, and to permit analysis by gel electrophoresis. This experiment sets a key go/no go condition. That is, if the ruthenium complex degrades the chemically relatively stable 16-T oligonucleotide, conventional DNA (barcode) oligomers would stand no chance of survival.

Accordingly, the 16-T oligomer chain was incubated with the ruthenium complexes for 30 min, then analyzed by gel electrophoresis. Lane 1 in Fig. 5b shows the control experiment, with no ruthenium present. Lanes 2 and 3 show the impact of 1 equiv of **Ru-1** or **AM**, respectively, in the absence of added chloride. The intensity of the DNA band drops by <20% for **Ru-1**, vs. nearly 60% for **AM**. High concentrations of NaCl (0.4 M) enabled near-quantitative DNA recovery with **Ru-1**, as compared to <70% with **AM** (lanes 4 vs. 5). At a tenfold higher concentration of either Ru complex, no DNA was recovered in the absence of NaCl (lanes 6, 7). High-salt conditions, however, rescued the DNA for **Ru-1**, but not **AM** (Lanes 8, 9, 83% vs. 0%). HPLC analysis confirmed the gel electrophoresis results (Supplementary Table S4 and Fig. S11).

We conclude that even the chemically relatively robust oligothymidine is readily degraded by **AM**, which induces backbone hydrolysis at very low proportions of ruthenium under mild conditions (1 equiv **AM**, 0.4 M NaCl, ambient temperature; Supplementary Fig. S10). In contrast, **Ru-1** caused much less DNA damage, and high concentrations of NaCl successfully inhibited degradation by even a tenfold excess of **Ru-1** versus DNA. The protective role of NaCl is thus effective for **Ru-1**, but inadequate to mitigate DNA damage by **AM** (Supplementary Fig. S11). Similar protective effects of NaCl were observed for DNA and RNA sequences identical to those used in the RCM reaction described below. Incubation of these 16mers with 10 equivalents of **Ru-1** showed minimal loss of integrity, whether at room temperature or at 70 °C. In contrast, **AM** caused very extensive nucleic acid degradation under the same conditions (Supplementary Tables S5–S9 and Figs. S12–S21).

### RCM of RNA- and DNA-tagged olefins in water

**Fig. 6:**
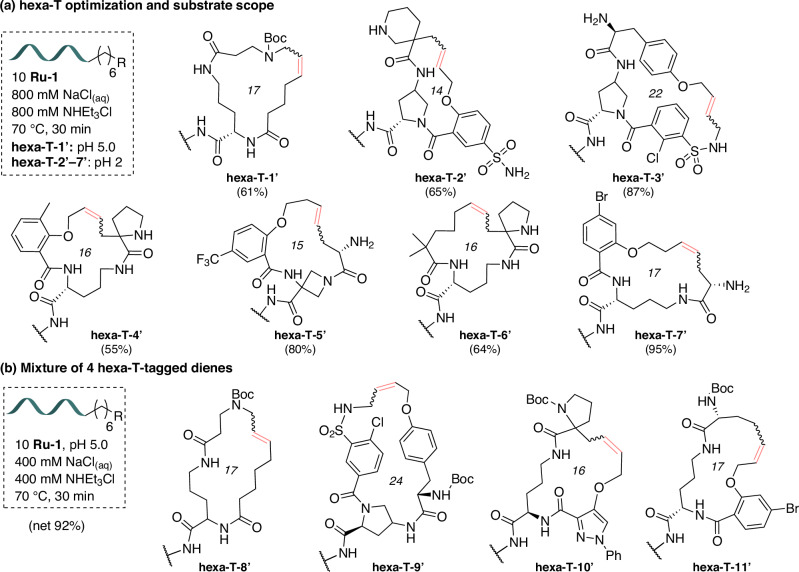
RCM in situ yields (in brackets) for macrocyclization of hexa-T-tagged substrates in water. General reaction scheme shown in Fig. 1d. **a** Optimization (for **hexa-T-1**) and substrate scope. **b** Mixture of 4 hexa-T dienes.

Having established conditions that maximize the mutual tolerance of **Ru-1** and the oligonucleotides, we turned to on-RNA and on-DNA metathesis (Fig. 6), the key cyclization step in encoded library synthesis. Four essential goals were established: (1) high in situ RCM yields; (2) ring-size selectivity^20^; (3) conservation of the oligonucleotide functionalities (e.g., nucleobase amines, ribosidic bonds) during metathesis, to preserve the encoded information derived by sequencing, and (4) isolation of the DNA-macrocycle conjugate from the reaction medium in high yields.

RCM macrocyclization presents a considerably greater challenge than RCM of the diallylamine **1**, as the lower probability of chain-end encounter (among other factors) retards cyclization^41^. Additional measures are therefore required for metathesis to outpace catalyst decomposition. One major risk factor for metathesis in water, even at neutral pH, is hydroxide ion, which triggers rapid degradation of the ruthenium catalysts^18,19^. To block replacement of the chloride ligands by hydroxide, we employed mild, DNA-compatible acidity (pH 5), in addition to high chloride concentrations. Likewise, critical is the choice of buffer. We drew on the Young-Simmons discovery that ammonium chloride salts provide a dual buffer/chloride source that helps conserve metathesis productivity (see below), whereas conventional DEL buffers drive catalyst degradation^14^. We confirmed the latter finding in experiments with phosphate, HEPES, and MES, all of which reduced in situ macrocycle yields to below 10% (Supplementary Table S12).

Reaction development was initiated with the chemically robust hexathymidine (**hexa-T**) oligomer, which permits initial optimization and exploration of scope without the complications of barcode degradation^42^. The requisite terminal olefins were tethered via a diaminocarboxylic acid to form **hexa-T-1** (Fig. 6 and Supplementary Table S12). We commenced RCM by translating the established conditions for metathesis of nucleoside-tagged olefins^20^ to the DNA-tagged olefins. Accordingly, **hexa-T-1** was reacted with excess **Ru-1** (5–10×) for 30–120 min at 70 °C in 0.4 M NaCl. Although in situ yields of **hexa-T-1′** were near-quantitative, isolated DNA yield was only 33%, suggesting that the high salt concentrations optimal for metathesis of more conventional organic substrates in water^18,37,38^ caused the oligonucleotide to precipitate. Use of organic ammonium salts as a buffer and an additional source of chloride ion, as noted above, improved total DNA recovery (that is, as a mixture of acyclic and cyclized constructs but again led to consistently lower in situ yields (Supplementary Table S12, Fig. S25; see Supplementary Tables S13–S15 and Figs. S26–S28 for isolated yields of the DNA-macrocycle conjugate, and total oligonucleotide recovery). Compromise conditions, involving use of 0.8 M NaCl with 0.8 M NEt_3_•HCl as a mild source of HCl, yielded acceptable in situ yield with excellent total DNA recovery (61% and 87%, respectively) within a reaction time of 30 min. This mixture was employed in the optimized reactions of Fig. 6b. The ammonium buffer results in a pH of 5, an acidity compatible with native DNA, which is susceptible to depurination at lower pH. (Of note, experiments conducted at pH 2 with **hexa-T-1** showed rapid RCM, but recovery of even this relatively robust oligonucleotide was limited by acid-induced degradation: Supplementary Table S12). Importantly, MALDI-MS analysis of the crude reactions revealed no ring-contracted side-products (Supplementary Fig. S25).

In a preliminary survey of substrate scope, we targeted a set of six chemically diverse, non-peptidic DNA-tagged artificial macrocycles^43^, including aromatic and heterocyclic scaffolds, a Boc-protected amine for library expansion, and ring sizes of 14–24 members. The targeted ring architectures were successfully generated on the **hexa-T**-oligonucleotide (Fig. 6a), with in situ yields of 55–95% (Supplementary Fig. S29). To simulate the complex substrate mixtures characteristic of DEL synthesis scenarios, we cyclized a mixture of hexa-T-tagged dienes under the same reaction conditions (Fig. 6b). All four underwent RCM at acceptable rates, with excellent net in situ yield (92%) and full selectivity, confirming compatibility with library development. In each case, the desired macrocycle product is the sole species evident within the detection limits, with no evidence for ring contraction, dimerization, or higher-order structures or complex mixtures of products and side-products (Supplementary Fig. S30). These results meet the go/no go condition for encoded library technologies.

**Fig. 7:**
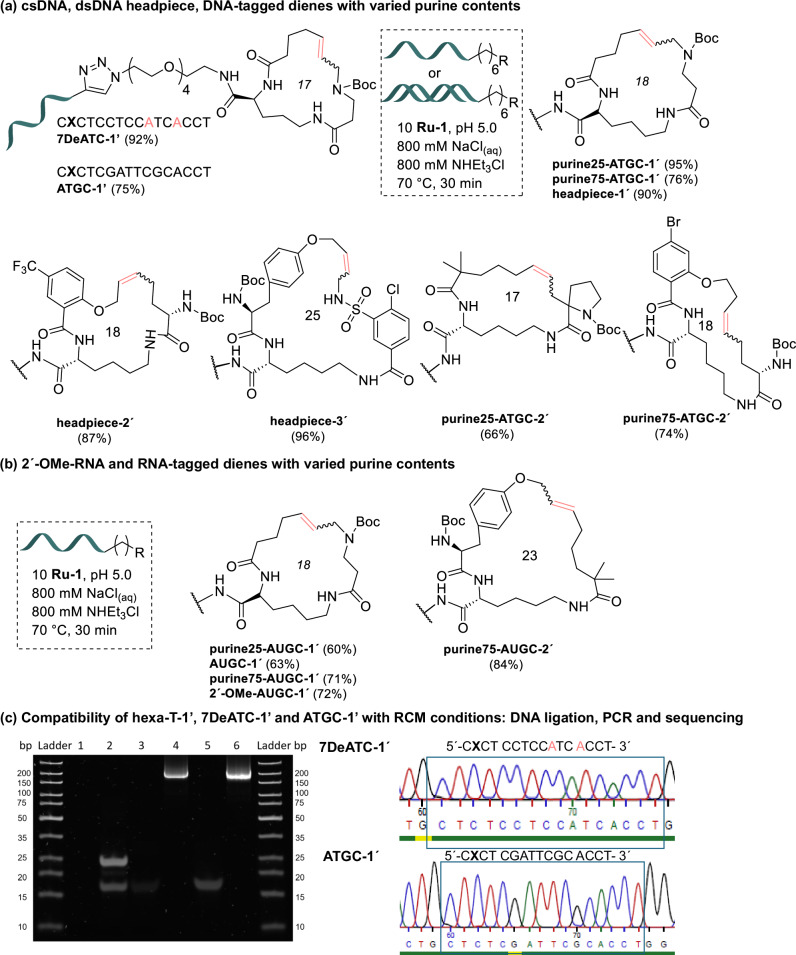
Macrocyclization of DNA/RNA-tagged substrates in water, showing in situ yields (in brackets). For the general reaction scheme, see Fig. 1d. **a** RCM on chemically stabilized DNA (csDNA), dsDNA headpiece and DNA-tagged dienes with varied purine content and scope. **b** RCM on 2′-OMe-RNA-tagged and RNA-tagged dienes with varied purine content. **c** Gel electrophoresis showing ligation of DNA oligomers **hexa-T-1′**, **7DeATC-1′**, and **ATGC-1′**. Lane 1: **hexa-T-1′** before ligation (no band due to its short hexathymidine sequence). Lane 2: **hexa-T-1′** after ligation; 22 base pairs (bp). Lane 3: 16-nucleotide (nt) **7DeATC-1′** before ligation. Lane 4: 17-bp **7DeATC-1′** ligation product. Lane 5: 16-nt **ATGC-1′** before ligation. Lane 6: 178-bp **ATGC-1′** ligation product. Sanger sequencing of barcodes.

Building on the successful optimization for hexa-T DNA, we transferred these reaction conditions to barcode mimics (Fig. 7a). Two oligomers were selected: our chemically stabilized, single-stranded 16mer barcode **7DeATC-DNA**, in which the vulnerable purine bases are replaced by 7-deazaadenine^44^, and a native single-stranded 16mer, **ATGC-DNA**. In optimizing further, we sought to balance in situ yields of the desired macrocycles with DNA integrity and recovery from the reaction solution (Supplementary Tables S16 and S17). Reaction at pH 5 and 70 °C for 30 min afforded the target macrocycles **7DeATC-1′** and **ATGC-1′** in ca. 60% in situ yields, with 80% total recovery of DNA-tagged diene and macrocycle product after HPLC (Fig. 7a). Extending the reaction time to 60 min significantly decreased total DNA recovery, almost certainly due to DNA damage, as indicated by early-eluting molecules on the HPLC column (Supplementary Figs. S31 and S32). Attempts to use milder reaction temperatures failed, as in our original report^20^. No **ATGC-1′** macrocycle was detected following reactions at 25–60 °C (Supplementary Table S17), reflecting faster decomposition than metathesis. Use of common alternative buffers (phosphate, HEPES, and MES) likewise proved unsatisfactory, with <10% in situ yields of **ATGC-1′** even under our optimized conditions (Supplementary Table S17). Similar behavior was noted for **DA**, consistent with attack of these basic buffers on the ruthenium complexes^14^.

To further probe the DNA-compatibility of our RCM protocol, we examined DNA oligomers containing different purine contents (**purine25-ATGC,** **purine75-ATGC**), as well as a dsDNA (**headpiece-DNA**), in high-throughput 96-well plate format. RCM in the presence of 0.8 M NaCl and NEt_3_·HCl proceeded with in situ yields of 66–96%. Isolated yields of the DNA-macrocycle product ranged from 42–82% (Fig. 7a), and mass analysis again revealed neither depurination or ring-contracted side products (Supplementary Fig. S34), providing further evidence for the utility of this methodology in encoded library technologies.

In a further, ambitious experiment, we explored the use of RCM to modify RNA oligomers, an area with vast application potential in which no reports have yet appeared. We began with the chemically more stable 2′-methyl-RNA as an element of diversity widely employed in RNA drug design, with experiment on the 2′-O-methyl-RNA substrate **2′-OMe AUGC-1**. Macrocyclization was achieved with an in situ yield of 72%, and the RNA-macrocycle conjugate was isolated in 35% yield (Fig. 7b and Supplementary Fig. S33). To gain insight into the generality of the reaction with respect to RNA sequence composition, we also examined RNA oligomers with different proportions of purines and pyrimidines. A 16-mer RNA with balanced, approximately equimolar nucleobase content (**AUGC**) was used as a baseline, for comparison, with purine-poor and purine-rich RNA sequences (**purine25-AUGC** and **purine75-AUGC**). The RNA oligomers with higher purine content (that is, **AUGC** and **purine75-AUGC**) gave the desired macrocycles with good in situ yields (63–84%) and 43–58% isolated yield (Fig. 7b), with no detectable depurination (Supplementary Fig. S34). Somewhat unexpectedly, isolated yields were lower for **purine25-AUGC-1′** (30%; Fig. 7b and Supplementary Fig. S33), perhaps because the purine-rich RNAs can be precipitated more easily from the reaction. These results are important in indicating a greater degree of tolerance of this RCM chemistry for diverse RNA sequences.

Finally, we tested the compatibility of the newly-developed RCM protocols for the remaining essential steps in DEL library synthesis and screening: that is, post-RCM library buildup via coupling of further barcodes (enzymatic ligation of DNA barcode oligomers); amplification of the barcodes by DNA polymerase, and sequencing of the amplicons to confirm the integrity of the nucleobase sequence. As shown in Fig. 7, both the **hexa-T-1′** barcode mimic and the true barcodes **7DeATC-1′** and **ATGC-1′** were successfully ligated to further DNA sequences (Supplementary Table S18 and Fig. S35), as confirmed by gel electrophoresis of the products versus appropriate controls. The **7DeATC-1′** and **ATGC-1′** barcodes sequenced correctly (Fig. 7c), confirming that the genetic information is unaffected by metathesis. Collectively, these results provide crucial evidence for the mutual compatibility of appropriately designed metathesis and DEL technologies.

The catalyst design, reaction conditions, and DNA-recovery protocols above meet the stringent requirements for incorporating RCM into nucleic acid-based library technologies. To assess this advance relative to the prior state of the art, we carried out a side-by-side comparison with the methods of Fig. 1c, again using dienes appended to **hexa-T**, native DNA sequence **ATGC-1**, chemically-stabilized DNA sequence **7DeATC-1**, and RNA sequences **AUGC-1** and **2′-OMe AUGC-1**. In the GSK protocol employing **GIII**, the 3:2 H_2_O:tBuOH solution became biphasic after adding MgCl_2_, and the HPLC chromatograms showed considerable decomposition following attempted RCM, with neither starting materials nor products. In the **DA** protocol, the **hexa-T-1′** macrocyclic product was obtained in 32% isolated yield, but **ATGC-1′** and **7DeATC-1′** were isolated in only ca. 10% yield. Negligible oligonucleotide recovery was evident for the RNA experiments, for which the cyclized products could be observed by MALDI-MS, but not by HPLC.

The foregoing demonstrates that the metathesis catalyst **Ru-1**, in which a sulfonate functionality confers water-solubility and improved compatibility with nucleic acids, enables the synthesis of oligonucleotide-tagged macrocycles in neat water. Tags employed include RNA, single-stranded DNA and double-stranded DNA. These advances provide an enabling tool for the expansion of encoded library technologies. They also open the door to broader applications of nucleic acids, potentially including novel DNA- and RNA-oligonucleotide drugs.

## Methods

Additional raw data, methods, procedures, oligonucleotide sequences, as well as characterization of compounds, are described in the Supplementary Information.

### General procedures for the synthesis of DNA and RNA conjugates on solid phase

#### Cu-catalyzed azide-alkyne cycloaddition

The CPG-bound oligonucleotide-alkyne conjugate (350 nmol, 1 equiv) was washed with 300 μL H_2_O. The aqueous catalyst mixture (composed of 21 μL H_2_O, 1.75 μL THPTA (100 mM, 0.500 equiv), 26.25 μL sodium ascorbate (100 mM, 7.5 equiv), and 0.875 μL CuSO_4_·5H_2_O (100 mM, 0.25 equiv); 50 μL total volume) was shaken at RT for 10 min. The aqueous catalyst mixture was added to the CPG beads along with t-Boc-N-amido-PEG4-azide (1 mg in 75 μL DMSO, 2800 nmol, 8 equiv), phosphate buffer (Dulbecco´s Phosphate Buffered saline, 30 μL, 10×) and H_2_O (145 μL). The reaction mixture was shaken at 37 °C for 1 h on an Eppendorf Thermocycler. The CPG-bound conjugate was then passed through a filter column and washed with 200 μL of 0.1 M EDTA(aq), water, DMF, MeOH, MeCN and CH_2_Cl_2_ (3× each) and dried under vacuum. The procedure was repeated once only, to maximize conversion.

#### Boc deprotection

The Boc group was cleaved from the PEG linker by shaking the CPG-coupled oligonucleotide in 10% v/v trifluoroacetic acid in CH_2_Cl_2_ (200 μL) at RT for 10 min and repeating 3×.

#### Fmoc deprotection

The Fmoc group was cleaved from CPG-coupled oligonucleotides by shaking the beads in 20% v/v piperidine in DMF (200 μL) at RT for 5 min and repeating 3×.

#### DMT (MMT)-deprotection

The DMT/MMT-protecting group of CPG-coupled DNA was cleaved by suspending the beads in 3% v/v trichloroacetic acid in CH_2_Cl_2_ (200 μL) at RT for 1 min. Development of an orange color in the solution indicated successful removal of the protecting group. Deprotection was repeated until the solution remained colorless. CPG-bound deprotected DNA was washed (3× each) with 200 μL of 1% NEt3 in MeCN, DMF, MeOH, MeCN and CH_2_Cl_2_ and then dried in vacuo.

With RNA: as above, using 3% v/v dichloroacetic acid in CH_2_Cl_2_.

#### Amide coupling reaction

CPG-bound oligonucleotides, carboxylic acids and HATU were dried in vacuo for 15 min. Stock solutions of all reactants in dry DMF were prepared fresh before the reaction was started. To the solution of the carboxylic acid (100 equiv) in 160 μL dry DMF were added HATU (100 equiv) dissolved in 40 μL dry DMF, and DIPEA (250 equiv). The mixture was shaken at 37 °C for 10 min and added to the CPG-bound oligonucleotides, and further shaken at 37 °C for 1 h. Then, the CPG-bound oligonucleotides were filtered over a filter column, washed with each 3 × 200 μL of DMF, MeOH, ACN and CH_2_Cl_2_ and dried under vacuum. The amide coupling was repeated on the CPG-bound oligonucleotides twice to maximize conversion.

#### Microcleavage for assessing conversion

A minimum amount of CPG beads was picked up with a plastic pipette tip and transferred to 50 μL of AMA solution (aqueous ammonia (30%)/aqueous methylamine (40%), 1:1, vol/vol). The suspension was shaken at 65 °C for 10 min, the mixture was dried in a SpeedVac, and the oligonucleotides were dissolved in 50 μL Millipore H_2_O for analysis by MALDI-MS and analytical RP-HPLC. In case of incomplete reactions (<90%), the reaction on the CPG-bound oligonucleotides was repeated.

#### Capping after amide coupling reactions

Unreacted amines were capped with capping solution (1:1 mixture of THF/methylimidazole, 9:1 v/v, and THF/pyridine/acetic acid anhydride 8:1:1 v/v). The CPG was treated with 200 μL of the capping solution (3×; 30 s each). The capped CPG-bound oligonucleotide was washed with 200 μL of DMF, MeOH, MeCN and CH_2_Cl_2_ (3 × each) and dried in vacuo.

### RCM of oligonucleotide-olefin conjugates in H_2_O

RCM reactions were carried out with 500 pmol of DNA or RNA oligomers. Aqueous solutions at pH 2.0 and pH 5.0 were prepared by acidifying Millipore H_2_O with 1 M HCl. pH 5.0 phosphate buffer (75 mM sodium phosphate dibasic and 25 mM sodium phosphate monobasic) was prepared by acidifying the commercial stock solution with 1 M HCl, pH 5.0 HEPES buffer (100 mM) was prepared by diluting 1 M stock solution and by acidifying with 1 M HCl, pH 5.0 MES buffer (100 mM) was prepared by dissolving 639.75 mg in 30 mL Millipore H_2_O, followed by basifying by 1 M NaOH. Stock solutions of 4 M NaCl, 4 M NHEt_3_Cl, and 0.6 mg/mL **Ru-1** were prepared in pH 2.0 and pH 5.0 H_2_O, respectively. Prior to RCM reactions, all stock solutions were filtered via sterile Millex™-GV Filter Unit (pore size 0.22 μm, 33 mm diameter, PVDF membrane). All RCM reactions were performed in a total volume of 50 μL in 1.5 mL Eppendorf tubes. To the tubes were added 5 μL oligonucleotide, 5 μL 4 M NaCl and 5 μL 4 M NHEt_3_Cl solutions (40,000 equiv NaCl and NHEt_3_Cl), 5 μL **Ru-1** solution, following which pH 2.0 or pH 5.0 H_2_O was added to reach a final volume of 50 μL. The solution was flushed with argon, sealed with Parafilm and shaken on an Eppendorf thermocycler at the temperature and time specified (typically 70 °C, 30 min). The reactions were quenched by adding SnatchCat (1,4-bis(3-isocyanopropyl) piperazine; 10 equiv vs. Ru) and shaking at 37 °C for 30 min. The reactions were analyzed by MALDI-MS and analytical RP-HPLC. Samples were desalted prior to MALDI-MS analysis using ZipTip pipette tips according to the manufacturer's protocol.

### DNA ligation and Sanger sequencing

#### 5′-phosphorylation of DNA

For 5′-phosphorylation of DNA in a total reaction volume of 20 μL, 10 units of T4 polynucleotide kinase (T4 PNK, Thermo Fisher), 1x PNK Buffer A (50 mM Tris-HCl, 10 mM MgCl_2_, 5 mM DTT, 0.1 mM spermidine, pH = 7.6 at 25 °C, Thermo Fisher) and 1 mM ATP (Thermo Fisher) were used. Reaction mixtures were incubated at 37 °C for 20 min, then heat-inactivated at 75 °C for 15 min and slowly cooled to 4 °C.

#### Ligation of DNA

Prior to enzymatic ligation of DNA, the oligonucleotides were annealed by incubation at 65 °C for 10 min, then slowly cooled down to 4 °C. For ligation (40 μL scale), 100 pmol of each oligonucleotide, 600 units of T4 DNA Ligase (T4 DNA ligase, New England Biolabs), and 1x T4 DNA Ligase Buffer (50 mM Tris-HCl, 10 mM MgCl_2_, 10 mM DTT, 1 mM ATP, pH = 7.5 at 25 °C, New England Biolabs) were mixed. Ligation reactions were performed at 25 °C for 16 h, then stopped by heat inactivation at 75 °C for 15 min and cooled down to 4 °C.

#### Analysis of DNA ligation

DNA ligation reactions were analyzed by gel electrophoresis (12% polyacryamide). Electrophoresis was carried out in TBE buffer (89 mM tris/borate, 2 mM EDTA, pH 8.3) at 120 V constant voltage for 45 min. The DNA ligation products were stained using SYBR™ Gold (Thermo Fisher) with GeneRuler Ultra Low Range DNA Ladder (Thermo Fisher) as a reference. Gel imaging was performed using the Bio-Rad GelDoc Go Gel imaging system.

#### Purification of DNA ligation product by E-Gel™ Power Snap Electrophoresis System

The ligation products of native and csDNA were purified by 2% E-Gel™ SizeSelect™ II Agarose Gels (Thermo Fisher) using E-Gel™ Power Snap Electrophoresis System (Thermo Fisher). The purification was performed in accordance with the manufacturer’s protocol.

#### Sanger sequencing

Sanger sequencing of purified ligation products was performed by Eurofins Genomics. The sample preparations were carried out according to the TubeSeq Supreme protocol.

##### Representative RCM reaction with diene 1

A stock of substrate solution was prepared by dissolving diene **1** (32 mg, 0.10 mmol), NaCl (48 mg, 0.82 mmol, 820 equiv), and dimethyl sulfone, Me_2_SO_2_ (ca. 5 mg, internal standard) in 4 mL D_2_O; final concentration 100 mM **1**. A 500 μL aliquot was reserved for NMR analysis to establish the initial ratio of **1**: Me_2_SO_2_. For each reaction, 500 μL of diene **1** stock solution and 30 μL of the additive (7.5 mM stock solution of nucleosides **dA,** **dG,** **dC,** **dT**, or **Urd**) were added; one reaction was run without an additive (control). To the stirred solutions at 70 ± 1 °C (glovebox, degassed oil bath) was added 1.0 mol% **Ru-1** (85 μL of a stock solution of 2.0 mg **Ru-1** in 1.00 mL D_2_O). After 2 h, the solutions were quenched with KTp in THF (10 mg/mL; 10 equiv vs starting Ru) and analyzed (NMR).

##### Stability of Ru-1 in the presence of native DNA

To a screw-capped quartz cuvette with a septum seal was added H_2_O (1.5 mL, pH = 5 prior to catalyst addition) and solid NaCl (95 mg, 1.62 mmol, 40,000 equiv). The cuvette was sealed, wrapped with Parafilm and removed from the glovebox to the spectrometer to measure the blank read. An aliquot of a stock solution of **Ru-1** in water (14 μL, 41 nmol, and 2.0 mg/mL) was injected via gas-tight syringe (puncture promptly covered with Parafilm) to give a final Ru concentration of 27.6 μM. The first UV–vis spectrum was taken 5 min after preparing the catalyst stock solution. An aliquot of a stock solution of the amide-capped native DNA **Nat-0** (80 μL, 0.51 nmol/μL, 1 equiv) was injected. Spectra were recorded periodically up to 2 h. UV–vis spectra showing the stability of **Ru-1** to various oligonucleotides appear in Fig. 4 in the main text.

##### Evaluating deoxyadenosine depurination by Ru catalyst

Deoxyadenosine **dA** (2 mg, 0.008 mmol, 1 equiv), NaCl (11 mg, 0.20 mmol, 25 equiv), and dimethyl sulfone, Me_2_SO_2_ (ca. 1 mg, internal standard) were dissolved in 0.5 mL D_2_O. A 50 μL aliquot was removed for NMR analysis to establish the initial ratio of **dA**:Me_2_SO_2_. To the stirred solution at 70 ± 1 °C (glovebox, degassed oil bath) was added **Ru-1** (0.5 mL of a stock solution of 10.0 mg **Ru-1** in 1.00 mL D_2_O). After 30 min, the stirred solution was quenched with KTp in THF (10 mg/mL; 10 equiv vs starting Ru) and analyzed. NMR spectra of authentic samples of **dA** and the depurination product adenine **A** in D_2_O are shown in Fig. S5.

With ethylene: As above, in a screw-capped vial with a septum through which ethylene was bubbled prior to catalyst injection. Decomposition of **dA** was observed in all cases, to varying extents, but no depurination product **A** was evident under the conditions examined (Table S2; for representative NMR analysis, see Fig. S6).

##### Evaluating cytosine deamination by catalyst

Cytosine **C** (1 mg, 0.009 mmol, 1 equiv), NaCl (11 mg, 0.20 mmol, 20 equiv), and dimethyl sulfone, Me_2_SO_2_ (ca. 1 mg, internal standard) were dissolved in 0.5 mL D_2_O. A 50 μL aliquot was reserved for NMR analysis to establish the initial ratio of **C**:Me_2_SO_2_. To the stirred solution at 70 ± 1 °C (glovebox, degassed oil bath) was added **Ru-1** (0.5 mL of a stock solution of 12.0 mg **Ru-1** in 1.00 mL D_2_O). After 1 h, the stirred solution was quenched with KTp in THF (10 mg/mL; 10 equiv vs starting Ru) and analyzed (NMR). NMR spectra of authentic samples of cytosine **C** and its deamination product, uracil **U**, in D_2_O are shown in Fig. S7.

With ethylene: as above, in a screw-capped vial with a septum through which ethylene was bubbled prior to injecting the catalyst. Deamination product **U** was absent under the conditions examined (Table S3; for representative NMR spectrum, see Fig. S8).

### Binding assay: assessing oligonucleotide adduct formation by Ru-1 and AM

Five different capped nucleic acids (100 pmol): hexa-T, csDNA, native DNA, native RNA and 2′-OMe RNA were incubated with 10 equiv **Ru-1** in the absence of NaCl or with NaCl (400 mM, 40,000 equiv) in H_2_O at pH 5.0 and 25 °C for 10 min. Additionally, native DNA was incubated with 10 equiv **AM** without NaCl, or with NaCl (400 mM, 40,000 equiv) under the same conditions. Reaction mixtures were desalted using ZipTip prior to MALDI-MS analysis.

### Assaying degradation of DNA/RNA backbone during aqueous metatheis using DNA-RNA-FAM 16mer model (Ru-1 vs. AM)

Five hundred pmol of DNA/RNA-FAM (16mer T-FAM, native DNA-FAM, same sequence as **ATGC-1** and native RNA-FAM, same sequence as **purine25-AUGC-1**) was used in all degradation assays. 4 M NaCl, 0.8 mg/mL **AM** and 0.6 mg/mL **Ru-1** stock solutions were prepared in pH 5.0 H_2_O. Prior to the reactions, all stock solutions were filtered via sterile Millex™-GV Filter Unit (pore size 0.22 μm, diam. 33 mm, PVDF membrane). All reactions were performed in a total volume of 50 μL in 1.5 mL Eppendorf tubes. 35 μL H_2_O (pH 5.0), 500 pmol of DNA/RNA, dissolved in 5 μL of water, 5 μL 4 M NaCl stock solution or 5 μL pH 5.0 H_2_O and 5 μL Ruthenium catalyst solutions were added to 1.5 mL Eppendorf tubes. These were briefly flushed with argon, sealed with Parafilm and shaken on an Eppendorf thermocycler at RT and 70 °C for 30 min. The reactions were quenched by adding 10 equiv (vs Ru) SnatchCat (1,4-bis(3-isocyanopropyl) piperazine) and shaking at 37 °C for 30 min. The reactions were analyzed by gel electrophoresis, MALDI-MS, and analytical reverse-phase (RP)-HPLC. *Method*. Linear gradient of 5–50% MeOH within 15 min, then 50% to 100% MeOH within 1 min, followed by 100% MeOH for 2 min, then 100% to 5% MeOH within 1 min, followed by 5% MeOH for 2 min. HPLC chromatograms were recorded at 260, 280, and 495 nm wavelengths. Before MALDI-MS measurements, the samples were desalted using ZipTip pipette tips according to the manufacturer´s protocol. Gel electrophoresis and band quantification was assessed using ImageJ software.

## Supplementary information

Supplementary information

Transparent Peer Review File

## Data Availability

All data generated in this study are provided within the article and in the Supplementary Information file, or available from the corresponding authors on request: experimental procedures for synthesis of oligonucleotide conjugates and macrocycles, materials and methods, spectra (NMR, UV–Vis, and mass spectrometry), chromatograms.
