# Supplementary material for: On-oligonucleotide olefin metathesis in water
Source: Nat Commun. 2026 May 12;17:6329. doi: 10.1038/s41467-026-72995-4 (PMC13377165; doi:10.1038/s41467-026-72995-4)
Supplement: Supplementary file 1 — Supplementary information [file 41467_2026_72995_MOESM1_ESM.pdf]

Supplementary Information

on-Oligonucleotide Olefin Metathesis in Water

Chun Zhang[1], Christian O. Blanco[2], Anastasiya Khimich[1], Deryn E. Fogg[2,3]* and Andreas Brunschweiger[1]*

[1]Institute of Pharmacy and Food Chemistry, Julius-Maximilians-University Würzburg, 97074 Würzburg, Germany. [2]Center for Catalysis Research & Innovation, and Department of Chemistry and Biomolecular Sciences, University of Ottawa, Ottawa, Ontario, K1N 6N5, Canada.[3]Department of Chemistry, University of Bergen, N-5007 Bergen, Norway.

These authors contributed equally: Chun Zhang, Christian O. Blanco

Table of Contents

1. **Abbreviations and definitions** **2**
2. **Materials and instruments** **3**
3. **Structures of DNAs and RNAs** **4**
4. **Representative procedures for synthesis of DNA and RNA conjugates** **6**
5. **DNA ligation and Sanger sequencing** **8**
6. **Impact of oligonucleotide constituents on aqueous metathesis.** **9**
7. **Assessing catalyst stability to DNA.** **10**
8. **Binding Assay: Assessing oligonucleotide adduct formation by Ru-1-and AM** **15**
9. **Assaying degradation of DNA/RNA backbone during aqueous metathesis using DNA/RNA-FAM 16mer model (Ru-1 vs. AM).** **27**
10. **Comparison of different RCM protocols** **53**
11. **Optimizing conditions for RCM of hexa-T-tagged substrates (hexa-T-1) in H_2O** **65**
12. **DNA precipitation assays** **70**
12. **Substrate scope of hexa-T-tagged macrocycles (hexa-T-2´-7´)** **84**
13. **RCM of a mixture of 4 hexa-T-tagged substrates to demonstrate DEL feasibility** **91**
14. **Optimizing conditions for RCM of csDNA-tagged dienes (7DeATC-1) in H_2O.** **94**
15. **Optimizing conditions for RCM of DNA-tagged substrates (ATGC-1) in H_2O** **98**
16. **Synthesis of RNA-tagged macrocycle (purine25-AUGC-1´) via RCM in H_2O** **106**
17. **Synthesis of 2'-O-methyl-RNA-tagged macrocycle via RCM in H_2O** **110**
18. **Tolerance to different purine content in DNA/RNA oligomers and dsDNA headpiece in optimized RCM conditions in water** **113**
19. **DNA damage assessment** **131**
20. **Supplementary references** **132**

1. Abbreviations and definitions

2´-OMe RNA: 2´-O-methyl-ribonucleic acid
3-HPA: 3-hydroxypropionic acid
ACN: acetonitrile
AM: AquaMet
AMA: aqueous ammonia/aqueous methylamine
ATP: adenosine triphosphate
Boc: *tert*-butyloxycarbonyl
CH_2Cl_2: dichloromethane
CPG: controlled pore glass
csDNA: chemically stabilized deoxyribonucleic acid
DEL: DNA-encoded library
DIPEA: *N*,*N*-diisopropylethylamine
DMF: *N*,*N*-dimethylformamide
DMSO: dimethyl sulfoxide
DMT: dimethoxytrityl
DTT: dithiothreitol
EDTA: ethylenediaminetetraacetic acid
equiv: equivalent(s)
Fmoc: fluorenylmethyloxycarbonyl
HATU: O-(7-azabenzotriazol-1-yl)-N,N,N',N'-tetramethyluronium hexafluorophosphate
hexa-T DNA: hexathymidine deoxyribonucleic acid
KTp: potassium tris(pyrazolyl) borate
m/z: mass-to-charge ratio
MALDI-MS: matrix-assisted laser desorption/ionization-mass spectrometry
Me_2SO_2: methylsulfonylmethane
MeOH: methanol
MMT: monomethoxytrityl
NEt_4Cl: tetraethylammonium chloride
$NHEt_3Cl$: triethylammonium chloride
$NHMe_3Cl$: trimethylammonium chloride
NMe_4Cl: tetramethylammonium chloride
NMR: nuclear magnetic resonance
N^nBu_4Cl: tetrabutylammonium chloride
Phos: 5´-phosphorylation
RCM: ring-closing metathesis
RP-HPLC: reverse-phase high performance liquid chromatography
RT: room temperature
SnatchCat: 1,4-bis(3-isocyanopropyl) piperazine
T4 PNK: T4 polynucleotide kinase
TBE: tris/borate/EDTA
THF: tetrahydrofuran
THPTA: tris(3-hydroxypropyltriazolylmethyl)amine
Tris-HCl: tris(hydroxylmethyl)aminomethane hydrochloride
UV-vis: ultraviolet-visible

Definitions for RCM reactions: in situ yield, yield of oligonucleotide-tagged cyclic product prior to isolation; isolated yield, yield of product isolated by HPLC; total DNA/RNA recovery, sum of isolated product and unreacted starting material isolated by HPLC.

2. Materials and instruments

Reagents. Unless otherwise noted, chemicals were purchased from Sigma-Aldrich (Taufkirchen, Germany), Thermo Fisher (Karlsruhe, Germany), and VWR (Langenfeld, Germany). *t*Boc-*N*-amido-PEG(4)-azide was purchased from BroadPharm (San Diego, CA). Phosphate buffer (Merck), 2-(N-morpholino)ethanesulfonic acid (MES, Merck), HEPES buffer (Merck), T4 DNA ligase (3 U/µL) was purchased from New England Biolabs (Frankfurt am Main, Germany). All DNAs for Sanger sequencing were synthesized by Sigma-Aldrich. LLC (Munich, Germany). Sanger sequencing was performed by Eurofins Genomics (Ebersberg, Germany), All DNA ligation reactions were performed in an Eppendorf Mastercycler (Hamburg, Germany). GeneRuler Ultra Low Range DNA marker (Thermo Fisher) and ligation products were run using 12% polyacrylamide gel electrophoresis and stained with SYBR™ Gold (Thermo Fisher). Gel documentation was done with GelDoc Go Gel imaging system from Bio-Rad (Hercules, CA). The 16mer native DNA, 7-deazaA chemically modified DNA, hexa-T DNA oligonucleotide, 16mer RNA and 16mer 2´-OMe RNA attached to controlled pore glass solid phase (CPG, 1,000 Å) were synthesized by Ella Botech GmbH (Fürstenfeldbruck, Germany). Controlled pore glass solid phase was filtered on a synthesis column plugged onto a vacuum manifold (Vac-Man®, Promega). Commercially available anhydrous solvents (MeCN, CH_2Cl_2, DMF, MeOH) were used as received. Metathesis catalysts **GIII**[1], **DA**[2], and **Ru-1**[3] (Chart S1) and model substrate 2,2-diallylpropane-1,3-diol **1**[4], were prepared by literature methods. MilliQ H_2O, and D_2O (Cambridge Isotopes, 99.5%) were freeze-pump-thaw degassed (4×) and stored under N_2 in the glovebox. Dimethyl sulfone (Me_2SO_2, 98%; internal standard for NMR analysis), SnatchCat (quenching agent, Sigma, 95%), potassium trispyrazolyl borate (KTp; quenching agent;[5] Sigma, 98%), and **AM** (Sigma, >95%) were used as received. The purity of all catalysts was confirmed by ^1H NMR analysis prior to use. For accuracy, all catalysts and solid reagents were weighed in air using a microanalytical balance.

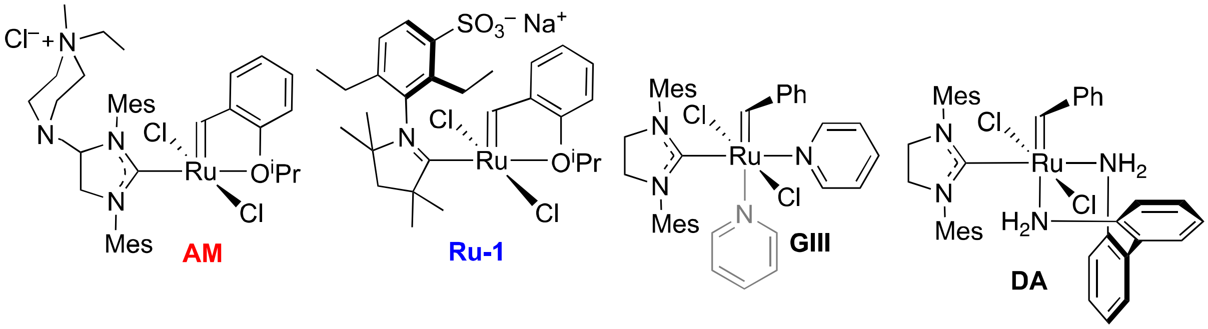

Figure S1. Catalysts employed.

NMR analysis. NMR spectra were recorded on Bruker Avance 300 and 400 NMR spectrometers at 25 ±0.5 °C. Chemical shifts (ppm) are referenced to the residual proton of the deuterated solvent for ^1H NMR spectra (D_2O: 4.79 ppm; CD_3OD: 3.31 ppm; $CDCl_3$: 7.26 ppm; DMSO-d_6: 2.50 ppm). Quantitative NMR experiments were used to quantify catalysis of 1,3-diol **1**, using a standard delay time (D1) of 30 seconds. UV-vis spectra of catalysts were measured with a Mettler-Toledo Easy UV spectrophotometer.

Analytical RP-HPLC. Oligonucleotide-small molecule conjugates were analyzed by ion-pair reverse-phase high-performance liquid chromatography (RP-HPLC, Agilent 1260 Infinity II) using a C_{18} stationary phase (Phenomenex, Gemini; 5 µm, C18, 110 Å, 100*4.6 mm). A gradient from 100 mM aqueous triethylammonium acetate (pH = 7.0, eluent A) to MeOH (eluent B) was used at a flow rate of 1 mL/min.

Method. Linear gradient of 20% to 80% B within 18 min, then 80% to 100% B within 1 min, followed by 100% B for 2 min, then 100% to 20% B within 1 min, followed by 20% B for 2 min. HPLC chromatograms were recorded at 260 and 280 nm wavelengths.

Semi-preparative RP-HPLC. Oligonucleotide-small molecule conjugates were purified by semi-preparative RP-HPLC (Agilent 1260 Infinity II) using a C_{18} stationary phase (Phenomenex, Gemini; 5 µm, C18, 110 Å, 100*10 mm). A gradient from 100 mM aqueous triethylammonium acetate (pH = 7.0, eluent A) to MeOH (eluent B) was used at a flow rate of 4 mL/min.
Method. Linear gradient of 20% to 80% B within 18 min, then 80% to 100% B within 1 min, followed by 100% B for 2 min, then 100% to 20% B within 1 min, followed by 20% B for 2min. HPLC chromatograms were recorded at 260 and 280 nm wavelengths. Fractions containing the desired products were concentrated using a SpeedVac evaporator.

Oligonucleotide concentrations. Oligonucleotide concentrations were determined by UV spectroscopy using a spectrophotometer (NanoPhotometer N120, Implen, Germany) and Qubit™ Flex Fluorometer (Thermo Fisher).

MALDI-TOF. Oligonucleotides were analyzed by MALDI-MS (Bruker UltrafleXtreme) using 3-hydroxypropionic acid (3-HPA) matrix (Dichrom).

3. Structures of DNAs and RNAs

hexa-T, DNA, csDNA, RNA and 2´-OMe RNA were synthesized by Ella Biotech (Germany). DNA and csDNA remained uncleaved on CPG beads with 5´-DMT-on. hexa-T, DNAs, RNAs and 2´-OMe RNA with 5´-amino C6 linker remained uncleaved on CPG beads with 5´-MMT-on.

16mer 7-deaza deoxyadenosine chemically modified DNA (7DeATC-DNA)
5´-CXCTCCTCCATCACCT-3´ (X, 5-Ethynyl-dU-CEP, A, 7-deaza dA)

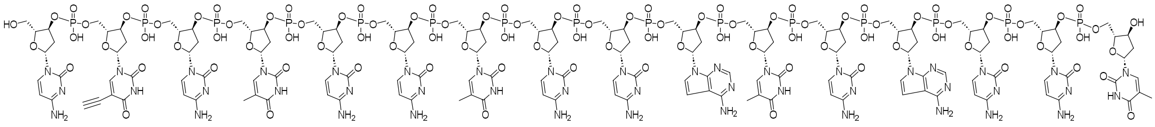

16mer native DNA (ATGC-DNA)
5´-CXCTCGATTCGCACCT-3´

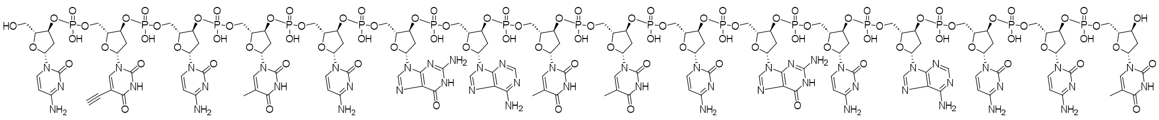

16mer native RNA with 25% purine content (Purine25-AUGC RNA)
5´-Amino-C6-CUCUCGAUUCGCACCU-3´

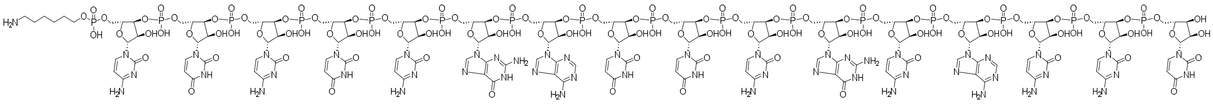

16mer 2´-OMe RNA (2´-OMe AUGC-RNA)
5´-Amino-C6-CUCUCGAUUCGCACCU-3´

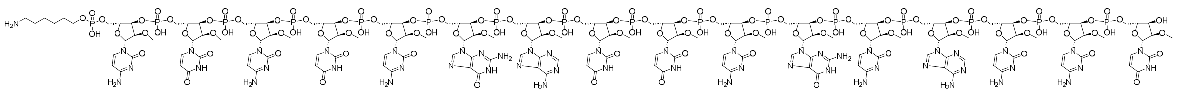

Hexathymidine (hexa-T-DNA)
5´-Amino-C6-TTTTTT-3´,

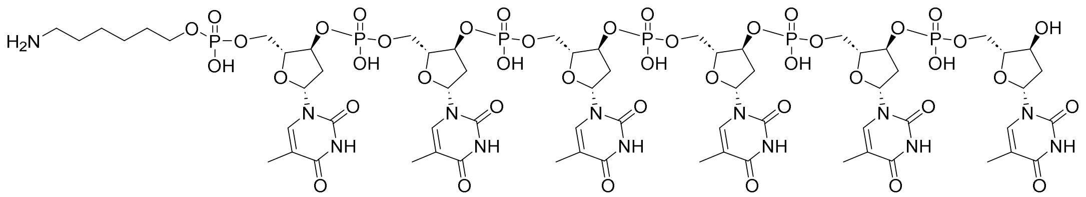

DNA and RNA sequences used in sequence scope experiments

16mer native RNA with 50% purine content (AUGC RNA)
5´-Amino-C6-CUGACGAUACGCACGU-3´

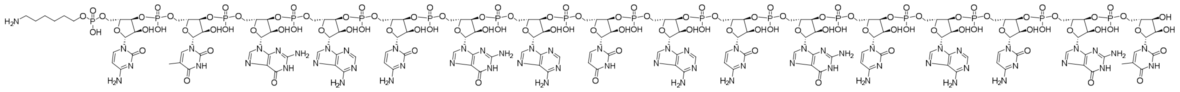

16mer native RNA with 75% purine content (Purine75-AUGC RNA)
5´-Amino-C6-AUGAAGAUACGGACGA-3´

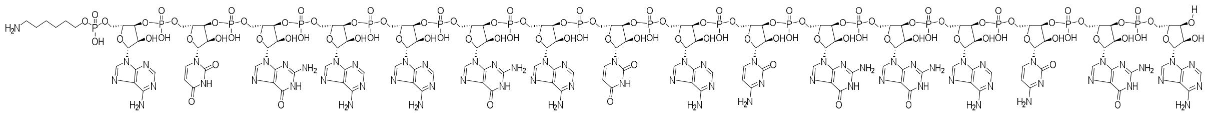

16mer native DNA with 25% purine content (purine25-ATGC DNA)
5´-Amino-C6-CTCTCAATTCACACCT-3´

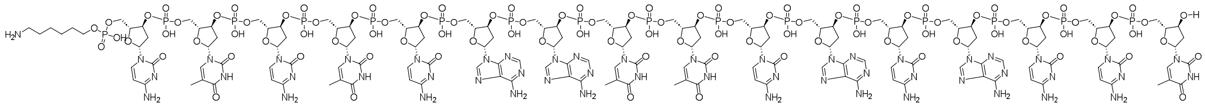

16mer native DNA with 75% purine content (purine75-ATGC DNA)
5´-Amino-C6-AAAACAATAAACACAA-3´

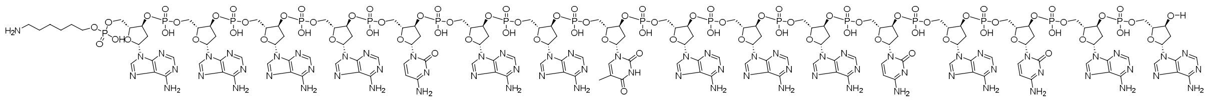

dsDNA headpiece (headpiece DNA)
5´-Phos-GAGTCAXZXTGACTCCC (X=Spacer 9, Z=C7 amino linker)

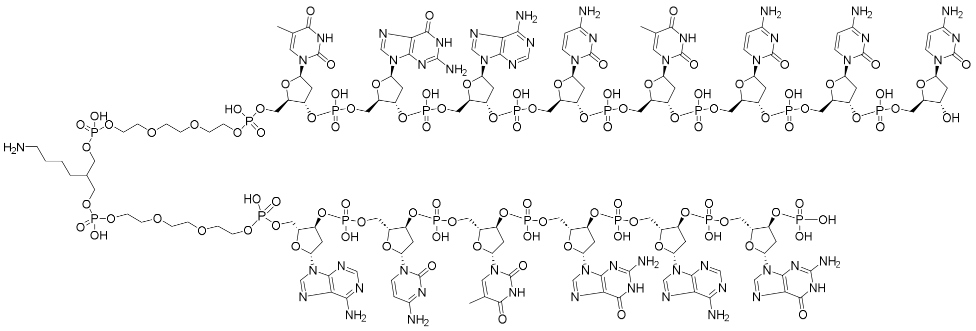

Figure S2. Sequences and structures of all DNA and RNA oligonucleotides.

4. Representative procedures for synthesis of DNA and RNA conjugates

Copper (I)-promoted alkyne-azide cycloaddition

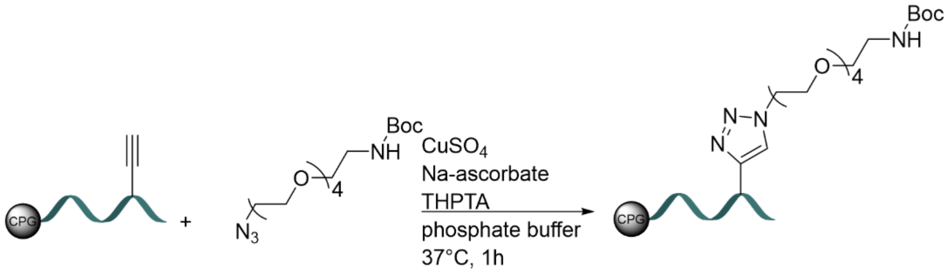

The CPG-bound oligonucleotide-alkyne conjugate (350 nmol, 1 equiv) was washed with 300 µL H_2O. The aqueous catalyst mixture (composed of 21 µL H_2O, 1.75 µL THPTA (100 mM, 0.500 equiv), 26.25 µL sodium ascorbate (100 mM, 7.5 equiv), and 0.875 µL $CuSO_4{\cdot}5H_2O$ (100 mM, 0.25 equiv); 50 µL total volume) was shaken at RT for 10 min. The aqueous catalyst mixture was added to the CPG beads along with *t*-Boc-*N*-amido-PEG4-azide (1 mg in 75 µL DMSO, 2800 nmol, 8 equiv), phosphate buffer (Dulbecco´s Phosphate Buffered saline, 30 µL, 10×) and H_2O (145 µL). The reaction mixture was shaken at 37 °C for 1 h on an Eppendorf Thermocycler. The CPG-bound conjugate was then passed through a filter column and washed with 200 µL of 0.1 M $EDTA_{(aq)}$, water, DMF, MeOH, MeCN and CH_2Cl_2 (3× each) and dried under vacuum. The procedure was repeated once only, to maximize conversion.

Boc Deprotection: The Boc group was cleaved from the PEG linker by shaking the CPG-coupled oligonucleotide in 10% v/v trifluoroacetic acid in CH_2Cl_2 (200 µL) at RT for 10 min and repeating 3×.

Fmoc Deprotection: The Fmoc group was cleaved from CPG-coupled oligonucleotides by shaking the beads in 20% v/v piperidine in DMF (200 µL) at RT for 5 min and repeating 3×.

DMT (MMT)-deprotection: The DMT/MMT-protecting group of CPG-coupled DNA was cleaved by suspending the beads in 3% v/v trichloroacetic acid in CH_2Cl_2 (200 µL) at RT for 1 min. Development of an orange colour in the solution indicated successful removal of the protecting group. Deprotection was repeated until the solution remained colourless. CPG-bound deprotected DNA was washed (3× each) with 200 µL of 1% NEt_3 in MeCN, DMF, MeOH, MeCN and CH_2Cl_2 and then dried in vacuo.

<u>With RNA</u>: As above, using 3% v/v dichloroacetic acid in CH_2Cl_2.

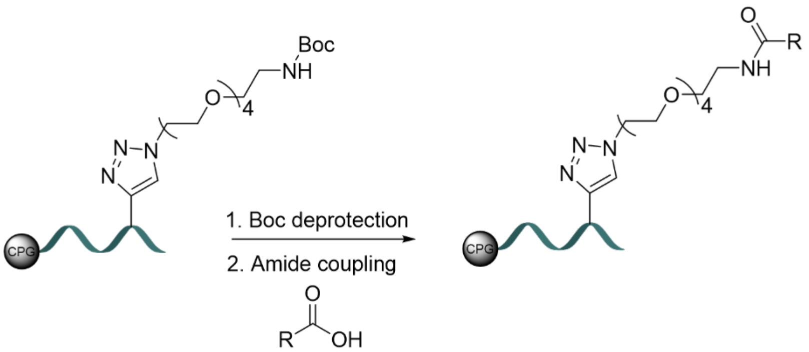

Amide coupling reaction (General Procedure 1): CPG-bound oligonucleotides, carboxylic acids and HATU were dried *in vacuo* for 15 min. Stock solutions of all reactants in dry DMF were prepared fresh before the reaction was started. To the solution of the carboxylic acid (100 equiv) in 160 µL dry DMF were added HATU (100 equiv) dissolved in 40 µL dry DMF, and DIPEA (250 equiv). The mixture was shaken at 37 °C for 10 min and added to the CPG-bound oligonucleotides, and further shaken at 37 °C for 1 h. Then, the CPG-bound oligonucleotides were filtered over a filter column, washed with each 3× 200 µL of DMF, MeOH, ACN and CH_2Cl_2 and dried under vacuum. The amide coupling was repeated on the CPG-bound oligonucleotides twice to maximize conversion.

Microcleavage for assessing conversion: A minimum amount of CPG beads was picked up with a pipette plastic tip and transferred to 50 µL of AMA solution (aqueous ammonia (30%)/ aqueous methylamine (40%), 1:1, vol/vol). The suspension was shaken at 65 °C for 10 min, the mixture was dried in a SpeedVac, and the oligonucleotides were dissolved in 50 µL Millipore H_2O for analysis by MALDI-MS and analytical RP-HPLC. In case of incomplete reactions (<90%), the reaction on the CPG-bound oligonucleotides was repeated.

Capping after amide coupling reactions: Unreacted amines were capped with capping solution (1:1 mixture of THF/methylimidazole, 9:1 v/v, and THF/pyridine/acetic acid anhydride 8:1:1 v/v). The CPG was treated with 200 µL of the capping solution (3×; 30 s each). The capped CPG-bound oligonucleotide was washed with 200 µL of DMF, MeOH, MeCN and CH_2Cl_2 (3× each) and dried in vacuo.

Synthesis of oligonucleotide precursors for RCM reaction

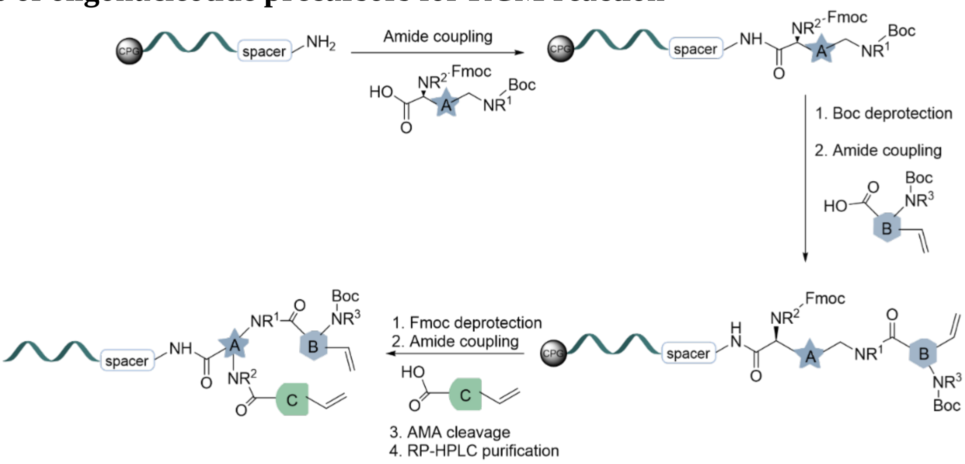

Figure S3. Synthesis of oligonucleotide diolefin conjugates for RCM reactions. For RNAs, Fmoc- and MMT-protected diamine carboxylic acids were used in amide coupling reactions.

RCM of oligonucleotide-olefin conjugates in H_2O (General Procedure 2)

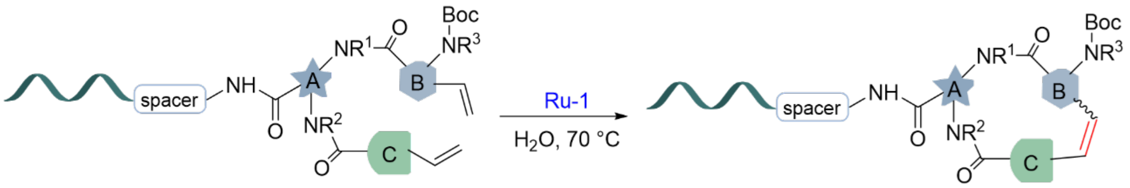

RCM reactions were carried out with 500 pmol of DNA or RNA oligomers. Aqueous solutions at pH 2.0 and pH 5.0 were prepared by acidifying Millipore H_2O with 1 M HCl. pH 5.0 phosphate buffer (75 mM sodium phosphate dibasic and 25 mM sodium phosphate monobasic) was prepared by acidifying the commercial stock solution with 1 M HCl, pH 5.0 HEPES buffer (100 mM) was prepared by diluting 1 M stock solution and by acidifying with 1 M HCl, pH 5.0 MES buffer (100 mM) was prepared by dissolving 639.75 mg in 30 mL Millipore H_2O, followed by basifying by 1 M NaOH. Stock solutions of 4 M NaCl and 4 M $NHEt_3Cl$ and 0.6 mg/mL **Ru-1** were prepared in pH 2.0 and pH 5.0 H_2O, respectively. Prior to RCM reactions, all stock solutions were filtered via sterile Millex™-GV Filter Unit (pore size 0.22 µm, 33 mm diameter, PVDF membrane). All RCM reactions were performed in a total volume of 50 µL in 1.5 mL Eppendorf tubes. To the tubes were added 5 µL oligonucleotide, 5 µL 4 M NaCl and 5 µL 4 M $NHEt_3Cl$ solutions (40,000 equiv NaCl and $NHEt_3Cl$), 5 µL **Ru-1** solution, following which pH 2.0 or pH 5.0 H_2O was added to reach a final volume of 50 µL. The solution was flushed with argon, sealed with Parafilm and shaken on an Eppendorf thermocycler at the temperature and time specified (typically 70 °C, 30 min). The reactions were quenched by adding SnatchCat (1,4-bis(3-isocyanopropyl) piperazine; 10 equiv vs Ru) and shaking at 37 °C for 30 min. The reactions were analyzed by MALDI-MS and analytical RP-HPLC. Samples were desalted prior to MALDI-MS analysis using ZipTip pipette tips according to the manufacturer protocol.

5. DNA ligation and Sanger sequencing

5´-Phosphorylation of DNA (General Procedure 3)

For 5'-phosphorylation of DNA in a total reaction volume of 20 µL, 10 units of T4 polynucleotide kinase (T4 PNK, Thermo Fisher), 1x PNK Buffer A (50 mM Tris-HCl, 10 mM $MgCl_2$, 5 mM DTT, 0.1 mM spermidine, pH = 7.6 at 25 °C, Thermo Fisher) and 1 mM ATP (Thermo Fisher) were used. Reaction mixtures were incubated at 37 °C for 20 min, then heat-inactivated at 75 °C for 15 min and slowly cooled to 4 °C.

Ligation of DNA (General Procedure 4)

Prior to enzymatic ligation of DNA, the oligonucleotides were annealed by incubation at 65 °C for 10 min, then slowly cooled down to 4 °C. For ligation (40 µL scale), 100 pmol of each oligonucleotide, 600 units of T4 DNA Ligase (T4 DNA ligase, New England Biolabs) and 1x T4 DNA Ligase Buffer (50 mM Tris-HCl, 10 mM $MgCl_2$, 10 mM DTT, 1 mM ATP, pH = 7.5 at 25 °C, New England Biolabs) were mixed. Ligation reactions were performed at 25 °C for 16 h, then stopped by heat inactivation at 75 °C for 15 min and cooled down to 4 °C.

Analysis of DNA ligation (General Procedure 5)

DNA ligation reactions were analyzed by gel electrophoresis (12% polyacryamide). Electrophoresis was carried out in TBE buffer (89 mM tris/borate, 2 mM EDTA, pH 8.3) at 120 V constant voltage for 45 min. The DNA ligation products were stained using SYBR™ Gold (Thermo Fisher) with GeneRuler Ultra Low Range DNA Ladder (Thermo Fisher) as a reference. Gel imaging was performed using the Bio-Rad GelDoc Go Gel imaging system.

Purification of DNA ligation product by E-Gel™ Power Snap Electrophoresis System
The ligation products of native and csDNA were purified by 2% E-Gel™ SizeSelect™ II Agarose Gels (Thermo Fisher) using E-Gel™ Power Snap Electrophoresis System (Thermo Fisher). The purification was performed in accordance with the manufacturer's protocol.

Sanger sequencing
Sanger sequencing of purified ligation products was performed by Eurofins Genomics. The sample preparations were carried out according to the TubeSeq Supreme protocol.

6. Impact of oligonucleotide constituents on aqueous metathesis.

Representative RCM Reaction with Diene 1. A stock of substrate solution was prepared by dissolving diene **1** (32 mg, 0.10 mmol), NaCl (48 mg, 0.82 mmol, 820 equiv), and dimethyl sulfone, Me_2SO_2 (ca. 5 mg, internal standard) in 4 mL D_2O; final concentration 100 mM **1**. A 500 µL aliquot was reserved for NMR analysis to establish the initial ratio of **1**: Me_2SO_2. For each reaction, 500 µL of diene **1** stock solution and 30 µL of the additive (7.5 mM stock solution of nucleosides **dA**, **dG**, **dC**, **dT**, or **Urd**) were added; one reaction was run without additive (control). To the stirred solutions at 70±1 °C (glovebox, degassed oil bath) was added 1.0 mol% **Ru-1** (85 µL of a stock solution of 2.0 mg **Ru-1** in 1.00 mL D_2O). After 2 h, the solutions were quenched with KTp in THF (10 mg/mL; 10 equiv vs starting Ru) and analyzed (NMR). Table S1 shows conversions of **1** and yields of **1'** (from the 2H olefinic signal for **1'** at 5.73 ppm; Figure S4, assigned by analogy to the reported signal in $CDCl_3$ at 5.65 ppm).[6]

Table S1. Impact of nucleosides on the RCM of diol **1**.

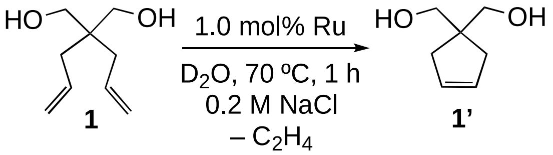

Catalyst	Additive	Yield 2' (%)	Drop
**Ru-1**	None	96	–
**Ru-1**	**dA**	50	46
**Ru-1**	**dG**	84	12
**Ru-1**	**dC**	81	15
**Ru-1**	**dT**	85	11
**Ru-1**	**Urd**	86	10
**AM**	None	89	–
**AM**	**dA**	30	59
**AM**	**dG**	24	65
**AM**	**dC**	43	46
**AM**	**dT**	28	61
**AM**	**Urd**	52	37

[a]Numerical data for **Figure 3** in main text.

7. Assessing catalyst stability to DNA.

Stability of Ru-1 in the Presence of Native DNA. To a screw-capped quartz cuvette with a septum seal was added H_2O (1.5 mL, pH = 5 prior to catalyst addition) and solid NaCl (95 mg, 1.62 mmol, 40,000 equiv). The cuvette was sealed, wrapped with Parafilm and removed from the glovebox to the spectrometer to measure the blank read. An aliquot of a stock solution of **Ru-1** in water (14 µL, 41 nmol, 2.0 mg/mL), was injected via gas-tight syringe (puncture promptly covered with Parafilm) to give a final Ru concentration of 27.6 µM. The first UV-vis spectrum was taken 5 min after preparing the catalyst stock solution. An aliquot of a stock solution of the amide-capped native DNA **Nat-0** (80 µL, 0.51 nmol/µL, 1 equiv) was injected. Spectra were recorded periodically up to 2 h. UV-vis spectra showing the stability of **Ru-1** to various oligonucleotides appear in Figure 4 in the main text.

Evaluating Deoxyadenosine Depurination by Ru Catalyst. Deoxyadenosine **dA** (2 mg, 0.008 mmol, 1 equiv), NaCl (11 mg, 0.20 mmol, 25 equiv), and dimethyl sulfone, Me_2SO_2 (ca. 1 mg, internal standard) were dissolved in 0.5 mL D_2O. A 50 µL aliquot was removed for NMR analysis to establish the initial ratio of **dA**:Me_2SO_2. To the stirred solution at 70±1 °C (glovebox, degassed oil bath) was added **Ru-1** (0.5 mL of a stock solution of 10.0 mg **Ru-1** in 1.00 mL D_2O). After 30 min, the stirred solution was quenched with KTp in THF (10 mg/mL; 10 equiv vs starting Ru) and analyzed. NMR spectra of authentic samples of **dA** and the depurination product adenine **A** in D_2O are shown in Figure S5.

With ethylene: As above, in a screw-capped vial with a septum through which ethylene was bubbled prior to catalyst injection. Decomposition of **dA** was observed in all cases, to varying extents, but no depurination product **A** was evident under the conditions examined (Table S2; for representative NMR analysis, see Figure S6).

Table S2. Impact of catalyst on depurination of **dA**.

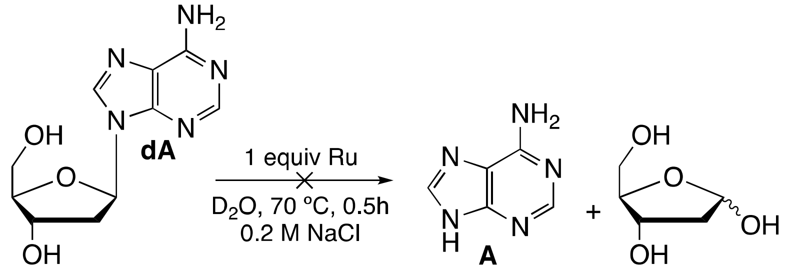

Catalyst	Additive	% dA	% A
**AM**	-	3	0
**AM**	Ethylene	0	0
**Ru-1**	-	43	0
**Ru-1**	Ethylene	82	0

Evaluating Cytosine Deamination by Catalyst. Cytosine **C** (1 mg, 0.009 mmol, 1 equiv), NaCl (11 mg, 0.20 mmol, 20 equiv), and dimethyl sulfone, Me_2SO_2 (ca. 1 mg, internal standard) were dissolved in 0.5 mL D_2O. A 50 µL aliquot was reserved for NMR analysis to establish the initial ratio of **C**:Me_2SO_2. To the stirred solution at 70±1 °C (glovebox, degassed oil bath) was added **Ru-1** (0.5 mL of a stock solution of 12.0 mg **Ru-1** in 1.00 mL D_2O). After 1 h, the stirred solution was quenched with KTp in THF (10 mg/mL; 10 equiv vs starting Ru) and analyzed (NMR). NMR spectra of authentic samples of cytosine **C** and its deamination product, uracil **U**, in D_2O are shown in Figure S7.

With ethylene: As above, in a screw-capped vial with a septum though which ethylene was bubbled prior to injecting catalyst. Deamination product **U** was absent under the conditions examined (Table S3; for representative NMR spectrum, see Figure S8).

Table S3. Catalyst impact on the deamination of cytosine.

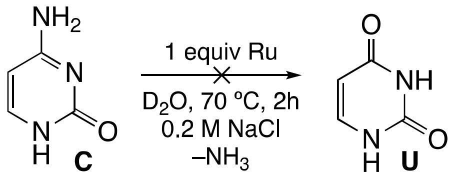

Catalyst	Additive	%C	%U
**AM**	None	99	0
**AM**	Ethylene	99	0
**Ru-1**	None	55[a]	0
**Ru-1**	Ethylene	70[a]	0

[a]Partial precipitation observed, likely causing loss of starting material and incomplete mass balance. No formation of uracil was observed in any of the four experiments. The compatibility of cytosine with Ru-1 was later confirmed by DNA sequencing experiments.

NMR Spectra and Chromatograms.

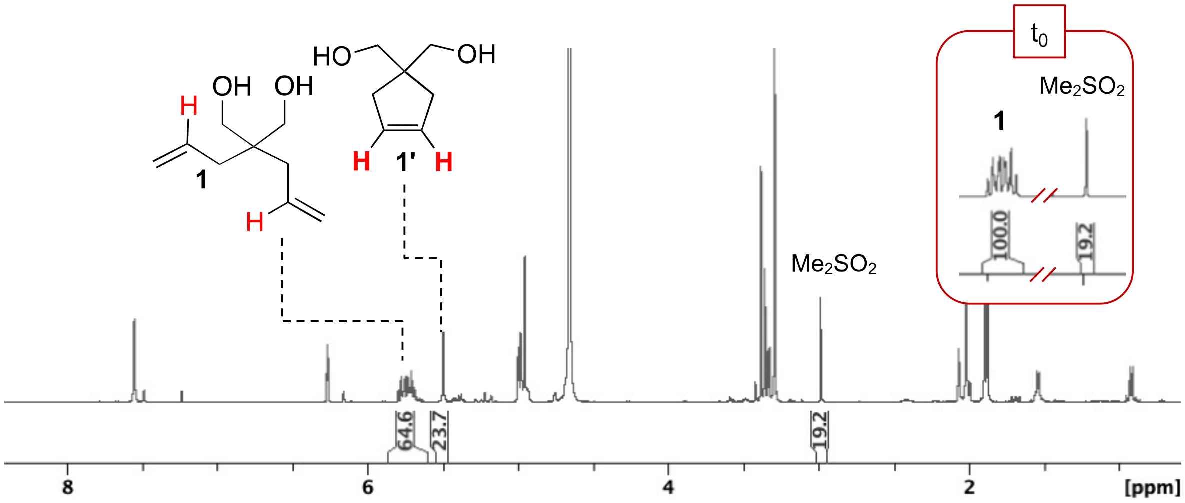

Figure S4. Quantifying RCM of **1** by **AM** (1.0 mol%). Representative ^1H NMR spectrum (400 MHz, D_2O) after RCM in the presence of **dG**. Internal standard (IS) = dimethylsulfone, Me_2SO_2.

(a) Deoxyadenosine

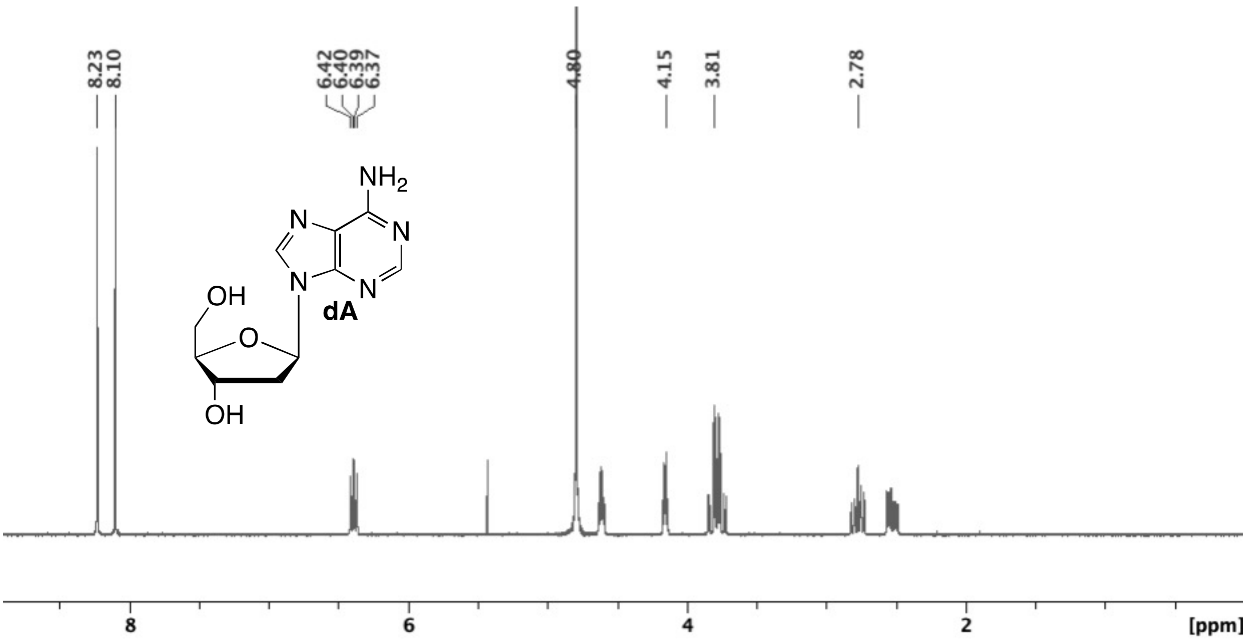

(b) Adenine

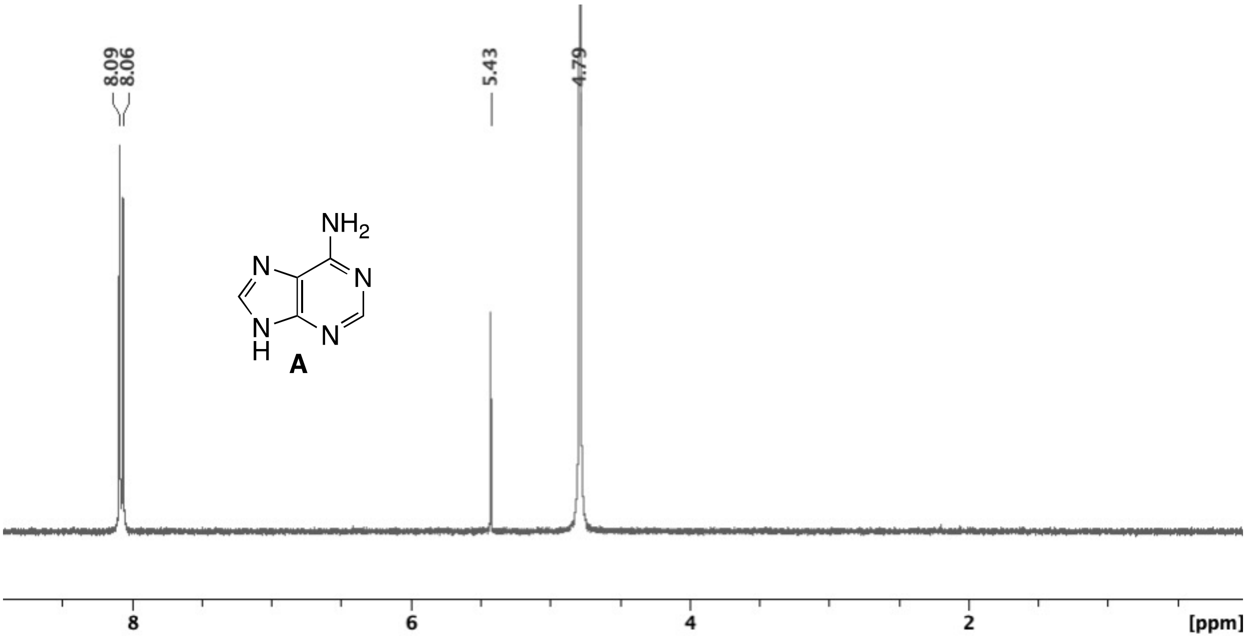

Figure S5. ^1H NMR spectra (300 MHz, D_2O) of authentic samples of (a) deoxyadenosine **dA** and (b) adenine **A**.

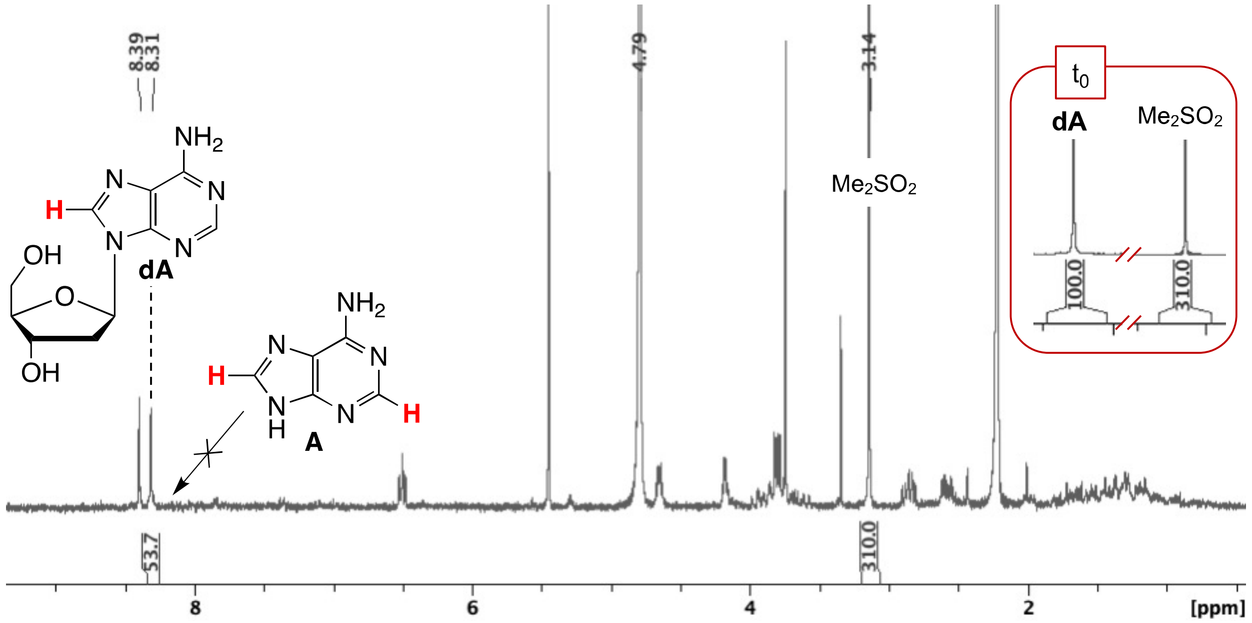

Figure S6. Representative ^{1}H NMR spectrum (300 MHz, D_2O) showing no depurination after exposure of **dA** to **Ru-1**. Internal standard = dimethylsulfone, Me_2SO_2.

(a) Cytosine

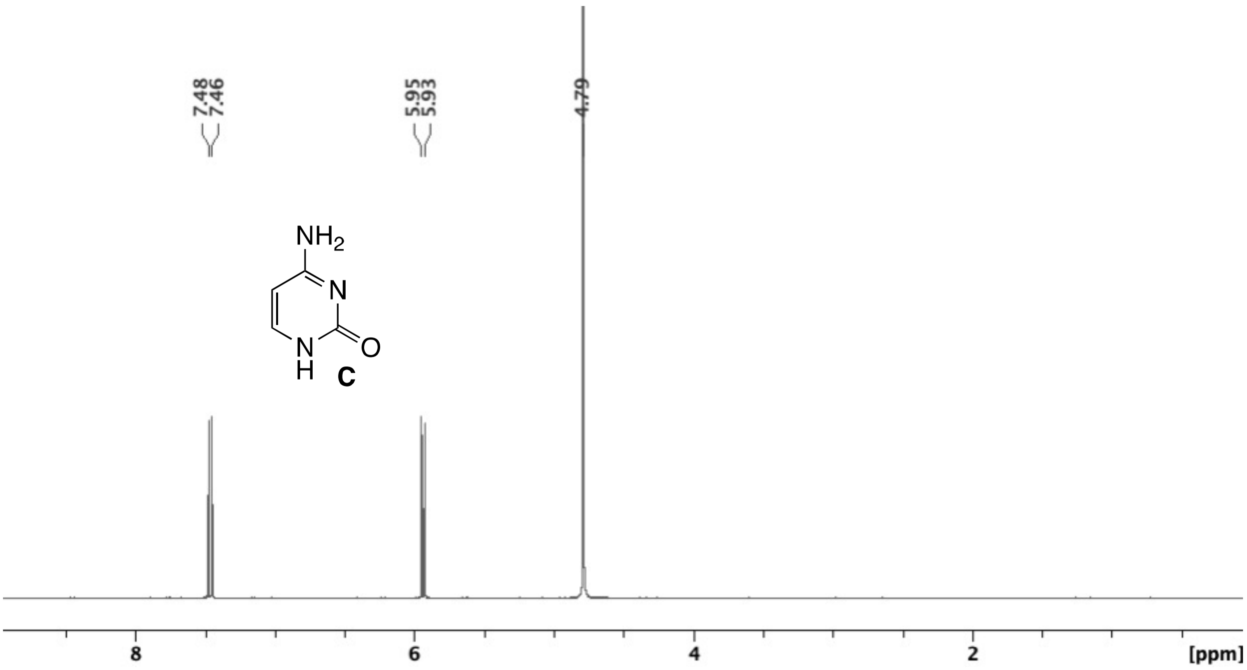

(b) Uracil

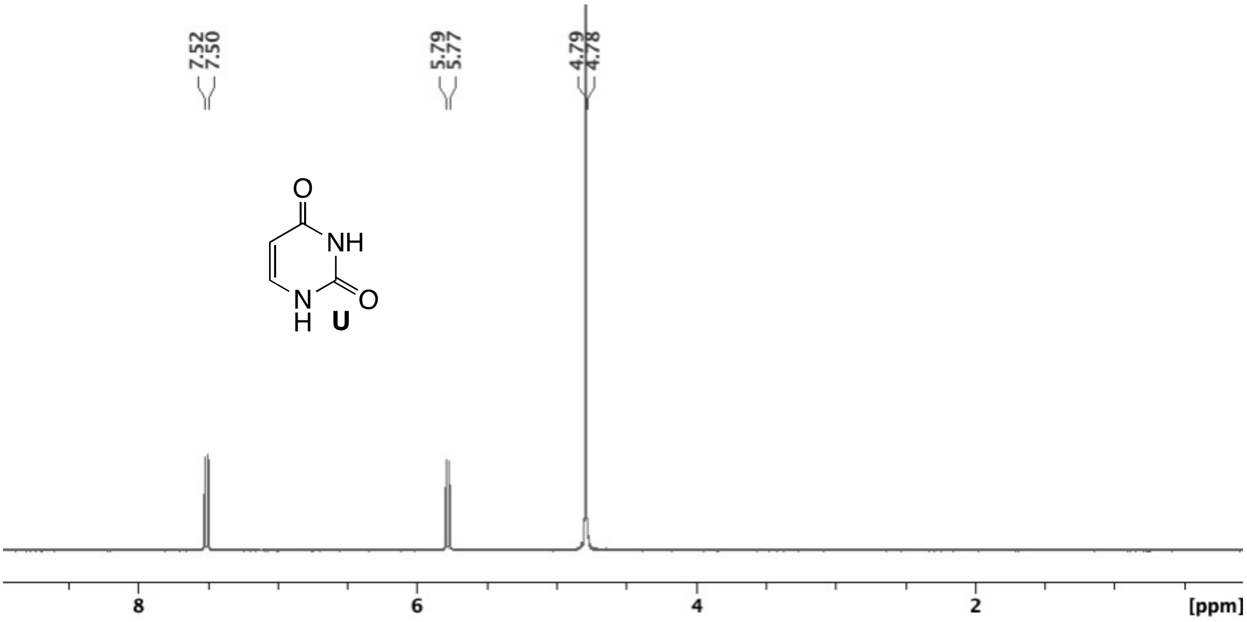

Figure S7. ^{1}H NMR spectra (300 MHz, D_2O) of authentic samples of (a) cytosine **C** and (b) uracil **U**.

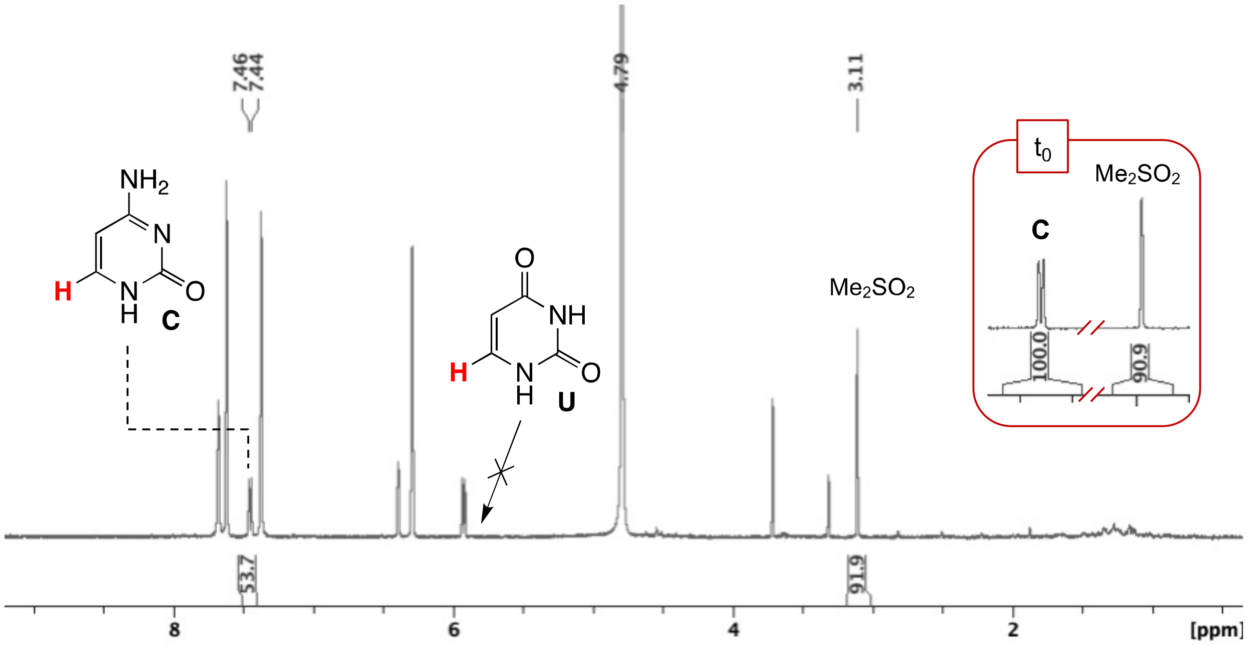

Figure S8. Representative ^{1}H NMR spectrum (300 MHz, D_2O) showing no deamination after exposing **C** to **Ru-1**. Internal standard = dimethylsulfone, Me_2SO_2.

8. Binding Assay: Assessing oligonucleotide adduct formation by Ru-1-and AM

5 different capped nucleic acids (100 pmol): hexa-T, csDNA, native DNA, native RNA and 2´-OMe RNA were incubated with 10 equiv **Ru-1** in the absence of NaCl, or with NaCl (400 mM, 40 ,000 equiv) in H_2O at pH 5.0 and 25 °C for 10 min. Additionally, native DNA was incubated with 10 equiv **AM** without NaCl, or with NaCl (400 mM, 40 ,000 equiv) under the same conditions. Reaction mixtures were desalted using ZipTip prior to MALDI-MS analysis. Spectra are shown below.

MALDI-MS Data: Assaying binding of **AM**

Native DNA (calculated m/z 5083, AM, no NaCl)

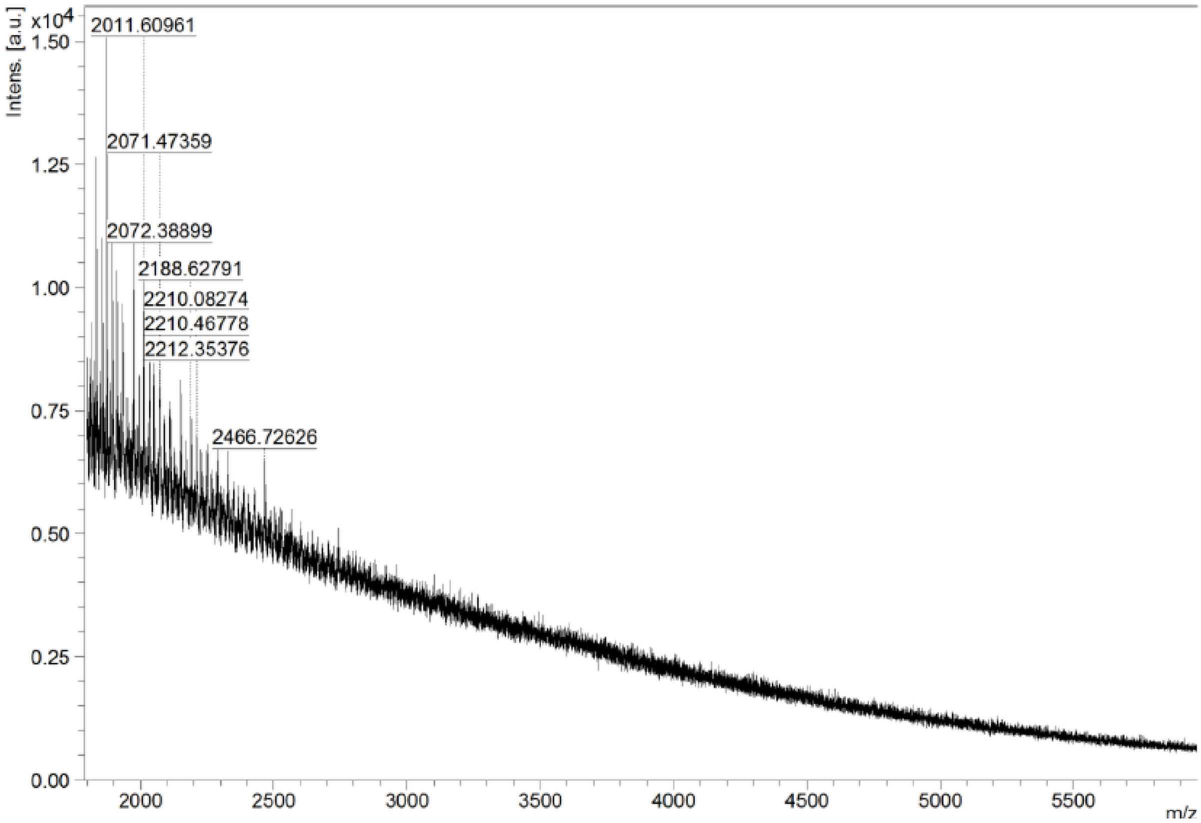

Native DNA (calculated m/z 5083, AM, with NaCl)

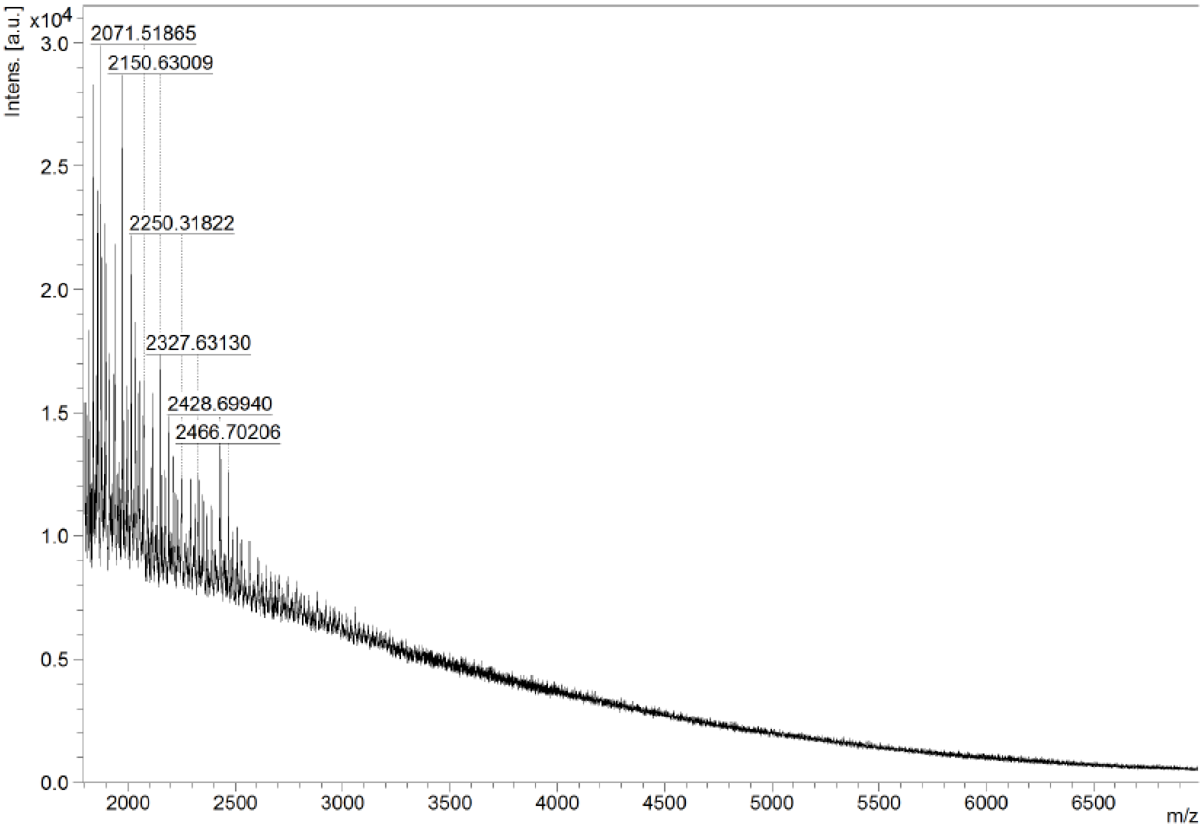

MALDI-MS Data: Assaying binding of **Ru-1** to DNAs/RNAs

hexa-T (calculated m/z 1984, no NaCl, full spectrum):

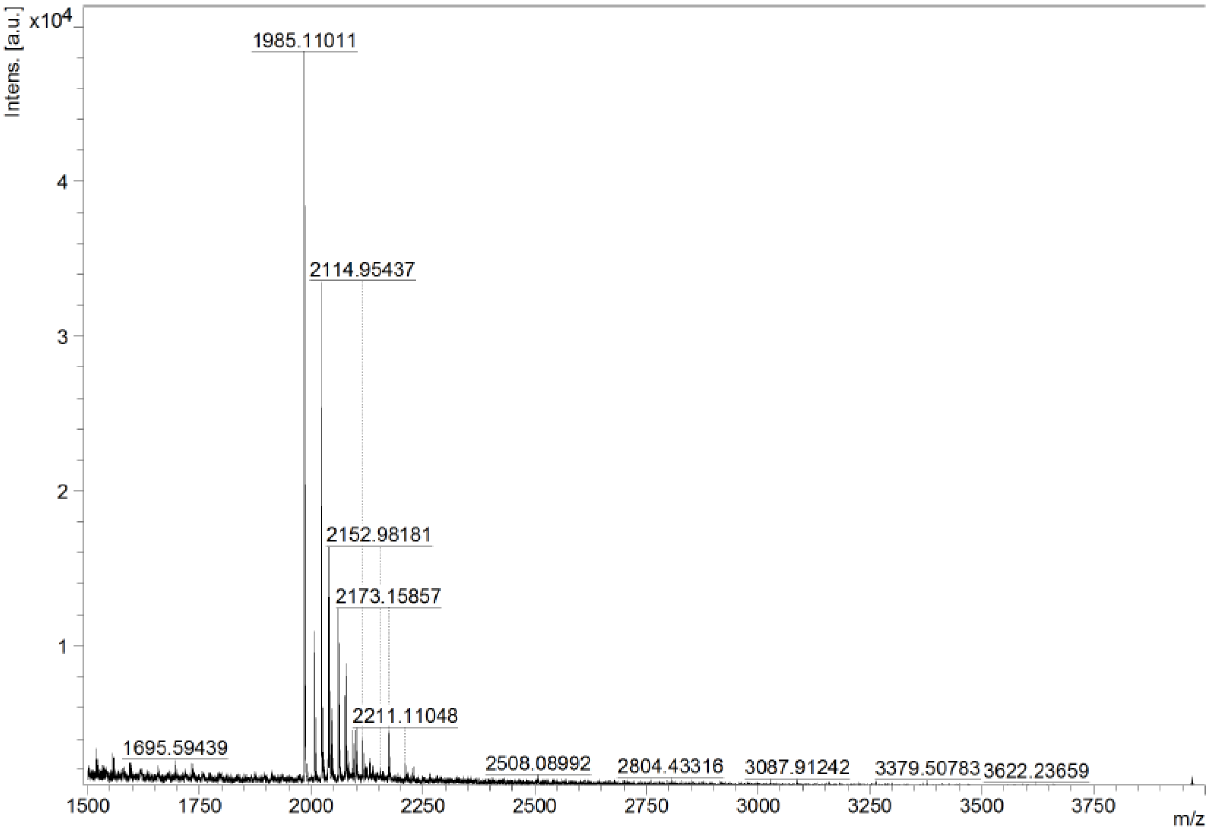

hexa-T (calculatd m/z 1984, no NaCl, zoomed-in spectrum):

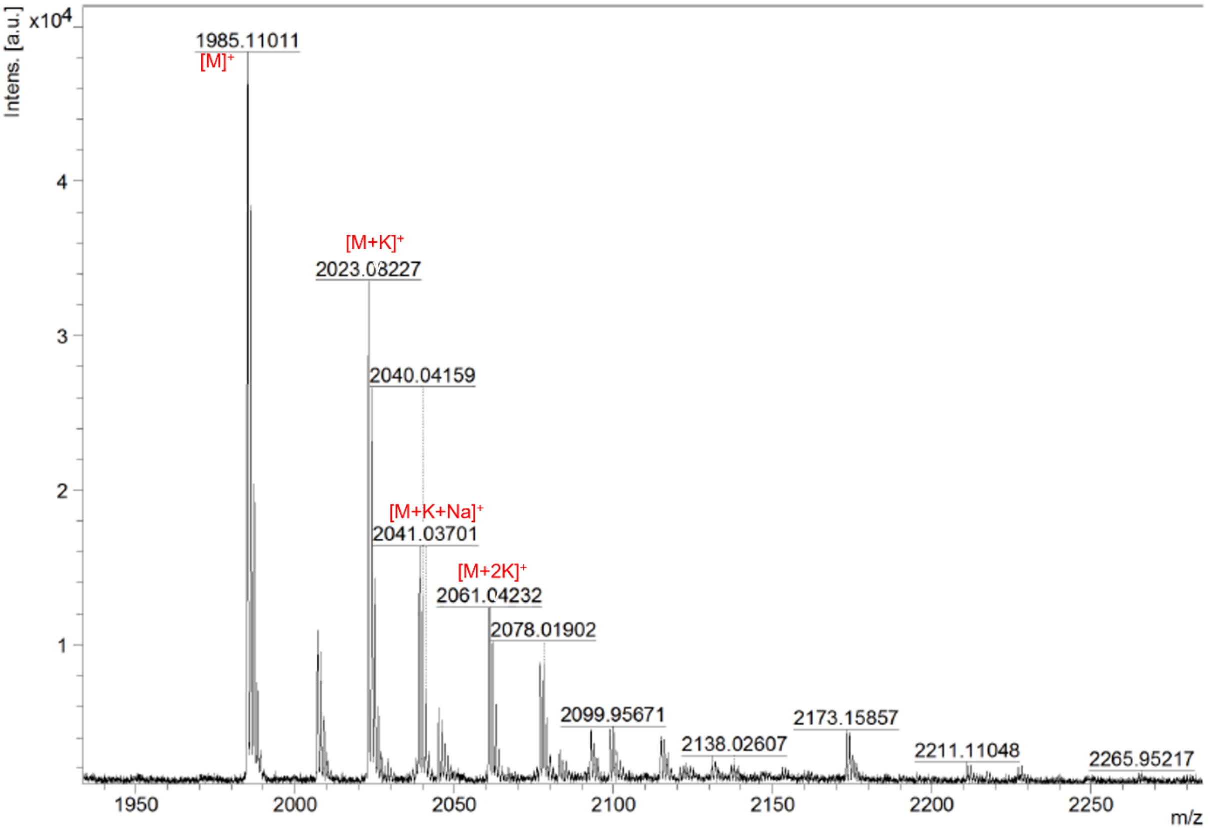

hexa-T (calculated m/z 1984, with NaCl, full spectrum):

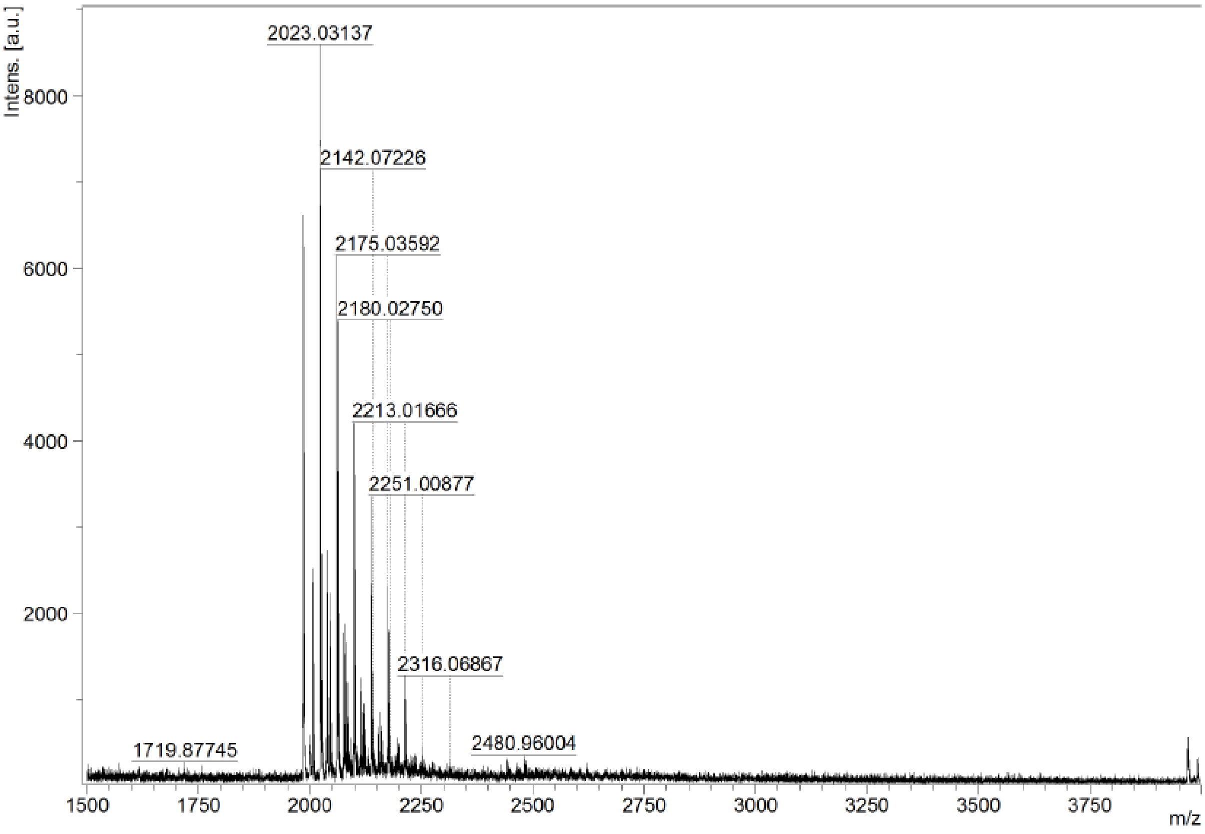

hexa-T (calculated m/z 1984, with NaCl, zoomed-in spectrum):

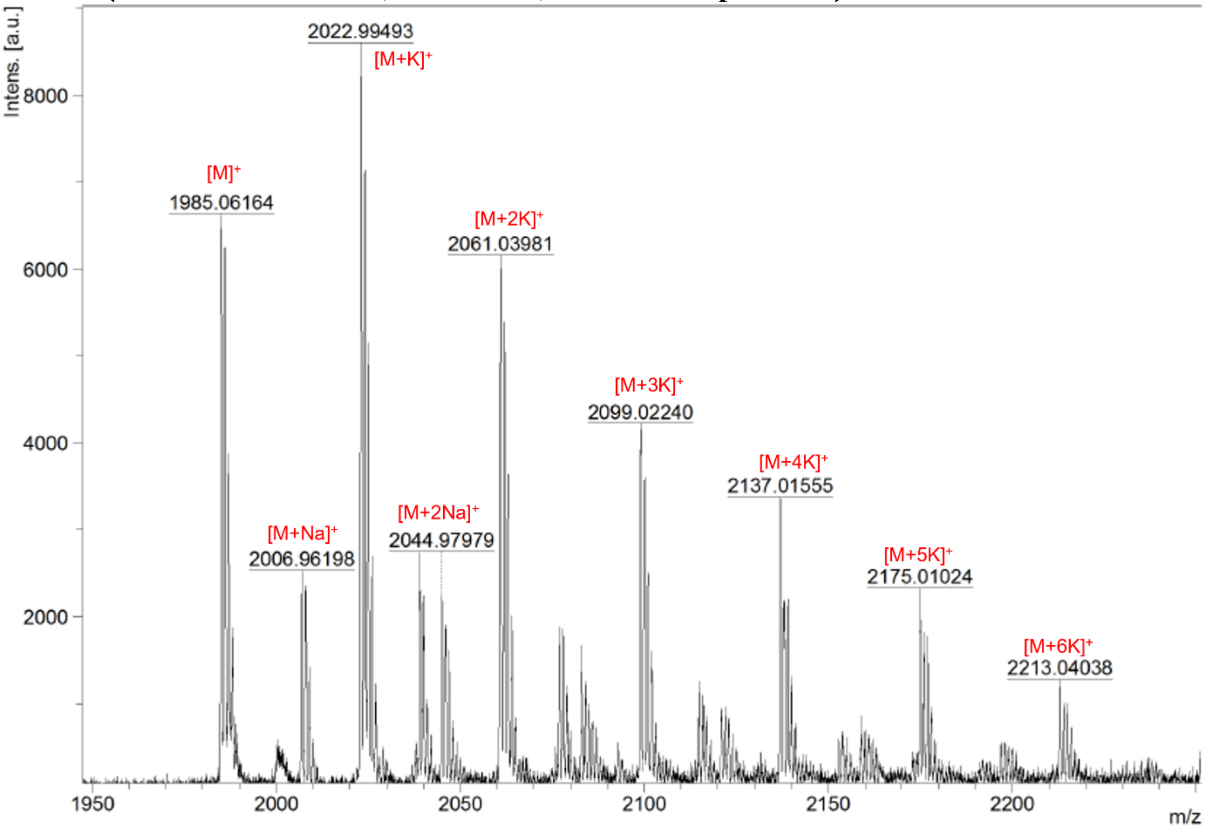

csDNA (calculated m/z 5001, no NaCl, full spectrum):

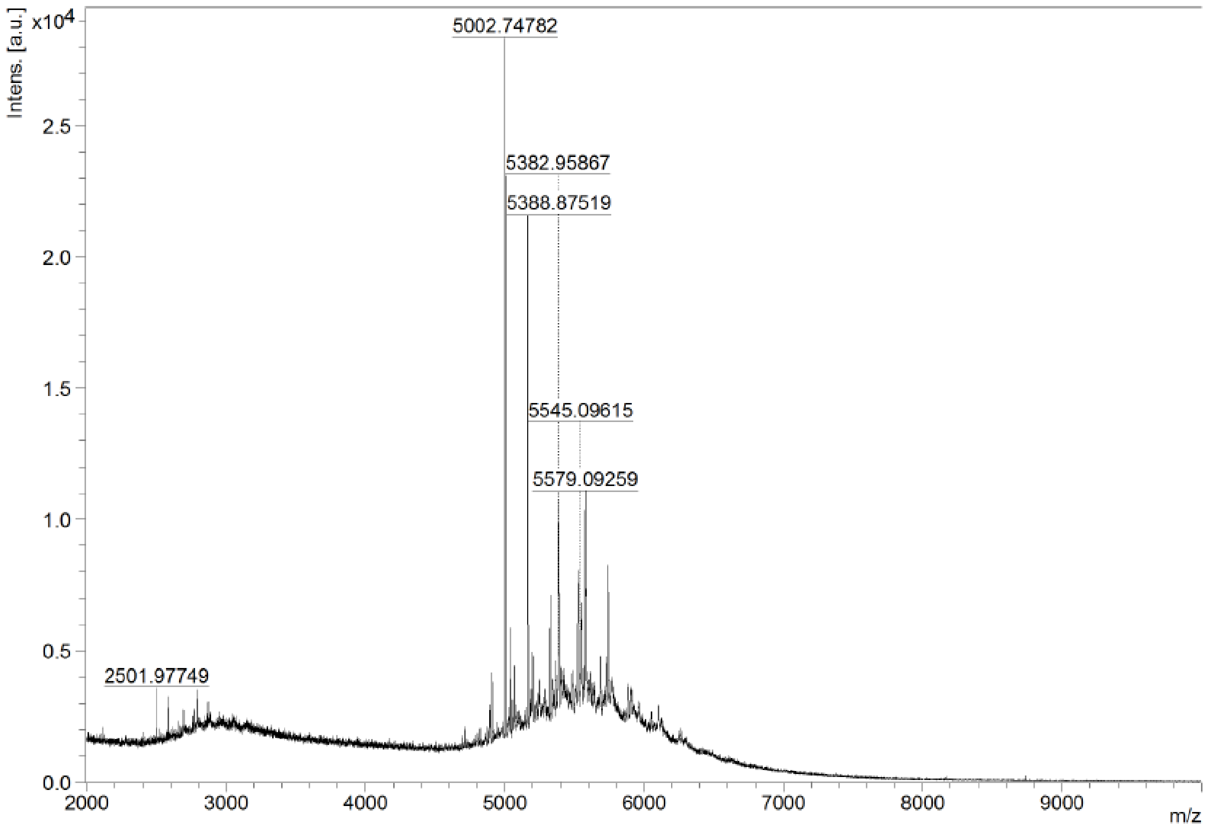

csDNA (calculated m/z 5001, no NaCl, zoomed-in spectrum):

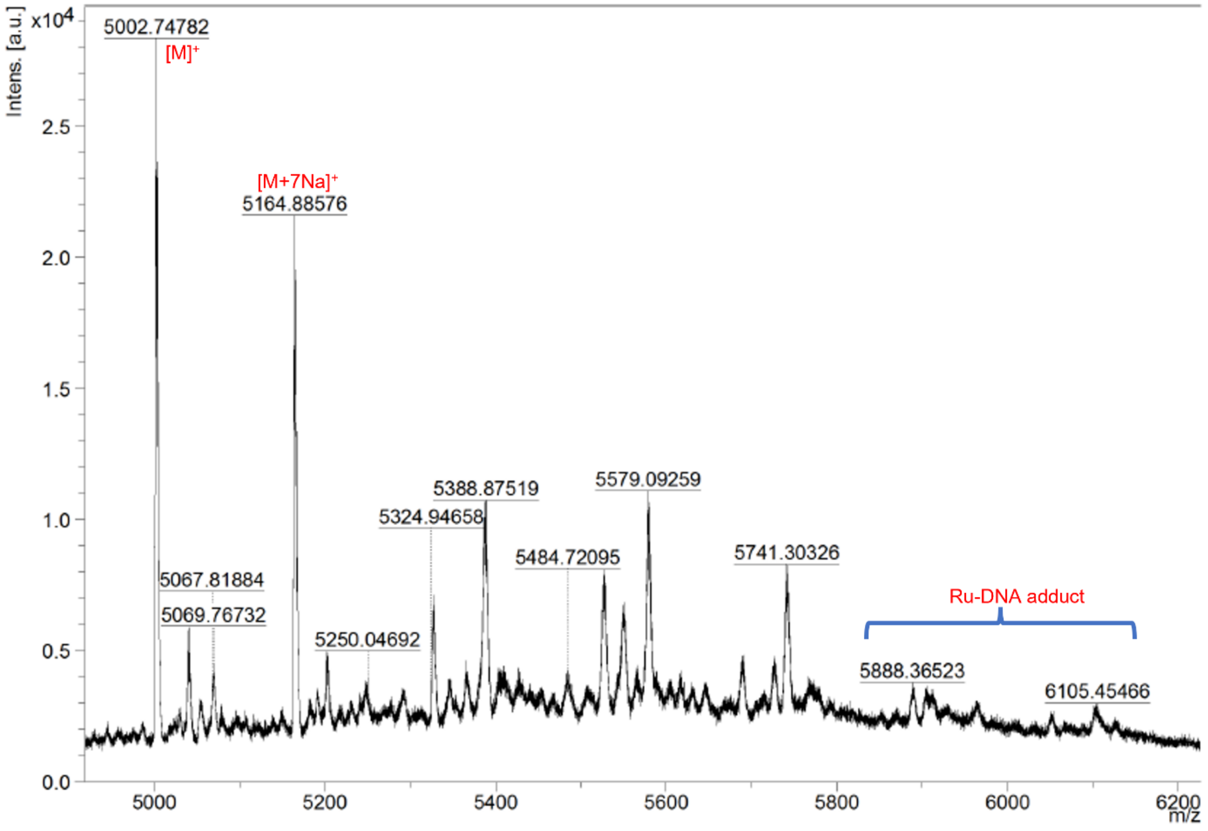

csDNA (calculated m/z 5001, with NaCl, full spectrum):

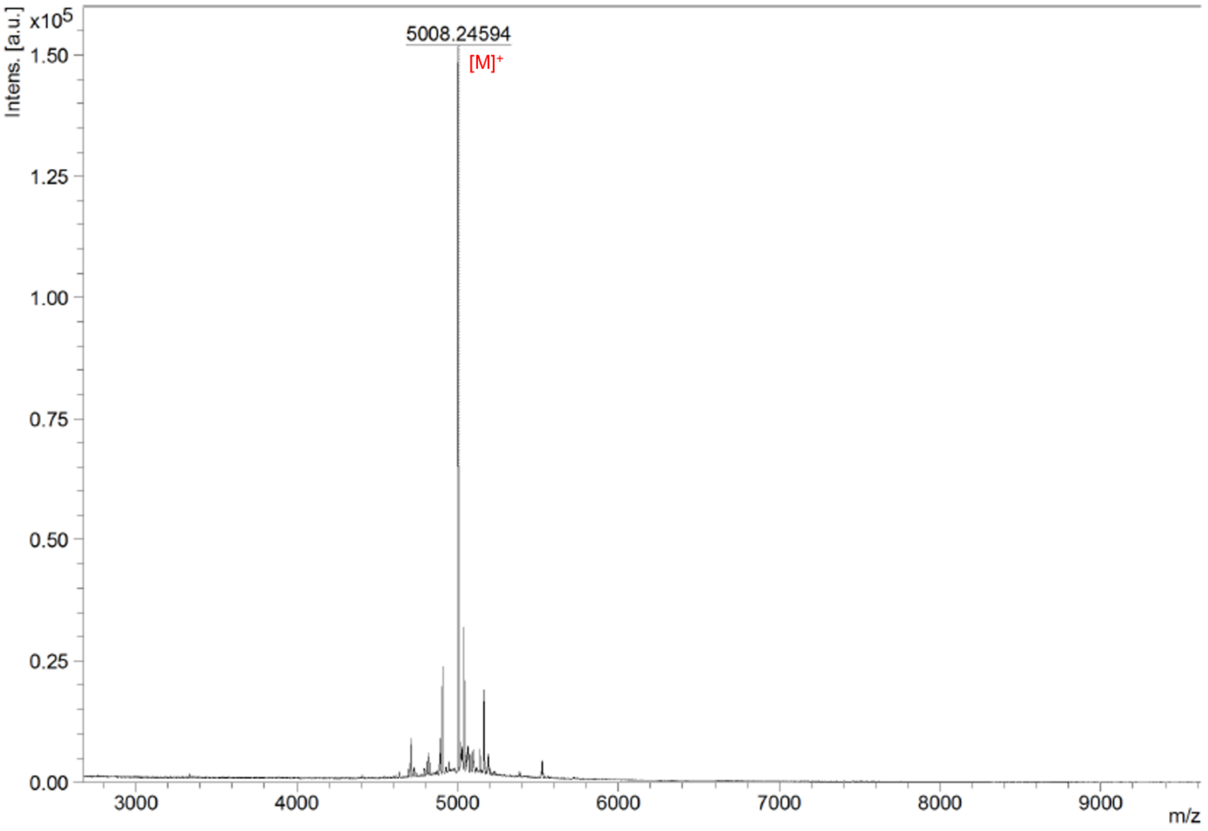

Native DNA (calculated m/z 5083, no NaCl, full spectrum):

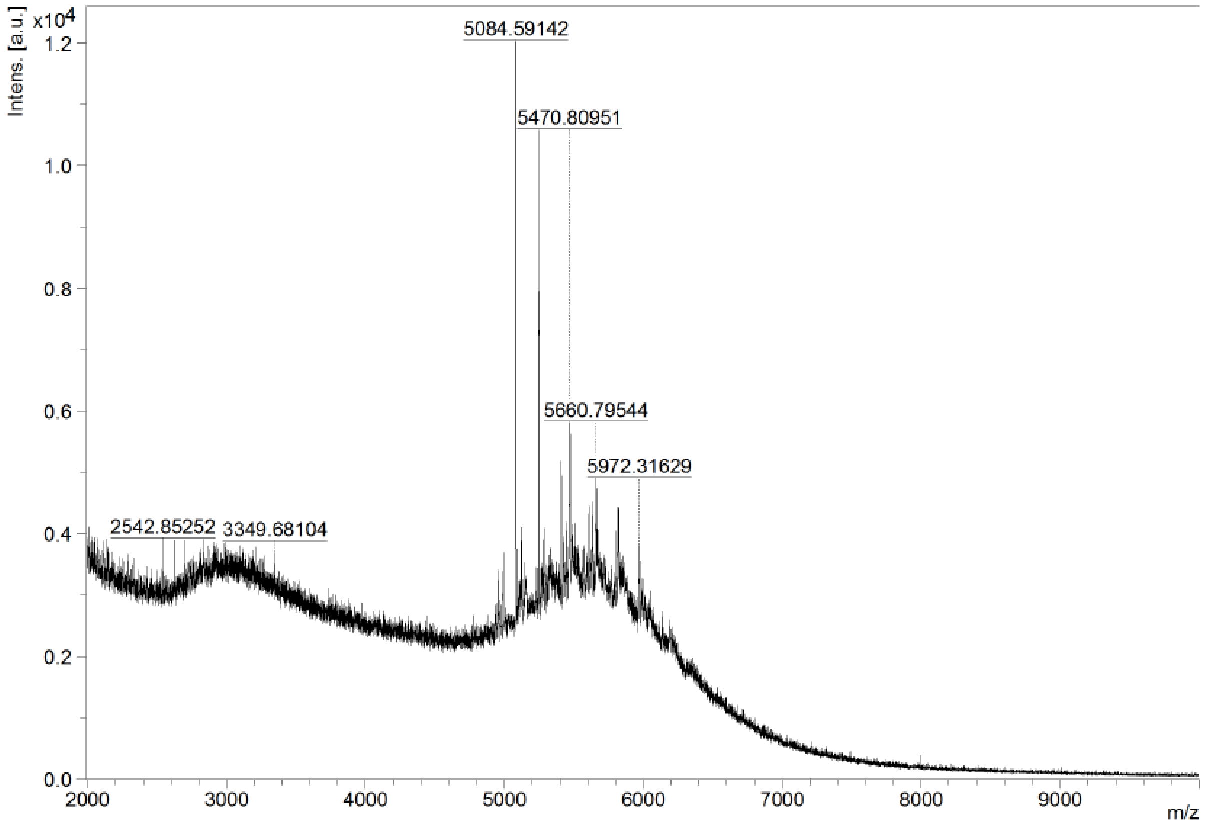

Native DNA (calculated m/z 5083, no NaCl, zoomed-in spectrum):

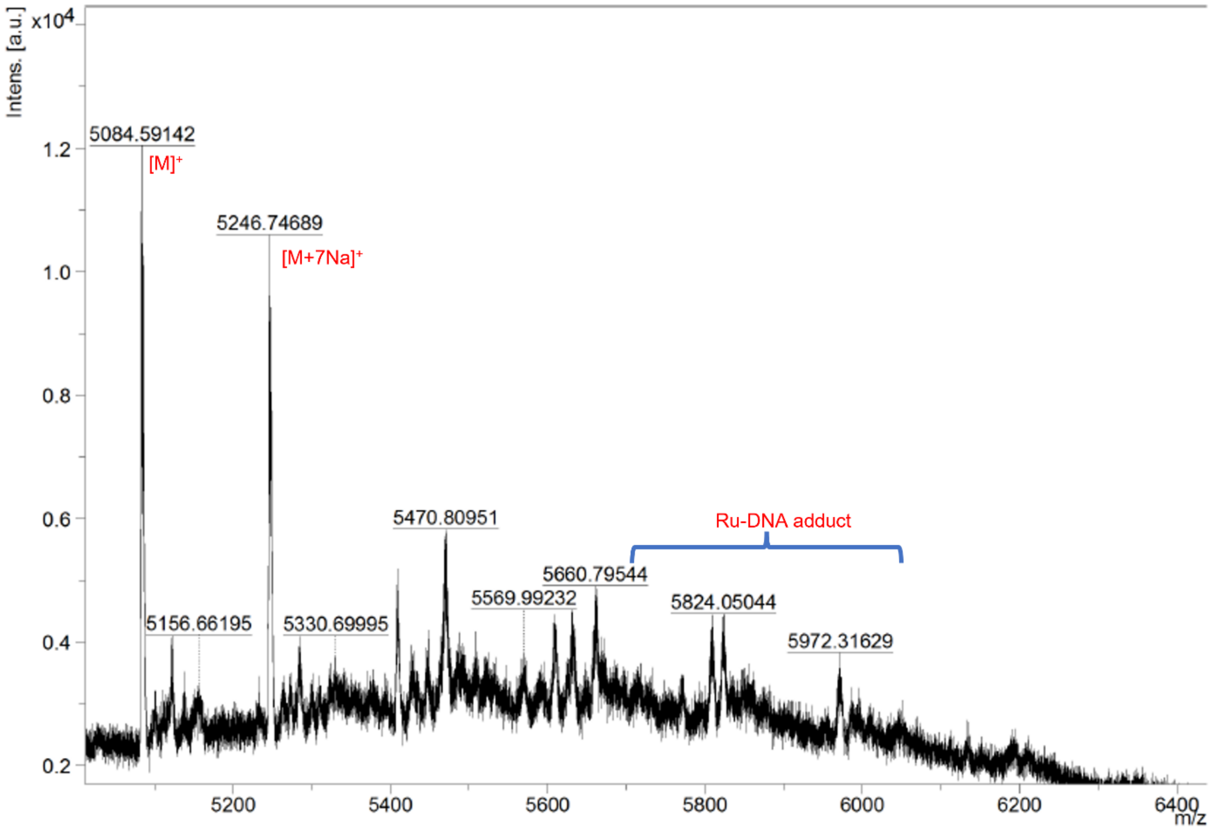

Native DNA (calculated m/z 5083, with NaCl, full spectrum):

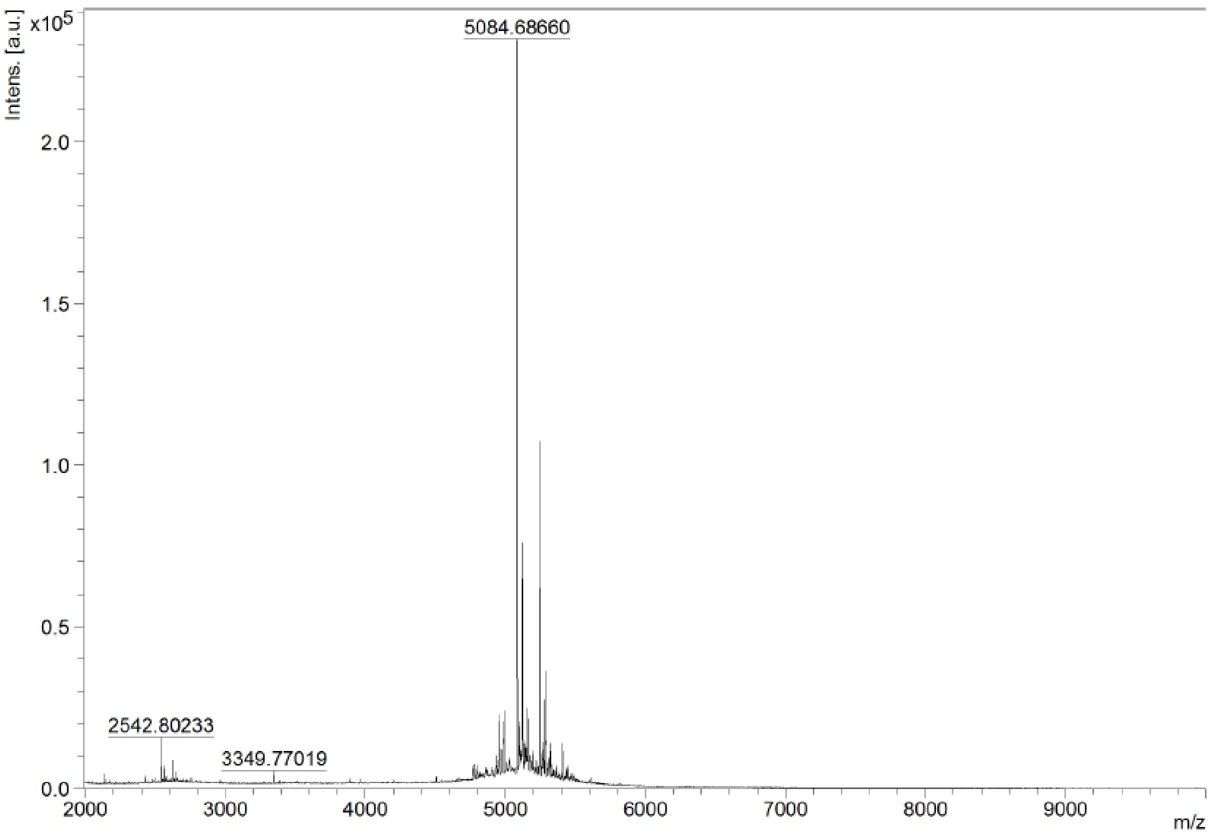

Native DNA (calculated m/z 5083, with NaCl, zoomed-in spectrum):

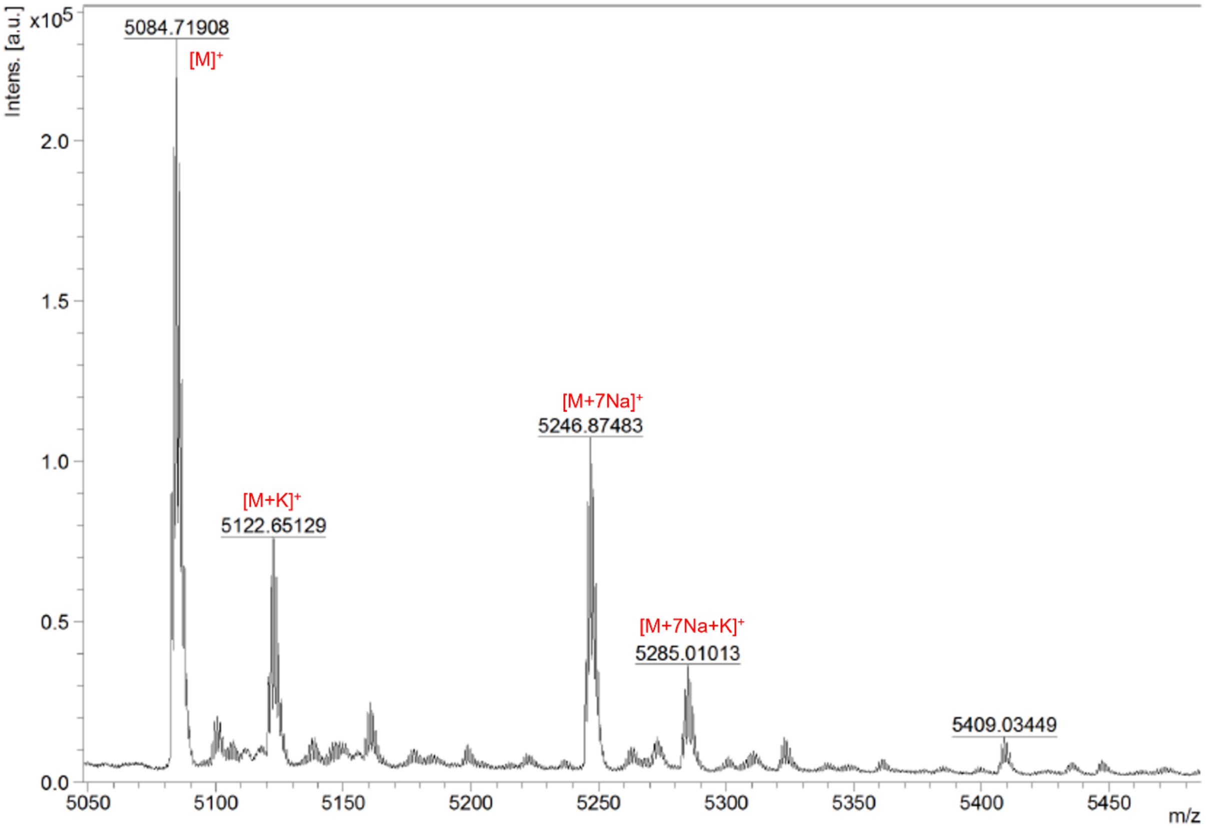

Native RNA (calculated m/z 5176, no NaCl, full spectrum):

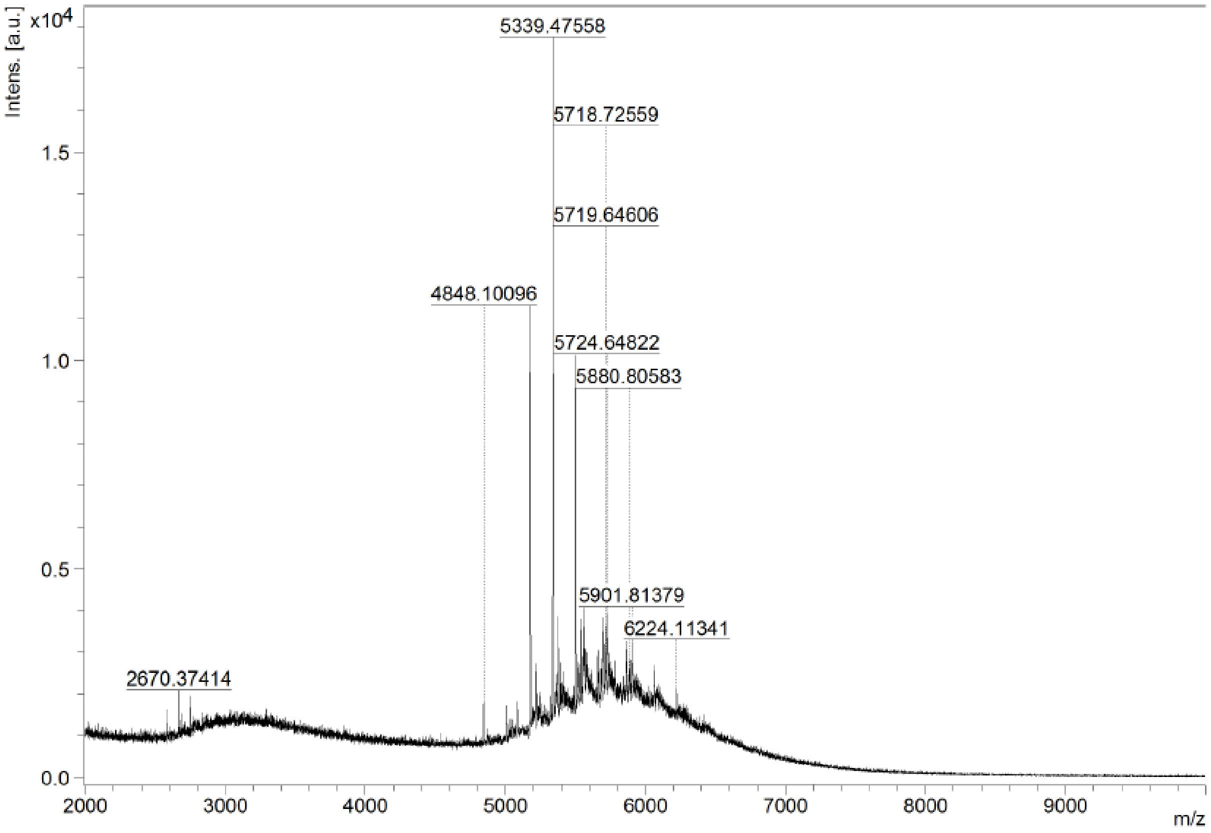

Native RNA (calculated m/z 5176, no NaCl, zoomed-in spectrum):

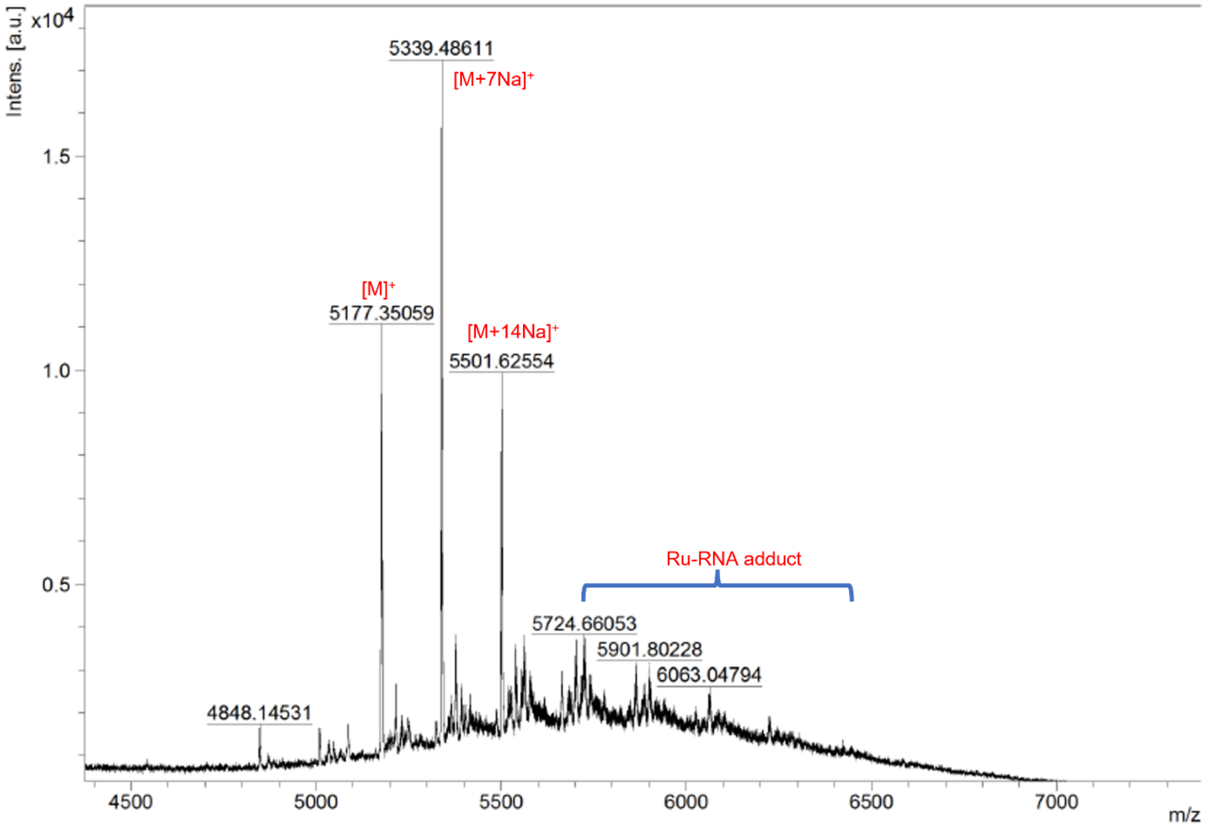

Native RNA (calculated m/z 5176, with NaCl, full spectrum):

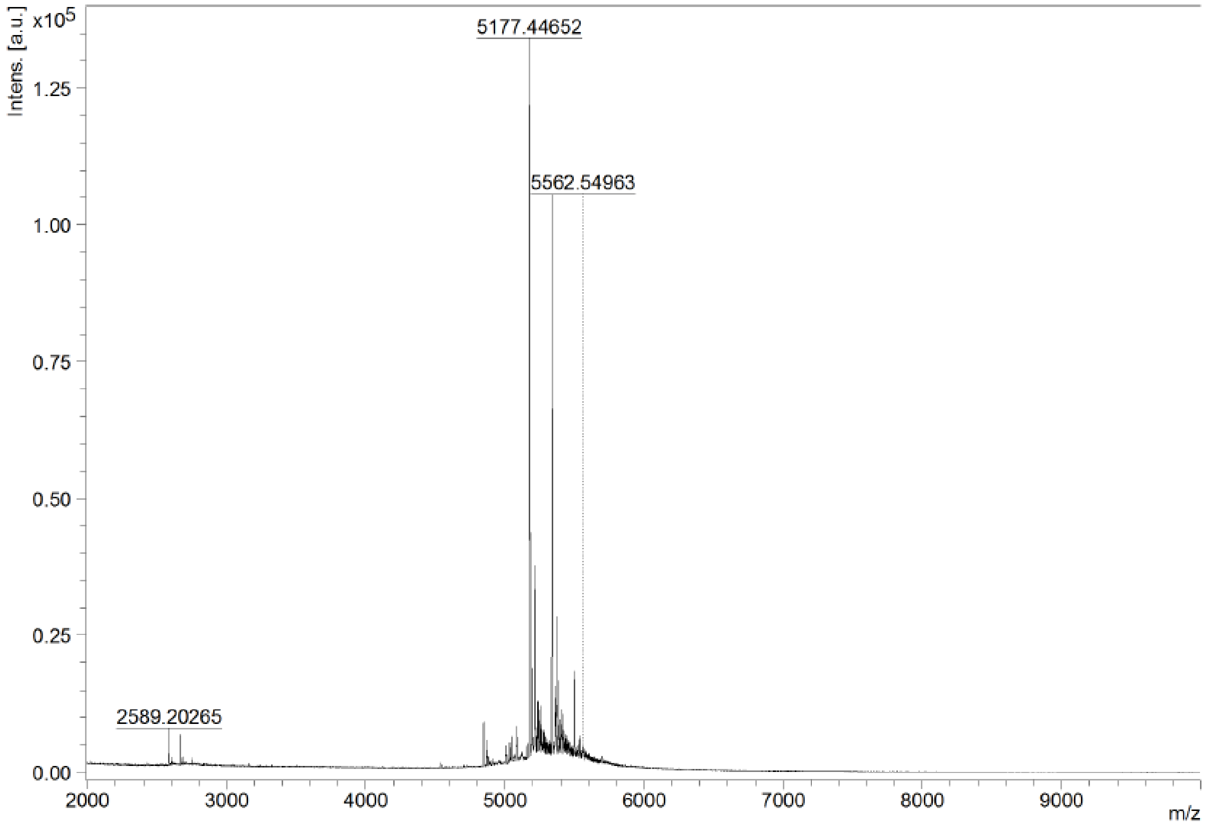

Native RNA (calculated m/z 5176, with NaCl, zoomed-in spectrum):

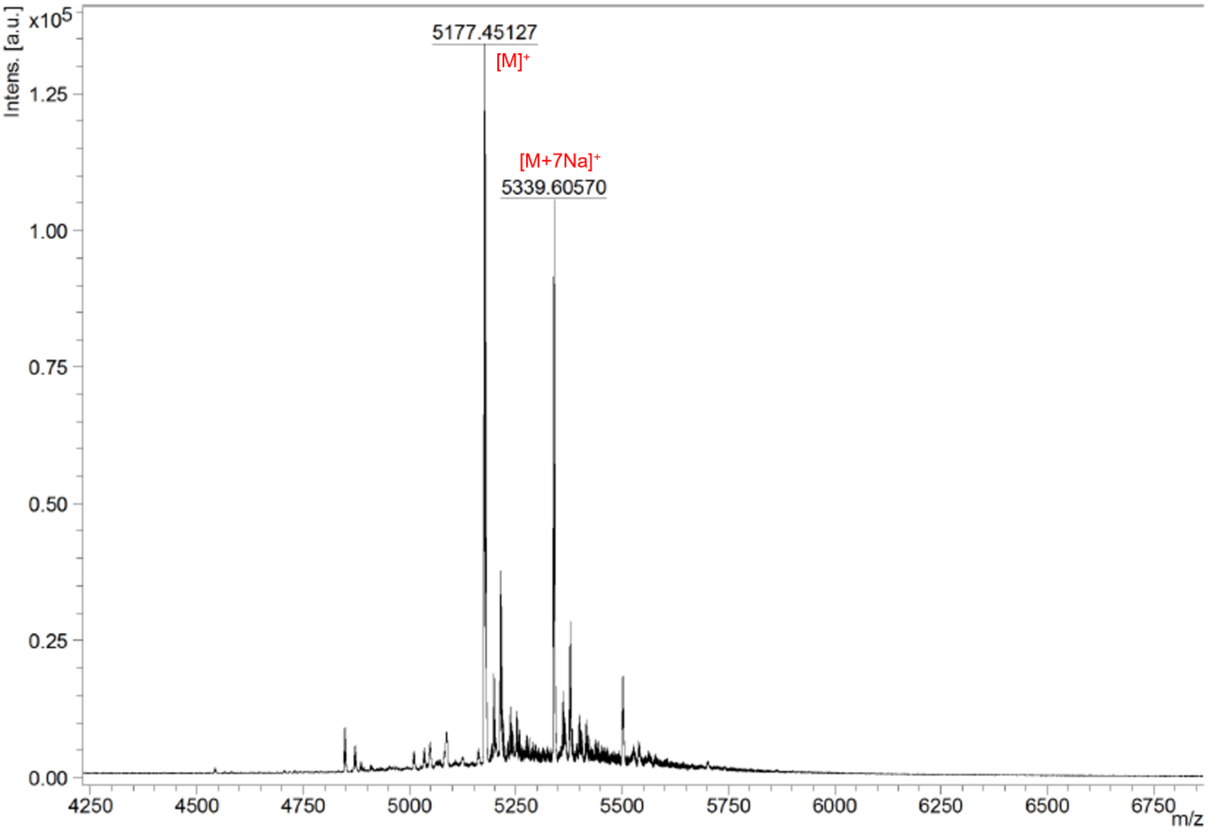

2'-OMe RNA (calculated m/z 5,400, no NaCl, full spectrum):

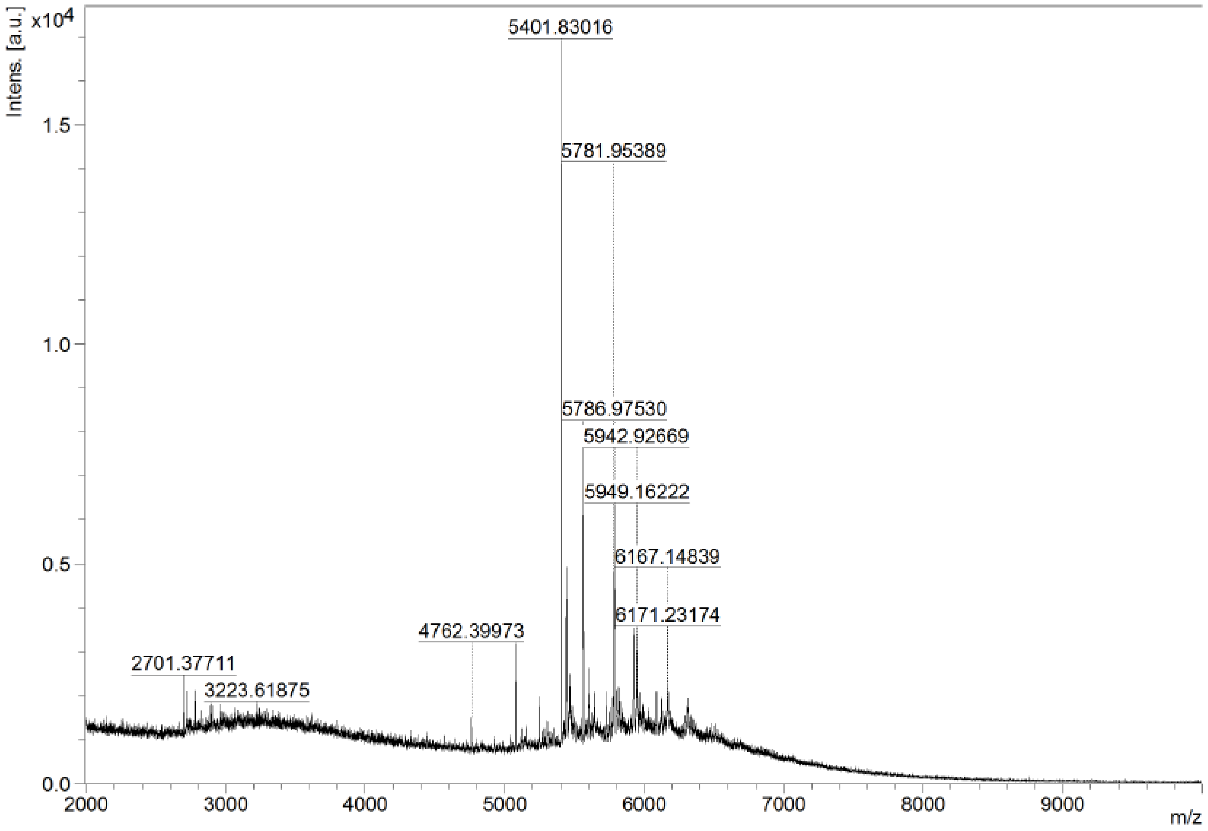

2'-OMe RNA (calculated m/z 5,400, no NaCl, zoomed-in spectrum):

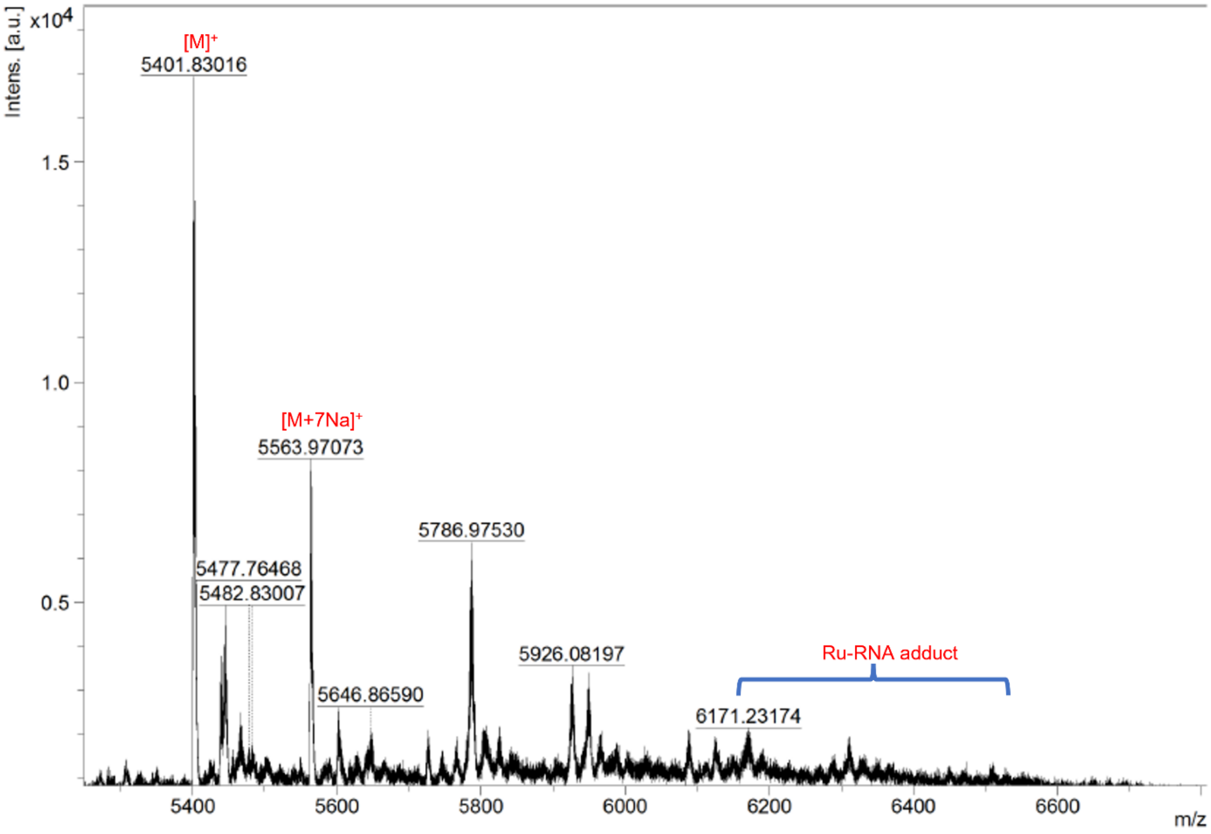

2´-OMe RNA (calculated m/z 5,400, with NaCl, full spectrum):

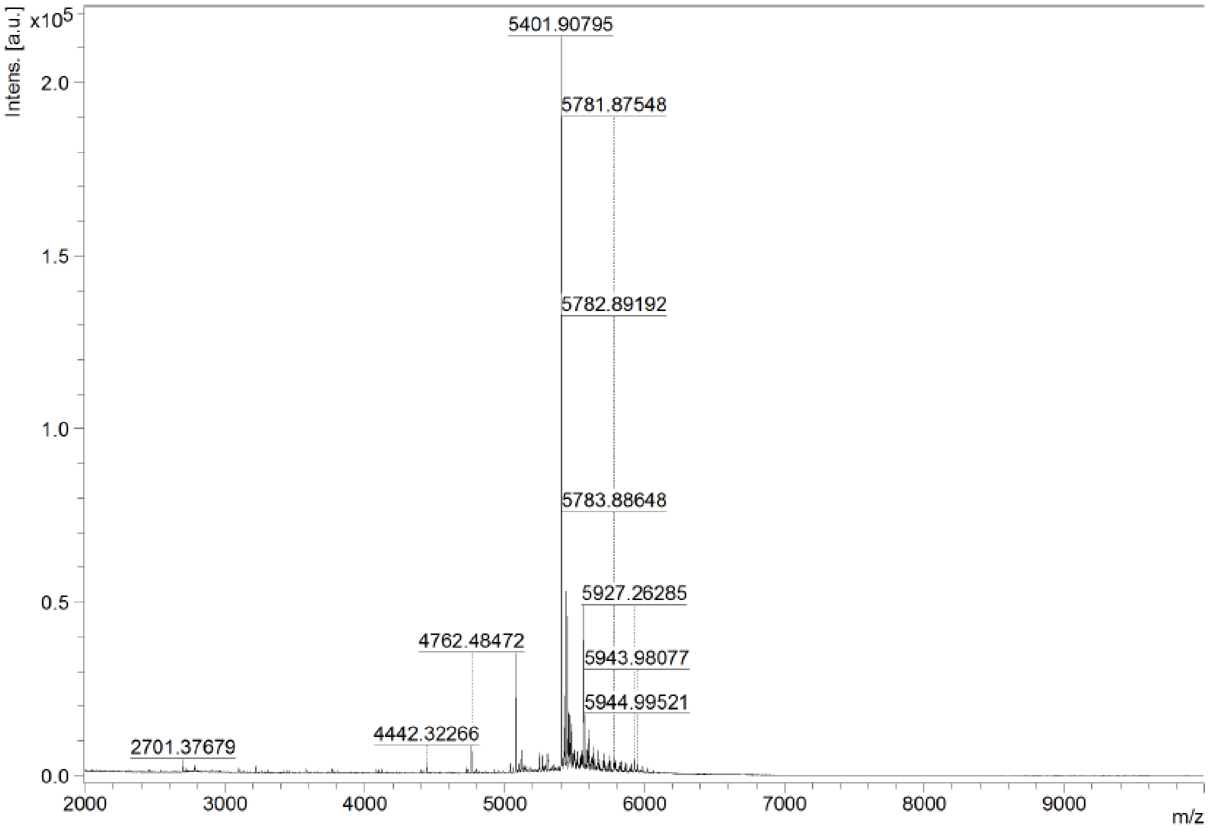

2´-OMe RNA (calculated m/z 5,400, with NaCl, zoomed-in spectrum):

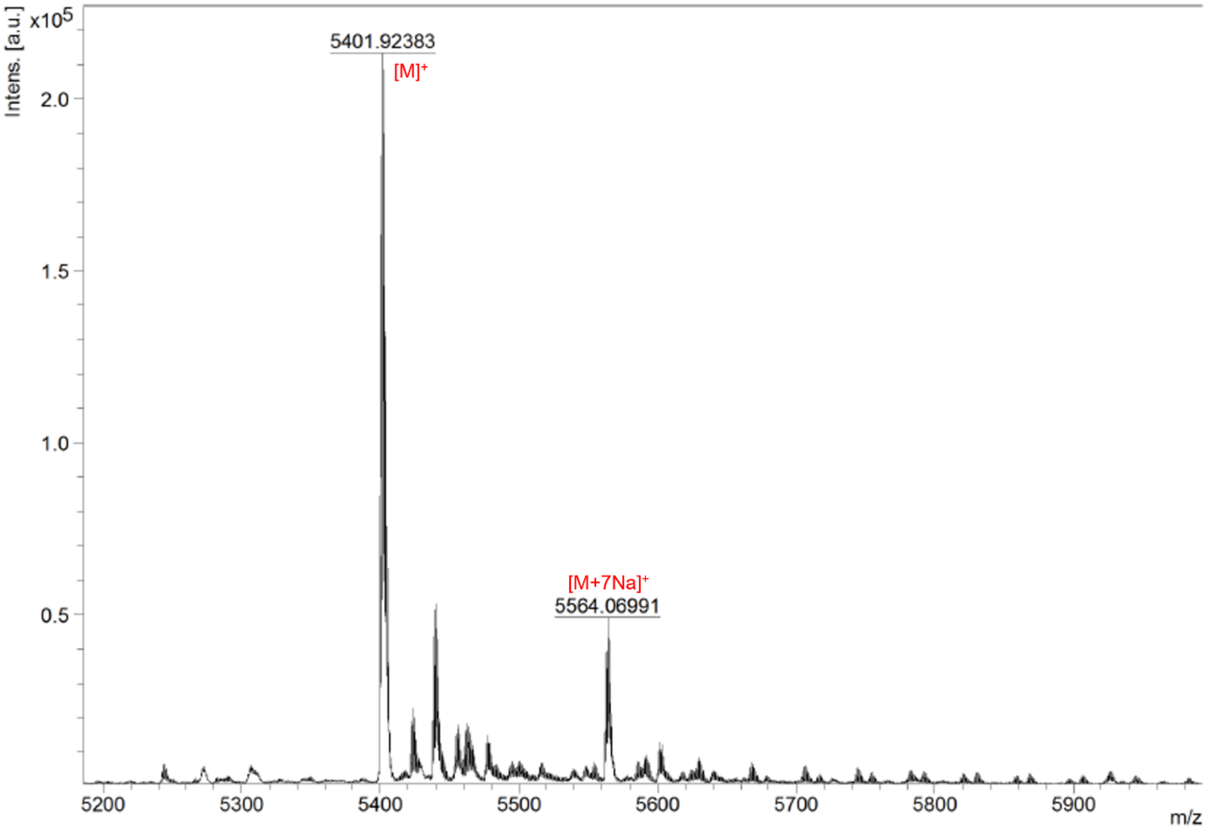

Figure S9. MALDI-MS spectra for assessing oligonucleotide adduct formation by **Ru-1**-and **AM** binding assay.

9. Assaying degradation of DNA/RNA backbone during aqueous metathesis using DNA/RNA-FAM 16mer model (Ru-1 vs. AM).

500 pmol of DNA/RNA-FAM (16mer T-FAM, native DNA-FAM, same sequence as **ATGC-1** and native RNA-FAM, same sequence as **purine25-AUGC-1**) was used in all degradation assays. 4 M NaCl, 0.8 mg/mL **AM** and 0.6 mg/mL **Ru-1** stock solutions were prepared in pH 5.0 H_2O. Prior to the reactions, all stock solutions were filtered via sterile Millex™-GV Filter Unit (pore size 0.22 µm, diam. 33 mm, PVDF membrane). All reactions were performed in a total volume of 50 µL in 1.5 mL Eppendorf tubes. 35 µL H_2O (pH 5.0), 500 pmol of DNA/RNA, dissolved in 5 µL of water, 5 µL 4 M NaCl stock solution or 5 µL pH 5.0 H_2O and 5 µL Ruthenium catalyst solutions were added to 1.5 mL Eppendorf tubes. These were briefly flushed with argon, sealed with Parafilm and shaken on an Eppendorf thermocycler at RT and 70 °C for 30 min. The reactions were quenched by adding 10 equiv (vs Ru) SnatchCat (1,4-bis(3-isocyanopropyl) piperazine) and shaking at 37 °C for 30 min. The reactions were analyzed by gel electrophoresis, MALDI-MS, and analytical reverse-phase (RP)-HPLC. **Method**. Linear gradient of 5% to 50% MeOH within 15 min, then 50% to 100% MeOH within 1 min, followed by 100% MeOH for 2 min, then 100% to 5% MeOH within 1 min, followed by 5% MeOH for 2 min. HPLC chromatograms were recorded at 260 nm, 280 nm and 495 nm wavelengths. Before MALDI-MS measurements, the samples were desalted using ZipTip pipette tips according to the manufacturer´s protocol. Gel electrophoresis was performed according to General Procedure 5; band quantification was assessed using ImageJ software.

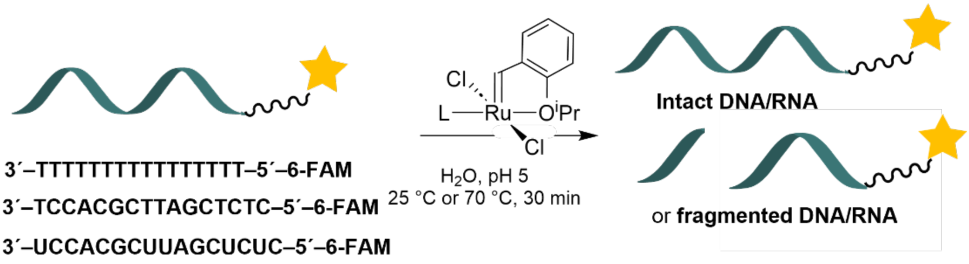

16mer T-FAM, 25 °C

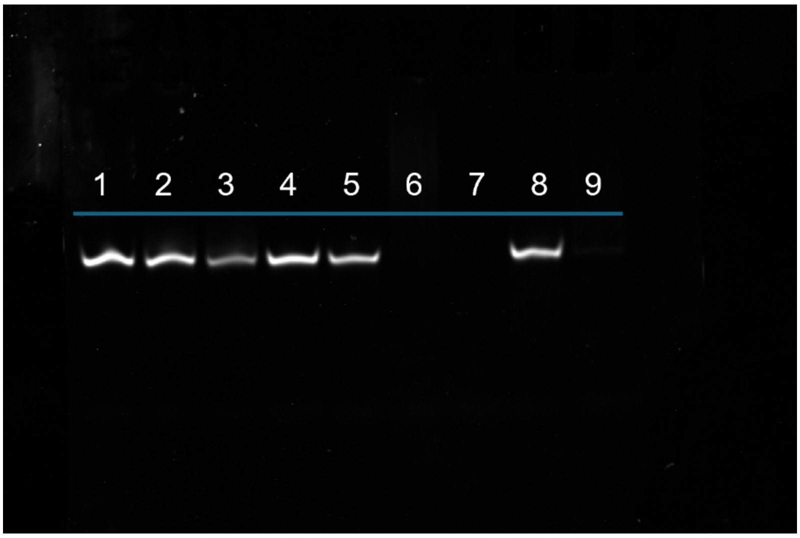

Figure S10. Backbone degradation of fluorescein-labelled oligothymidine 16mer by **Ru-1** and **AM** at 25 °C. Lane 1: Ru-free control, normalized to 100% for band intensity quantification (BQ). Lanes 2-3: 1 equiv **Ru-1** or **AM**; BQ 82% or 41%. Lanes 4-5: 1 equiv **Ru-1** or **AM** in

0.4 M NaCl; BQ 95% or 67%. Lanes 6-7: 10 equiv **Ru-1** or **AM**, BQ 0%. Lanes 8-9: 10 equiv **Ru-1** or **AM** in 0.4 M NaCl; BQ 83% or 0%.

Table S4: 16mer T-FAM backbone degradation assay comparing **Ru-1** and **AM** at 25 °C. DNA was analyzed by gel electrophoresis and HPLC. DNA degradation upon treatment with either **Ru-1** or **AM** was calculated based on product peak integration vs the non-treated control.

Entry	Catalyst (equiv)	Additives (equiv)	DNA quantification by electrophoresis (%)	DNA quantification by HPLC analysis (Area under the curve)
1	None	None	100	100
2	1 **Ru-1**	None	82	70
3	1 **AM**	None	41	27
4	1 **Ru-1**	40,000 NaCl	95	71
5	1 **AM**	40,000 NaCl	67	56
6	10 **Ru-1**	None	0	0
7	10 **AM**	None	0	0
8	10 **Ru-1**	40,000 NaCl	83	65
9	10 **AM**	40,000 NaCl	0	0

RP-HPLC chromatograms of DNA backbone degradation assay.

Entry 1: Control

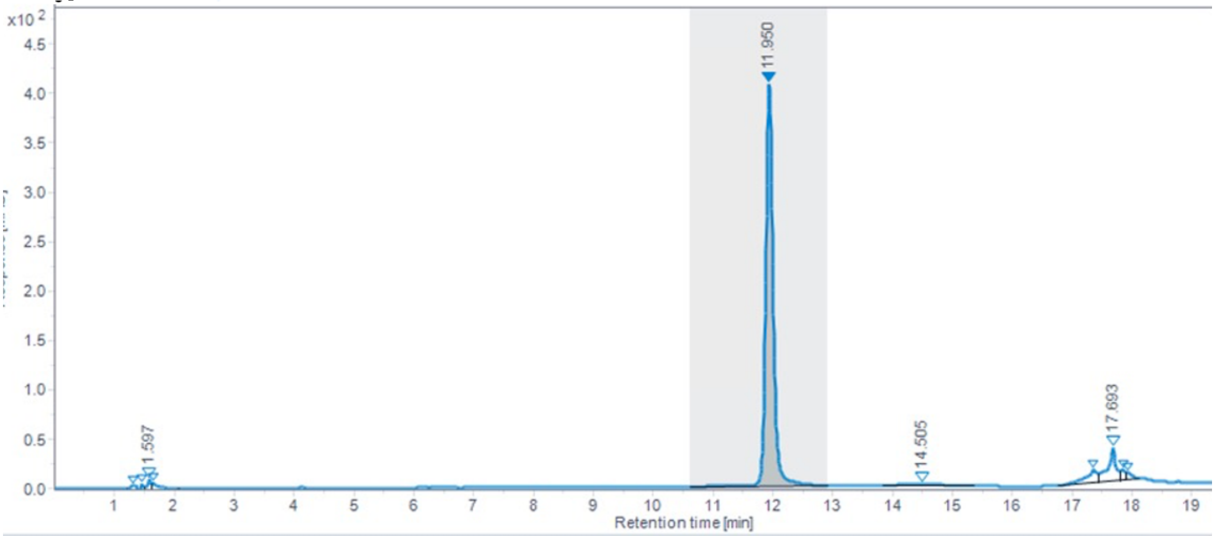

Injection Results

Peaks Summary

#	Name	Signal description	RT (min)	Area (mAU·s)	Area%	Height (mAU)	Height%
5		DAD1A,Sig=260,4 Ref=off	11.950	3573.303	70.902	406.839	74.53

MALDI-MS: 16mer oligothymidine-FAM starting material.

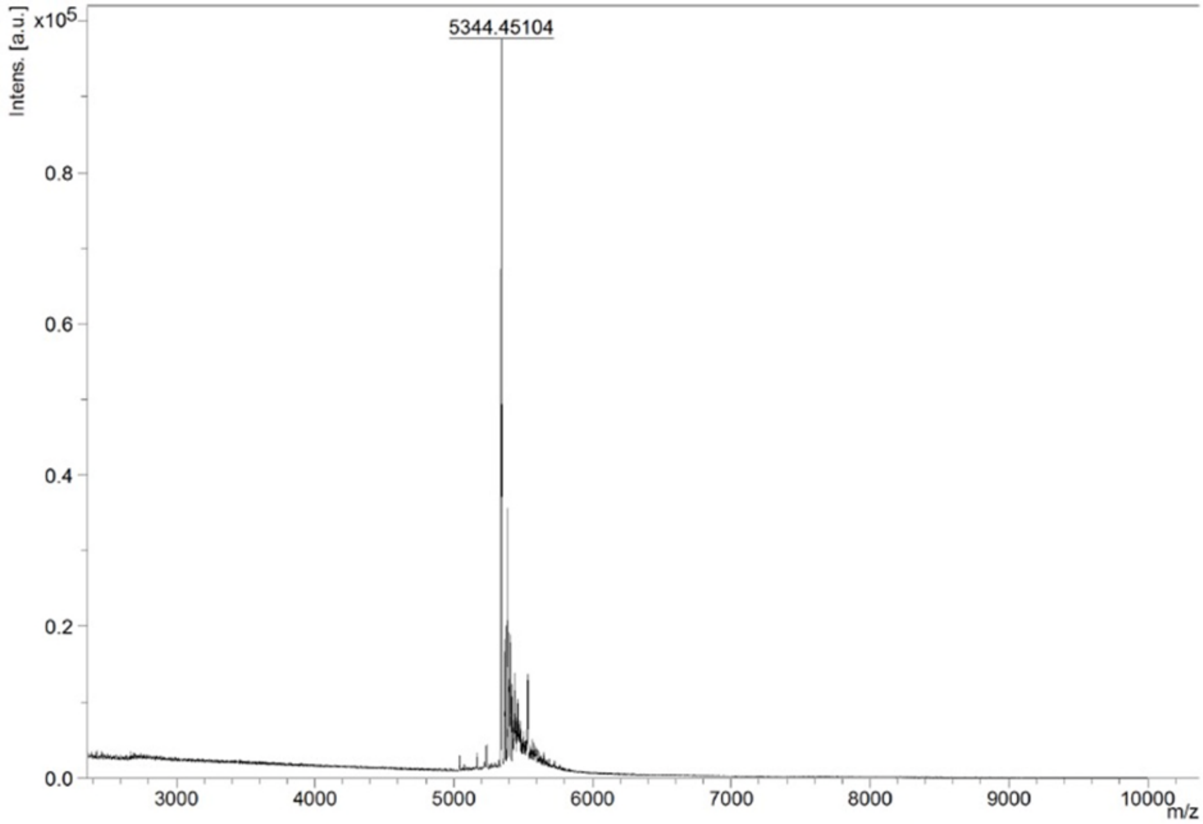

Entry 2: 1 equiv **Ru-1**, no additives

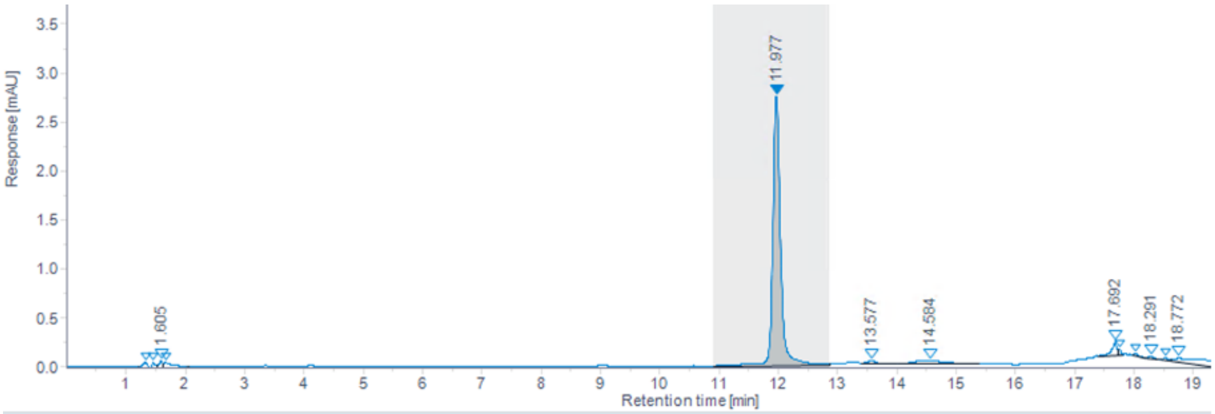

Injection Results

Peaks | Summary

#	Name	Signal description	RT (min)	Area (mAU·s)	Area%	Height (mAU)	Height%
5		DAD1A,Sig=260,4 Ref=off	11.977	2502.342	63.415	274.541	72.33

Entry 3: 1 equiv **AM**, no additives

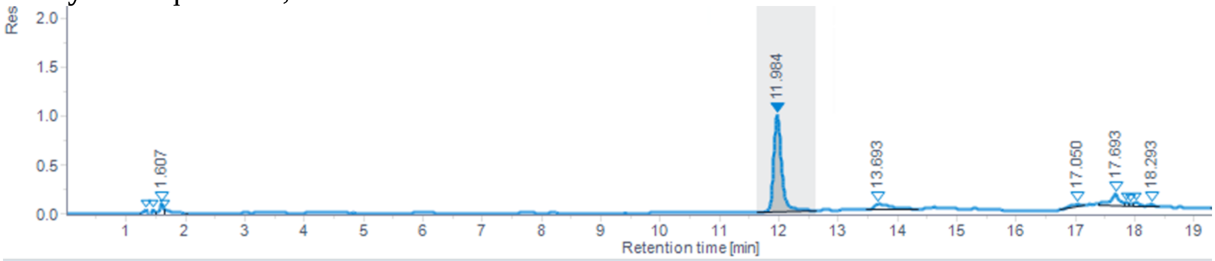

Injection Results

Peaks | Summary

#	Name	Signal description	RT (min)	Area (mAU·s)	Area%	Height (mAU)	Height%
5		DAD1A,Sig=260,4 Ref=off	11.984	949.856	45.646	99.014	50.39

Entry 4: 1 equiv **Ru-1**; 40,000 equiv NaCl

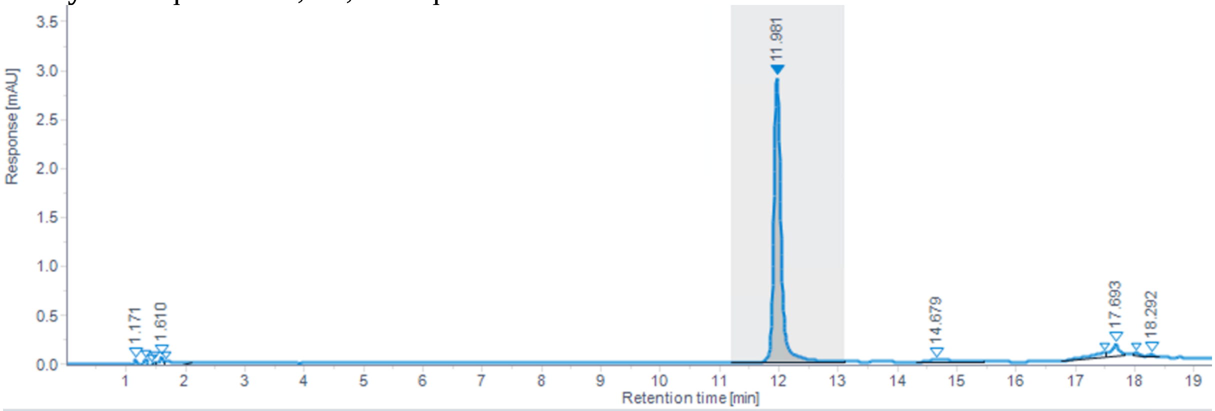

Injection Results

Peaks | Summary

#	Name	Signal description	RT (min)	Area (mAU·s)	Area%	Height (mAU)	Height%
7		DAD1A,Sig=260,4 Ref=off	11.981	2542.144	71.504	291.447	74.63

Entry 5: 1 equiv **AM**; 40,000 equiv NaCl

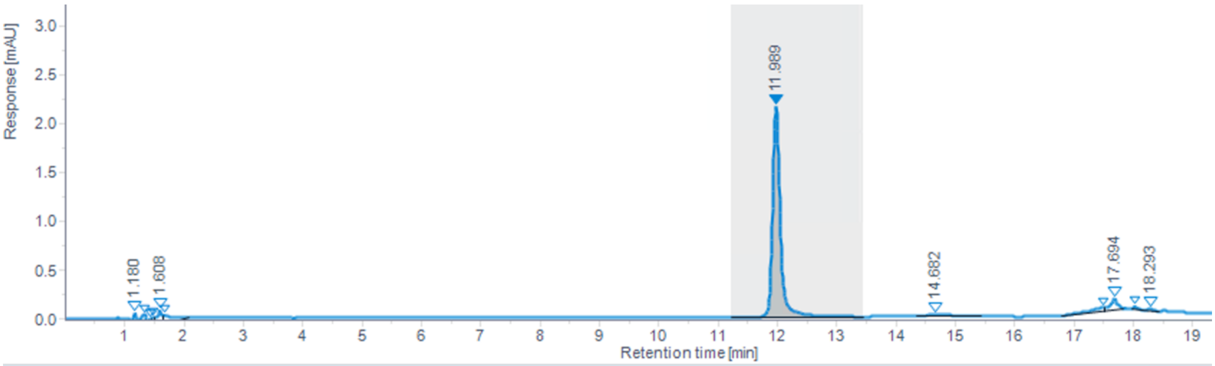

Injection Results

Peaks | Summary

#	Name	Signal description	RT (min)	Area (mAU·s)	Area%	Height (mAU)	Height%
7		DAD1A,Sig=260,4 Ref=off	11.989	2018.386	66.614	216.072	67.18

Entry 6: 10 equiv **Ru-1**, no additives

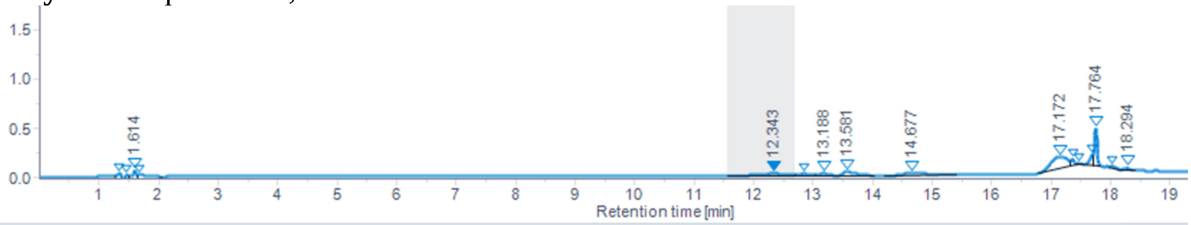

Injection Results

Peaks Summary

#	Name	Signal description	RT (min)	Area (mAU·s)	Area%	Height (mAU)	Height%
5		DAD1A,Sig=260,4 Ref=off	12.343	77.737	5.542	3.294	2.24

Entry 7: 10 equiv **AM**, no additives

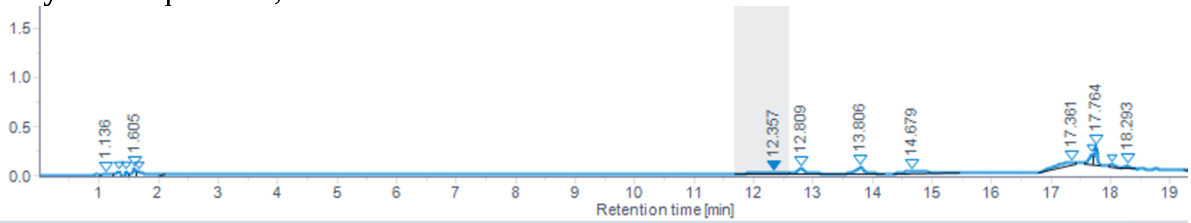

Injection Results

Peaks Summary

#	Name	Signal description	RT (min)	Area (mAU·s)	Area%	Height (mAU)	Height%
6		DAD1A,Sig=260,4 Ref=off	12.357	48.897	3.431	1.708	1.33

Entry 8: 10 equiv **Ru-1**; 40,000 equiv NaCl

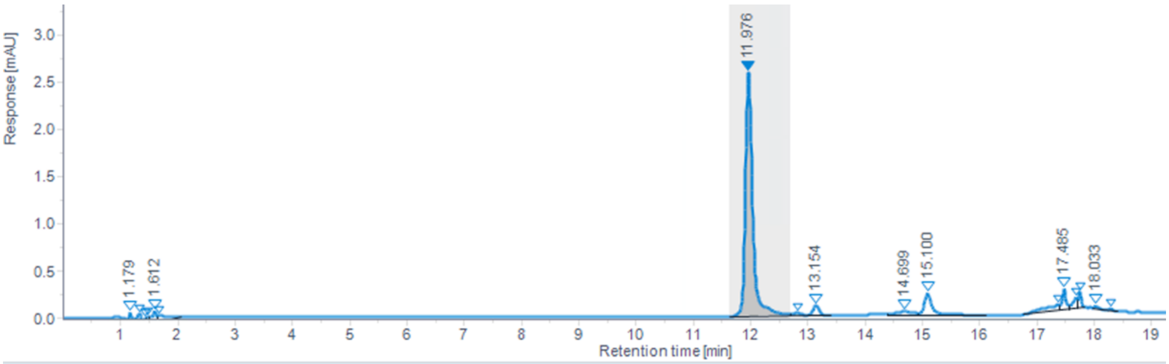

Injection Results

Peaks Summary

#	Name	Signal description	RT (min)	Area (mAU·s)	Area%	Height (mAU)	Height%
7		DAD1A,Sig=260,4 Ref=off	11.976	2313.237	60.897	257.951	59.40

Entry 9: 10 equiv **AM**; 40,000 equiv NaCl

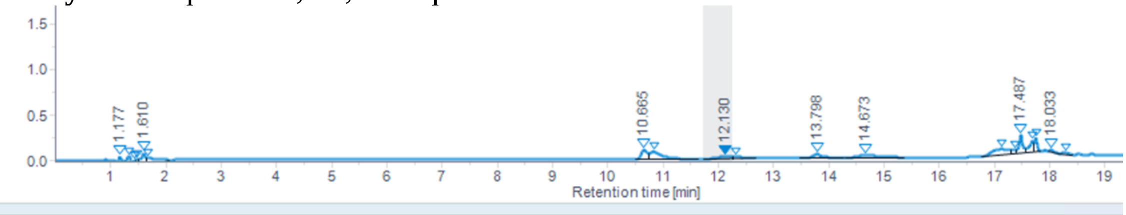

Injection Results

Peaks | Summary

#	Name	Signal description	RT (min)	Area (mAU·s)	Area%	Height (mAU)	Height%
7		DAD1A,Sig=260,4 Ref=off	10.665	80.685	5.646	9.842	6.13
8		DAD1A,Sig=260,4 Ref=off	10.841	126.449	8.848	8.320	5.18
9		DAD1A,Sig=260,4 Ref=off	12.130	45.589	3.190	3.001	1.87

Maldi-MS analysis: Entry 9: 10 equiv **AM**; 40,000 equiv NaCl

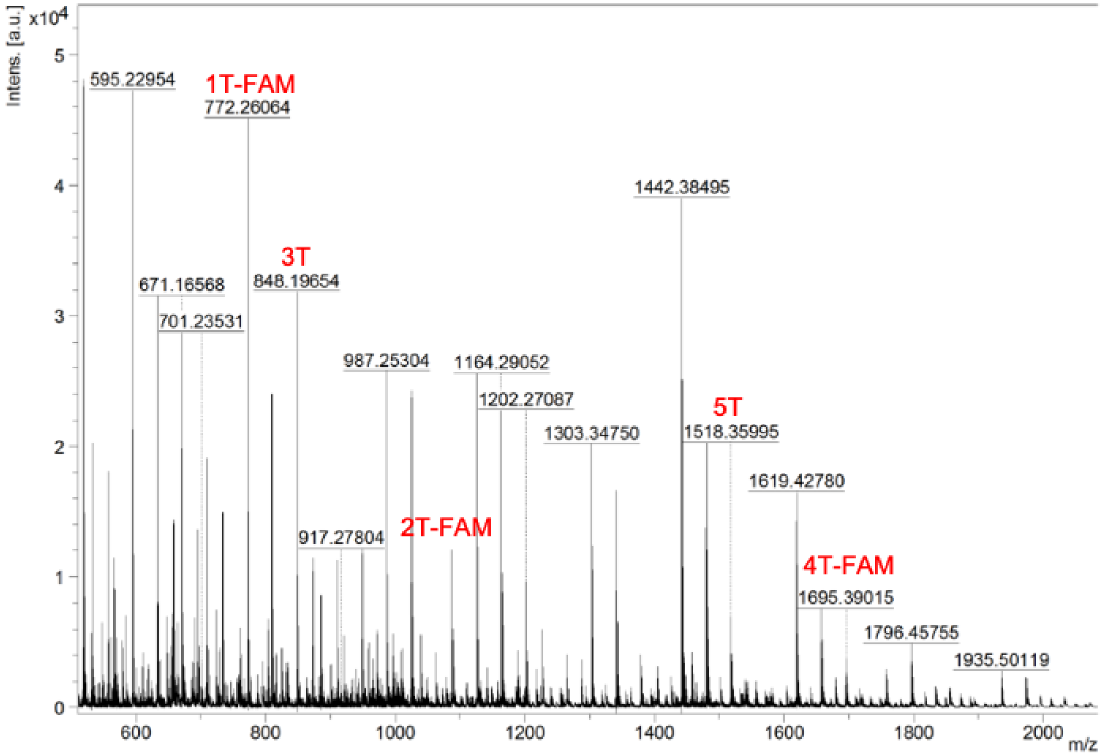

Figure S11. RP-HPLC traces for **Table S4** (entries 1-9) and MALDI-MS spectra showing degradation of DNA backbone of 16mer oligothymidine-FAM model during aqueous metathesis using **Ru-1** or **AM** at 25 °C.

16mer T-FAM, 70°C

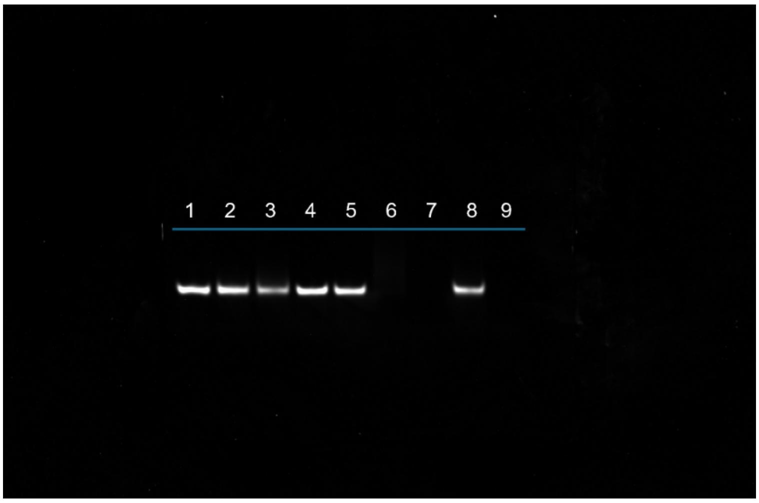

Figure S12. Backbone degradation of fluorescein-labelled oligothymidine 16mer by **Ru-1** and **AM** at 70 °C. Lane 1: Ru-free control, normalized to 100% for band intensity quantification (BQ). Lanes 2-3: 1 equiv **Ru-1** or **AM**; BQ 87% or 55%. Lanes 4-5: 1 equiv **Ru-1** or **AM** in 0.4 M NaCl; BQ 92% or 77%. Lanes 6-7: 10 equiv **Ru-1** or **AM**, BQ 0%. Lanes 8-9: 10 equiv **Ru-1** or **AM** in 0.4 M NaCl; BQ 62% or 0%.

Table S5: 16mer T-FAM backbone degradation assay comparing **Ru-1** and **AM** at 70 °C. DNA was analyzed by gel electrophoresis and HPLC. DNA degradation upon treatment with either **Ru-1** or **AM** was calculated based on product peak integration vs the non-treated control.

Entry	Catalyst (equiv)	Additives (equiv)	DNA quantification by electrophoresis (%)	DNA quantification by HPLC analysis (Area under the curve, %)
1	None	None	100	100
2	1 **Ru-1**	None	87	74
3	1 **AM**	None	55	45
4	1 **Ru-1**	40,000 NaCl	92	87
5	1 **AM**	40,000 NaCl	77	69
6	10 **Ru-1**	None	0	0
7	10 **AM**	None	0	0
8	10 **Ru-1**	40,000 NaCl	62	73
9	10 **AM**	40,000 NaCl	0	0

RP-HPLC chromatograms of 16mer T backbone degradation assay at 70 °C.

Entry 1: Control

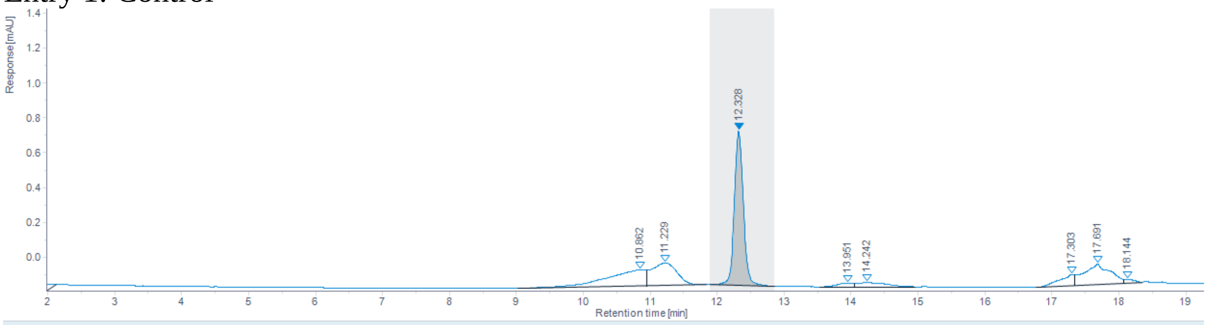

Injection Results

Peaks Summary

#	Name	Signal description	RT (min)	Area (mAU·s)	Area%	Height (mAU)	Height%	Amount	Concentration	Start time (min)	End time (min)
3		DAD1A,Sig=260,4 Ref=off	1.546	2253.686	44.309	646.625	76.25			1.486	2.142
4		DAD1A,Sig=260,4 Ref=off	10.862	373.826	7.350	9.278	1.09			9.027	10.952
5		DAD1A,Sig=260,4 Ref=off	11.229	343.996	6.763	12.936	1.53			10.952	11.848
6		DAD1A,Sig=260,4 Ref=off	12.328	835.556	16.427	88.478	10.43			11.900	12.860

Entry 2: 1 equiv **Ru-1**, no additives

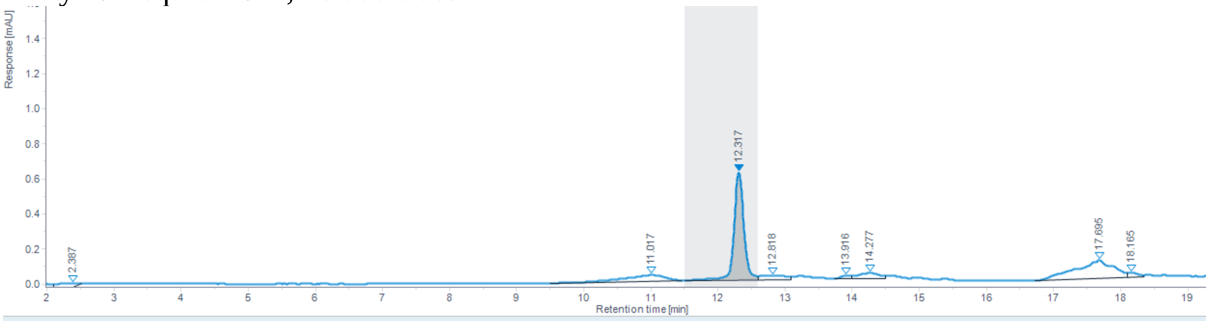

Injection Results

Peaks Summary

#	Name	Signal description	RT (min)	Area (mAU·s)	Area%	Height (mAU)	Height%	Amount	Concentration	Start time (min)	End time (min)
3		DAD1A,Sig=260,4 Ref=off	1.539	2332.442	53.901	611.015	81.96			1.475	2.106
4		DAD1A,Sig=260,4 Ref=off	2.387	81.915	1.893	2.155	0.29			2.106	2.517
5		DAD1A,Sig=260,4 Ref=off	11.017	145.195	3.355	3.358	0.45			9.510	11.465
6		DAD1A,Sig=260,4 Ref=off	12.317	616.749	14.253	61.217	8.21			11.517	12.601

Entry 3: 1 equiv **AM**, no additives

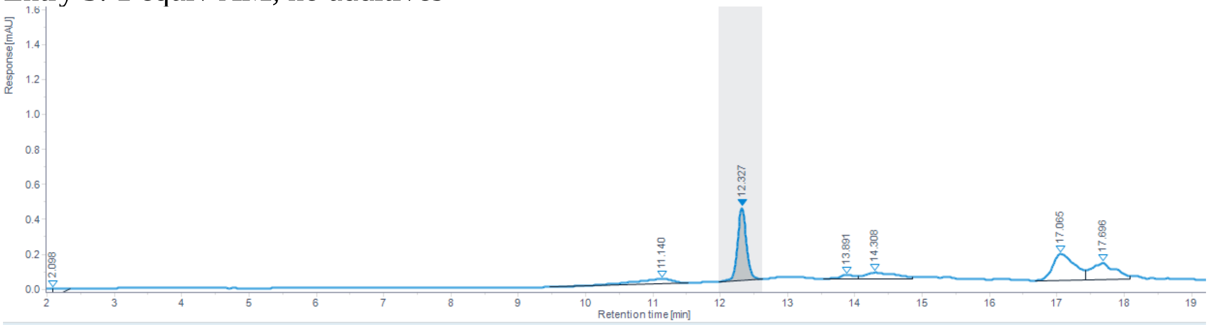

Injection Results

Peaks Summary

#	Name	Signal description	RT (min)	Area (mAU·s)	Area%	Height (mAU)	Height%	Amount	Concentration	Start time (min)	End time (min)
2		DAD1A,Sig=260,4 Ref=off	1.458	73.773	2.189	23.188	4.67			1.404	1.483
3		DAD1A,Sig=260,4 Ref=off	1.543	1494.869	44.366	368.690	74.30			1.483	2.096
4		DAD1A,Sig=260,4 Ref=off	2.098	35.314	1.048	4.581	0.92			2.096	2.349
5		DAD1A,Sig=260,4 Ref=off	11.140	118.569	3.519	2.801	0.56			9.477	11.533
6		DAD1A,Sig=260,4 Ref=off	12.327	378.700	11.239	41.499	8.36			11.984	12.633

Entry 4: 1 equiv **Ru-1**, 40,000 equiv NaCl

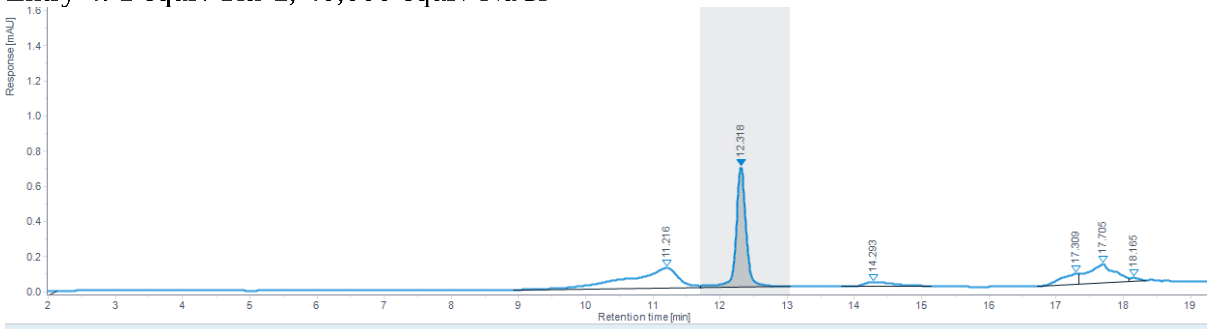

Injection Results

Peaks | Summary

#	Name	Signal description	RT (min)	Area (mAU·s)	Area%	Height (mAU)	Height%	Amount	Concentration	Start time (min)	End time (min)
2		DAD1A,Sig=260,4 Ref=off	1.313	90.012	1.882	15.066	1.84			1.252	1.389
3		DAD1A,Sig=260,4 Ref=off	1.463	65.115	1.361	16.576	2.02			1.396	1.482
4		DAD1A,Sig=260,4 Ref=off	1.545	2323.588	48.572	663.318	80.93			1.482	2.130
5		DAD1A,Sig=260,4 Ref=off	11.216	597.852	12.497	11.496	1.40			8.929	11.719
6		DAD1A,Sig=260,4 Ref=off	12.318	724.277	15.140	68.490	8.36			11.719	13.052

Entry 5: 1 equiv **AM**, 40,000 equiv NaCl

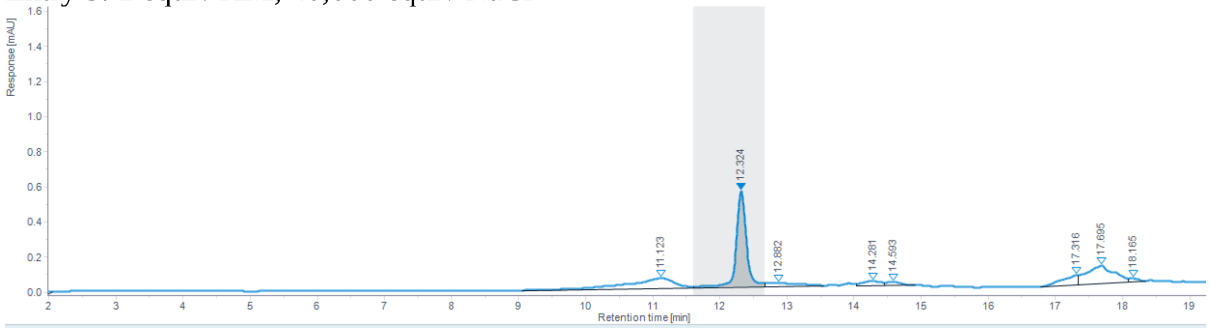

Injection Results

Peaks | Summary

#	Name	Signal description	RT (min)	Area (mAU·s)	Area%	Height (mAU)	Height%	Amount	Concentration	Start time (min)	End time (min)
2		DAD1A,Sig=260,4 Ref=off	1.312	91.582	2.200	15.608	2.11			1.253	1.388
3		DAD1A,Sig=260,4 Ref=off	1.461	63.403	1.523	16.066	2.17			1.389	1.478
4		DAD1A,Sig=260,4 Ref=off	1.543	2128.532	51.133	598.182	80.81			1.478	2.061
5		DAD1A,Sig=260,4 Ref=off	11.123	271.196	6.515	5.604	0.76			9.054	11.617
6		DAD1A,Sig=260,4 Ref=off	12.324	578.248	13.891	54.657	7.38			11.617	12.686

Entry 6: 10 equiv **Ru-1**, no additives

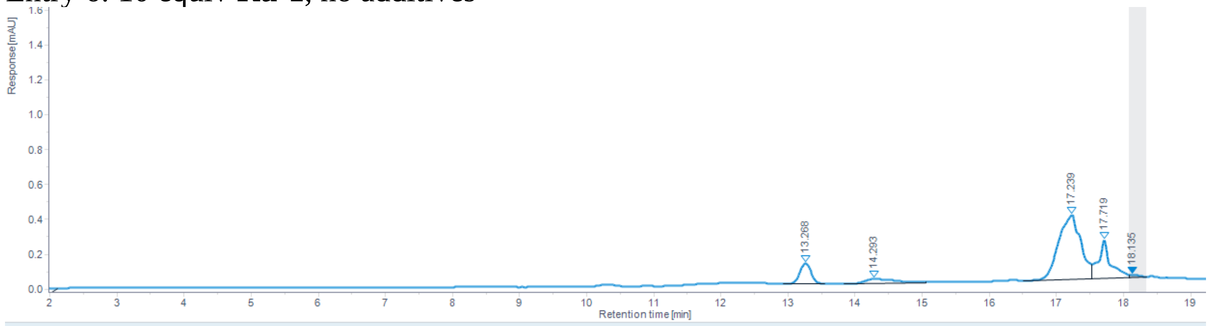

Injection Results

Peaks | Summary

#	Name	Signal description	RT (min)	Area (mAU·s)	Area%	Height (mAU)	Height%	Amount	Concentration	Start time (min)	End time (min)
2		DAD1A,Sig=260,4 Ref=off	1.148	27.734	1.096	5.792	3.19			1.104	1.186
3		DAD1A,Sig=260,4 Ref=off	1.317	157.439	6.219	17.216	9.50			1.186	1.405
4		DAD1A,Sig=260,4 Ref=off	1.460	83.194	3.286	22.860	12.61			1.408	1.496
5		DAD1A,Sig=260,4 Ref=off	1.547	117.839	4.655	25.138	13.86			1.496	1.589
6		DAD1A,Sig=260,4 Ref=off	1.610	267.336	10.560	18.529	10.22			1.589	2.128
7		DAD1A,Sig=260,4 Ref=off	13.268	140.751	5.560	11.757	6.48			12.934	13.551

Entry 7: 10 equiv **AM**, no additives

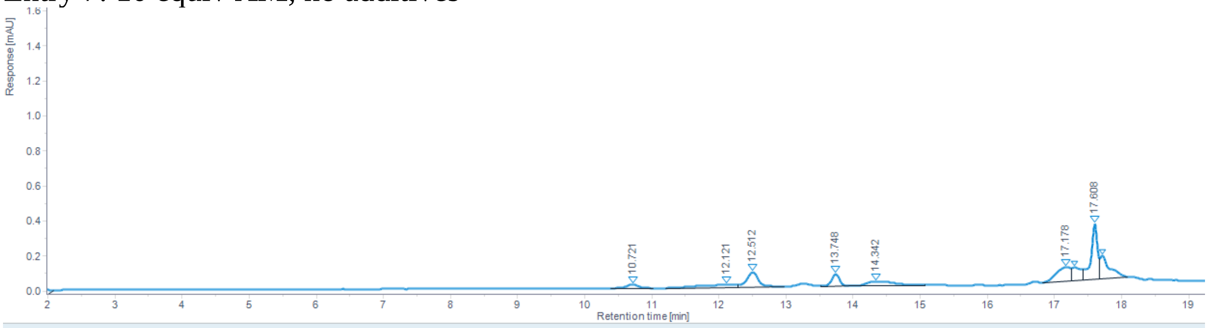

Injection Results

Peaks Summary

#	Name	Signal description	RT (min)	Area (mAU·s)	Area%	Height (mAU)	Height%	Amount	Concentration	Start time (min)	End time (min)
1		DAD1A,Sig=260,4 Ref=off	1.163	16.417	0.865	3.429	2.16			1.107	1.202
2		DAD1A,Sig=260,4 Ref=off	1.320	140.198	7.389	15.965	10.06			1.202	1.413
3		DAD1A,Sig=260,4 Ref=off	1.466	89.167	4.699	22.826	14.38			1.413	1.509
4		DAD1A,Sig=260,4 Ref=off	1.577	353.898	18.652	24.717	15.58			1.509	2.090
5		DAD1A,Sig=260,4 Ref=off	10.721	27.666	1.458	2.239	1.41			10.393	11.026
6		DAD1A,Sig=260,4 Ref=off	12.121	59.571	3.140	1.742	1.10			11.220	12.289

Entry 8: 10 equiv **Ru-1**, 40,000 equiv NaCl

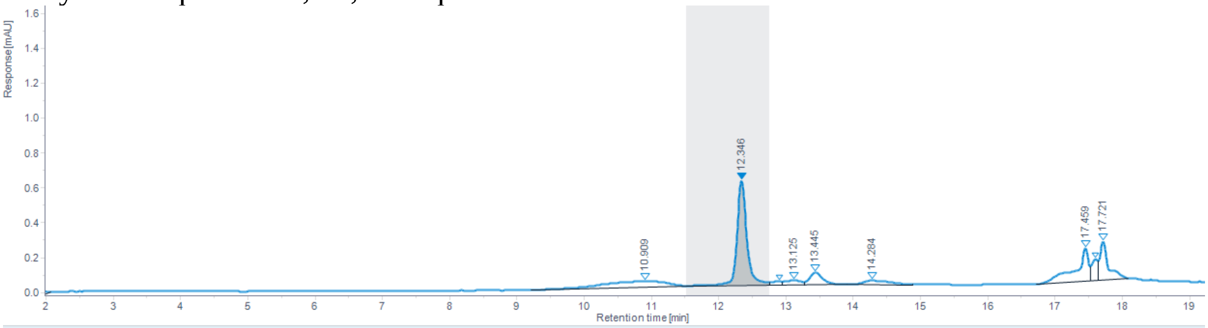

Injection Results

Peaks Summary

#	Name	Signal description	RT (min)	Area (mAU·s)	Area%	Height (mAU)	Height%	Amount	Concentration	Start time (min)	End time (min)
6		DAD1A,Sig=260,4 Ref=off	10.909	216.201	4.590	3.288	0.39			9.219	11.526
7		DAD1A,Sig=260,4 Ref=off	12.346	613.236	13.019	59.706	7.05			11.526	12.759

Entry 9: 10 equiv **AM**, 40,000 equiv NaCl

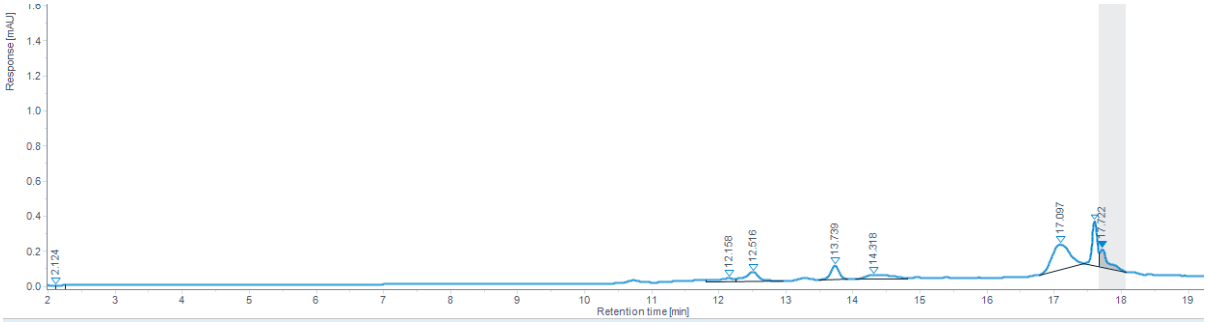

Injection Results

Peaks Summary

#	Name	Signal description	RT (min)	Area (mAU·s)	Area%	Height (mAU)	Height%	Amount	Concentration	Start time (min)	End time (min)
3		DAD1A,Sig=260,4 Ref=off	1.475	82.612	4.467	17.095	11.41			1.405	1.512
4		DAD1A,Sig=260,4 Ref=off	1.581	410.507	22.195	22.245	14.85			1.512	2.123
5		DAD1A,Sig=260,4 Ref=off	2.124	35.057	1.895	4.863	3.25			2.123	2.270
6		DAD1A,Sig=260,4 Ref=off	12.158	33.001	1.784	1.796	1.20			11.824	12.255
7		DAD1A,Sig=260,4 Ref=off	12.516	77.599	4.196	5.402	3.61			12.255	12.967
8		DAD1A,Sig=260,4 Ref=off	13.739	68.986	3.730	7.837	5.23			13.497	13.928

Figure S13. RP-HPLC traces for **Table S5** (entries 1-9) of DNA backbone of 16mer oligothymidine-FAM model during aqueous metathesis using **Ru-1** or **AM** at 70 °C.

16mer DNA-FAM (3´-TCCACGCTTAGCTCTC-5´-6-FAM), 25°C

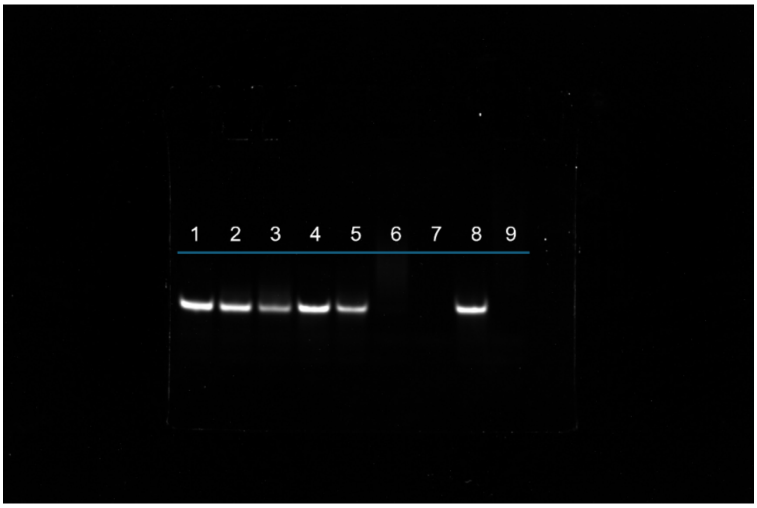

Figure S14. Backbone degradation of fluorescein-labelled native DNA 16mer by **Ru-1** and **AM** at 25 °C. Lane 1: Ru-free control, normalized to 100% for band intensity quantification (BQ). Lanes 2-3: 1 equiv **Ru-1** or **AM**; BQ 78% or 44%. Lanes 4-5: 1 equiv **Ru-1** or **AM** in 0.4 M NaCl; BQ 85% or 49%. Lanes 6-7: 10 equiv **Ru-1** or **AM**, BQ 0%. Lanes 8-9: 10 equiv **Ru-1** or **AM** in 0.4 M NaCl; BQ 76% or 0%.

Table S6: 16mer DNA-FAM backbone degradation assay comparing **Ru-1** and **AM** at 25 °C. DNA was analyzed by gel electrophoresis and HPLC. DNA degradation upon treatment with either **Ru-1** or **AM** was calculated based on product peak integration vs the non-treated control.

**Entry**	**Catalyst (equiv)**	**Additives (equiv)**	**DNA quantification by electrophoresis (%)**	**DNA quantification by HPLC analysis (Area under the curve, %)**
1	None	None	100	100
2	1 **Ru-1**	None	78	88
3	1 **AM**	None	44	56
4	1 **Ru-1**	40,000 NaCl	85	90
5	1 **AM**	40,000 NaCl	49	64
6	10 **Ru-1**	None	0	0
7	10 **AM**	None	0	0
8	10 **Ru-1**	40,000 NaCl	76	74
9	10 **AM**	40,000 NaCl	0	0

RP-HPLC chromatograms of 16mer native DNA backbone degradation assay at 25 °C.
Entry 1: Control

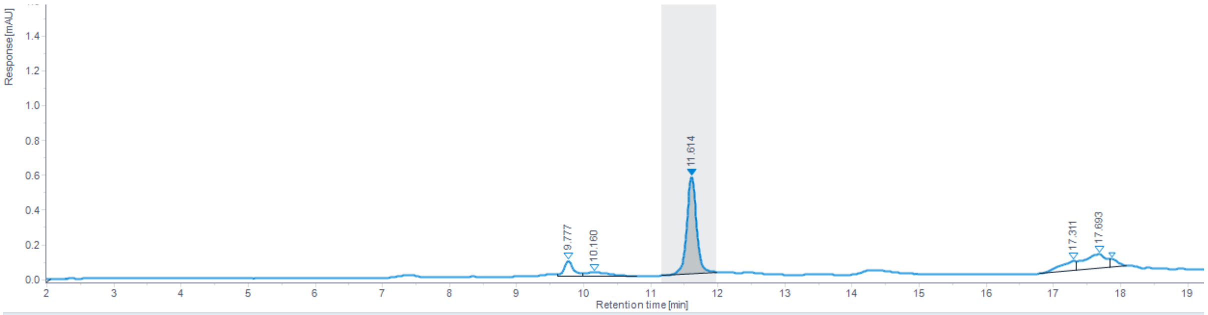

Injection Results

Peaks | Summary

#	Name	Signal description	RT (min)	Area (mAU·s)	Area%	Height (mAU)	Height%	Amount	Concentration	Start time (min)	End time (min)
1		DAD1A,Sig=260,4 Ref=off	1.318	117.236	2.734	15.115	1.69			1.167	1.400
2		DAD1A,Sig=260,4 Ref=off	1.457	67.639	1.578	22.665	2.54			1.402	1.479
3		DAD1A,Sig=260,4 Ref=off	1.543	2642.857	61.640	760.947	85.11			1.479	2.063
4		DAD1A,Sig=260,4 Ref=off	9.777	88.611	2.067	8.781	0.98			9.620	9.989
5		DAD1A,Sig=260,4 Ref=off	10.160	55.527	1.295	2.067	0.23			9.989	10.793
6		DAD1A,Sig=260,4 Ref=off	11.614	557.179	12.995	55.695	6.23			11.167	11.997

Entry 2: 1 equiv **Ru-1**, no additives

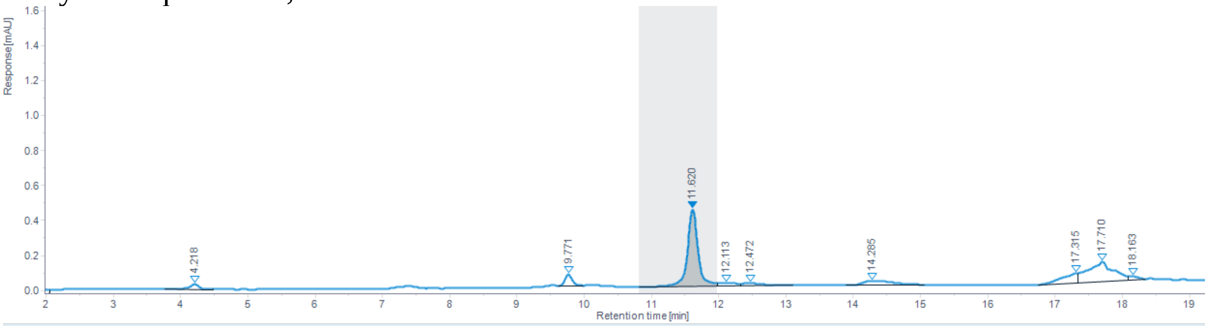

Injection Results

Peaks | Summary

#	Name	Signal description	RT (min)	Area (mAU·s)	Area%	Height (mAU)	Height%	Amount	Concentration	Start time (min)	End time (min)
1		DAD1A,Sig=260,4 Ref=off	1.186	66.113	1.359	14.418	1.50			1.132	1.252
2		DAD1A,Sig=260,4 Ref=off	1.311	86.859	1.785	15.106	1.57			1.252	1.385
3		DAD1A,Sig=260,4 Ref=off	1.460	59.148	1.216	16.387	1.70			1.386	1.470
4		DAD1A,Sig=260,4 Ref=off	1.539	3100.182	63.717	828.484	86.04			1.470	2.063
5		DAD1A,Sig=260,4 Ref=off	4.218	30.914	0.635	2.905	0.30			3.780	4.496
6		DAD1A,Sig=260,4 Ref=off	9.771	51.362	1.056	6.563	0.68			9.626	9.987
7		DAD1A,Sig=260,4 Ref=off	11.620	491.214	10.096	43.825	4.55			10.832	11.990

Entry 3: 1 equiv **AM**, no additives

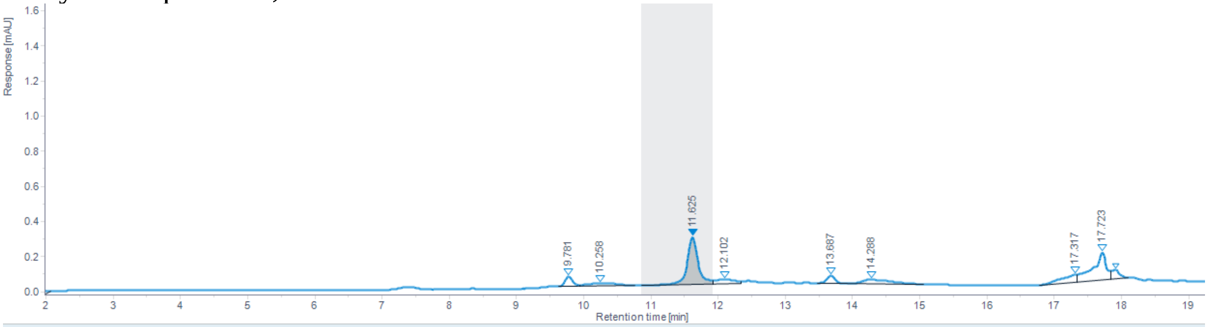

Injection Results

Peaks | Summary

#	Name	Signal description	RT (min)	Area (mAU·s)	Area%	Height (mAU)	Height%	Amount	Concentration	Start time (min)	End time (min)
1		DAD1A,Sig=260,4 Ref=off	1.187	72.370	1.993	15.853	2.24			1.137	1.252
2		DAD1A,Sig=260,4 Ref=off	1.312	96.298	2.652	15.754	2.22			1.252	1.390
3		DAD1A,Sig=260,4 Ref=off	1.464	65.017	1.791	16.324	2.31			1.393	1.483
4		DAD1A,Sig=260,4 Ref=off	1.547	2046.148	56.351	580.576	82.00			1.483	2.075
5		DAD1A,Sig=260,4 Ref=off	9.781	46.767	1.288	5.807	0.82			9.637	9.953
6		DAD1A,Sig=260,4 Ref=off	10.258	51.150	1.409	1.751	0.25			9.953	10.776
7		DAD1A,Sig=260,4 Ref=off	11.625	314.861	8.671	26.980	3.81			10.864	11.928

Entry 4: 1 equiv **Ru-1**, 40,000 equiv NaCl

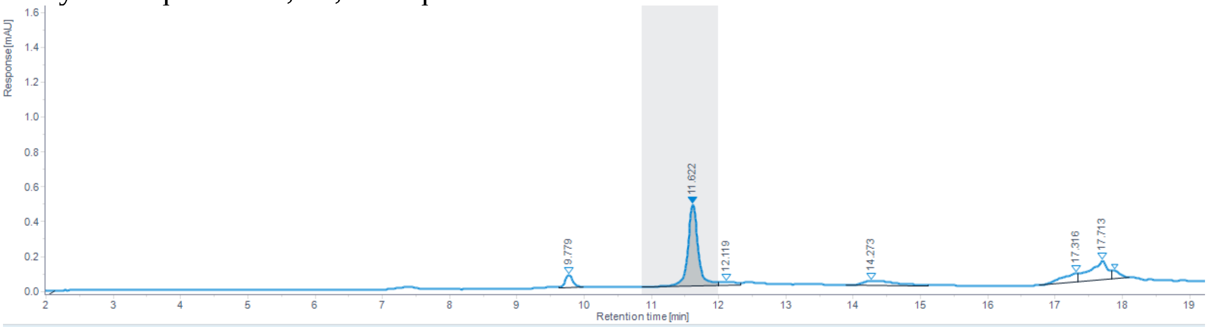

Injection Results

Peaks Summary

#	Name	Signal description	RT (min)	Area (mAU·s)	Area%	Height (mAU)	Height%	Amount	Concentration	Start time (min)	End time (min)
1		DAD1A,Sig=260,4 Ref=off	1.317	123.270	2.887	15.556	1.85			1.147	1.393
2		DAD1A,Sig=260,4 Ref=off	1.454	63.994	1.499	22.819	2.71			1.400	1.471
3		DAD1A,Sig=260,4 Ref=off	1.537	2640.451	61.841	713.786	84.83			1.471	2.135
4		DAD1A,Sig=260,4 Ref=off	9.779	54.056	1.266	7.024	0.83			9.628	9.995
5		DAD1A,Sig=260,4 Ref=off	11.622	500.941	11.732	46.630	5.54			10.867	12.002

Entry 5: 1 equiv **AM**, 40,000 NaCl

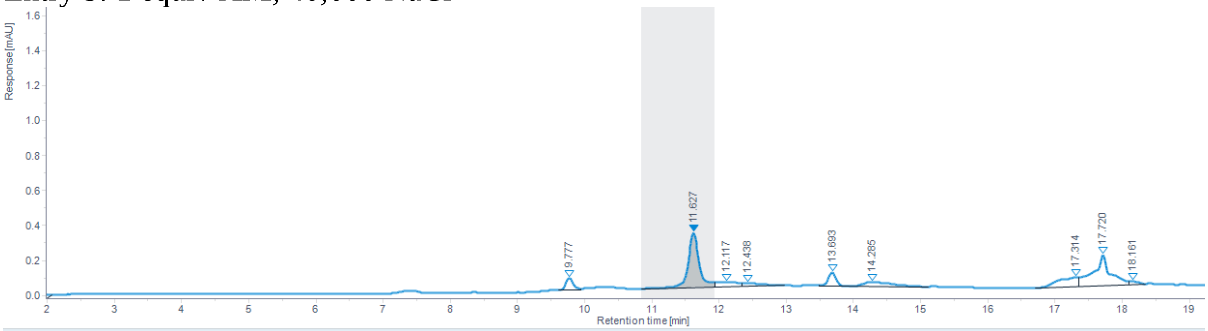

Injection Results

Peaks Summary

#	Name	Signal description	RT (min)	Area (mAU·s)	Area%	Height (mAU)	Height%	Amount	Concentration	Start time (min)	End time (min)
1		DAD1A,Sig=260,4 Ref=off	1.317	92.890	2.608	13.062	2.02			1.213	1.406
2		DAD1A,Sig=260,4 Ref=off	1.460	72.943	2.048	22.870	3.53			1.407	1.487
3		DAD1A,Sig=260,4 Ref=off	1.547	1856.256	52.108	523.514	80.87			1.487	2.078
4		DAD1A,Sig=260,4 Ref=off	9.777	53.717	1.508	6.794	1.05			9.620	9.955
5		DAD1A,Sig=260,4 Ref=off	11.627	355.016	9.966	31.048	4.80			10.860	11.948

Entry 6: 10 equiv **Ru-1**, no additives

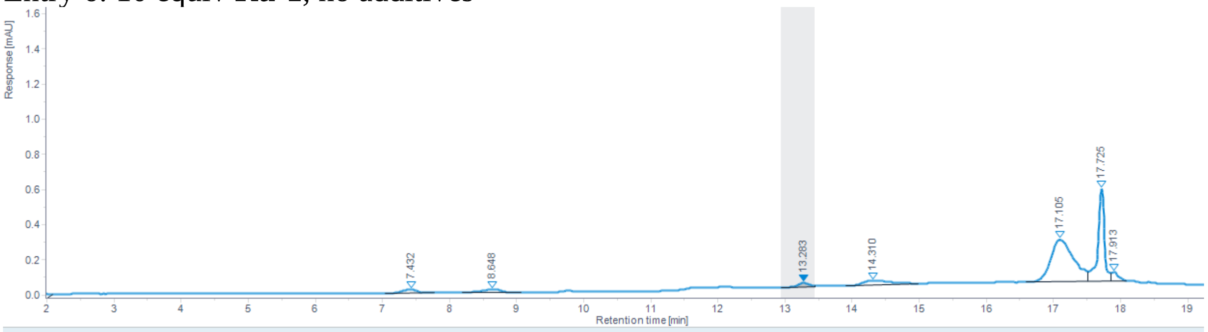

Injection Results

Peaks Summary

#	Name	Signal description	RT (min)	Area (mAU·s)	Area%	Height (mAU)	Height%	Amount	Concentration	Start time (min)	End time (min)
1		DAD1A,Sig=260,4 Ref=off	1.317	171.909	8.034	16.362	8.23			1.084	1.410
2		DAD1A,Sig=260,4 Ref=off	1.466	83.551	3.905	22.841	11.49			1.417	1.504
3		DAD1A,Sig=260,4 Ref=off	1.559	446.424	20.863	55.327	27.84			1.504	2.088
4		DAD1A,Sig=260,4 Ref=off	7.432	28.671	1.340	1.953	0.98			7.044	7.783
5		DAD1A,Sig=260,4 Ref=off	8.648	38.726	1.810	1.872	0.94			8.197	9.076
6		DAD1A,Sig=260,4 Ref=off	13.283	31.140	1.455	2.401	1.21			12.950	13.453

Entry 7: 10 equiv **AM**, no additives

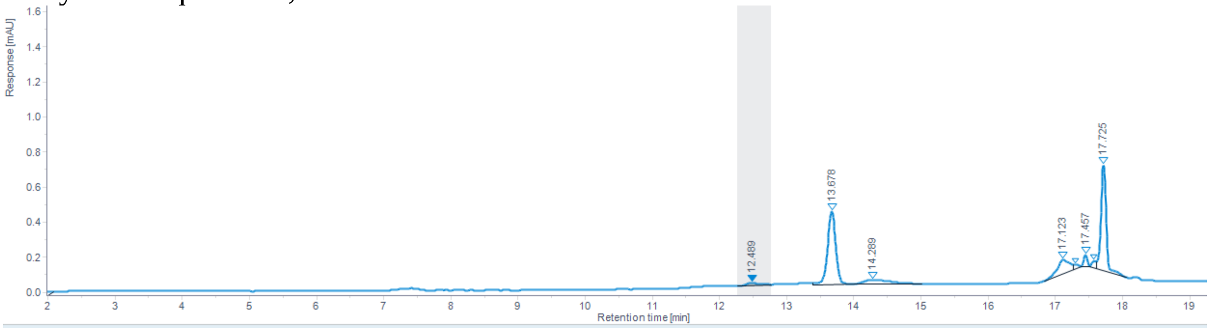

Injection Results

Peaks | Summary

#	Name	Signal description	RT (min) △	Area (mAU·s)	Area%	Height (mAU)	Height%	Amount	Concentration	Start time (min)	End time (min)
1		DAD1A,Sig=260,4 Ref=off	1.317	90.179	4.779	13.287	6.33			1.214	1.400
2		DAD1A,Sig=260,4 Ref=off	1.457	83.491	4.425	22.709	10.82			1.401	1.493
3		DAD1A,Sig=260,4 Ref=off	1.544	97.312	5.157	20.164	9.60			1.493	1.580
4		DAD1A,Sig=260,4 Ref=off	1.610	257.932	13.670	18.045	8.59			1.580	2.098
5		DAD1A,Sig=260,4 Ref=off	12.489	24.768	1.313	1.749	0.83			12.280	12.778

Entry 8: 10 equiv **Ru-1**, 40,000 equiv NaCl

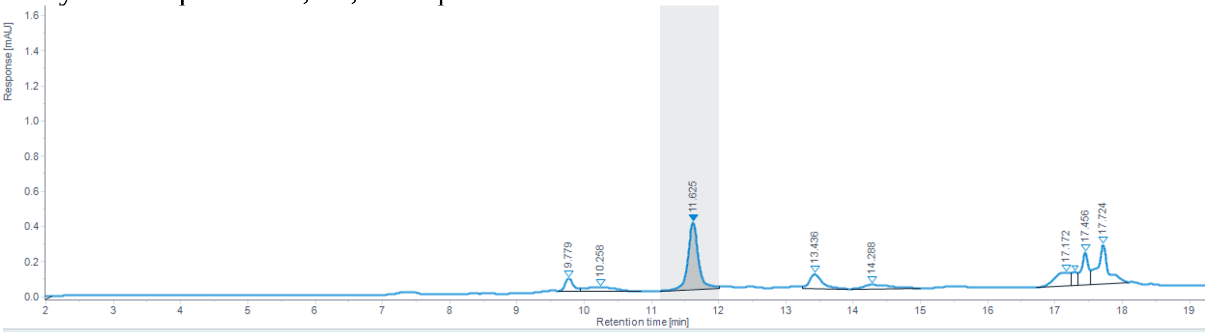

Injection Results

Peaks | Summary

#	Name	Signal description	RT (min) △	Area (mAU·s)	Area%	Height (mAU)	Height%	Amount	Concentration	Start time (min)	End time (min)
5		DAD1A,Sig=260,4 Ref=off	9.779	59.957	1.320	7.216	0.78			9.621	9.947
6		DAD1A,Sig=260,4 Ref=off	10.258	69.272	1.525	2.193	0.24			9.947	10.860
7		DAD1A,Sig=260,4 Ref=off	11.625	415.259	9.141	38.077	4.14			11.147	12.024

Entry 9: 10 equiv **AM**, 40,000 equiv NaCl

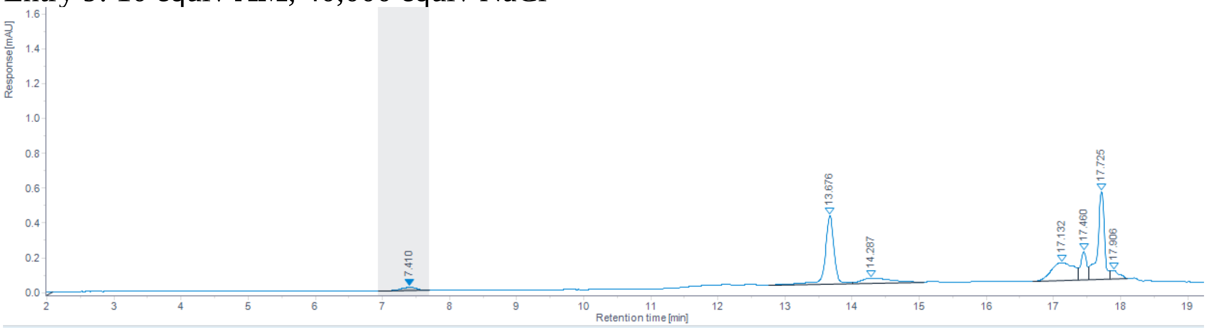

Injection Results

Peaks | Summary

#	Name	Signal description	RT (min) △	Area (mAU·s)	Area%	Height (mAU)	Height%	Amount	Concentration	Start time (min)	End time (min)
4		DAD1A,Sig=260,4 Ref=off	1.552	382.665	16.152	46.355	19.75			1.496	2.089
5		DAD1A,Sig=260,4 Ref=off	7.410	31.635	1.335	1.854	0.79			6.948	7.713
6		DAD1A,Sig=260,4 Ref=off	13.676	355.620	15.011	39.026	16.63			12.759	14.008
7		DAD1A,Sig=260,4 Ref=off	14.287	96.815	4.087	2.931	1.25			14.008	15.078

Figure S15. RP-HPLC traces for **Table S6** (entries 1-9) of DNA backbone of 16mer DNA-FAM model during aqueous metathesis using **Ru-1** or **AM** at 25 °C.

16mer DNA-FAM (3´-TCCACGCTTAGCTCTC-5´-6-FAM), 70 °C

Entry	Catalyst (equiv)	Additives (equiv)	DNA quantification by electrophoresis (%)	DNA quantification by HPLC analysis (Area under the curve, %)
1	None	None	100	100
2	1 **Ru-1**	None	78	50
3	1 **AM**	None	50	41
4	1 **Ru-1**	40,000 NaCl	83	67
5	1 **AM**	40,000 NaCl	60	45
6	10 **Ru-1**	None	0	0
7	10 **AM**	None	0	0
8	10 **Ru-1**	40,000 NaCl	57	58
9	10 **AM**	40,000 NaCl	0	0

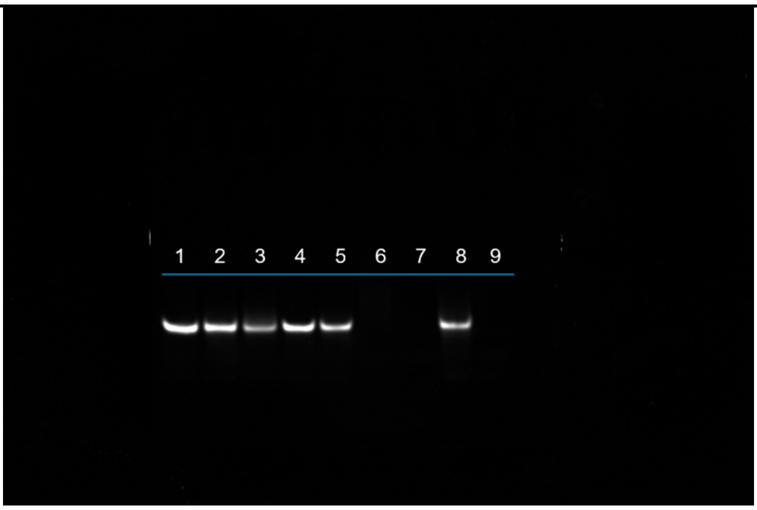

Figure S16. Backbone degradation of fluorescein-labelled 16mer native DNA by **Ru-1** and **AM** at 70 °C. Lane 1: Ru-free control, normalized to 100% for band intensity quantification (BQ). Lanes 2-3: 1 equiv **Ru-1** or **AM**; BQ 78% or 50%. Lanes 4-5: 1 equiv **Ru-1** or **AM** in 0.4 M NaCl; BQ 83% or 60%. Lanes 6-7: 10 equiv **Ru-1** or **AM**, BQ 0%. Lanes 8-9: 10 equiv **Ru-1** or **AM** in 0.4 M NaCl; BQ 57% or 0%.

Table S7: 16mer DNA-FAM backbone degradation assay comparing **Ru-1** and **AM** at 70 °C. DNA was analyzed by gel electrophoresis and HPLC. DNA degradation upon treatment with either **Ru-1** or **AM** was calculated based on product peak integration vs the non-treated control.

RP-HPLC traces of 16mer native DNA backbone degradation assay at 70 °C.

Entry 1: Control

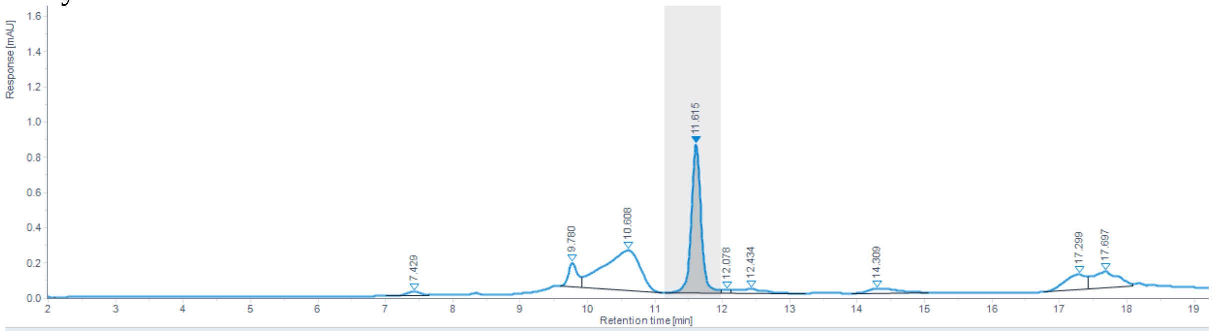

Injection Results

Peaks Summary

#	Name	Signal description	RT (min)	Area (mAU·s)	Area%	Height (mAU)	Height%	Amount	Concentration	Start time (min)	End time (min)
4		DAD1A,Sig=260,4 Ref=off	7.429	33.164	0.690	2.166	0.31			7.021	7.655
5		DAD1A,Sig=260,4 Ref=off	9.780	125.020	2.602	13.237	1.89			9.600	9.922
6		DAD1A,Sig=260,4 Ref=off	10.608	849.398	17.679	22.627	3.23			9.922	11.115
7		DAD1A,Sig=260,4 Ref=off	11.615	852.012	17.733	84.155	12.03			11.151	11.987

Entry 2: 1 equiv **Ru-1**, no additives

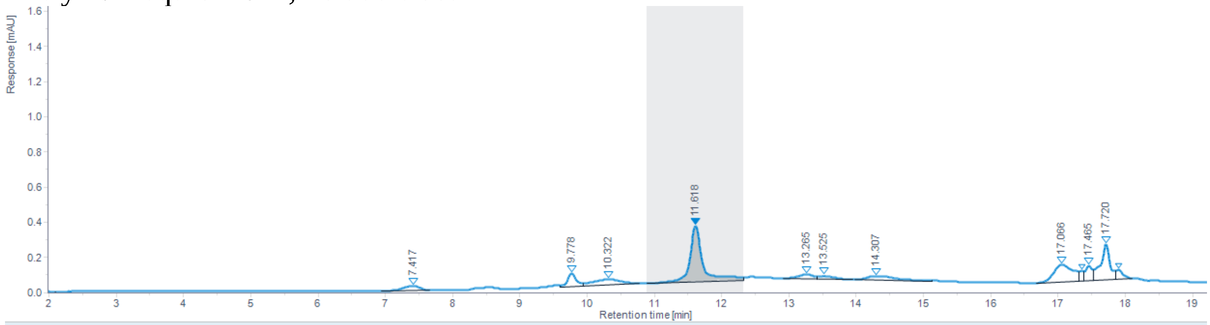

Injection Results

Peaks Summary

#	Name	Signal description	RT (min)	Area (mAU·s)	Area%	Height (mAU)	Height%	Amount	Concentration	Start time (min)	End time (min)
8		DAD1A,Sig=260,4 Ref=off	10.322	91.659	1.945	3.064	0.32			9.945	10.787
9		DAD1A,Sig=260,4 Ref=off	11.618	422.311	8.959	31.574	3.26			10.893	12.329

Entry 3: 1 equiv **AM**, no additives

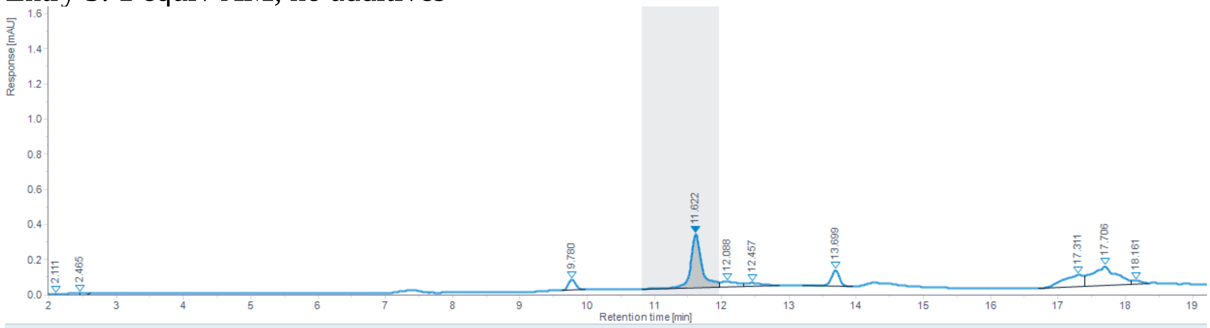

Injection Results

Peaks Summary

#	Name	Signal description	RT (min)	Area (mAU·s)	Area%	Height (mAU)	Height%	Amount	Concentration	Start time (min)	End time (min)
5		DAD1A,Sig=260,4 Ref=off	2.111	102.051	2.430	7.184	0.94			2.106	2.460
6		DAD1A,Sig=260,4 Ref=off	2.465	10.998	0.262	2.188	0.29			2.460	2.618
7		DAD1A,Sig=260,4 Ref=off	9.780	47.622	1.134	6.173	0.81			9.629	9.978
8		DAD1A,Sig=260,4 Ref=off	11.622	348.158	8.289	30.189	3.96			10.831	11.974

Entry 4: 1 equiv **Ru-1**, 40,000 equiv NaCl

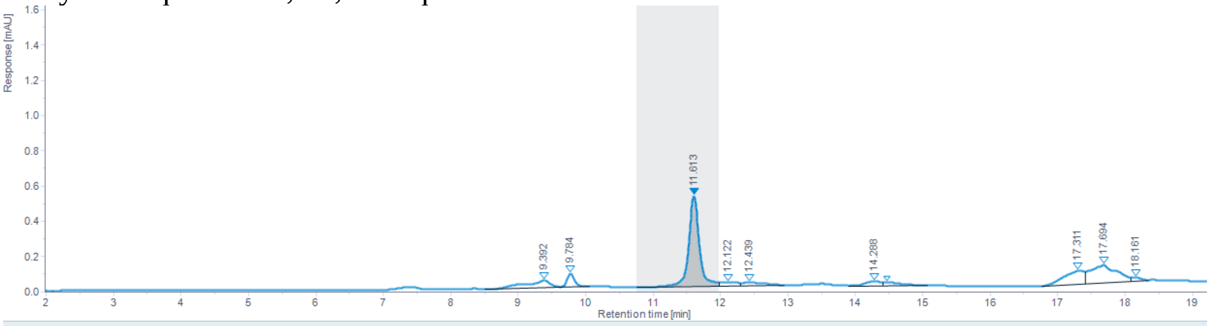

Injection Results

Peaks | Summary

#	Name	Signal description	RT (min)	Area (mAU·s)	Area%	Height (mAU)	Height%	Amount	Concentration	Start time (min)	End time (min)
5		DAD1A,Sig=260,4 Ref=off	9.784	57.867	1.194	7.445	0.78			9.630	10.065
6		DAD1A,Sig=260,4 Ref=off	11.613	572.046	11.800	51.264	5.35			10.773	11.990

Entry 5: 1 equiv **AM**, 40,000 equiv NaCl

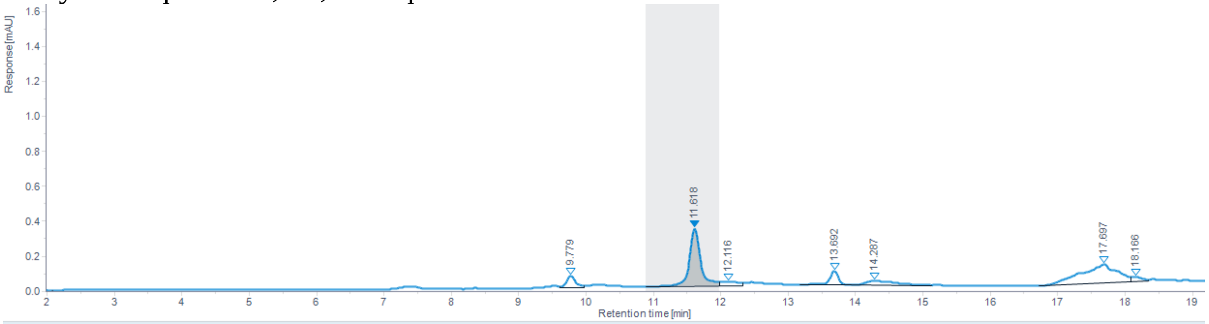

Injection Results

Peaks | Summary

#	Name	Signal description	RT (min)	Area (mAU·s)	Area%	Height (mAU)	Height%	Amount	Concentration	Start time (min)	End time (min)
3		DAD1A,Sig=260,4 Ref=off	1.461	62.985	1.528	16.498	2.10			1.390	1.475
4		DAD1A,Sig=260,4 Ref=off	1.542	2395.810	58.124	663.415	84.29			1.475	2.095
5		DAD1A,Sig=260,4 Ref=off	9.779	65.148	1.581	6.924	0.88			9.623	9.978
6		DAD1A,Sig=260,4 Ref=off	11.618	381.105	9.246	32.904	4.18			10.903	11.991

Entry 6: 10 equiv **Ru-1**, no additives

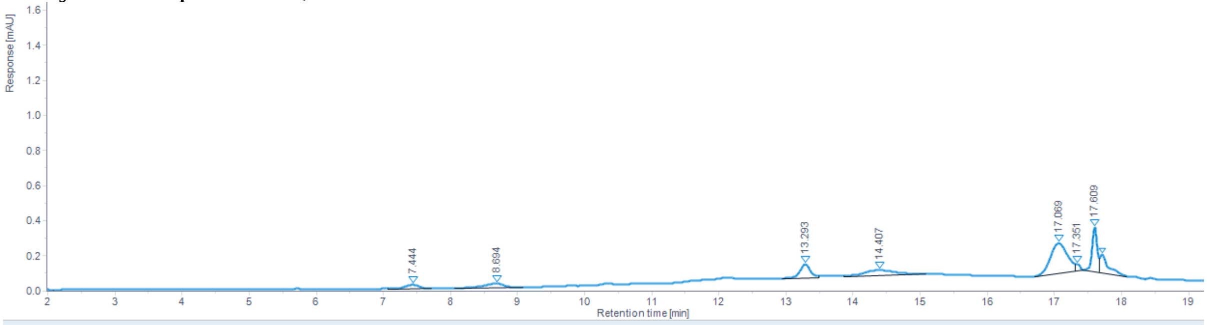

Injection Results

Peaks | Summary

#	Name	Signal description	RT (min)	Area (mAU·s)	Area%	Height (mAU)	Height%	Amount	Concentration	Start time (min)	End time (min)
1		DAD1A,Sig=260,4 Ref=off	1.319	134.375	6.446	14.946	7.68			1.154	1.414
2		DAD1A,Sig=260,4 Ref=off	1.469	85.293	4.092	22.786	11.71			1.416	1.508
3		DAD1A,Sig=260,4 Ref=off	1.574	501.336	24.049	68.752	35.32			1.508	2.091
4		DAD1A,Sig=260,4 Ref=off	7.444	30.176	1.448	2.103	1.08			7.080	7.724
5		DAD1A,Sig=260,4 Ref=off	8.694	50.220	2.409	2.374	1.22			8.074	9.093
6		DAD1A,Sig=260,4 Ref=off	13.293	81.503	3.910	7.559	3.88			12.948	13.495

Entry 7: 10 equiv **AM**, no additives

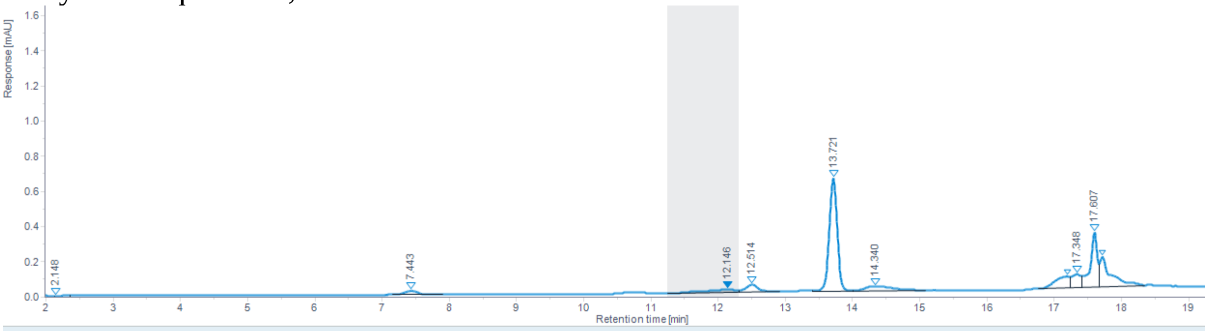

Injection Results

Peaks Summary

#	Name	Signal description	RT (min)	Area (mAU·s)	Area%	Height (mAU)	Height%	Amount	Concentration	Start time (min)	End time (min)
1		DAD1A,Sig=260,4 Ref=off	1.319	177.910	6.744	16.428	7.48			1.074	1.414
2		DAD1A,Sig=260,4 Ref=off	1.469	91.375	3.464	23.113	10.52			1.416	1.513
3		DAD1A,Sig=260,4 Ref=off	1.580	500.010	18.953	26.474	12.05			1.513	2.142
4		DAD1A,Sig=260,4 Ref=off	2.148	53.951	2.045	5.627	2.56			2.142	2.370
5		DAD1A,Sig=260,4 Ref=off	7.443	29.263	1.109	2.192	1.00			7.161	7.908
6		DAD1A,Sig=260,4 Ref=off	12.146	63.049	2.390	1.859	0.85			11.254	12.314
7		DAD1A,Sig=260,4 Ref=off	12.514	60.126	2.279	4.406	2.01			12.314	12.922

Entry 8: 10 equiv **Ru-1**, 40,000 NaCl

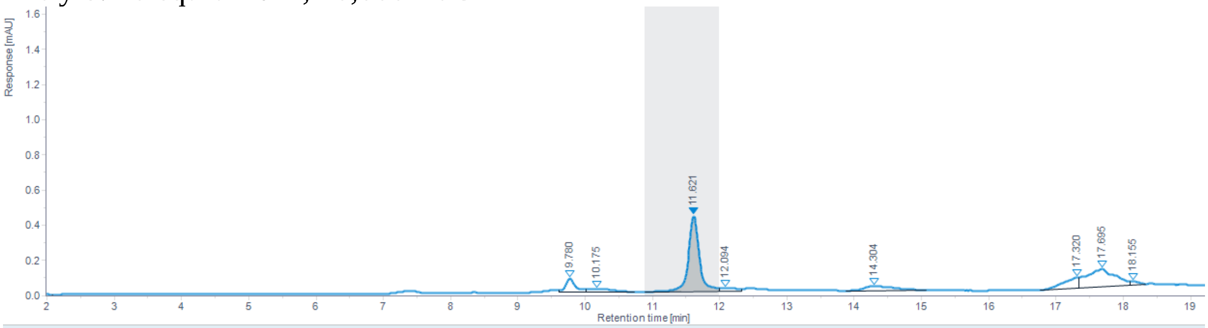

Injection Results

Peaks Summary

#	Name	Signal description	RT (min)	Area (mAU·s)	Area%	Height (mAU)	Height%	Amount	Concentration	Start time (min)	End time (min)
5		DAD1A,Sig=260,4 Ref=off	1.542	2746.844	60.095	770.279	85.04			1.476	2.090
6		DAD1A,Sig=260,4 Ref=off	9.780	82.216	1.799	7.783	0.86			9.625	10.022
7		DAD1A,Sig=260,4 Ref=off	10.175	46.565	1.019	1.826	0.20			10.022	10.743
8		DAD1A,Sig=260,4 Ref=off	11.621	490.516	10.732	42.865	4.73			10.891	12.003

Entry 9: 10 equiv **AM**, 40,000 NaCl

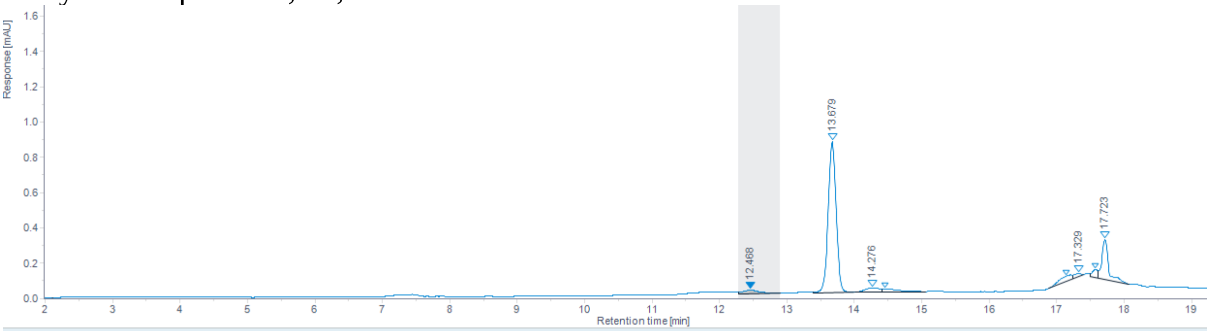

Injection Results

Peaks Summary

#	Name	Signal description	RT (min)	Area (mAU·s)	Area%	Height (mAU)	Height%	Amount	Concentration	Start time (min)	End time (min)
5		DAD1A,Sig=260,4 Ref=off	1.541	347.282	16.499	26.984	12.93			1.478	2.120
6		DAD1A,Sig=260,4 Ref=off	12.468	36.712	1.744	2.140	1.03			12.285	12.913
7		DAD1A,Sig=260,4 Ref=off	13.679	705.132	33.500	85.367	40.90			13.398	14.002

Figure S17. RP-HPLC traces for **Table S7** (entries 1-9) of DNA backbone of 16mer DNA-FAM model during aqueous metathesis using **Ru-1** or **AM** at 70 °C.

16mer RNA-FAM (3´-UCCACGCUUAGCUCUC-5´-6-FAM), 25 °C

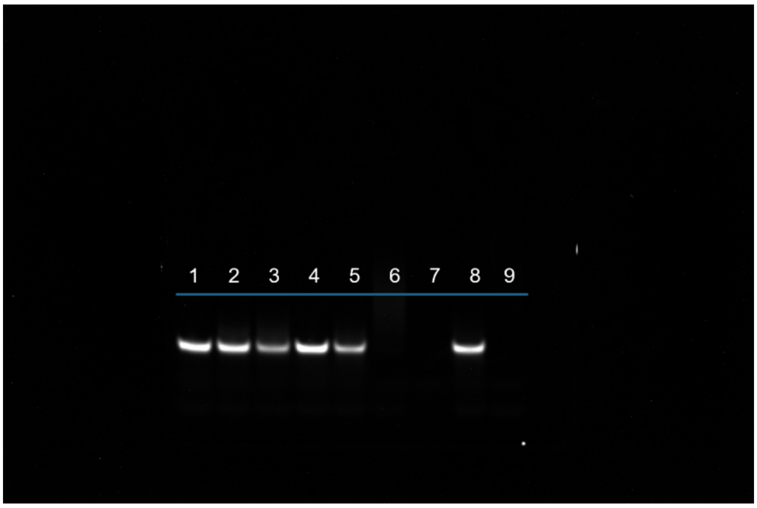

Figure S18. Backbone degradation of fluorescein-labelled 16mer native RNA by **Ru-1** and **AM** at 25 °C. Lane 1: Ru-free control, normalized to 100% for band intensity quantification (BQ). Lanes 2-3: 1 equiv **Ru-1** or **AM**; BQ 85% or 48%. Lanes 4-5: 1 equiv **Ru-1** or **AM** in 0.4 M NaCl; BQ 93% or 52%. Lanes 6-7: 10 equiv **Ru-1** or **AM**, BQ 0%. Lanes 8-9: 10 equiv **Ru-1** or **AM** in 0.4 M NaCl; BQ 70% or 0%.

Table S8: 16mer RNA-FAM backbone degradation assay comparing **Ru-1** and **AM** at 25 °C. RNA was analyzed by gel electrophoresis and HPLC. RNA degradation upon treatment with either **Ru-1** or **AM** was calculated based on product peak integration vs the non-treated control.

Entry	Catalyst (equiv)	Additives (equiv)	DNA quantification by electrophoresis (%)	DNA quantification by HPLC analysis (Area under the curve, %)
1	None	None	100	100
2	1 **Ru-1**	None	85	73
3	1 **AM**	None	48	40
4	1 **Ru-1**	40,000 NaCl	93	91
5	1 **AM**	40,000 NaCl	52	50
6	10 **Ru-1**	None	0	0
7	10 **AM**	None	0	0
8	10 **Ru-1**	40,000 NaCl	70	54
9	10 **AM**	40,000 NaCl	0	0

RP-HPLC chromatograms of 16mer native RNA backbone degradation assay at 25 °C.
Entry 1: Control

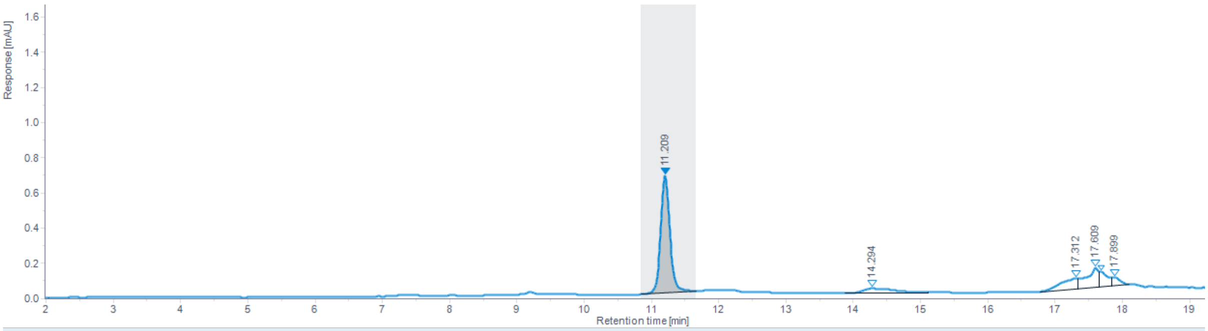

Injection Results

Peaks Summary

#	Name	Signal description	RT (min)	Area (mAU·s)	Area%	Height (mAU)	Height%	Amount	Concentration	Start time (min)	End time (min)
1		DAD1A,Sig=260,4 Ref=off	1.315	98.564	2.546	13.616	1.81			1.200	1.400
2		DAD1A,Sig=260,4 Ref=off	1.458	66.975	1.730	22.352	2.97			1.407	1.480
3		DAD1A,Sig=260,4 Ref=off	1.543	2176.209	56.222	607.934	80.66			1.480	2.050
4		DAD1A,Sig=260,4 Ref=off	11.209	642.989	16.612	66.505	8.82			10.853	11.671

Entry 2: 1 equiv **Ru-1**, no additives

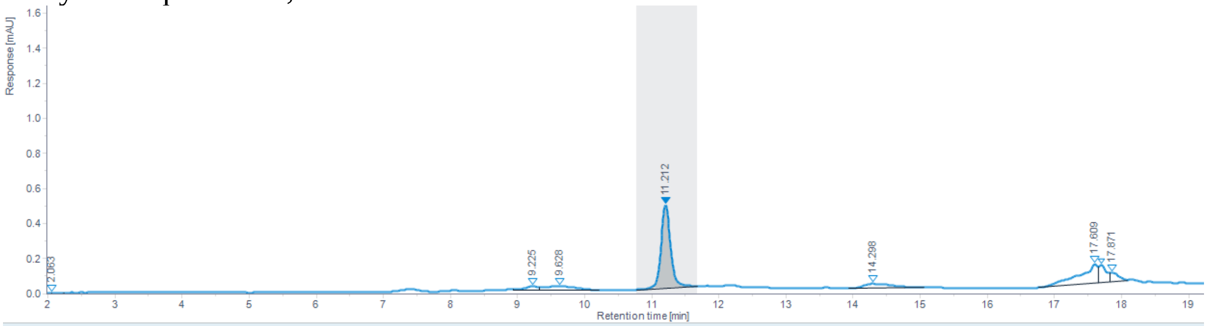

Injection Results

Peaks Summary

#	Name	Signal description	RT (min)	Area (mAU·s)	Area%	Height (mAU)	Height%	Amount	Concentration	Start time (min)	End time (min)
5		DAD1A,Sig=260,4 Ref=off	1.546	2928.419	63.757	822.790	85.52			1.481	2.062
6		DAD1A,Sig=260,4 Ref=off	2.063	16.198	0.353	2.885	0.30			2.062	2.244
7		DAD1A,Sig=260,4 Ref=off	9.225	33.187	0.723	2.480	0.26			8.949	9.330
8		DAD1A,Sig=260,4 Ref=off	9.628	75.054	1.634	2.149	0.22			9.330	10.224
9		DAD1A,Sig=260,4 Ref=off	11.212	469.988	10.233	47.360	4.92			10.777	11.695

Entry 3: 1 equiv **AM**, no additives

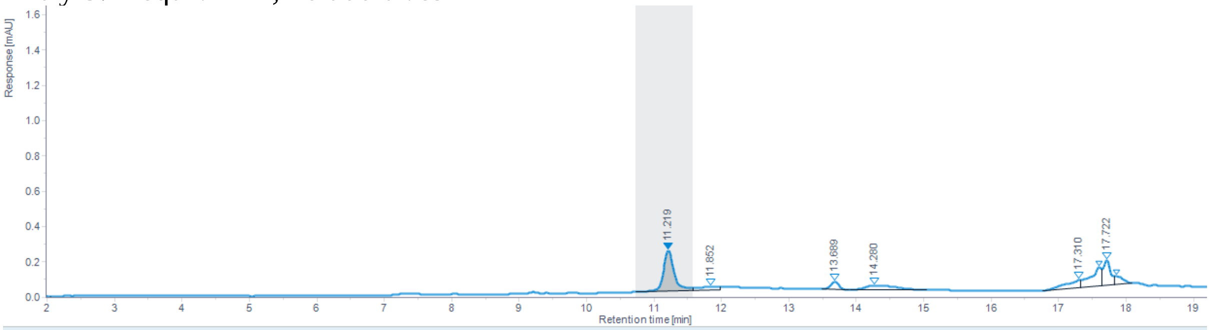

Injection Results

Peaks Summary

#	Name	Signal description	RT (min)	Area (mAU·s)	Area%	Height (mAU)	Height%	Amount	Concentration	Start time (min)	End time (min)
4		DAD1A,Sig=260,4 Ref=off	1.541	2089.094	59.883	554.264	82.03			1.474	2.061
5		DAD1A,Sig=260,4 Ref=off	11.219	259.998	7.453	22.961	3.40			10.737	11.589

Entry 4: 1 equiv **Ru-1**, 40,000 equiv NaCl

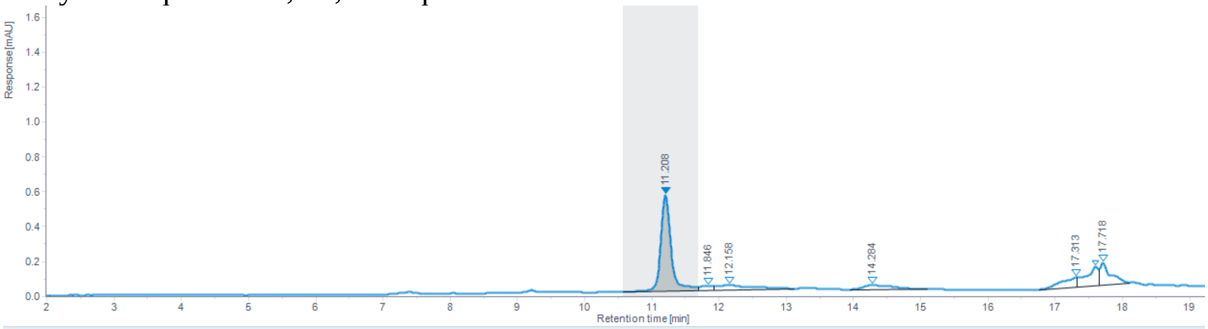

Injection Results

Peaks Summary

#	Name	Signal description	RT (min)	Area (mAU·s)	Area%	Height (mAU)	Height%	Amount	Concentration	Start time (min)	End time (min)
2		DAD1A,Sig=260,4 Ref=off	1.458	66.078	1.638	22.350	2.89			1.402	1.479
3		DAD1A,Sig=260,4 Ref=off	1.542	2274.547	56.389	635.471	82.14			1.479	2.049
4		DAD1A,Sig=260,4 Ref=off	11.208	590.338	14.635	55.119	7.13			10.580	11.703

Entry 5: 1 equiv **AM**, 40,000 NaCl

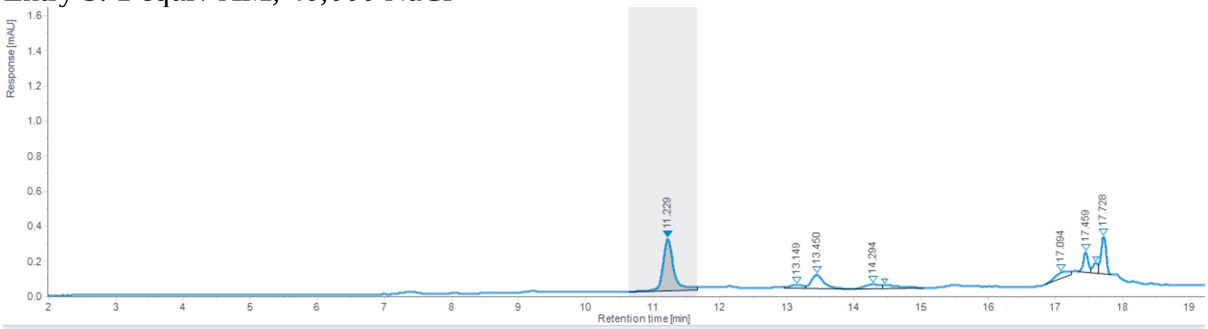

Injection Results

Peaks Summary

#	Name	Signal description	RT (min)	Area (mAU·s)	Area%	Height (mAU)	Height%	Amount	Concentration	Start time (min)	End time (min)
2		DAD1A,Sig=260,4 Ref=off	1.310	79.526	2.142	13.695	1.81			1.253	1.387
3		DAD1A,Sig=260,4 Ref=off	1.459	59.331	1.598	16.879	2.23			1.394	1.474
4		DAD1A,Sig=260,4 Ref=off	1.541	2324.907	62.623	622.418	82.32			1.474	2.119
5		DAD1A,Sig=260,4 Ref=off	11.229	324.524	8.741	29.410	3.89			10.654	11.675

Entry 6: 10 equiv **Ru-1**, no additives

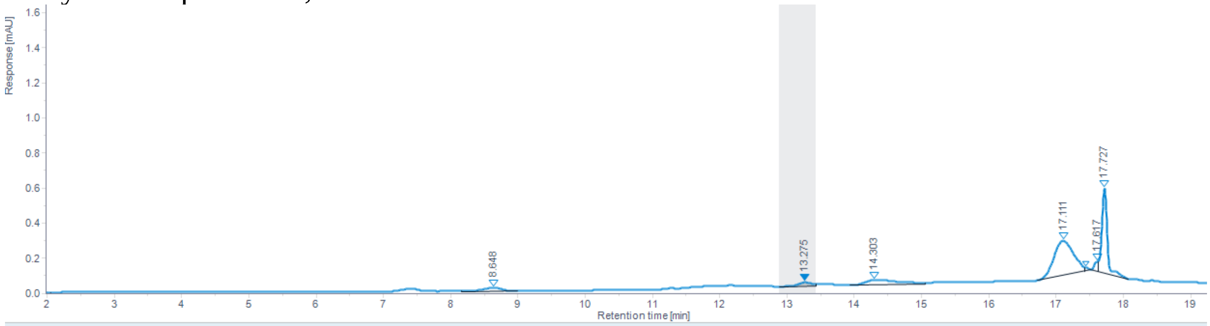

Injection Results

Peaks Summary

#	Name	Signal description	RT (min)	Area (mAU·s)	Area%	Height (mAU)	Height%	Amount	Concentration	Start time (min)	End time (min)
2		DAD1A,Sig=260,4 Ref=off	1.461	79.701	4.763	22.784	13.16			1.408	1.494
3		DAD1A,Sig=260,4 Ref=off	1.549	423.694	25.322	46.679	26.96			1.494	2.069
4		DAD1A,Sig=260,4 Ref=off	8.648	38.358	2.292	1.879	1.09			8.165	9.005
5		DAD1A,Sig=260,4 Ref=off	13.275	29.382	1.756	2.157	1.25			12.902	13.434

Entry 7: 10 equiv **AM**, no additives

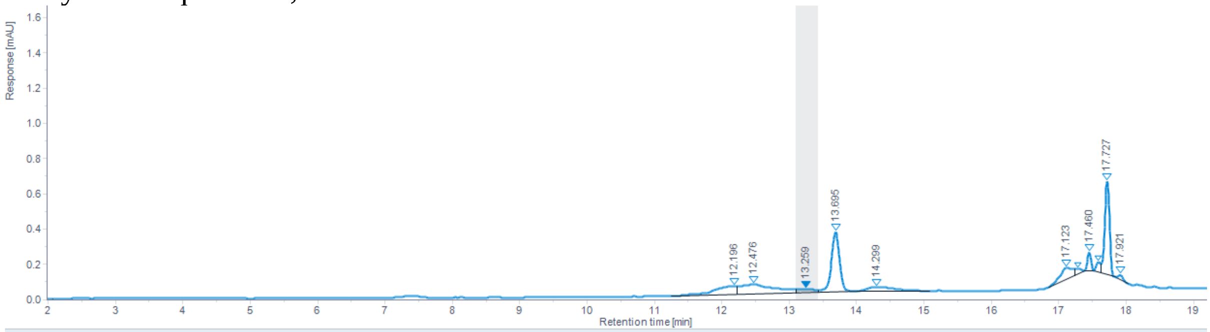

Injection Results

Peaks | Summary

#	Name	Signal description	RT (min)	Area (mAU·s)	Area%	Height (mAU)	Height%	Amount	Concentration	Start time (min)	End time (min)
2		DAD1A,Sig=260,4 Ref=off	1.315	130.572	6.065	16.653	7.49			1.224	1.400
3		DAD1A,Sig=260,4 Ref=off	1.458	82.914	3.851	22.359	10.05			1.403	1.495
4		DAD1A,Sig=260,4 Ref=off	1.548	90.964	4.225	19.047	8.56			1.495	1.580
5		DAD1A,Sig=260,4 Ref=off	1.612	248.354	11.536	17.644	7.93			1.580	2.085
6		DAD1A,Sig=260,4 Ref=off	12.196	116.315	5.403	4.817	2.17			11.260	12.234
7		DAD1A,Sig=260,4 Ref=off	12.476	190.811	8.863	5.540	2.49			12.234	13.104
8		DAD1A,Sig=260,4 Ref=off	13.259	34.564	1.605	1.908	0.86			13.104	13.446

Entry 8: 10 equiv **Ru-1**, 40,000 equiv NaCl

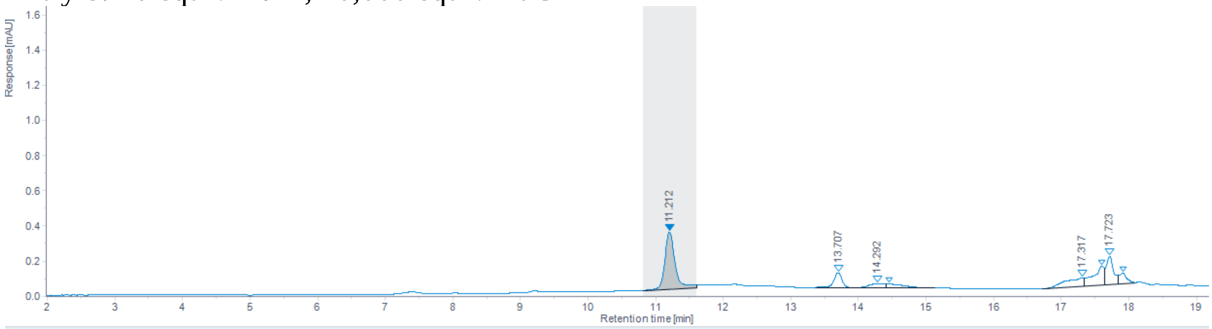

Injection Results

Peaks | Summary

#	Name	Signal description	RT (min)	Area (mAU·s)	Area%	Height (mAU)	Height%	Amount	Concentration	Start time (min)	End time (min)
2		DAD1A,Sig=260,4 Ref=off	1.459	69.974	2.169	22.583	3.92			1.404	1.483
3		DAD1A,Sig=260,4 Ref=off	1.545	1642.390	50.902	442.526	76.89			1.483	2.065
4		DAD1A,Sig=260,4 Ref=off	11.212	346.060	10.725	32.633	5.67			10.830	11.611

Entry 9: 10 equiv **AM**, 40,000 equiv NaCl

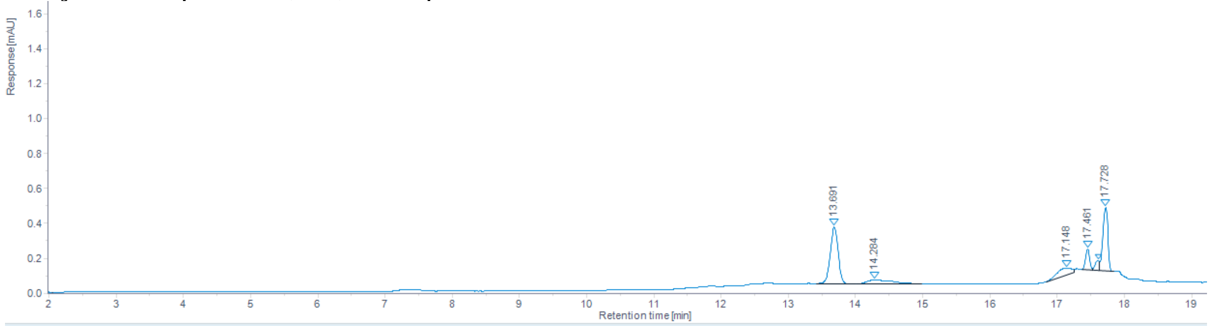

Injection Results

Peaks | Summary

#	Name	Signal description	RT (min)	Area (mAU·s)	Area%	Height (mAU)	Height%	Amount	Concentration	Start time (min)	End time (min)
2		DAD1A,Sig=260,4 Ref=off	1.310	82.729	4.475	14.104	7.16			1.252	1.390
3		DAD1A,Sig=260,4 Ref=off	1.460	68.812	3.722	16.759	8.51			1.390	1.483
4		DAD1A,Sig=260,4 Ref=off	1.543	361.141	19.535	38.576	19.59			1.483	2.068
5		DAD1A,Sig=260,4 Ref=off	13.691	265.047	14.337	32.327	16.41			13.424	14.010
6		DAD1A,Sig=260,4 Ref=off	14.284	64.538	3.491	2.259	1.15			14.011	14.986

Figure S19. RP-HPLC traces for **Table S8** (entries 1-9) of RNA backbone of 16mer RNA-FAM model during aqueous metathesis using **Ru-1** or **AM** at 25 °C.

16mer RNA-FAM (3´-UCCACGCUUAGCUCUC-5´-6-FAM), 70 °C

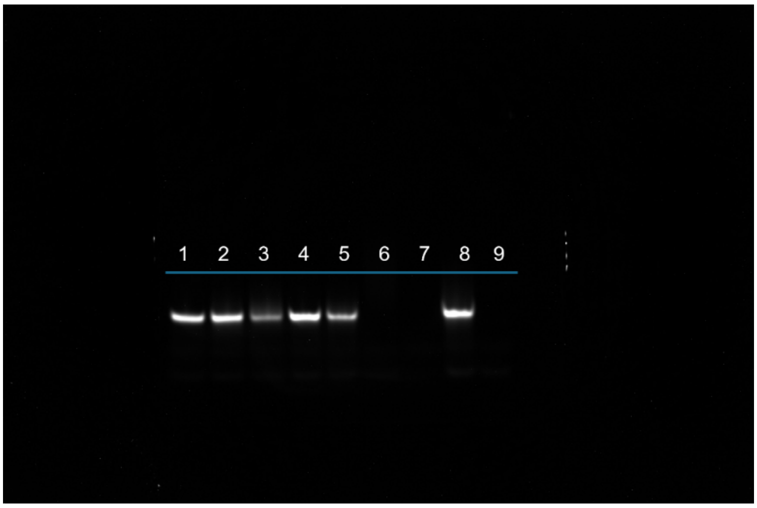

Figure S20. Backbone degradation of fluorescein-labelled 16mer native RNA by **Ru-1** and **AM** at 70 °C. Lane 1: Ru-free control, normalized to 100% for band intensity quantification (BQ). Lanes 2-3: 1 equiv **Ru-1** or **AM**; BQ 88% or 46%. Lanes 4-5: 1 equiv **Ru-1** or **AM** in 0.4 M NaCl; BQ 99% or 57%. Lanes 6-7: 10 equiv **Ru-1** or **AM**, BQ 0%. Lanes 8-9: 10 equiv **Ru-1** or **AM** in 0.4 M NaCl; BQ 85% or 0%.

Table S9: 16mer RNA-FAM backbone degradation assay comparing **Ru-1** and **AM** at 70 °C. RNA was analyzed by gel electrophoresis and HPLC. RNA degradation upon treatment with either **Ru-1** or **AM** was calculated based on product peak integration vs the non-treated control.

RP-HPLC chromatograms of 16mer native RNA backbone degradation assay at 70 °C.

Entry	Catalyst (equiv)	Additives (equiv)	DNA quantification by electrophoresis (%)	DNA quantification by HPLC analysis (Area under the curve, %)
1	None	None	100	100
2	1 **Ru-1**	None	88	86
3	1 **AM**	None	46	45
4	1 **Ru-1**	40,000 NaCl	99	97
5	1 **AM**	40,000 NaCl	57	49
6	10 **Ru-1**	None	0	0
7	10 **AM**	None	0	0
8	10 **Ru-1**	40,000 NaCl	85	60
9	10 **AM**	40,000 NaCl	0	0

Entry 1: Control

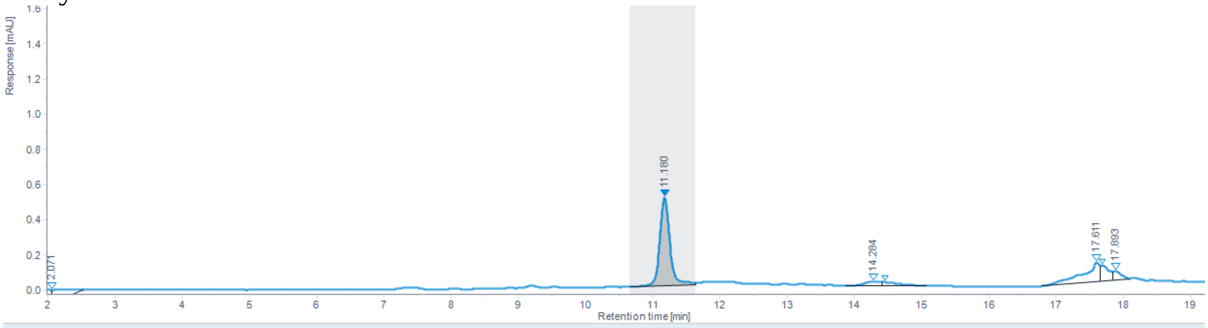

Injection Results

Peaks | Summary

#	Name	Signal description	RT (min)	Area (mAU·s)	Area%	Height (mAU)	Height%	Amount	Concentration	Start time (min)	End time (min)
2		DAD1A,Sig=260,4 Ref=off	1.458	72.646	1.754	23.665	2.94			1.404	1.481
3		DAD1A,Sig=260,4 Ref=off	1.543	2503.720	60.466	669.664	83.24			1.481	2.065
4		DAD1A,Sig=260,4 Ref=off	2.071	108.478	2.620	7.339	0.91			2.065	2.544
5		DAD1A,Sig=260,4 Ref=off	11.180	539.930	13.040	50.462	6.27			10.671	11.643

Entry 2: 1 equiv **Ru-1**, no additives

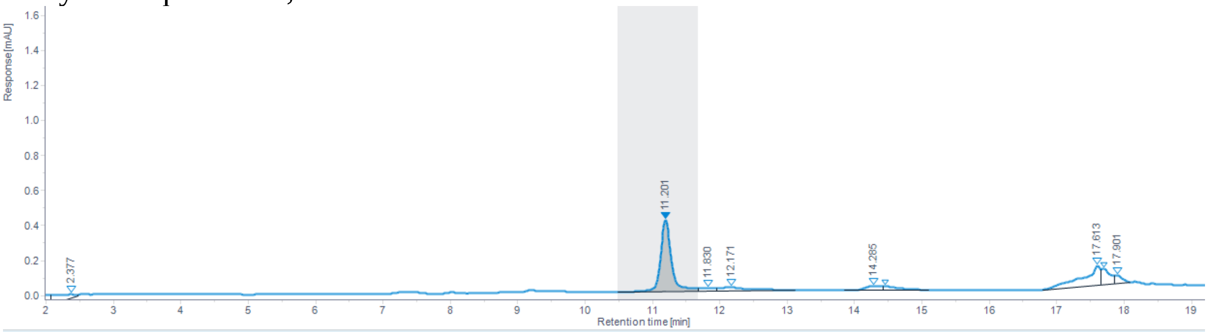

Injection Results

Peaks | Summary

#	Name	Signal description	RT (min)	Area (mAU·s)	Area%	Height (mAU)	Height%	Amount	Concentration	Start time (min)	End time (min)
2		DAD1A,Sig=260,4 Ref=off	1.310	84.956	1.876	14.429	1.62			1.252	1.390
3		DAD1A,Sig=260,4 Ref=off	1.461	62.245	1.375	17.224	1.93			1.390	1.475
4		DAD1A,Sig=260,4 Ref=off	1.542	2874.694	63.480	763.130	85.50			1.475	2.082
5		DAD1A,Sig=260,4 Ref=off	2.377	75.887	1.676	2.115	0.24			2.082	2.479
6		DAD1A,Sig=260,4 Ref=off	11.201	461.602	10.193	40.873	4.58			10.490	11.683

Entry 3: 1 equiv **AM**, no additives

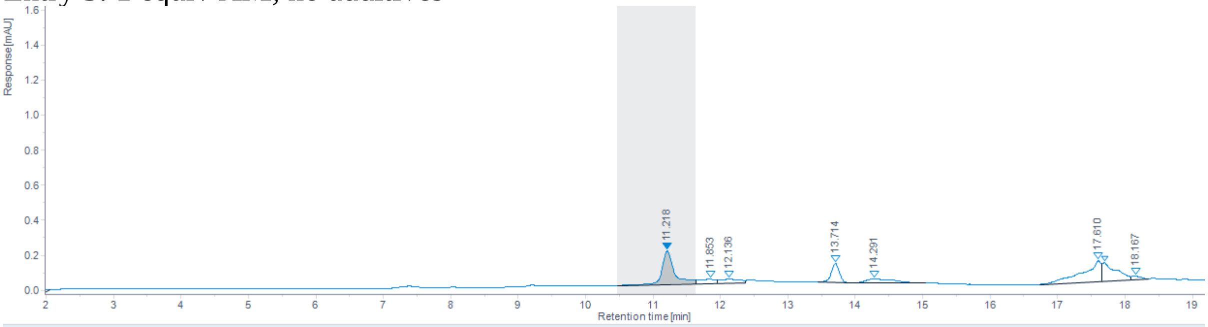

Injection Results

Peaks | Summary

#	Name	Signal description	RT (min)	Area (mAU·s)	Area%	Height (mAU)	Height%	Amount	Concentration	Start time (min)	End time (min)
2		DAD1A,Sig=260,4 Ref=off	1.186	63.252	1.802	13.212	2.04			1.134	1.252
3		DAD1A,Sig=260,4 Ref=off	1.311	90.394	2.575	15.014	2.32			1.252	1.392
4		DAD1A,Sig=260,4 Ref=off	1.461	63.926	1.821	16.874	2.61			1.395	1.479
5		DAD1A,Sig=260,4 Ref=off	1.544	1935.705	55.138	527.000	81.40			1.479	2.064
6		DAD1A,Sig=260,4 Ref=off	11.218	242.106	6.896	19.110	2.95			10.475	11.639

Entry 4: 1 equiv **Ru-1**, 40,000 equiv NaCl

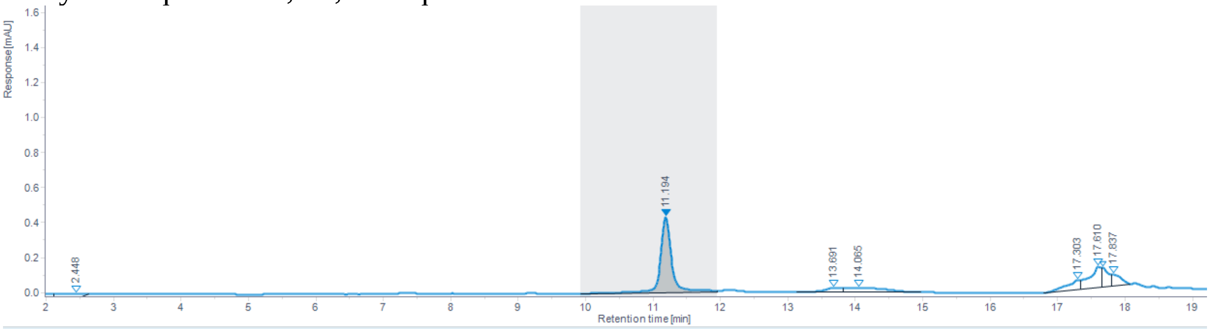

Injection Results

Peaks | Summary

#	Name	Signal description	RT (min)	Area (mAU·s)	Area%	Height (mAU)	Height%	Amount	Concentration	Start time (min)	End time (min)
2		DAD1A,Sig=260,4 Ref=off	1.455	67.441	1.786	24.872	4.21			1.399	1.469
3		DAD1A,Sig=260,4 Ref=off	1.536	2054.411	54.417	455.667	77.16			1.469	2.124
4		DAD1A,Sig=260,4 Ref=off	2.448	118.611	3.142	2.919	0.49			2.124	2.633
5		DAD1A,Sig=260,4 Ref=off	11.194	521.006	13.800	43.213	7.32			9.939	11.955

Entry 5: 1 equiv **AM**, no additives

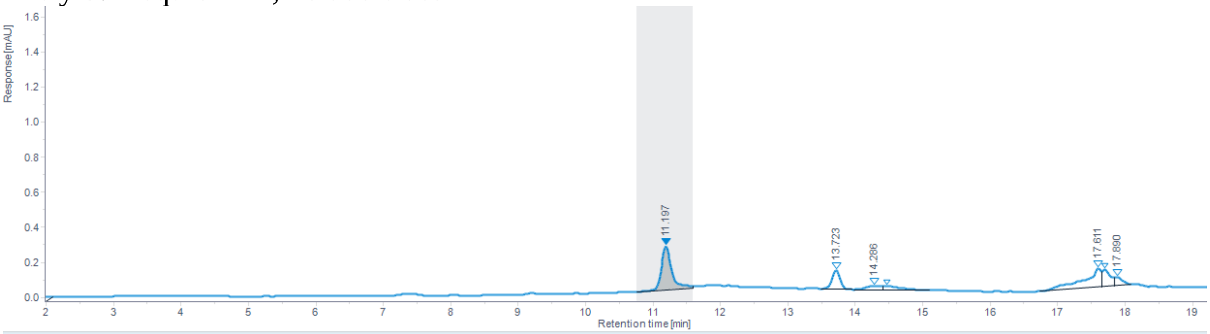

Injection Results

Peaks | Summary

#	Name	Signal description	RT (min)	Area (mAU·s)	Area%	Height (mAU)	Height%	Amount	Concentration	Start time (min)	End time (min)
2		DAD1A,Sig=260,4 Ref=off	1.316	127.800	4.061	16.107	2.77			1.213	1.402
3		DAD1A,Sig=260,4 Ref=off	1.458	69.843	2.220	22.855	3.93			1.403	1.480
4		DAD1A,Sig=260,4 Ref=off	1.542	1751.005	55.646	464.156	79.81			1.480	2.111
5		DAD1A,Sig=260,4 Ref=off	11.197	266.122	8.457	24.567	4.22			10.763	11.600

Entry 6: 10 equiv **Ru-1**, no additives

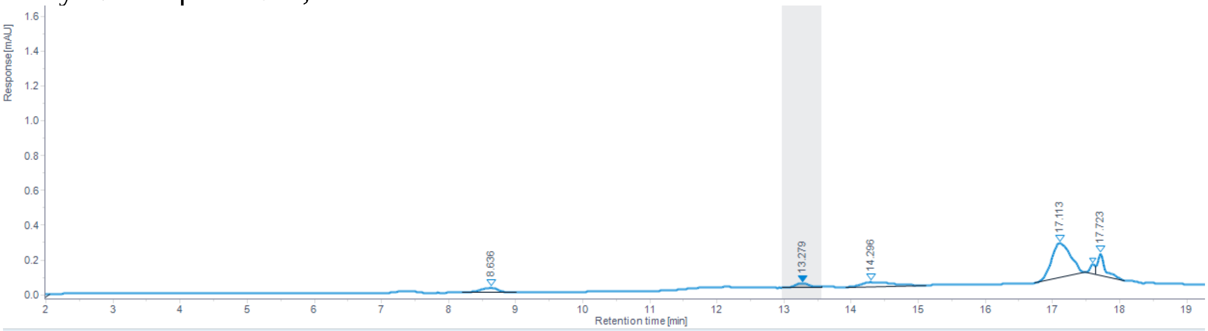

Injection Results

Peaks | Summary

#	Name	Signal description	RT (min)	Area (mAU·s)	Area%	Height (mAU)	Height%	Amount	Concentration	Start time (min)	End time (min)
1		DAD1A,Sig=260,4 Ref=off	1.316	95.891	5.463	13.619	9.22			1.201	1.401
2		DAD1A,Sig=260,4 Ref=off	1.457	76.815	4.376	22.739	15.39			1.401	1.486
3		DAD1A,Sig=260,4 Ref=off	1.541	461.179	26.272	56.063	37.94			1.486	2.066
4		DAD1A,Sig=260,4 Ref=off	8.636	52.606	2.997	2.757	1.87			8.215	9.021
5		DAD1A,Sig=260,4 Ref=off	13.279	37.305	2.125	2.505	1.70			12.981	13.572

Entry 7: 10 equiv **AM**, no additives

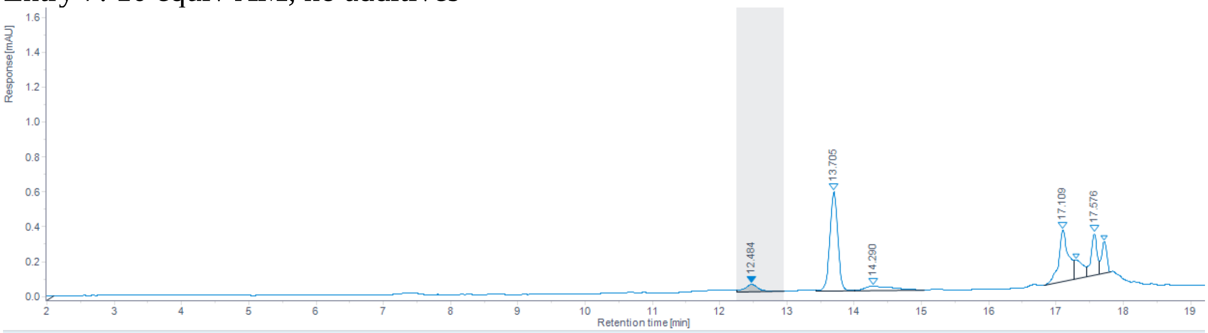

Injection Results

Peaks Summary

#	Name	Signal description	RT (min) △	Area (mAU·s)	Area%	Height (mAU)	Height%	Amount	Concentration	Start time (min)	End time (min)
1		DAD1A,Sig=260,4 Ref=off	1.024	15.554	0.670	2.423	1.02			0.966	1.078
2		DAD1A,Sig=260,4 Ref=off	1.316	209.617	9.030	17.806	7.52			1.078	1.400
3		DAD1A,Sig=260,4 Ref=off	1.458	82.305	3.546	22.566	9.53			1.406	1.494
4		DAD1A,Sig=260,4 Ref=off	1.546	103.107	4.442	21.075	8.90			1.494	1.584
5		DAD1A,Sig=260,4 Ref=off	1.611	255.702	11.015	17.971	7.59			1.584	2.103
6		DAD1A,Sig=260,4 Ref=off	12.484	64.549	2.781	4.327	1.83			12.264	12.964

Entry 8: 10 equiv **Ru-1**, 40,000 equiv NaCl

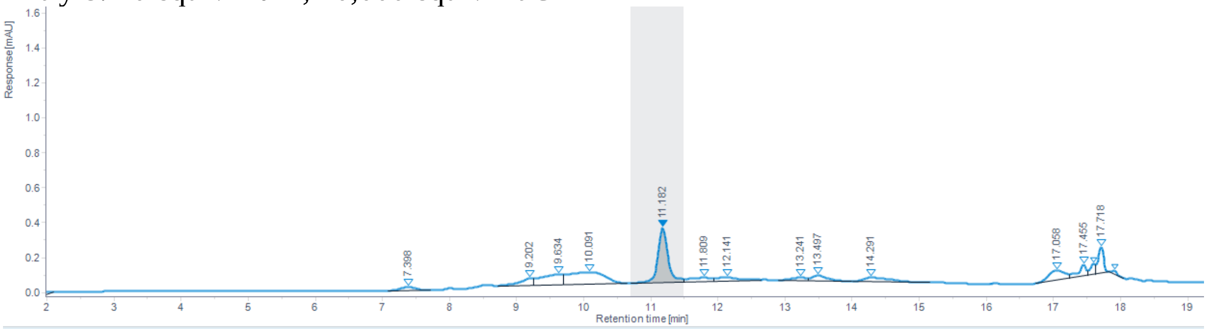

Injection Results

Peaks Summary

#	Name	Signal description	RT (min) △	Area (mAU·s)	Area%	Height (mAU)	Height%	Amount	Concentration	Start time (min)	End time (min)
11		DAD1A,Sig=260,4 Ref=off	10.091	248.389	7.084	6.703	1.12			9.712	10.651
12		DAD1A,Sig=260,4 Ref=off	11.182	326.329	9.307	30.974	5.18			10.718	11.504

Entry 9: 10 equiv **AM**, 40,000 equiv NaCl

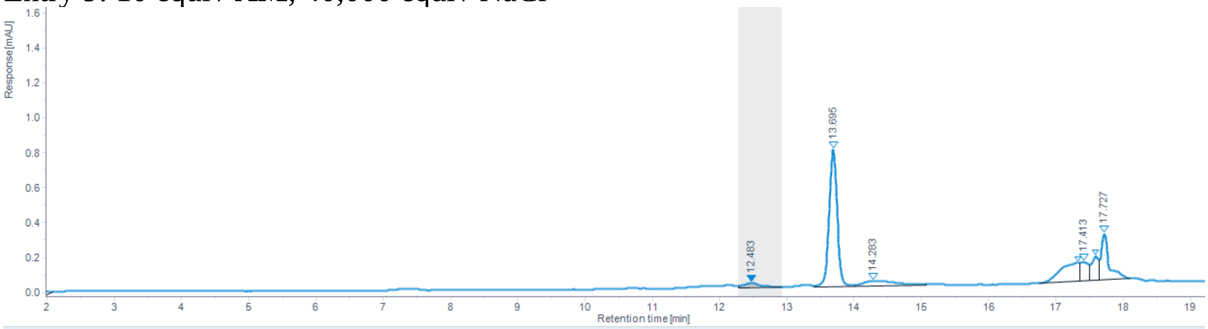

Injection Results

Peaks Summary

#	Name	Signal description	RT (min) △	Area (mAU·s)	Area%	Height (mAU)	Height%	Amount	Concentration	Start time (min)	End time (min)
4		DAD1A,Sig=260,4 Ref=off	1.546	113.848	4.813	23.245	9.83			1.486	1.588
5		DAD1A,Sig=260,4 Ref=off	1.609	201.907	8.536	15.362	6.50			1.588	2.096
6		DAD1A,Sig=260,4 Ref=off	12.483	42.433	1.794	2.613	1.11			12.290	12.931

Figure S21. RP-HPLC traces for **Table S9** (entries 1-9) of RNA backbone of 16mer RNA-FAM model during aqueous metathesis using **Ru-1** or **AM** at 70 °C.

10. Comparison of different RCM protocols

RCM reaction promoted by 150 equiv GIII in 3:2 H_2O:tBuOH

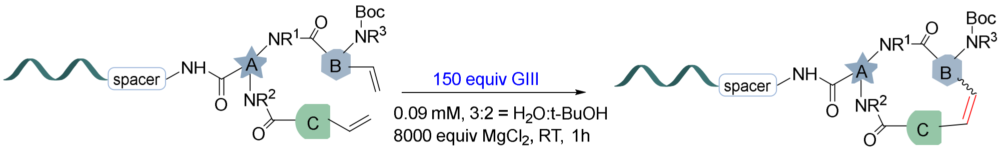

RCM reactions were performed according to the reported protocol[7]. DNA or RNA oligomers (0.09 mM) were added to 1.5 mL DNA LoBind® Eppendorf tubes, followed by the addition of 8,000 equiv $MgCl_2$, 150 equiv ruthenium complex **GIII** dissolved in tBuOH was added to the reaction mixture (3:2 H_2O:tBuOH). The tubes were sealed with Parafilm and shaken on an Eppendorf thermocycler at RT for 1 h. The reactions were quenched by adding SnatchCat (1,4-bis(3-isocyanopropyl) piperazine, 10 equiv vs Ru) and shaking at 37 °C for 30 min. The reactions were analyzed by analytical RP-HPLC and MALDI-MS after desalting by ZipTip. Replicate RCM reactions showed consistent results. Note: After addition of 8,000 equiv $MgCl_2$, the solution became biphasic. The peak eluting at 12.3 min is assigned to a **GIII**-derived side-product, as it appeared in all RP-HPLC chromatograms.

Table S10. RCM reaction promoted by 150 equiv **GIII** in 3:2 H_2O:tBuOH.

Entry	DNA/RNA	In situ yield[a] (%)	Isolated yield (%)
1	**ATGC-1**	not found	not found
2	**7DeATC-1**	not found	not found
3	**hexa-T-1**	not found	not found
4	**purine25-AUGC-1**	not found	not found
5	**2´-OMe-AUGC-1**	not found	not found

[a] In situ yields were calculated using the Agilent GC_LC area percent method.

RP-HPLC chromatograms of RCM reactions with DNA/RNA-tagged substrates.

Entry 1: Native DNA **ATGC-1**, both starting material **ATGC-1** and macrocycle **ATGC1´**were not detectable after the reaction.
(Macrocycle **ATGC1´** and **ATGC-1** should elute at 8.9 min and 11 min, respectively)

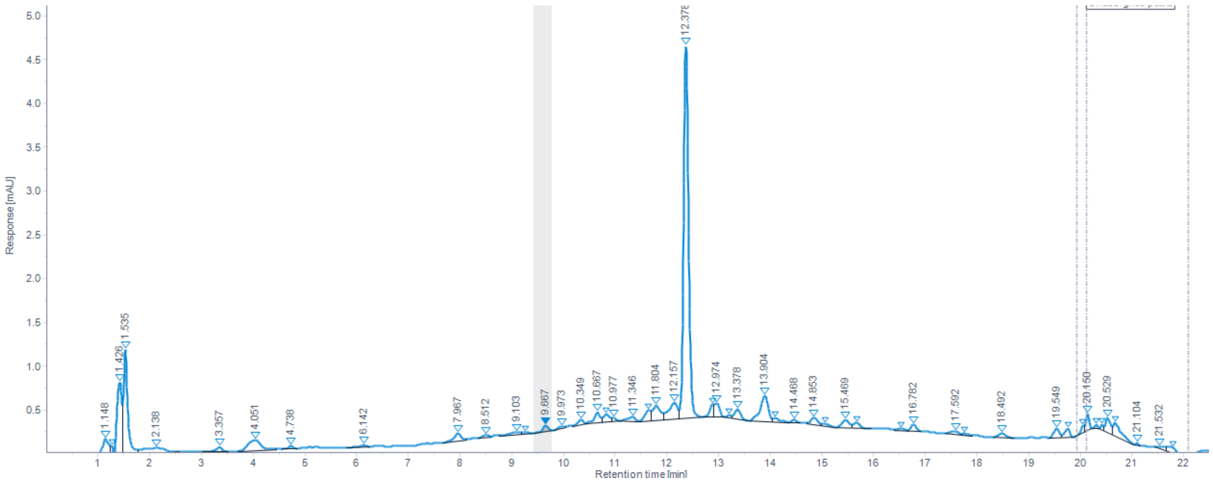

Entry 1: MALDI-MS (crude). **ATGC-1´**, Boc-on, calculated m/z 5435, **ATGC-1**, Boc-on, calculated m/z 5463.

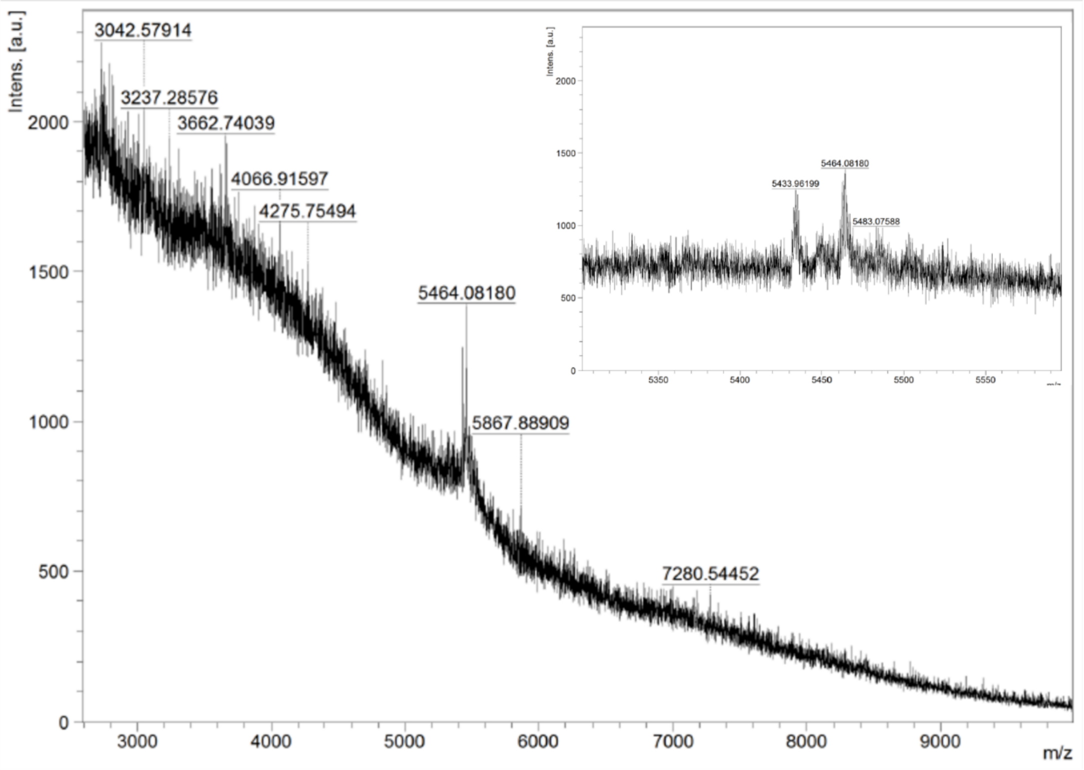

Entry 2: Chemically stabilized DNA **7DeATC-1**, both starting material **7DeATC-1** and macrocycle **7DeATC-1´** were not detectable after the reaction.
(Macrocycle **7DeATC-1´** and **7DeATC-1** should elute at 8.9 min and 10-11 min, respectively)

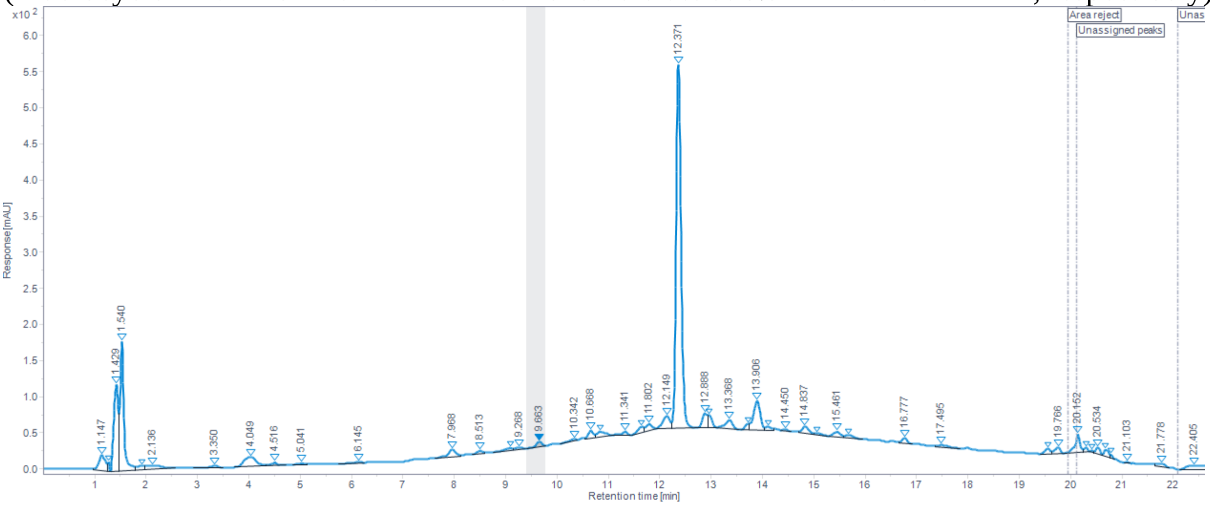

Entry 2: MALDI-MS (crude). **7DeATC-1´**, Boc-on, calculated m/z 5353, **7DeATC-1**, Boc-on, calculated m/z 5381.

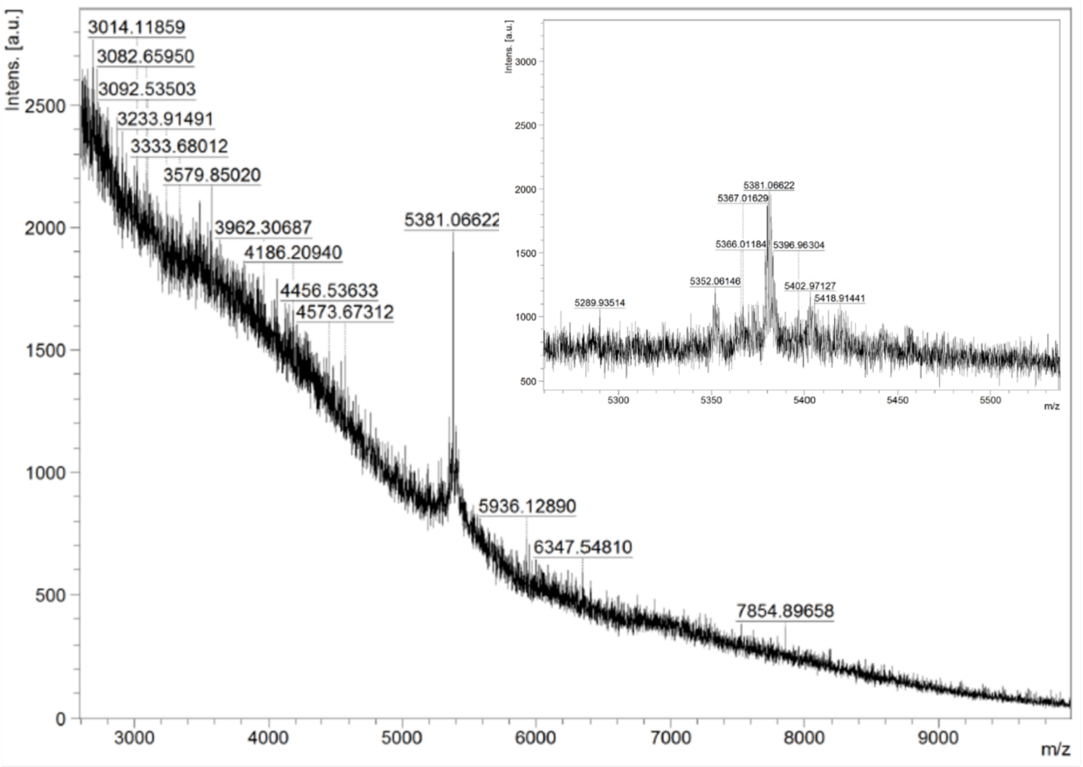

Entry 3: hexathymidine DNA **hexa-T-1**, both starting material **hexa-T-1** and macrocycle **hexa-T-1´** were not detectable after the reaction.
(Macrocycle **hexa-T-1´** and **hexa-T-1** should elute at 10 min and 12 min, respectively).

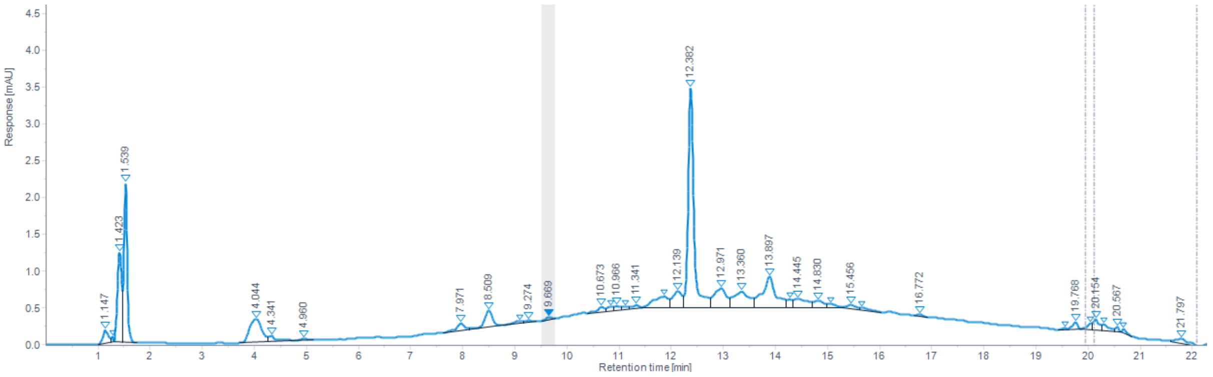

Entry 3: MALDI-MS (crude): **hexa-T-1´**, Boc-on, calculated m/z 2335, **hexa-T-1**, Boc-on, calculated m/z 2363.

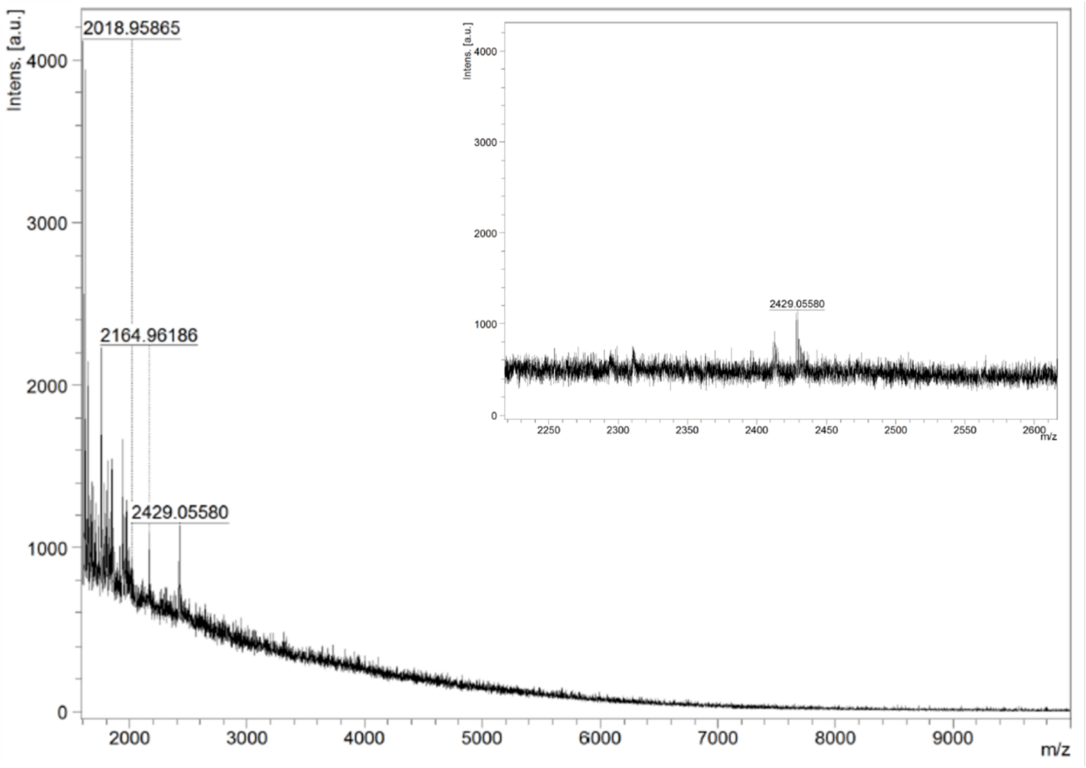

Entry 4: Native RNA **purine25-AUGC-1**, both starting material **purine25-AUGC-1** and macrocycle **purine25-AUGC-1´** were not detectable after the reaction.
(Macrocycle **purine25-AUGC-1´** and **purine25-AUGC-1** should elute at 8.8 min and 11.1 min, respectively)

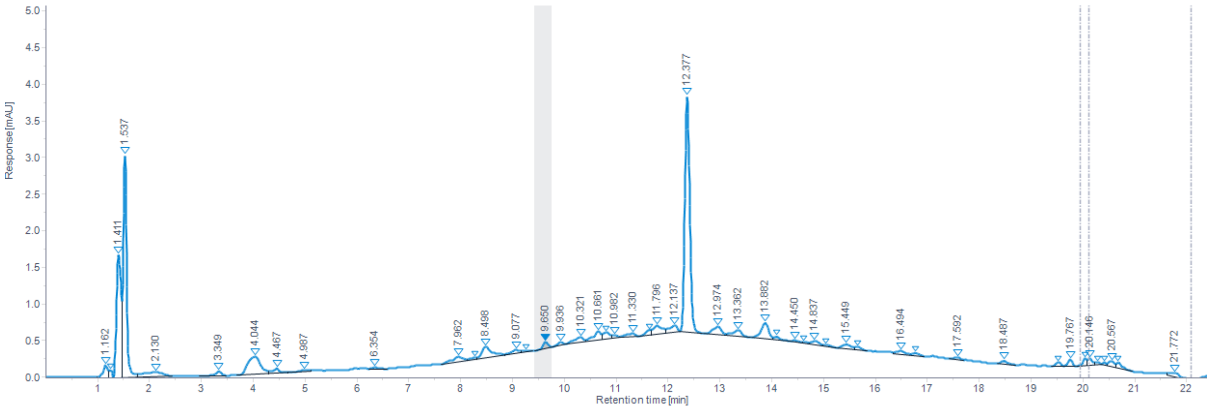

Entry 4: MALDI-MS (crude): **purine25-AUGC-1´**, Boc-on, calculated m/z 5541, **purine25-AUGC-1**, Boc-on, calculated m/z 5569.

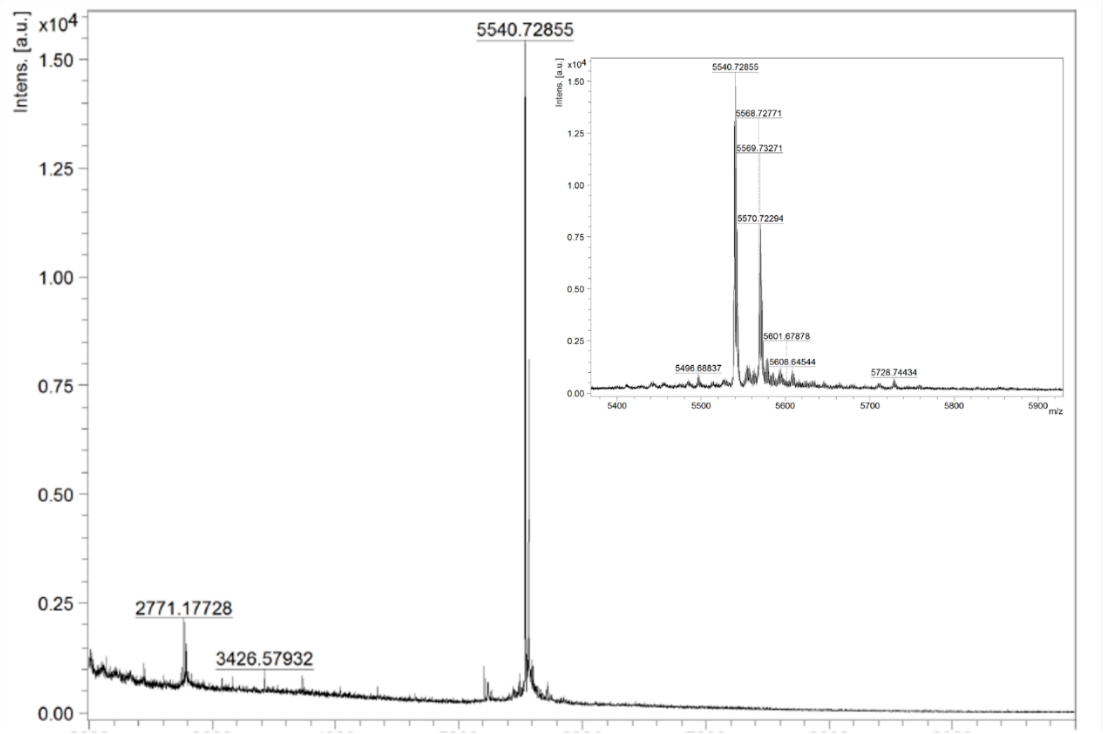

Entry 5: 2´-OMe RNA **2´-OMe-AUGC-1**, both starting material **2´-OMe-AUGC-1** and macrocycle **2´-OMe-AUGC-1´** were not detectable after the reaction.
(Macrocycle **2´-OMe-AUGC-1 ´** and **2´-OMe-AUGC-1** should elute at 9.5 min and 11.5 min, respectively)

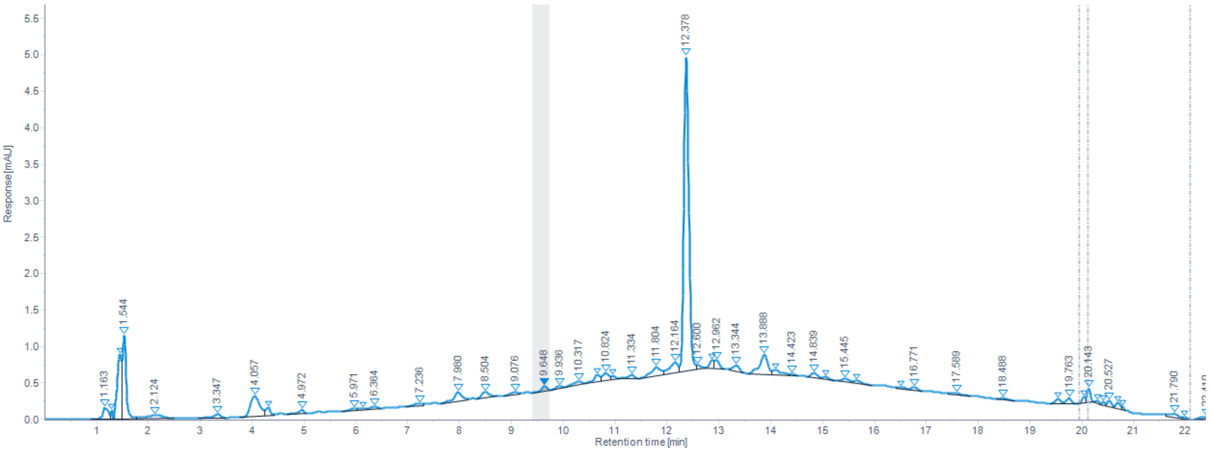

Entry 5: MALDI-MS (crude): **2´-OMe AUGC-1´**, Boc-on, calculated m/z 5766, **2´-OMe AUGC-1**, Boc-on, calculated m/z 5794.

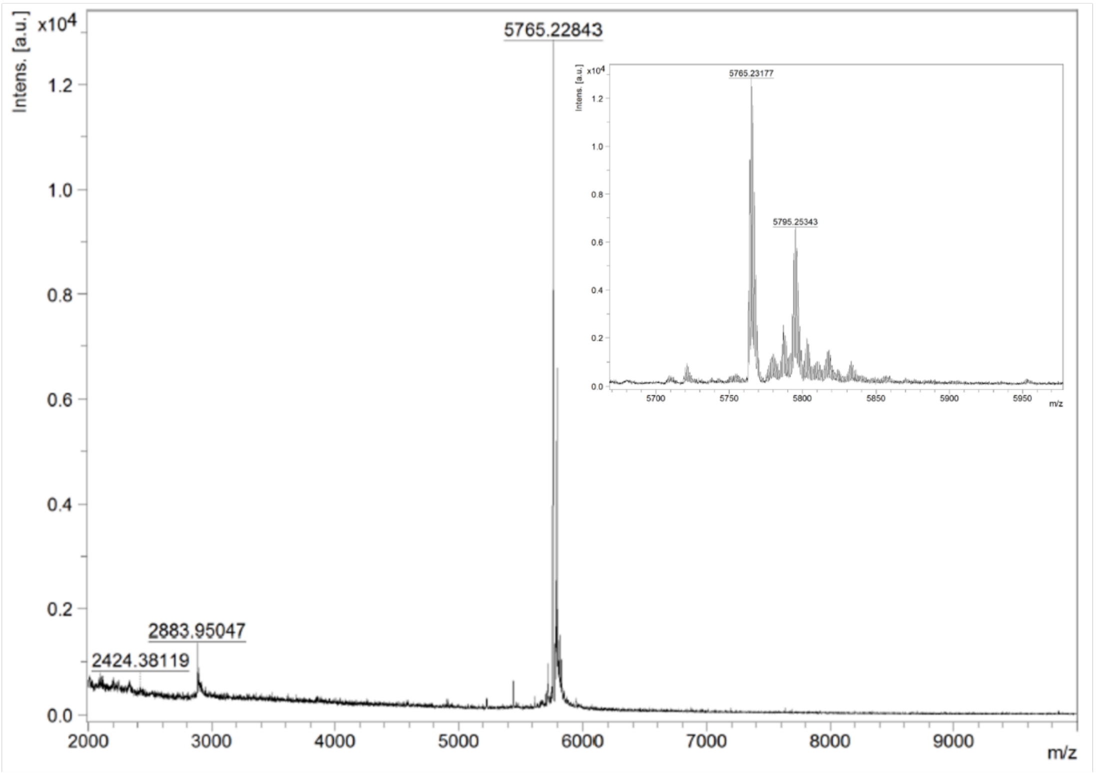

Figure S22. RP-HPLC traces and MALDI-MS analysis for **Table S10** (entries 1-5) of RCM reaction promoted by 150 equiv **GIII** ruthenium catalyst in H_2O:tBuOH, 3:2.

RCM reaction promoted by 10 equiv DA catalyst in 5:4:1 H_2O:EtOH:MeOAc

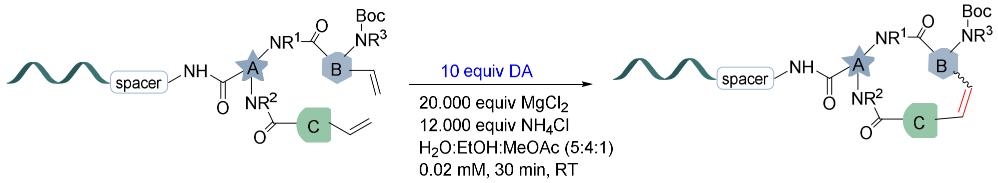

RCM reactions were performed according to the reported protocol[8]. Thus, 1 nmol DNA or RNA oligomers (0.02 mM) were added to 1.5 mL DNA LoBind® Eppendorf tubes, followed by 10 µL 2 M $MgCl_2$ and 3 µL 4 M NH_4Cl, then 20 µL EtOH and 5 µL of a 0.8 mg/mL solution of **DA** ruthenium catalyst in MeOAc, for a final volume of 50 µL (H_2O:EtOH:MeOAc 5:4:1, 25 µL:20 µL:5 µL). The reactions were sealed with Parafilm and shaken on a vortexer (Vortex-Genie® 2 mixer) at RT for 30 min. Before quenching, 1,000 equiv pH 5.3 piperazine buffer (vs DNA/RNA) was added to the solutions. The reaction mixtures were then quenched by adding sodium diethyldithiocarbamate; 400 equiv vs Ru) and shaking at 45 °C for 15 min. The reactions were analyzed by analytical RP-HPLC and MALDI-MS after desalting by ZipTip. The RCM reactions were repeated twice, and the last repeat were purified by semi-preparative RP-HPLC to determine oligonucleotide recoveries. The results were consistent. Note: after adding EtOH, the solution became slightly cloudy. The solution was shaken on thermocycler for 30 min according to the suggested protocol, and centrifuged at 15,800 × g for 10 min. A pellet was observed at the bottom of the Eppendorf tubes (**Fig. S23**). Note: peaks eluting at 11.2

min and 12.2 min are assigned as **DA** catalyst-derived side-products, as they appeared in all analytical RP-HPLC chromatograms.

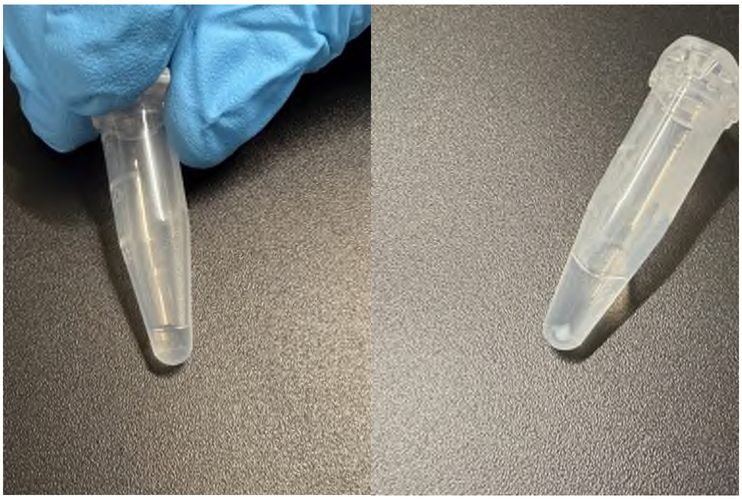

Figure S23. DNA precipitation on 1 nmol **ATGC-1** DNA (left) and 5 nmol **ATGC-1** DNA (right) after addition of EtOH, 30 min shaking and 10 min centrifugation at 15,800 × g.

Table S11. RCM reaction promoted by 10 equiv **DA** catalyst in 5:4:1 H_2O:EtOH:MeOAc. In situ yields were calculated using Agilent GC_LC area percent method.

Entry	DNA/RNA	In situ yield (%)	Isolated yield (%)
1	**ATGC-1**	38	10
2	**7DeATC-1**	60	12
3	**hexa-T-1**	95	32
4	**purine25-AUGC-1**	Not found	Not found
5	**2´-OMe-AUGC-1**	Not found	Not found

RP-HPLC chromatograms of RCM reactions with DNA/RNA-tagged substrates.
Entry 1: Native DNA **ATGC-1**. 8.9 min: **ATGC-1´**, Boc-on, 10.3 min: **ATGC-1**, Boc-on. Peaks eluting at 11.2 min and 12.2 min are side products from the **DA** catalyst complex.

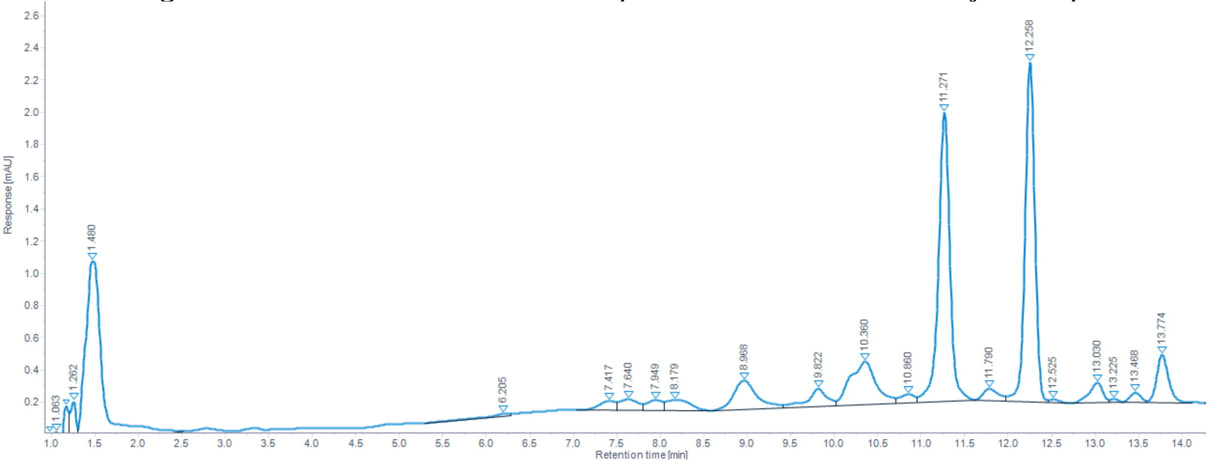

Entry 1: MALDI-MS (crude). **ATGC-1´**, Boc-on, calculated m/z 5435, **ATGC-1**, Boc-on, calculated m/z 5463.

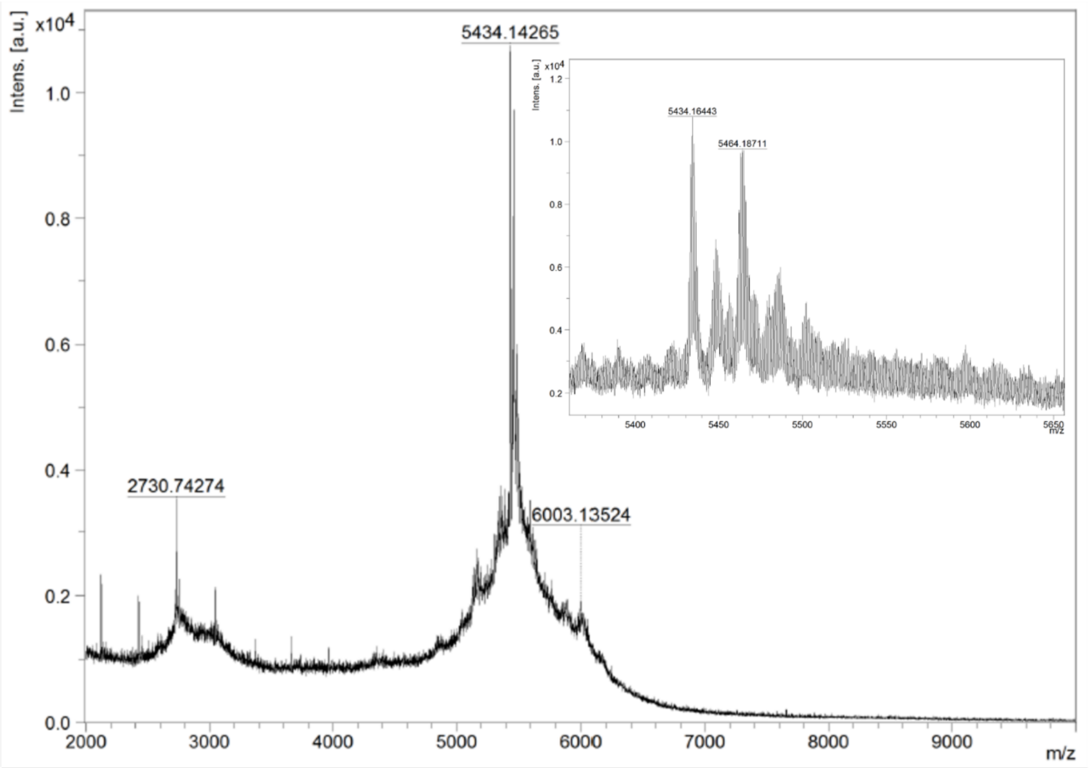

Entry 2: Chemically stabilized DNA **7DeATC-1**. 8.9 min: **7DeATC-1´**, Boc-on. 10.8 min: **7DeATC-1**, Boc-on. Peaks eluting at 11.2 min and 12.2 min are side products from the **DA** catalyst complex.

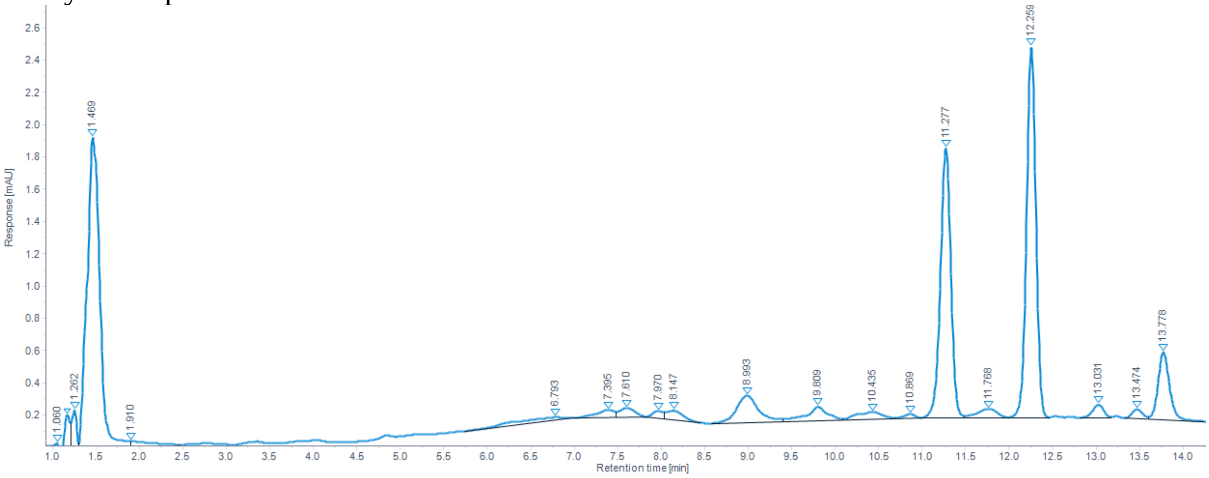

Entry 2: MALDI-MS (crude). **7DeATC-1´**, Boc-on, calculated m/z 5353, **7DeATC-1**, Boc-on, calculated m/z 5381.

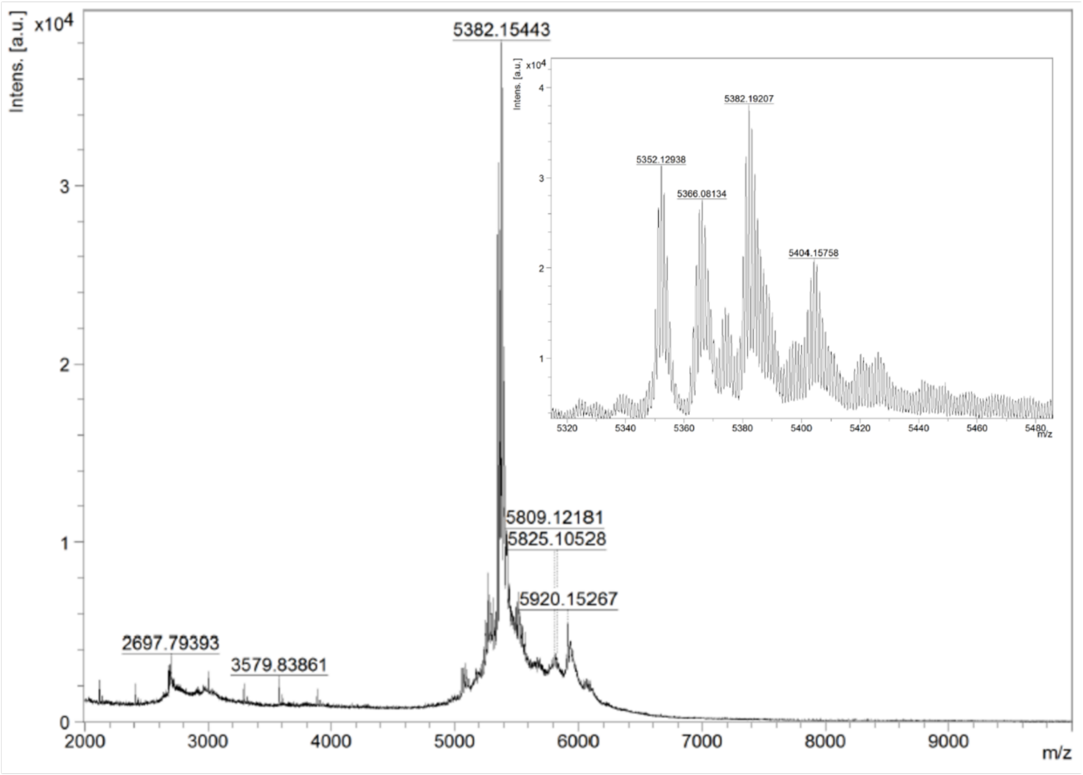

Entry 3: hexathymidine DNA **hexa-T-1**, 10.6 min: **hexa-T-1´**, Boc-on. Peaks eluting at 11.2 min and 12.2 min are side products from the **DA** catalyst complex.

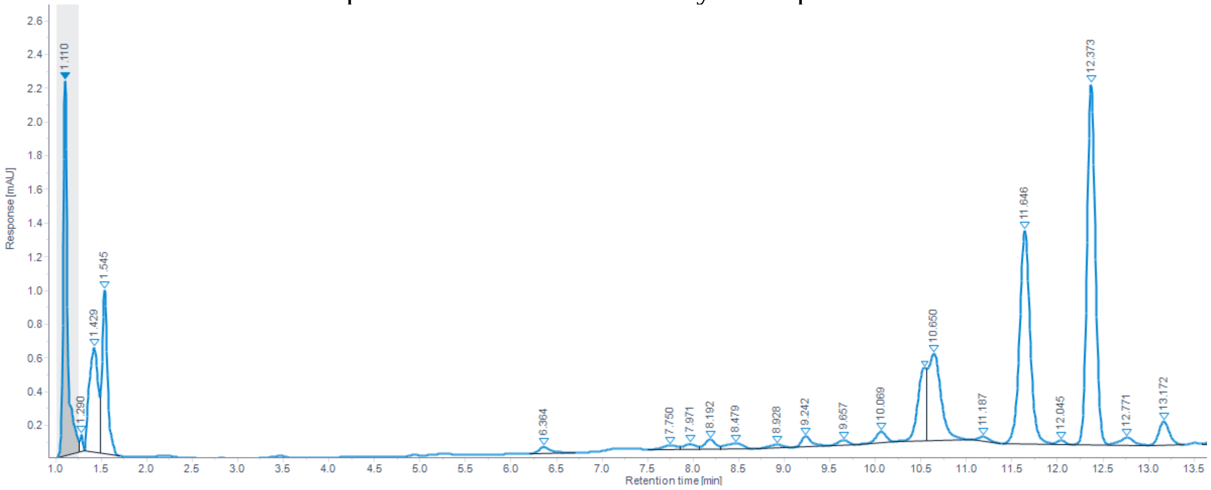

Entry 3: MALDI-MS (crude): **hexa-T-1´**, Boc-on, calculated m/z 2335, **hexa-T-1**, Boc-on, calculated m/z 2363.

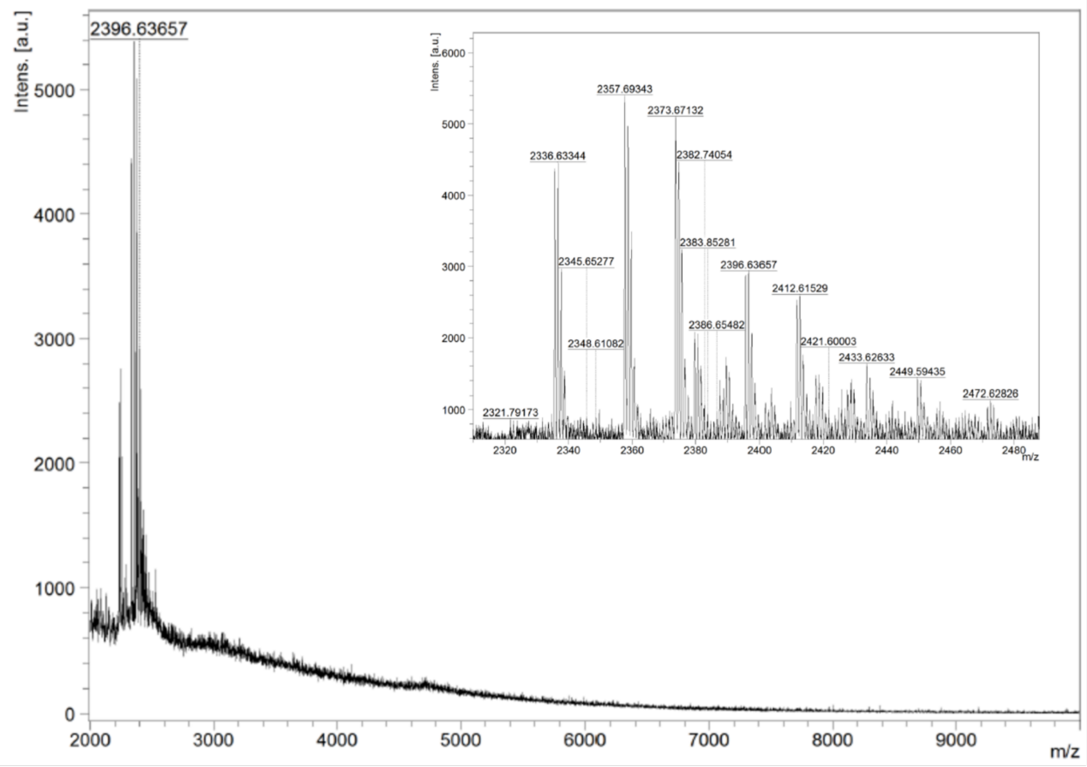

Entry 4: Native RNA **purine25-AUGC-1**, both starting material for **purine25-AUGC-1** and macrocycle **purine25-AUGC-1´** were not detectable after the reaction.
(Macrocycle **purine25-AUGC-1´** and **purine25-AUGC-1** should elute at 8.8 min and 11.1 min, respectively)

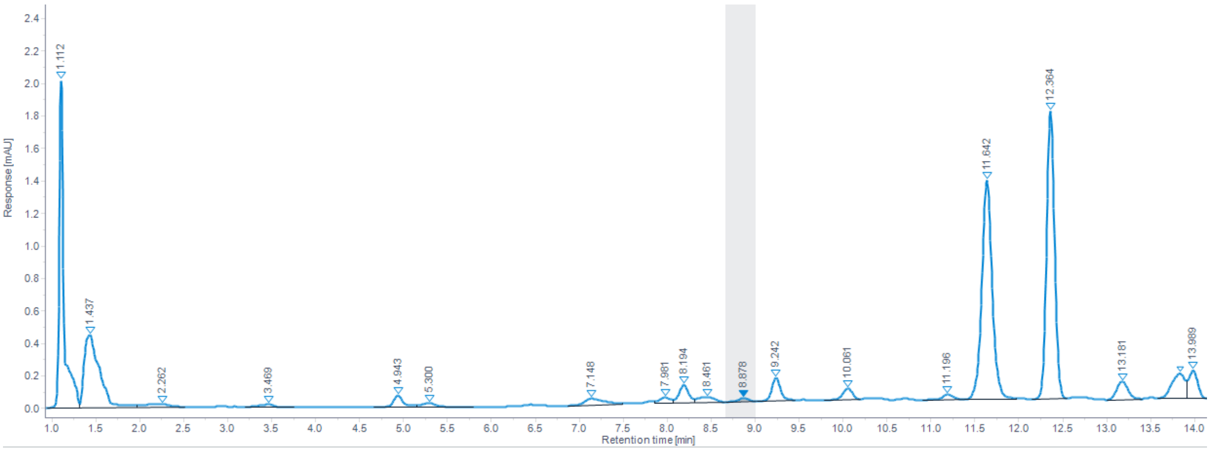

Entry 4: MALDI-MS (crude): **purine25-AUGC-1´**, Boc-on, calculated m/z 5541, **purine25-AUGC-1**, Boc-on, calculated m/z 5569.

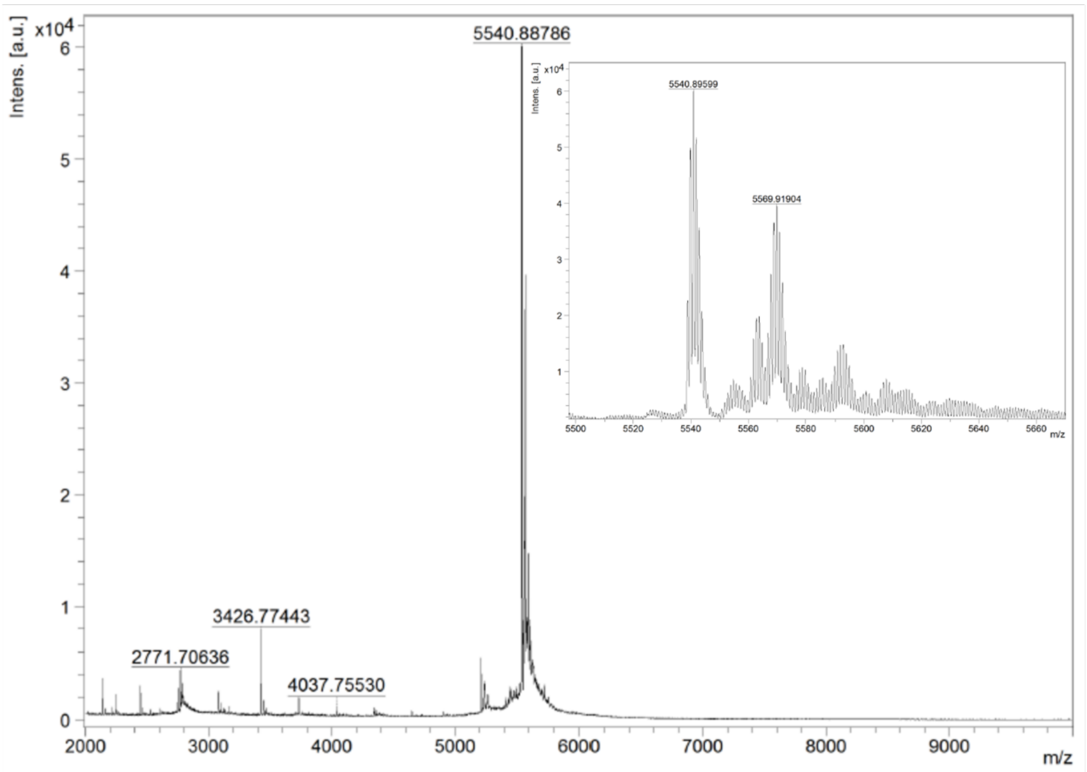

Entry 5: 2´-OMe RNA **2´-OMe-AUGC-1**, both starting material for **2´-OMe-AUGC-1** and macrocycle **2´-OMe-AUGC-1´** were not detectable after the reaction.
(Macrocycle **2´-OMe-AUGC-1 ´** and **2´-OMe-AUGC-1** should elute at 9.5 min and 11.5 min, respectively)

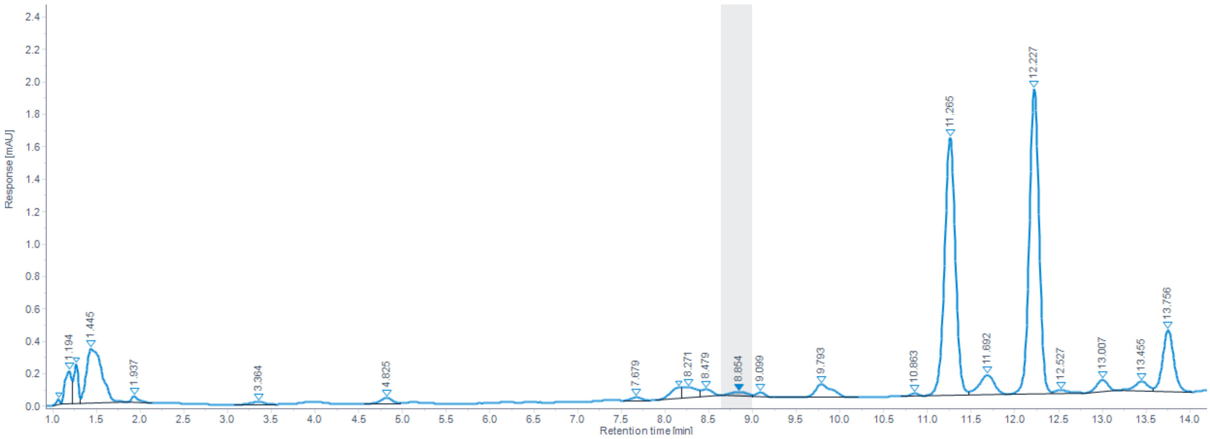

Entry 5: MALDI-MS (crude): **2´-OMe AUGC-1´**, Boc-on, calculated m/z 5766, **2´-OMe AUGC-1**, Boc-on, calculated m/z 5794.

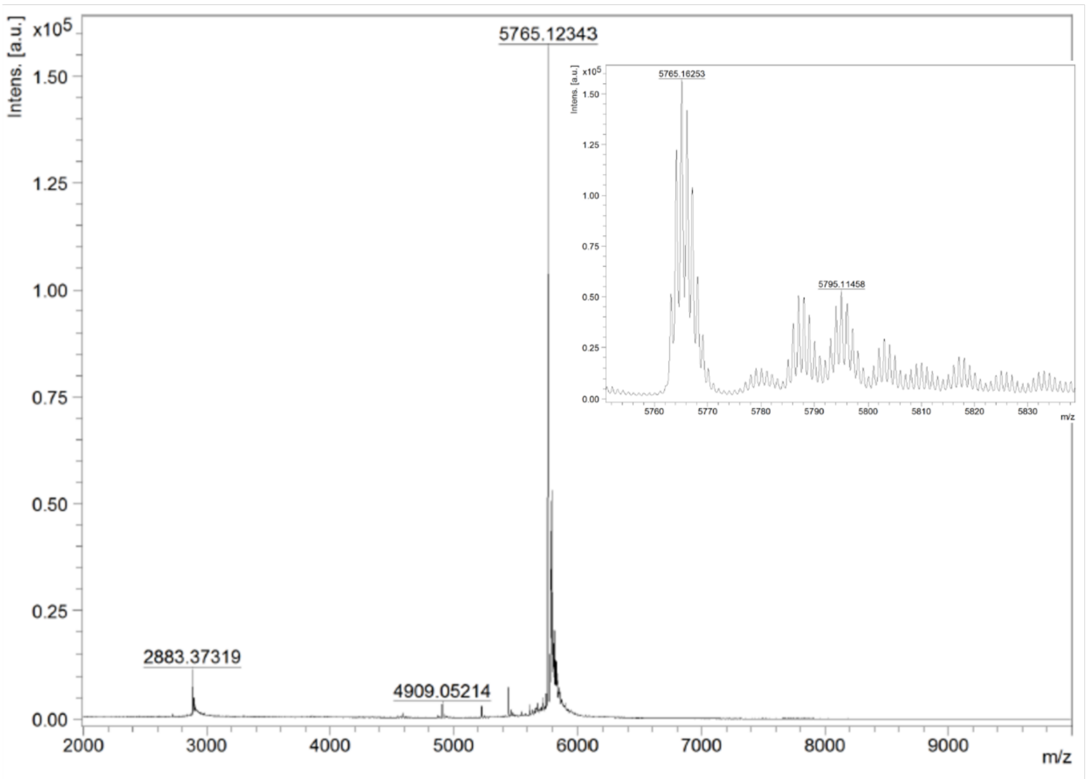

Figure S24. RP-HPLC traces and MALDI-MS analysis for **Table S11** (entries 1-5) of RCM reaction promoted by 10 equiv **DA** ruthenium catalyst in H_2O:EtOH:MeOAc, 5:4:1.

11. Optimizing conditions for RCM of hexa-T-tagged substrates (hexa-T-1) in H_2O

RCM reactions were performed as described in General Procedure 2.

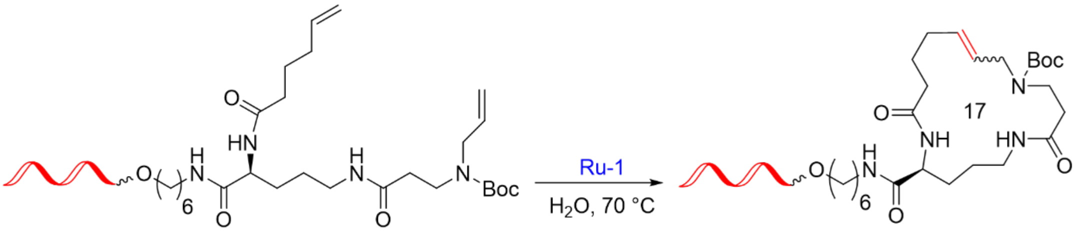

Table S12: Optimization of **hexa-T-1** RCM reaction conditions in H_2O.

**Entry**	**equiv Ru**	**Additive (equiv)**	**pH**	**Time (min)**	**In situ yield (%)**	**isolated yield (%)**	**DNA recovery (total DNA %)**
1	5	20,000 $MgCl_2$	2	30	44	20	49
2	5	20,000 $MgCl_2$	2	120	>95	13	13
3	5	40,000 N^nBu_4Cl	2	120	52	32	63
4	5	40,000 NaCl	2	120	>95	33	33
5	10	20,000 $MgCl_2$	5	30	68	21	31
6	10	40,000 NaCl	5	30	20	16	80
7	10	40,000 $NHEt_3Cl$	2	60	20	15	75
8	10	40,000 NaCl<br>40,000 $NHEt_3Cl$	2	60	27	19	71
**9**	**10**	**80,000 NaCl**<br>**80,000 $NHEt_3Cl$**	**5**	**30**	**61**	**53**	**87**
10	10	80,000 NaCl<br>80,000 $NHEt_3Cl$<br>Phosphate buffer	5	30	6	6	>95
11	10	80,000 NaCl<br>80,000 $NHEt_3Cl$<br>HEPES buffer	5	30	7	7	88
12	10	80,000 NaCl<br>80,000 $NHEt_3Cl$<br>MES buffer	5	30	7	7	93

RP-HPLC chromatograms of RCM reactions with hexa-T-tagged substrates (hexa-T-1) in H_2O.

Entry 1: 6.3 min: **hexa-T-1´**, Boc-off, 8.1 min: **hexa-T-1**, Boc-off.

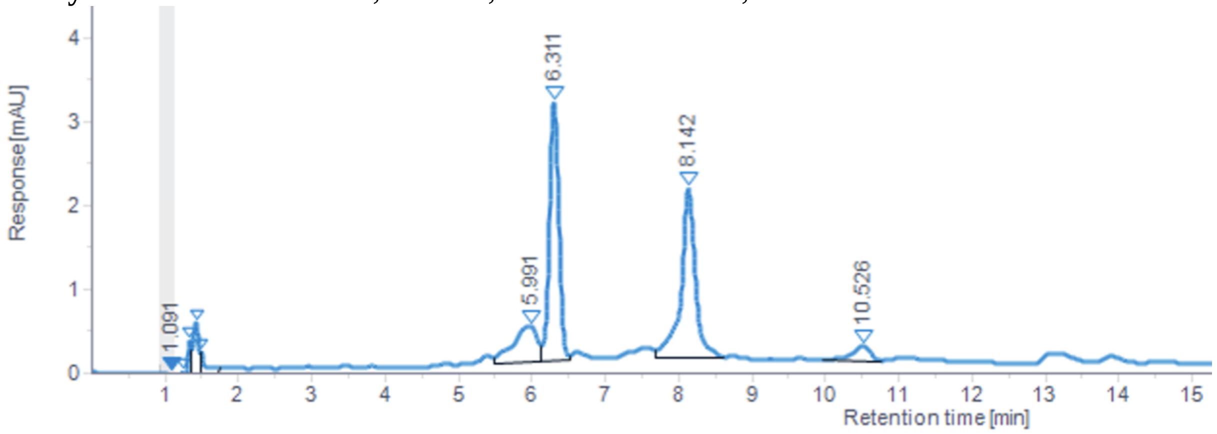

Entry 2: 6.3 min: **hexa-T-1´**, Boc-off.

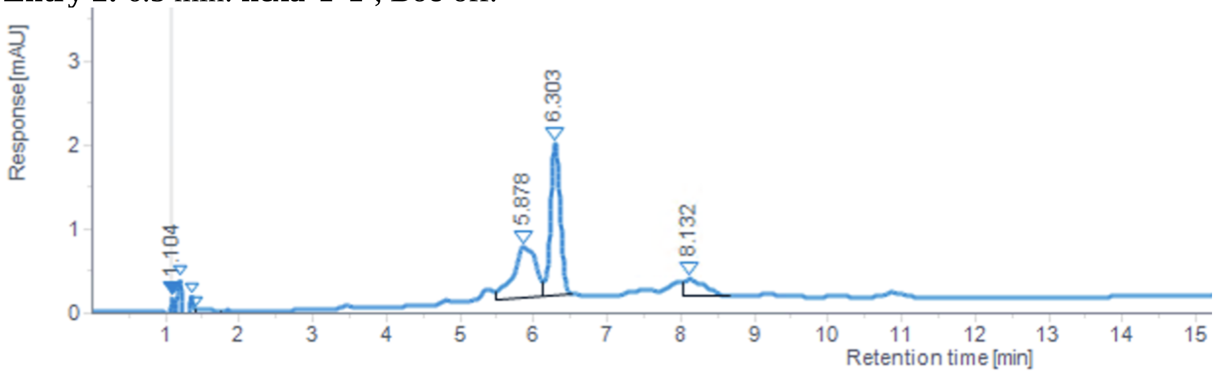

Entry 3: 7.4 min: **hexa-T-1´**, Boc-off, 8.5 min: **hexa-T-1**, Boc-off. N^nBu_4Cl shifted the retention time in RP-HPLC.

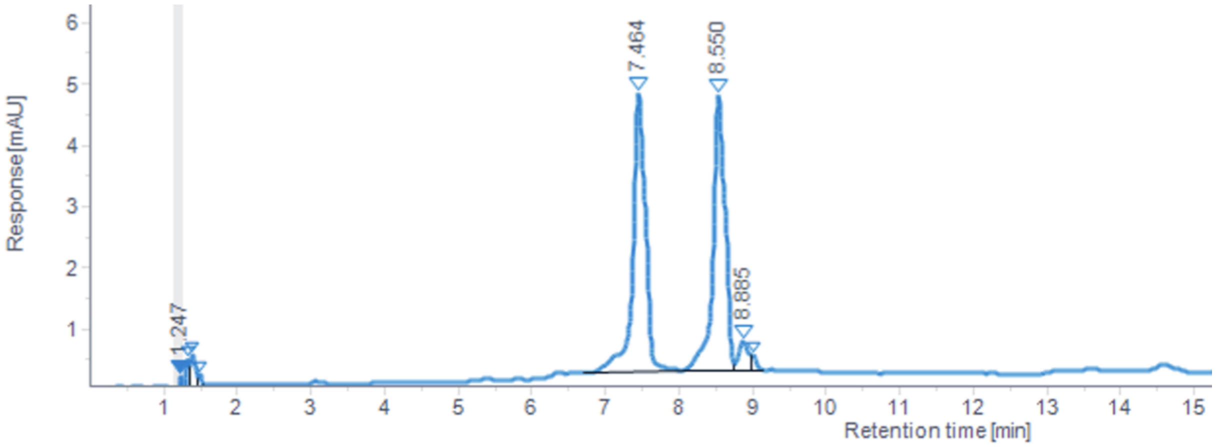

Entry 4: 6.3 min: **hexa-T-1´**, Boc-off, 8.1 min: **hexa-T-1**, Boc-off.

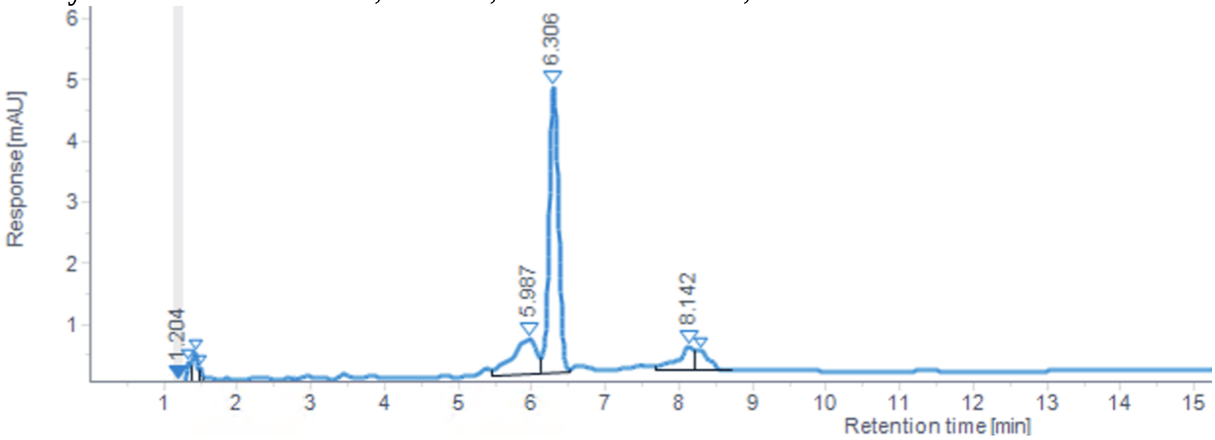

Entry 5: 10.4 min: **hexa-T-1´**, Boc-on, 12.5 min: **hexa-T-1**, Boc-on.

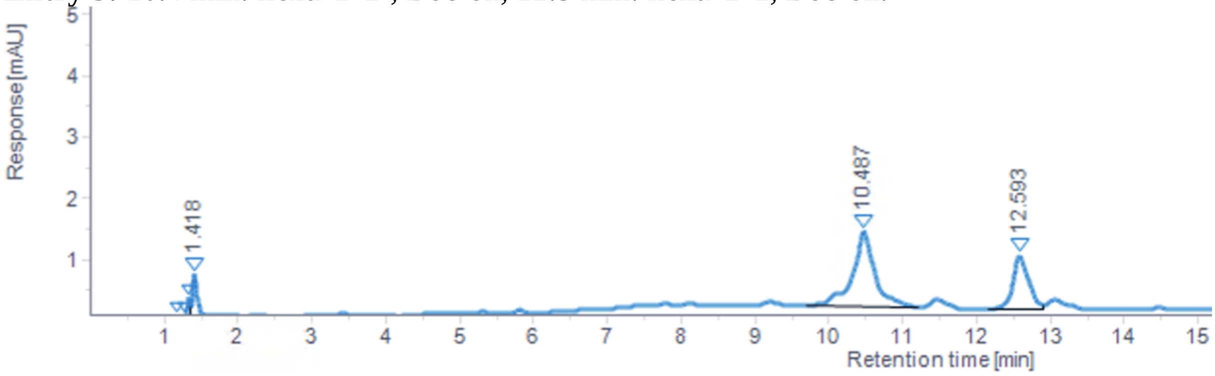

Entry 6: 10.4 min: **hexa-T-1´**, Boc-on, 12.5 min: **hexa-T-1**, Boc-on.

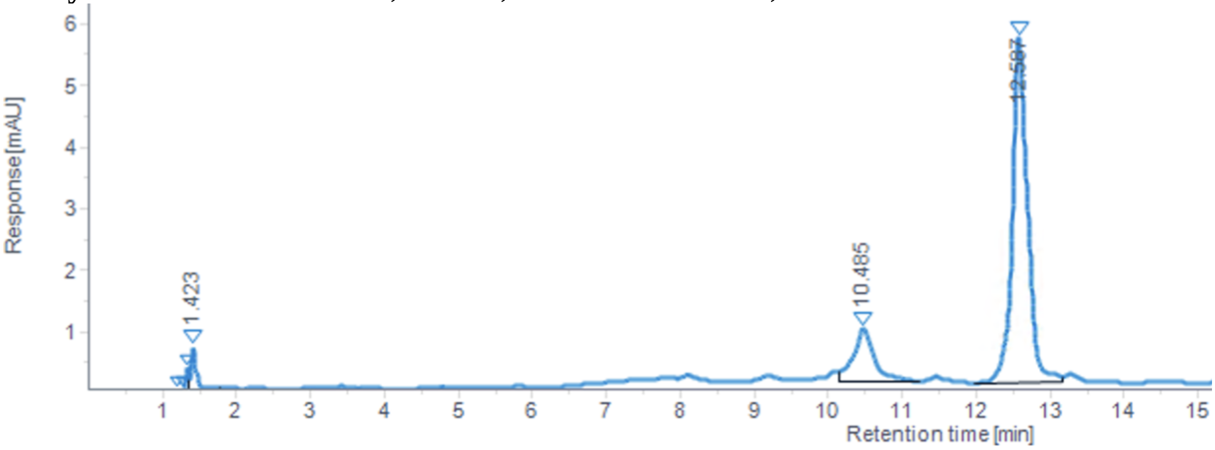

Entry 7: 6.2 min: **hexa-T-1´**, Boc-off, 8.1 min: **hexa-T-1**, Boc-off.

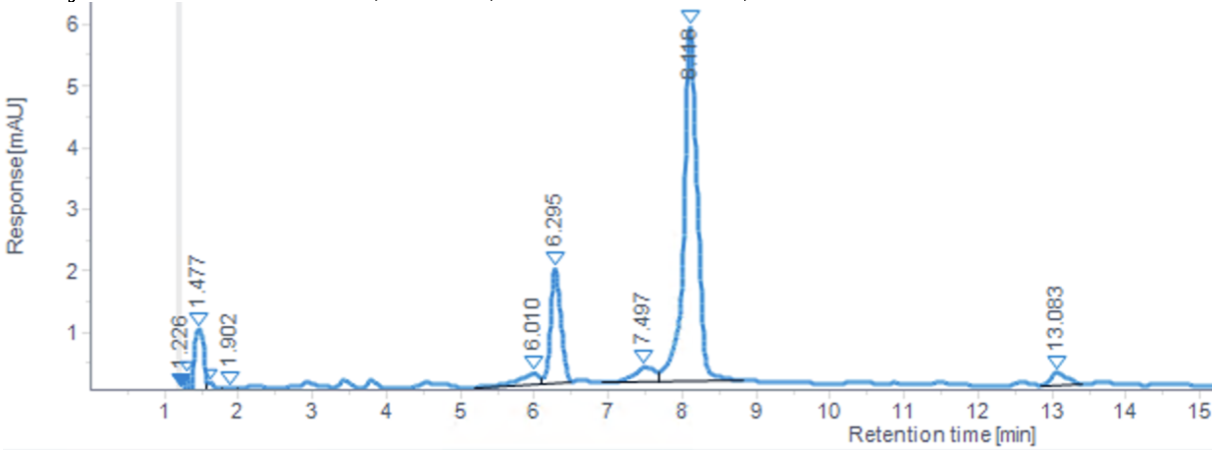

Entry 8: 6.2 min: **hexa-T-1´**, Boc-off, 8.1 min: **hexa-T-1**, Boc-off.

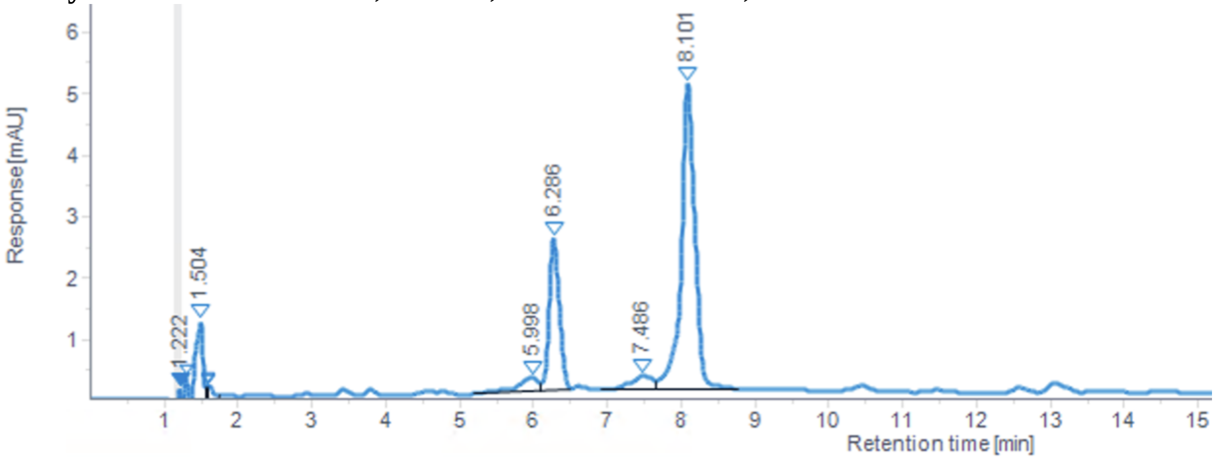

Entry 9: 9.8 min: **hexa-T-1´**, Boc-on, 11.9 min: **hexa-T-1**, Boc-on.

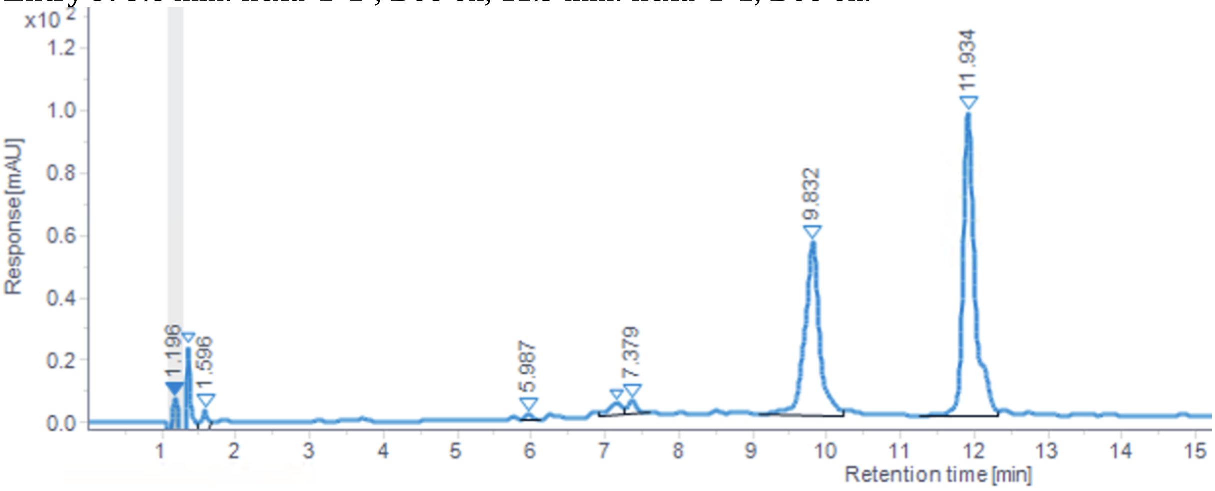

Entry 10: Phosphate buffer, 10.6 min: **hexa-T-1´**, Boc-on, 12.7 min: **hexa-T-1**, Boc-on.

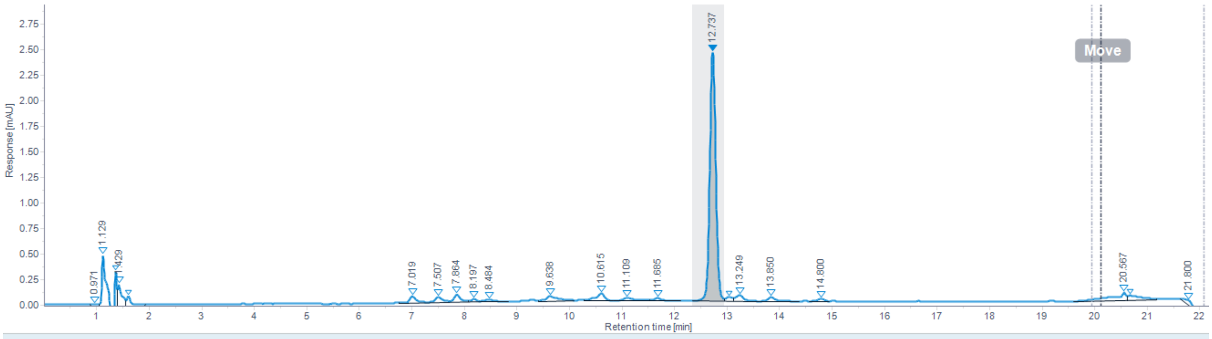

Injection Results

Peaks | Summary

#	Name	Signal description	RT (min)	Area (mAU·s)	Area%	Height (mAU)	Height%	Amount	Concentration	Start time (min)	End time (min)
10		DAD1A,Sig=260,4 Ref=off	8.484	19.803	0.414	1.835	0.34			8.308	8.840
11		DAD1A,Sig=260,4 Ref=off	9.638	86.271	1.805	4.731	0.87			9.418	10.102
12		DAD1A,Sig=260,4 Ref=off	10.615	85.013	1.779	7.086	1.30			10.287	10.846
13		DAD1A,Sig=260,4 Ref=off	11.109	41.896	0.877	2.772	0.51			10.846	11.538
14		DAD1A,Sig=260,4 Ref=off	11.685	28.493	0.596	2.695	0.49			11.538	12.133
15		DAD1A,Sig=260,4 Ref=off	12.737	1950.616	40.813	243.362	44.69			12.351	12.960
16		DAD1A,Sig=260,4 Ref=off	13.052	33.217	0.695	4.184	0.77			12.960	13.131

Entry 11: HEPES buffer, 10.6 min: **hexa-T-1´**, Boc-on, 12.7 min: **hexa-T-1**, Boc-on.

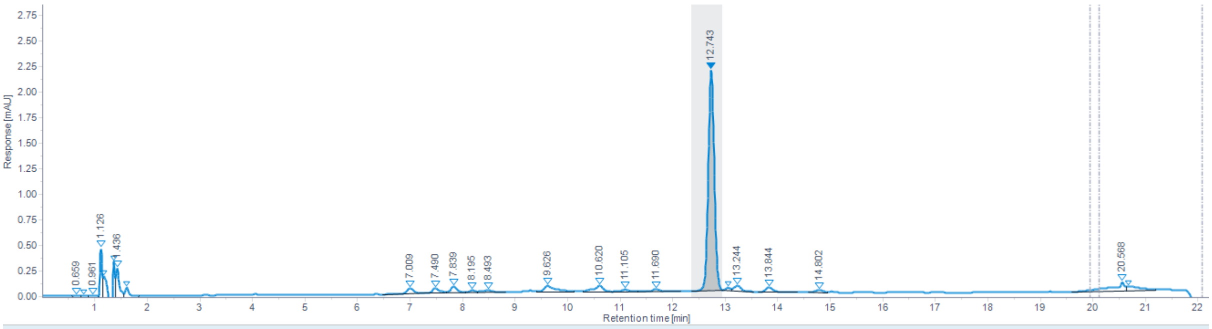

Injection Results

Peaks | Summary

#	Name	Signal description	RT (min)	Area (mAU·s)	Area%	Height (mAU)	Height%	Amount	Concentration	Start time (min)	End time (min)
14		DAD1A,Sig=260,4 Ref=off	9.626	95.844	2.037	5.779	0.97			9.420	10.129
15		DAD1A,Sig=260,4 Ref=off	10.620	73.531	1.563	5.925	1.00			10.311	10.847
16		DAD1A,Sig=260,4 Ref=off	11.105	18.815	0.400	1.855	0.31			10.850	11.353
17		DAD1A,Sig=260,4 Ref=off	11.690	30.586	0.650	2.623	0.44			11.353	12.162
18		DAD1A,Sig=260,4 Ref=off	12.743	1710.151	36.354	215.037	36.19			12.377	12.966

Entry 12: MES buffer, 10.6 min: **hexa-T-1´**, Boc-on, 12.7 min: **hexa-T-1**, Boc-on.

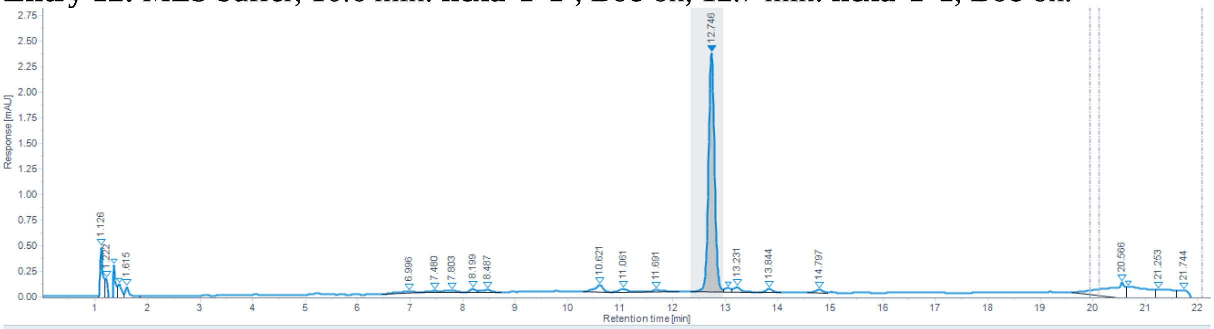

Injection Results

Peaks | Summary

#	Name	Signal description	RT (min)	Area (mAU·s)	Area%	Height (mAU)	Height%	Amount	Concentration	Start time (min)	End time (min)
10		DAD1A,Sig=260,4 Ref=off	8.487	30.712	0.576	2.503	0.45			8.300	8.743
11		DAD1A,Sig=260,4 Ref=off	10.621	77.040	1.446	7.020	1.25			10.312	10.824
12		DAD1A,Sig=260,4 Ref=off	11.061	31.855	0.598	2.788	0.50			10.830	11.531
13		DAD1A,Sig=260,4 Ref=off	11.691	28.606	0.537	2.379	0.42			11.531	12.130
14		DAD1A,Sig=260,4 Ref=off	12.746	1879.647	35.276	233.824	41.62			12.349	12.971
15		DAD1A,Sig=260,4 Ref=off	13.059	32.221	0.605	4.083	0.73			12.971	13.134

MALDI-MS: hexa-T-1´, Boc-off, calculated m/z 2235.

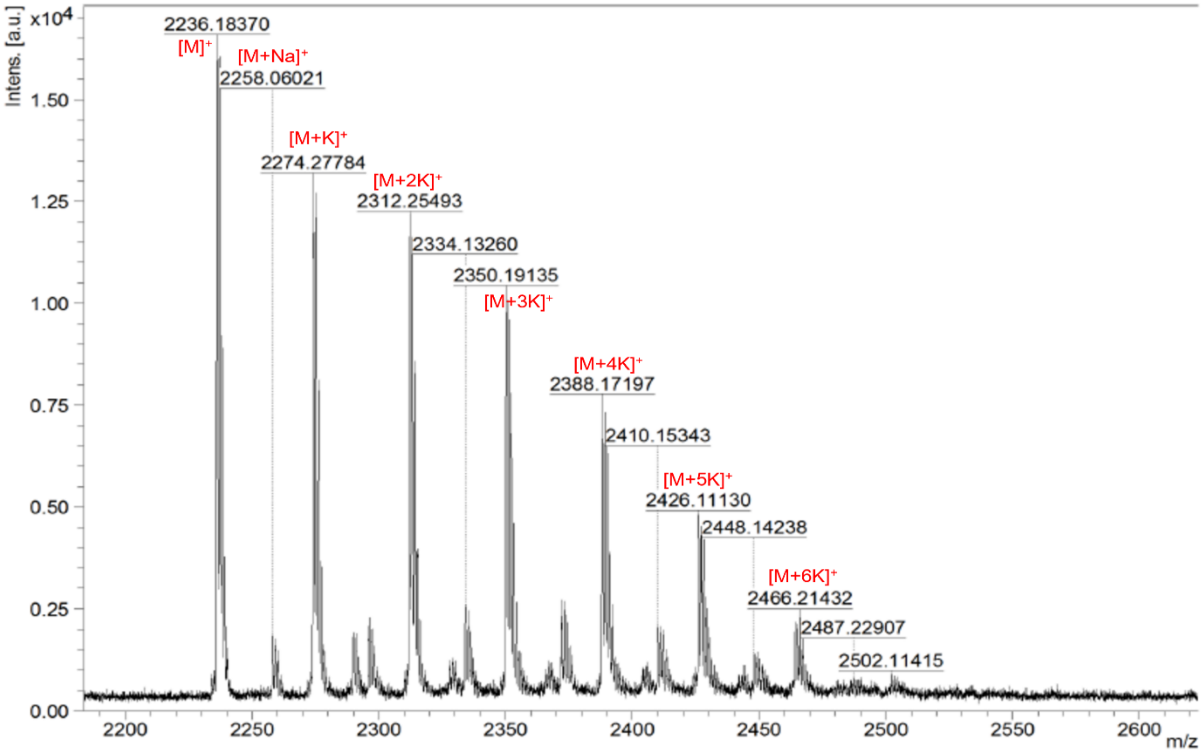

Figure S25. RP-HPLC traces for **Table S12** (entry 1-12), and MALDI-MS spectra for optimizing RCM reaction conditions with hexa-T-tagged substrate (**hexa-T-1**) in H_2O.

12. DNA precipitation assays

500 pmol of hexa-T (**hexa-T-1**), csDNA (**7DeATC-1**) and DNA (**ATGC-1**)-tagged substrates were dissolved in 50 μL H_2O (pH 2.0 for **hexa-T-1** and **7DeATC-1** samples, pH 5.0 for **ATGC-1** samples) together with the corresponding salts listed in Table S3. The reaction mixtures were shaken at 70 °C for 30 min. All samples were filtered and injected into analytical-HPLC for analysis. The DNA areas were integrated using Agilent GC_LC area percent method.

Table S13: Impact of the chloride salts on the solubility of **hexa-T-1** in H_2O.

Entry	Additives (equiv)	DNA integrated area
1	None, control	870
2	40,000 NaCl	787
3	20,000 $MgCl_2$	848
4	40,000 KCl	802
5	40,000 NMe_4Cl	844
6	40,000 $NHMe_3Cl$	679
7	40,000 NEt_4Cl	780
8	40,000 $NHEt_3Cl$	872
9	40,000 N^nBu_4Cl	1073

RP-HPLC chromatograms of hexa-T-1 precipitation assays.

Entry 1:

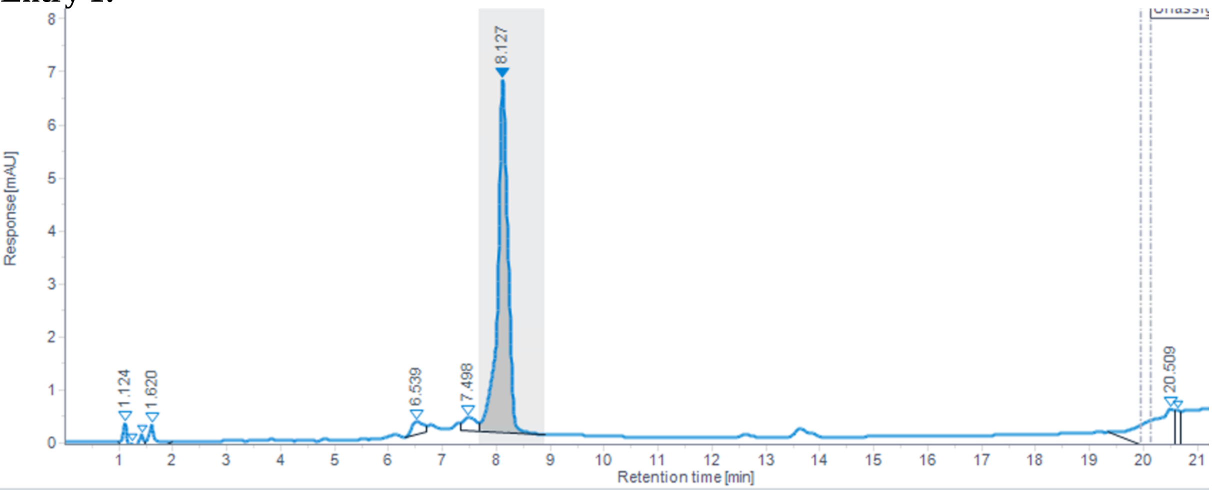

Injection Results

Peaks | Summary

#	Name	Signal description	RT (min)	Area (mAU·s)	Area%	Height (mAU)	Height%
7		DAD1B,Sig=280,4 Ref=off	8.127	870.257	32.002	66.373	50.90

Entry 2:

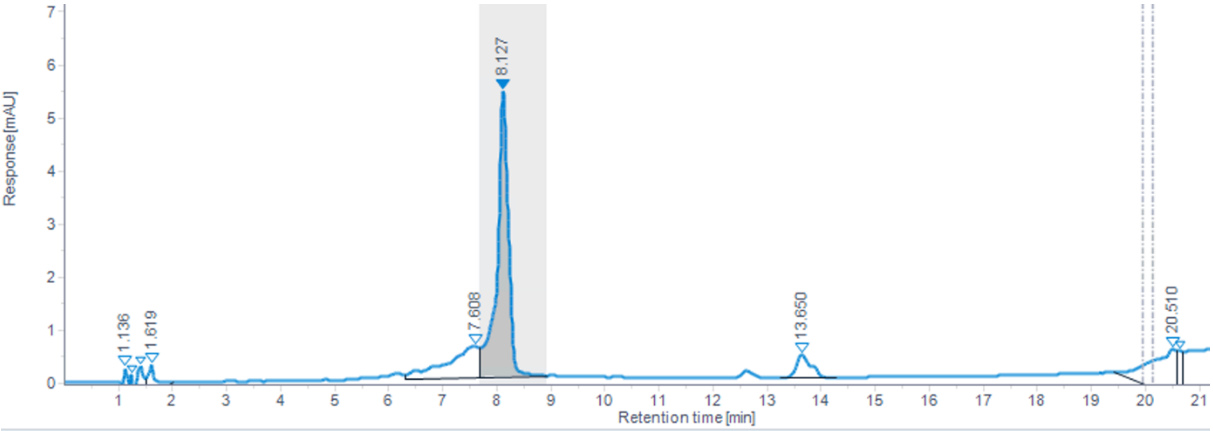

Injection Results

Peaks | Summary

#	Name	Signal description	RT (min)	Area (mAU·s)	Area%	Height (mAU)	Height%
6		DAD1B,Sig=280,4 Ref=off	8.127	786.981	27.159	53.785	42.02

Entry 3:

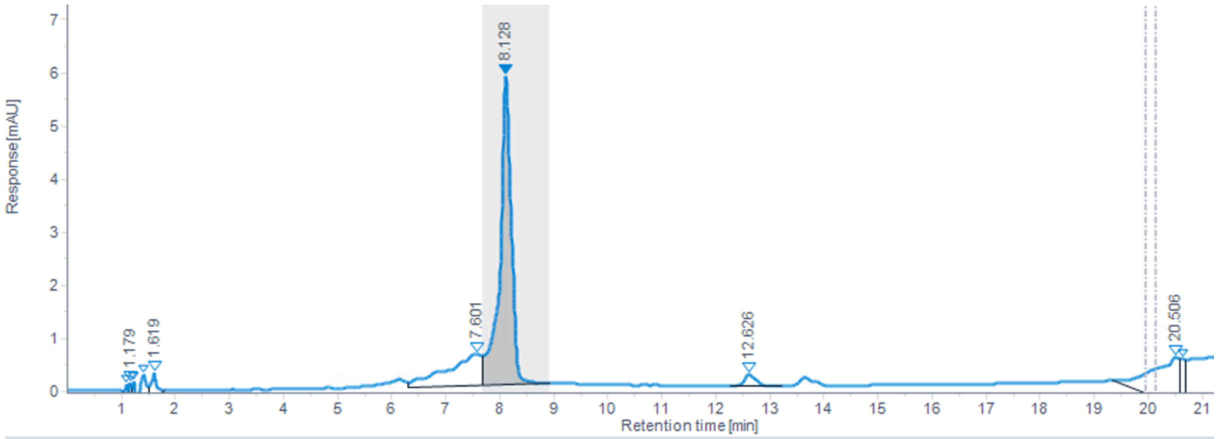

Injection Results

Peaks | Summary

#	Name	Signal description	RT (min)	Area (mAU·s)	Area%	Height (mAU)	Height%
7		DAD1B,Sig=280,4 Ref=off	8.128	848.479	29.097	57.892	44.85

Entry 4:

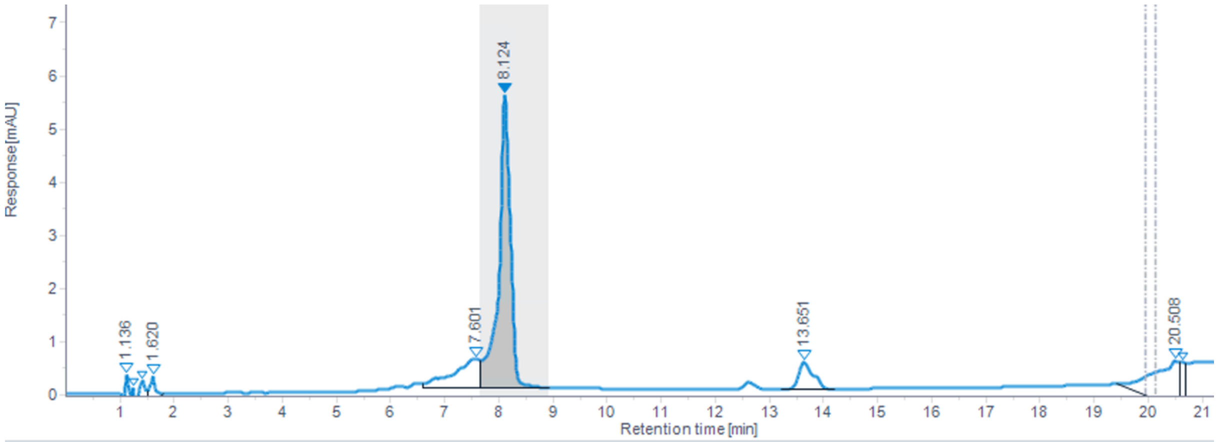

Injection Results

Peaks | Summary

#	Name	Signal description	RT (min)	Area (mAU·s)	Area%	Height (mAU)	Height%
6		DAD1B,Sig=280,4 Ref=off	8.124	801.916	28.489	54.947	43.01

Entry 5:

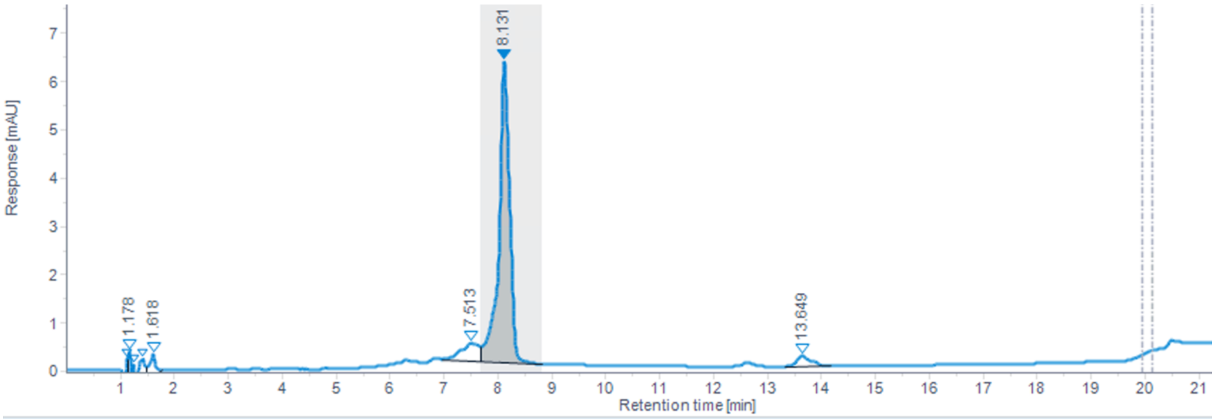

Injection Results

Peaks | Summary

#	Name	Signal description	RT (min)	Area (mAU·s)	Area%	Height (mAU)	Height%
4		DAD1B,Sig=280,4 Ref=off	1.423	44.793	3.025	5.228	5.09
5		DAD1B,Sig=280,4 Ref=off	1.618	37.058	2.503	4.613	4.49
6		DAD1B,Sig=280,4 Ref=off	7.513	83.736	5.655	3.596	3.50
7		DAD1B,Sig=280,4 Ref=off	8.131	843.616	56.977	62.272	60.57

Entry 6:

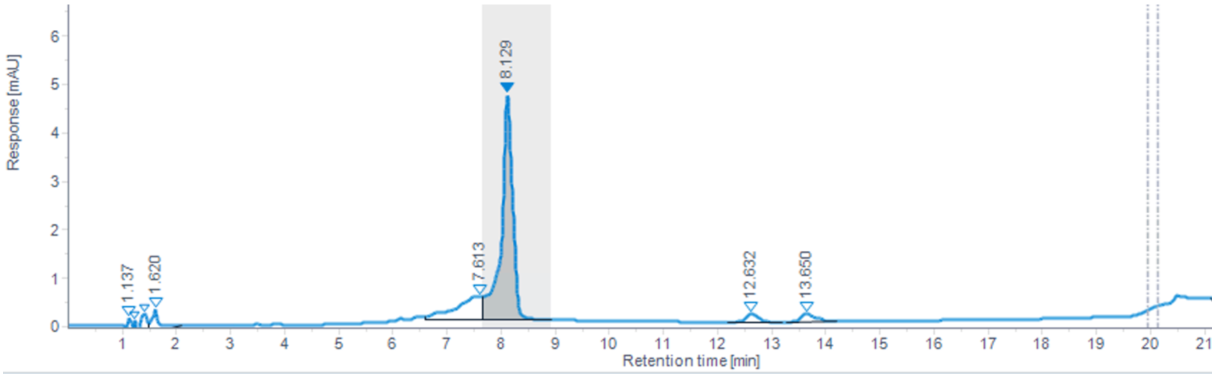

Injection Results

Peaks | Summary

#	Name	Signal description	RT (min)	Area (mAU·s)	Area%	Height (mAU)	Height%
5		DAD1B,Sig=280,4 Ref=off	7.613	173.444	10.288	4.943	5.62
6		DAD1B,Sig=280,4 Ref=off	8.129	679.059	40.280	46.395	52.73

Entry 7:

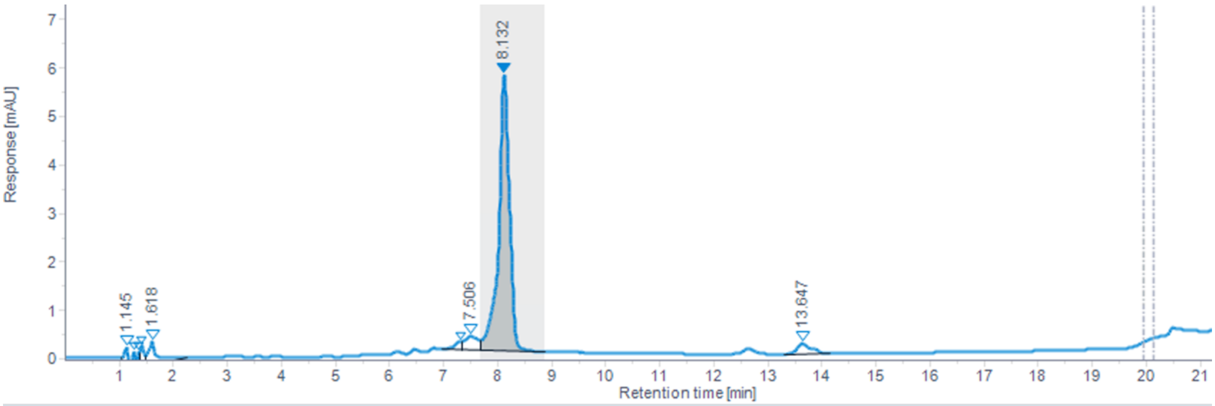

Injection Results

Peaks Summary

#	Name	Signal description	RT (min)	Area (mAU·s)	Area%	Height (mAU)	Height%
5		DAD1B,Sig=280,4 Ref=off	1.618	56.812	4.002	4.716	4.90
6		DAD1B,Sig=280,4 Ref=off	7.317	16.970	1.195	1.802	1.87
7		DAD1B,Sig=280,4 Ref=off	7.506	49.761	3.505	2.881	2.99
8		DAD1B,Sig=280,4 Ref=off	8.132	779.718	54.927	56.869	59.04

Entry 8:

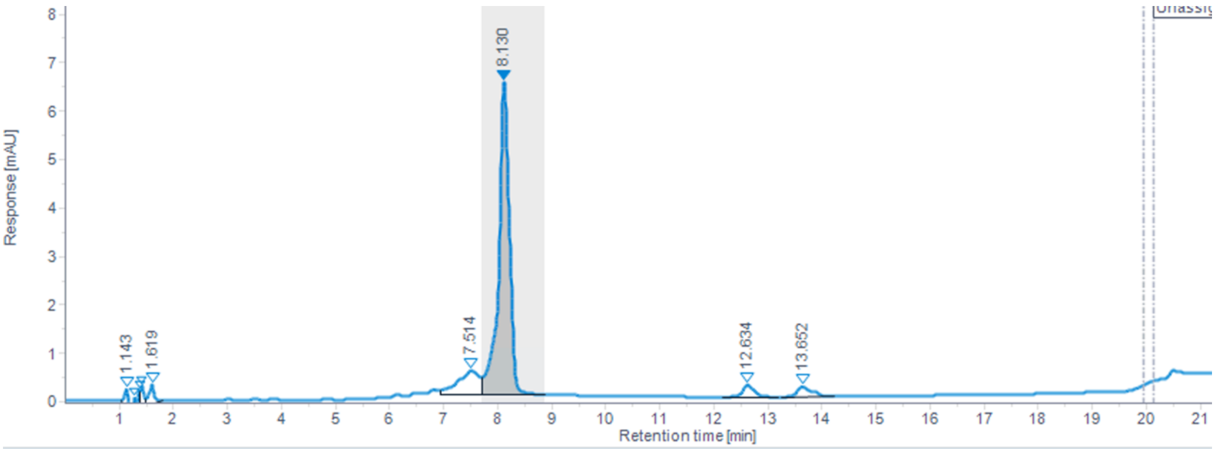

Injection Results

Peaks Summary

#	Name	Signal description	RT (min)	Area (mAU·s)	Area%	Height (mAU)	Height%
5		DAD1B,Sig=280,4 Ref=off	1.619	27.516	1.741	3.948	3.75
6		DAD1B,Sig=280,4 Ref=off	7.514	132.010	8.352	4.858	4.61
7		DAD1B,Sig=280,4 Ref=off	8.130	871.883	55.164	64.424	61.12

Entry 9:

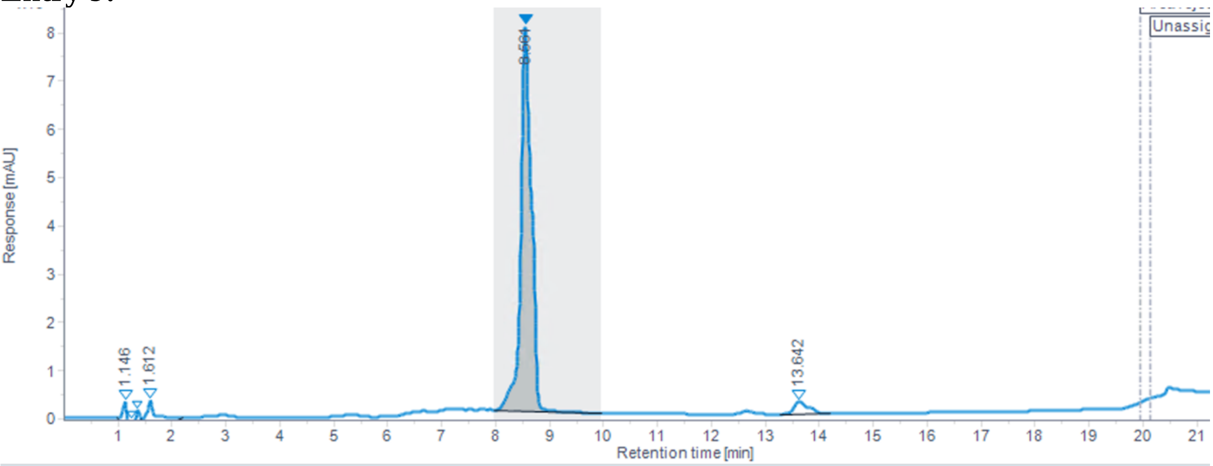

Injection Results

Peaks | Summary

#	Name	Signal description	RT (min)	Area (mAU·s)	Area%	Height (mAU)	Height%
2		DAD1B,Sig=280,4 Ref=off	1.266	7.942	0.467	3.347	2.95
3		DAD1B,Sig=280,4 Ref=off	1.376	35.392	2.080	5.867	5.17
4		DAD1B,Sig=280,4 Ref=off	1.612	109.973	6.465	6.707	5.91
5		DAD1B,Sig=280,4 Ref=off	8.561	1072.998	63.076	79.638	70.23

Figure S26. RP-HPLC traces for **Table S13** (entry 1-9) for impact of the chloride salts on the solubility of **hexa-T-1** in H_2O.

Table S14: Impact of the chloride salts on the solubility of **7DeATC-1** in H_2O.

Entry	Additives (equiv)	DNA integrated area
1	None, control	1289
2	40,000 NaCl	1298
3	20,000 $MgCl_2$	1418
4	40,000 KCl	1332
5	40,000 NMe_4Cl	1182
6	40,000 $NHMe_3Cl$	1058
7	40,000 NEt_4Cl	832
8	40,000 $NHEt_3Cl$	1433
9	40,000 N^nBu_4Cl	2467
10	40,000 $NHEt_3Cl$<br>40,000 NaCl	1630

RP-HPLC chromatograms of 7DeATC-1 precipitation assays.

Entry 1:

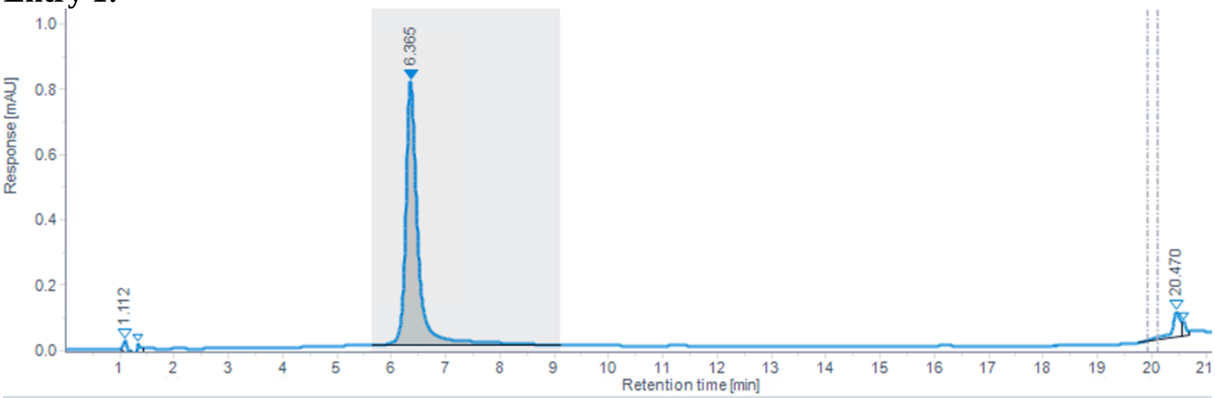

Injection Results

Peaks | Summary

#	Name	Signal description	RT (min)	Area (mAU·s)	Area%	Height (mAU)	Height%
2		DAD1B,Sig=280,4 Ref=off	1.351	19.980	0.937	3.826	3.18
3		DAD1B,Sig=280,4 Ref=off	6.365	1289.271	60.489	80.994	67.23

Entry 2:

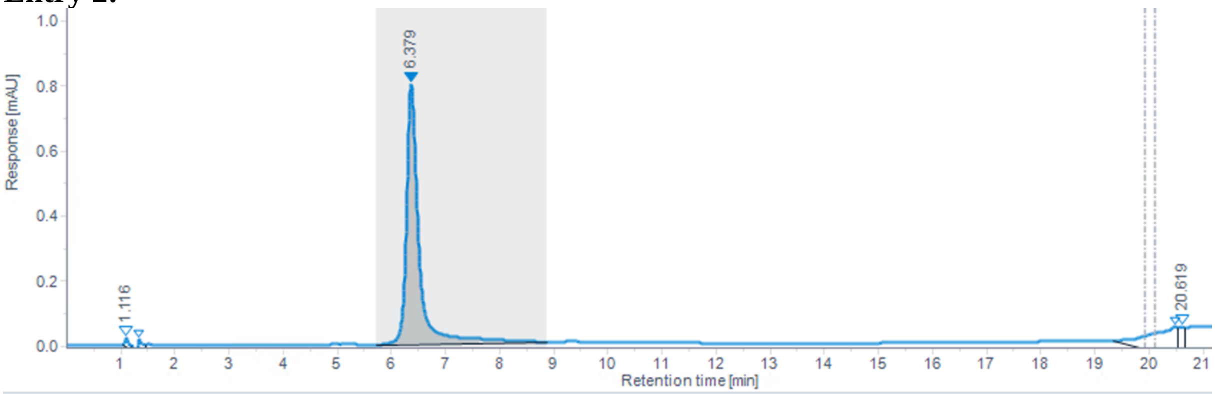

-2024-0829-1259-03066
CHWEIGER/CHUN ZHANG/CHUN DEFAULT/Results

Peaks | Summary

#	Name	Signal description	RT (min)	Area (mAU·s)	Area%	Height (mAU)	Height%
2		DAD1B,Sig=280,4 Ref=off	1.352	19.288	0.612	3.497	2.66
3		DAD1B,Sig=280,4 Ref=off	6.379	1298.560	41.177	79.931	60.80

Entry 3:

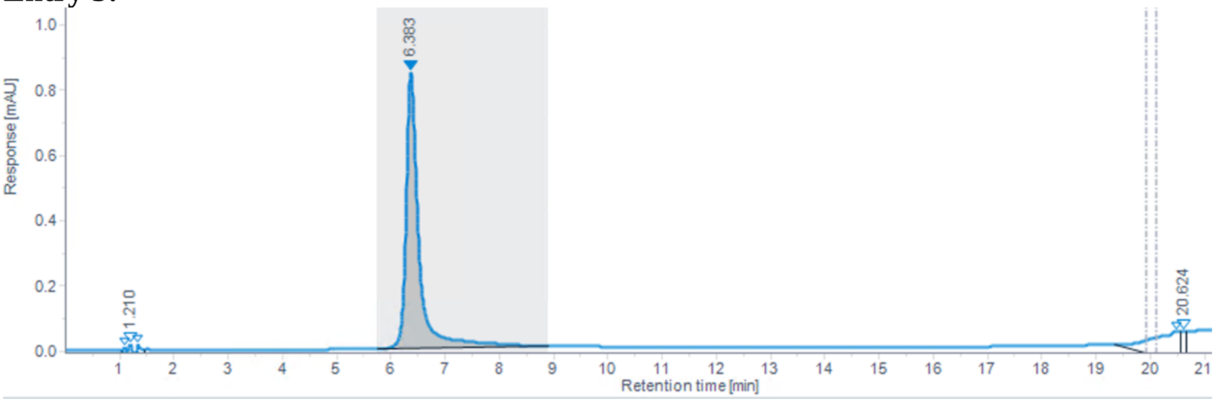

Injection Results

Peaks | Summary

#	Name	Signal description	RT (min)	Area (mAU·s)	Area%	Height (mAU)	Height%
2		DAD1B,Sig=280,4 Ref=off	1.210	12.334	0.375	3.137	2.27
3		DAD1B,Sig=280,4 Ref=off	1.353	13.982	0.425	2.690	1.95
4		DAD1B,Sig=280,4 Ref=off	6.383	1418.455	43.124	84.583	61.22

Entry 4:

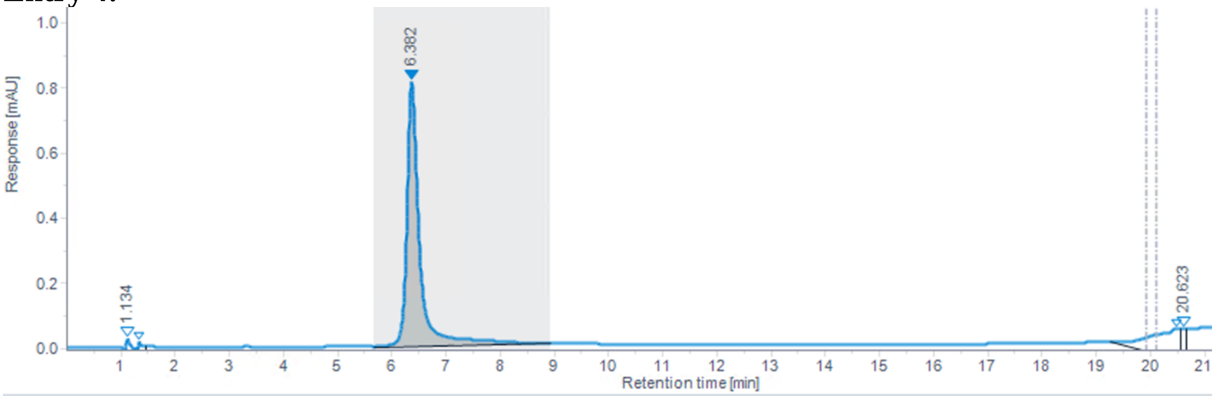

Injection Results

Peaks | Summary

#	Name	Signal description	RT (min)	Area (mAU·s)	Area%	Height (mAU)	Height%
2		DAD1B,Sig=280,4 Ref=off	1.352	16.709	0.517	3.239	2.41
3		DAD1B,Sig=280,4 Ref=off	6.382	1332.482	41.244	81.019	60.25

Entry 5:

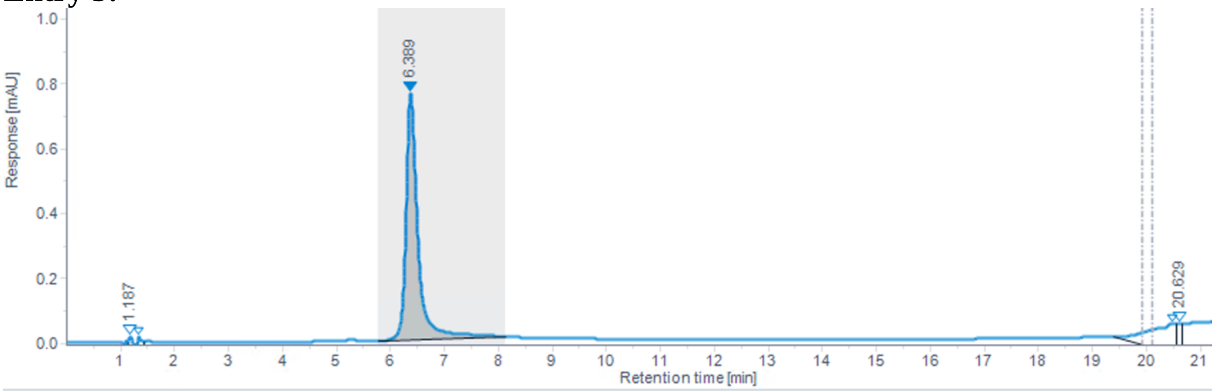

Injection Results

Peaks | Summary

#	Name	Signal description	RT (min)	Area (mAU·s)	Area%	Height (mAU)	Height%
2		DAD1B,Sig=280,4 Ref=off	1.353	16.625	0.547	3.213	2.51
3		DAD1B,Sig=280,4 Ref=off	6.389	1181.855	38.906	76.089	59.38

Entry 6:

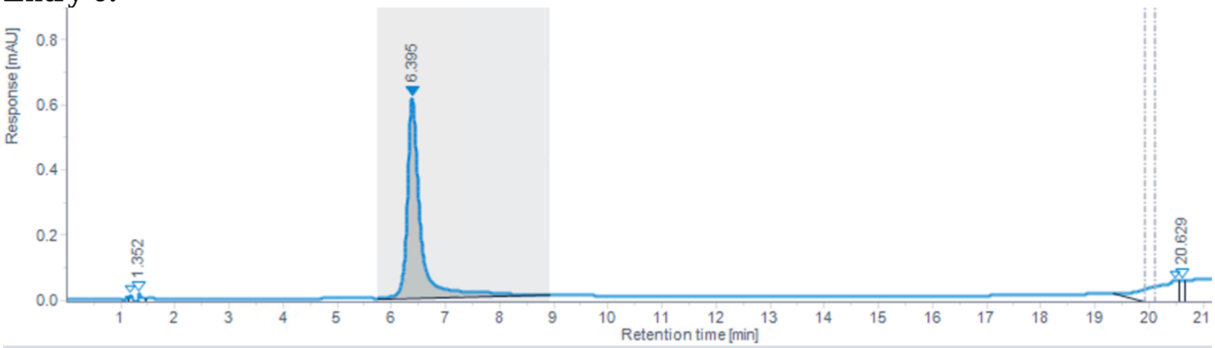

Injection Results

Peaks | Summary

#	Name	Signal description	RT (min)	Area (mAU·s)	Area%	Height (mAU)	Height%
2		DAD1B,Sig=280,4 Ref=off	1.352	17.659	0.609	3.272	2.92
3		DAD1B,Sig=280,4 Ref=off	6.395	1058.095	36.483	61.491	54.83

Entry 7:

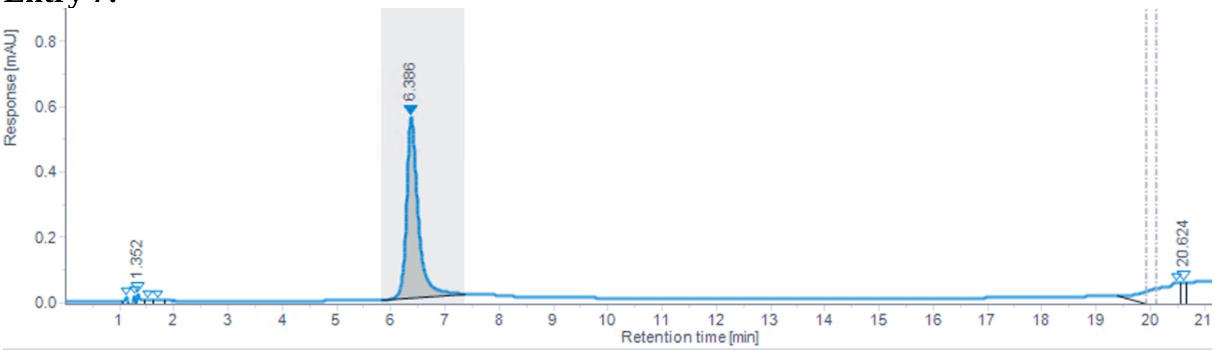

Injection Results

Peaks | Summary

#	Name	Signal description	RT (min)	Area (mAU·s)	Area%	Height (mAU)	Height%
5		DAD1B,Sig=280,4 Ref=off	1.726	24.733	0.887	2.252	1.88
6		DAD1B,Sig=280,4 Ref=off	6.386	832.867	29.861	55.451	46.19

Entry 8:

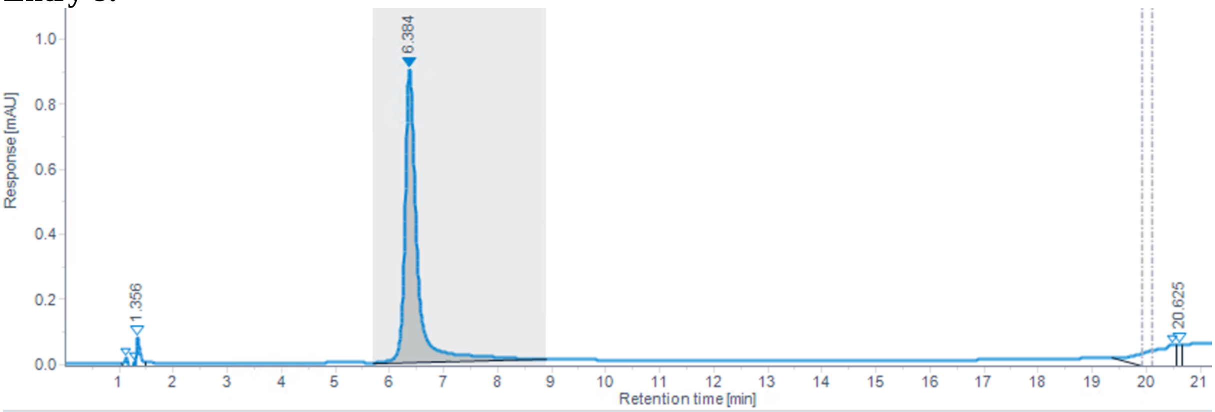

Injection Results

Peaks | Summary

#	Name	Signal description	RT (min)	Area (mAU·s)	Area%	Height (mAU)	Height%
3		DAD1B,Sig=280,4 Ref=off	1.356	57.543	1.722	11.130	7.17
4		DAD1B,Sig=280,4 Ref=off	6.384	1433.266	42.896	90.151	58.06

Entry 9: 7.8 min: **7DeATC-1**, Boc-off, N^nBu_4Cl led to longer retention time.

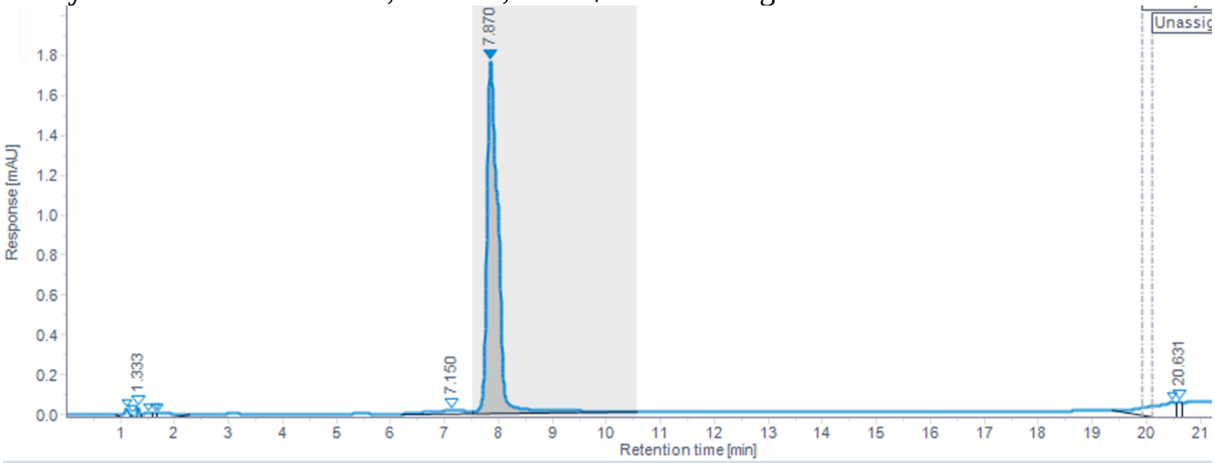

Injection Results

Peaks | Summary

#	Name	Signal description	RT (min)	Area (mAU·s)	Area%	Height (mAU)	Height%
7		DAD1B,Sig=280,4 Ref=off	7.150	78.752	1.713	1.836	0.73
8		DAD1B,Sig=280,4 Ref=off	7.870	2467.465	53.668	176.235	70.07

Entry 10:

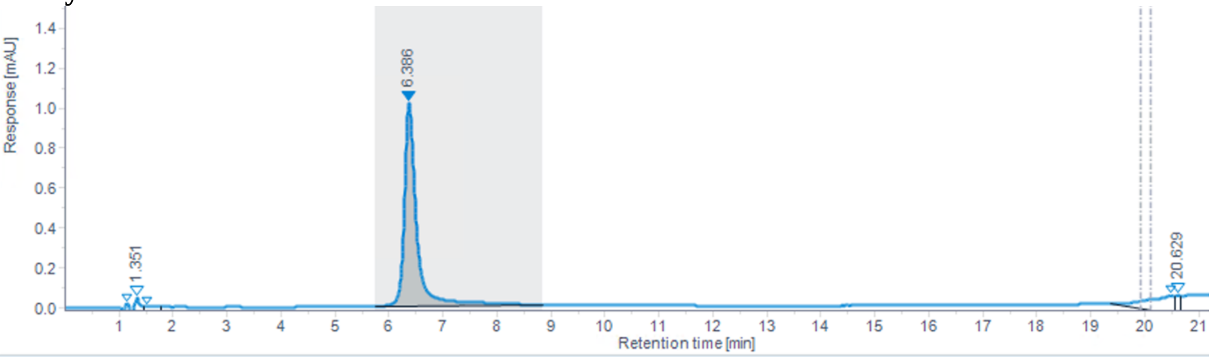

Injection Results

Peaks Summary

#	Name	Signal description	RT (min)	Area (mAU·s)	Area%	Height (mAU)	Height%
3		DAD1B,Sig=280,4 Ref=off	1.539	71.890	1.959	5.115	3.00
4		DAD1B,Sig=280,4 Ref=off	6.386	1630.391	44.426	101.575	59.56

Figure S27. RP-HPLC traces for **Table S14** (entry 1-10) for impact of chloride salts on the solubility of **7DeATC-1** in H_2O.

Table S15 Impact of chloride salts on the solubility of **ATGC-1** in H_2O.

Entry	Additives (equiv)	DNA integrated area
1	None, control	1866
2	40,000 NaCl	1298
3	20,000 $MgCl_2$	86
4	40,000 KCl	1530
5	40,000 NMe_4Cl	1670
6	40,000 $NHMe_3Cl$	932
7	40,000 NEt_4Cl	698
8	40,000 $NHEt_3Cl$	1249
9	40,000 N^nBu_4Cl	1092

RP-HPLC chromatograms of ATGC-1 precipitation assays.

Entry 1:

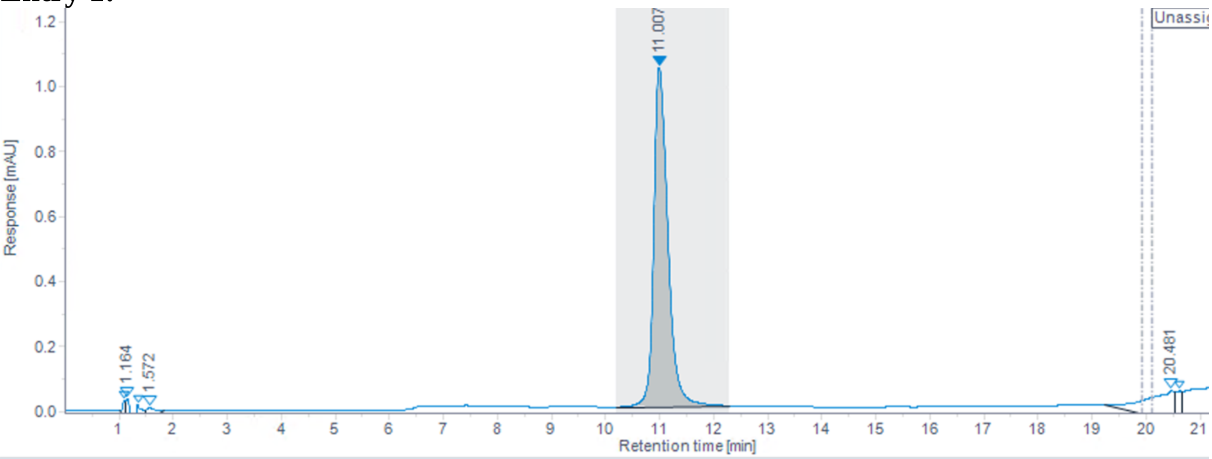

Injection Results

Peaks | Summary

#	Name	Signal description	RT (min)	Area (mAU·s)	Area%	Height (mAU)	Height%
4		DAD1B,Sig=280,4 Ref=off	1.572	41.057	1.066	3.500	2.04
5		DAD1B,Sig=280,4 Ref=off	11.007	1866.468	48.438	104.306	60.64

Entry 2:

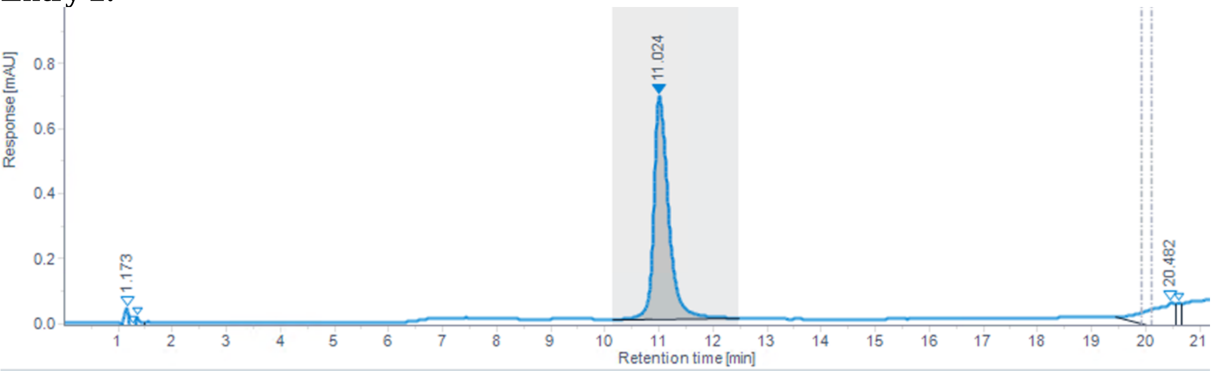

Injection Results

Peaks | Summary

#	Name	Signal description	RT (min)	Area (mAU·s)	Area%	Height (mAU)	Height%
3		DAD1B,Sig=280,4 Ref=off	1.361	36.088	1.144	5.451	4.25
4		DAD1B,Sig=280,4 Ref=off	11.024	1298.942	41.181	68.473	53.41

Entry 3:

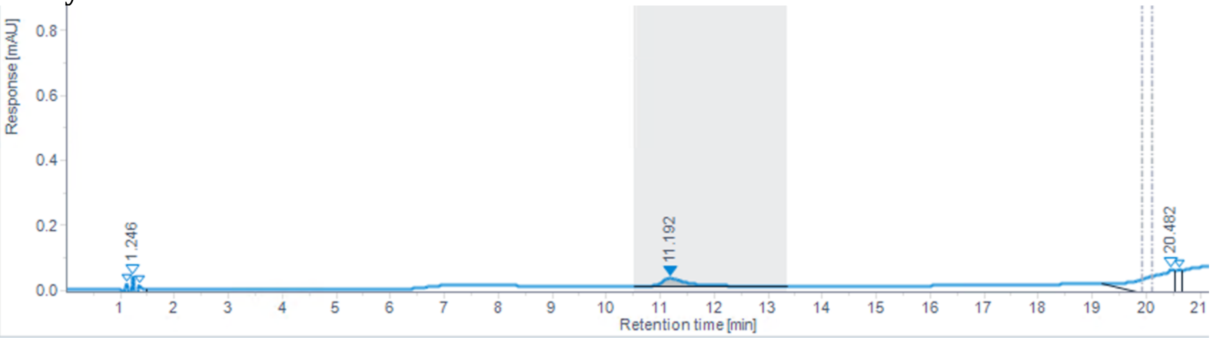

Injection Results

Peaks | Summary

#	Name	Signal description	RT (min)	Area (mAU·s)	Area%	Height (mAU)	Height%
3		DAD1B,Sig=280,4 Ref=off	1.366	26.748	1.327	3.964	6.22
4		DAD1B,Sig=280,4 Ref=off	11.192	86.020	4.269	2.448	3.84

Entry 4:

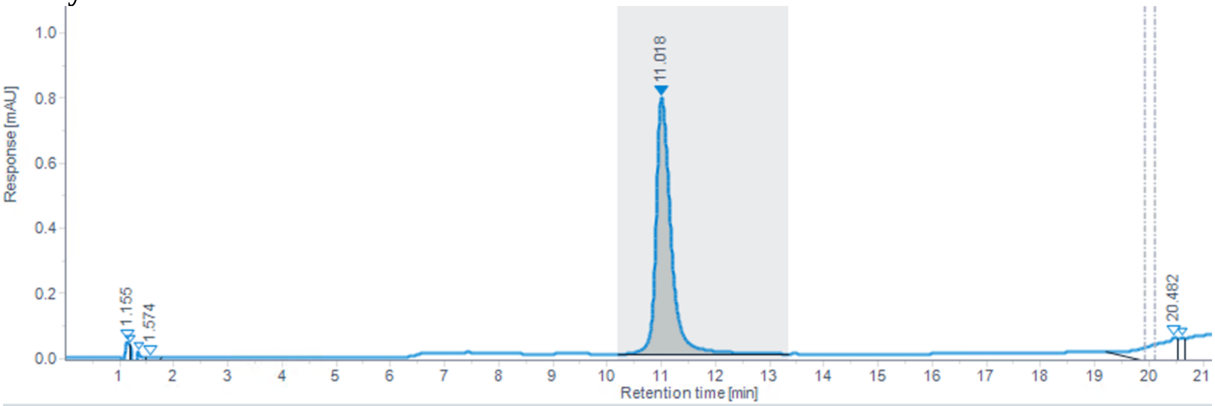

Injection Results

Peaks | Summary

#	Name	Signal description	RT (min)	Area (mAU·s)	Area%	Height (mAU)	Height%
4		DAD1B,Sig=280,4 Ref=off	1.574	44.709	1.238	4.148	2.58
5		DAD1B,Sig=280,4 Ref=off	11.018	1530.896	42.377	78.980	49.14

Entry 5:

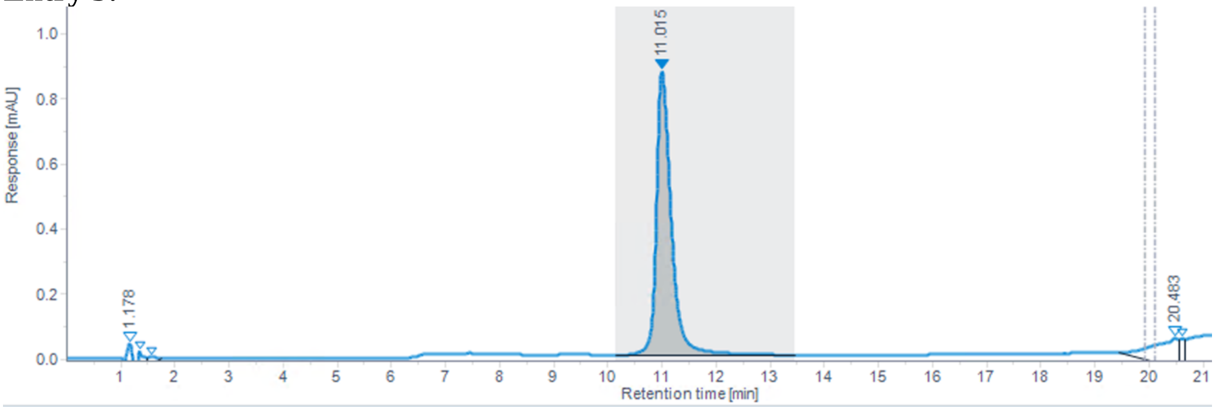

Injection Results

Peaks | Summary

#	Name	Signal description	RT (min)	Area (mAU·s)	Area%	Height (mAU)	Height%
3		DAD1B,Sig=280,4 Ref=off	1.570	23.069	0.647	2.401	1.62
4		DAD1B,Sig=280,4 Ref=off	11.015	1669.682	46.818	87.356	58.77

Entry 6: 6.5 min: **ATGC-1**, Boc-off, 11.0 min: **ATGC-1**, Boc-on.

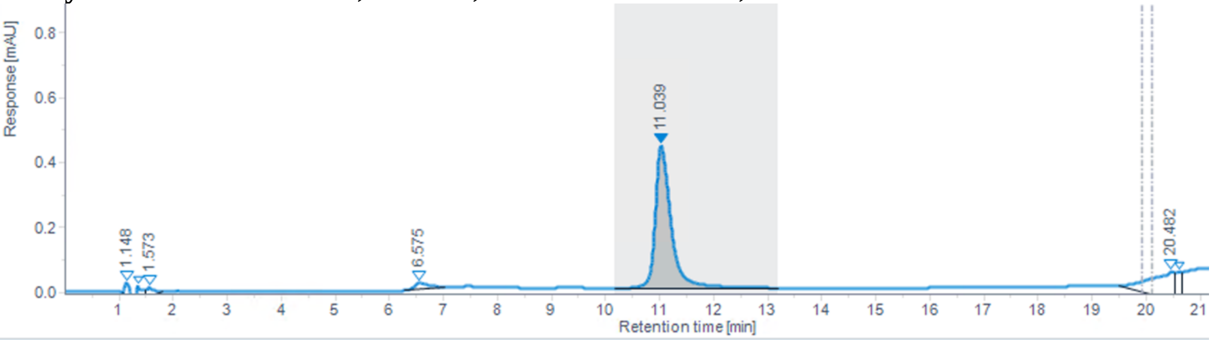

Injection Results

Peaks | Summary

#	Name	Signal description	RT (min)	Area (mAU·s)	Area%	Height (mAU)	Height%
3		DAD1B,Sig=280,4 Ref=off	1.573	24.272	0.874	2.515	2.47
4		DAD1B,Sig=280,4 Ref=off	6.575	34.175	1.231	1.957	1.92
5		DAD1B,Sig=280,4 Ref=off	11.039	898.159	32.360	43.952	43.15

Entry 7: 6.5 min: **ATGC-1**, Boc-off, 11.0 min: **ATGC-1**, Boc-on.

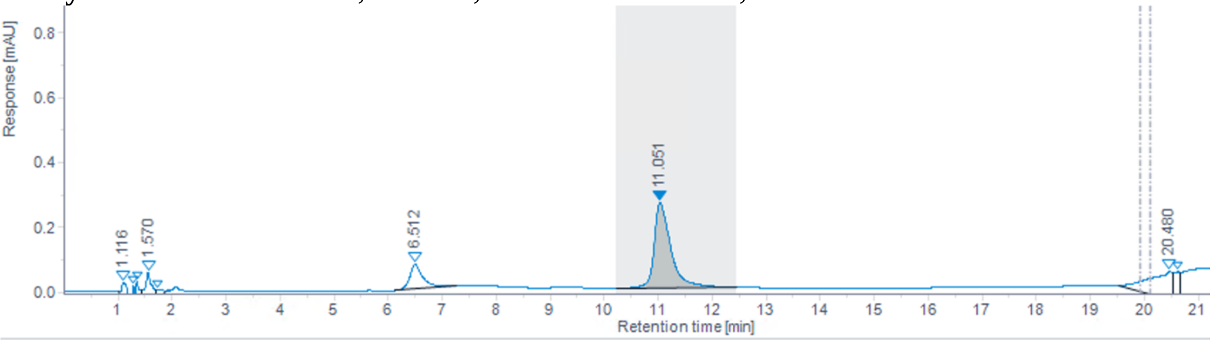

Injection Results

Peaks | Summary

#	Name	Signal description	RT (min)	Area (mAU·s)	Area%	Height (mAU)	Height%
6		DAD1B,Sig=280,4 Ref=off	6.512	133.223	4.949	7.641	6.54
7		DAD1B,Sig=280,4 Ref=off	11.051	565.405	21.003	26.301	22.51

Entry 8: 6.4 min: **ATGC-1**, Boc-off, 11.0 min: **ATGC-1**, Boc-on.

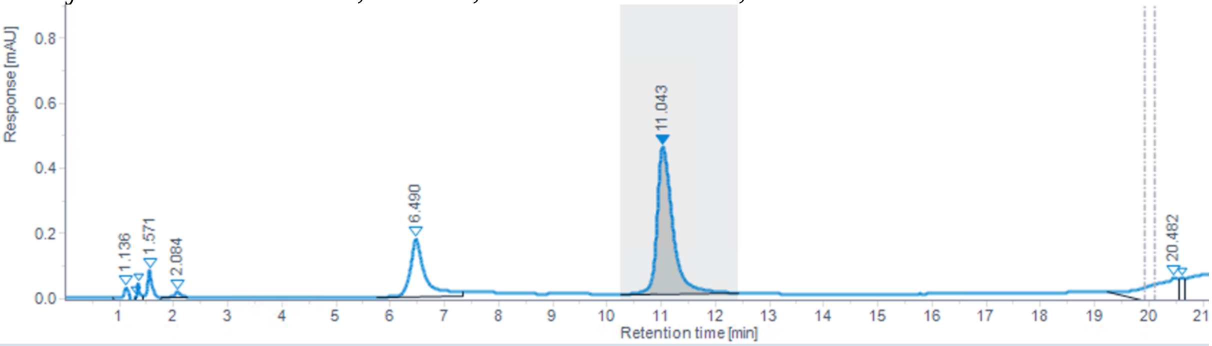

Injection Results

Peaks | Summary

#	Name	Signal description	RT (min)	Area (mAU·s)	Area%	Height (mAU)	Height%
6		DAD1B,Sig=280,4 Ref=off	6.490	335.079	9.578	17.672	10.81
7		DAD1B,Sig=280,4 Ref=off	11.043	914.393	26.136	45.305	27.72

Entry 9: 7.8 min: **ATGC-1**, Boc-off, 11.0 min: **ATGC-1**, Boc-on.

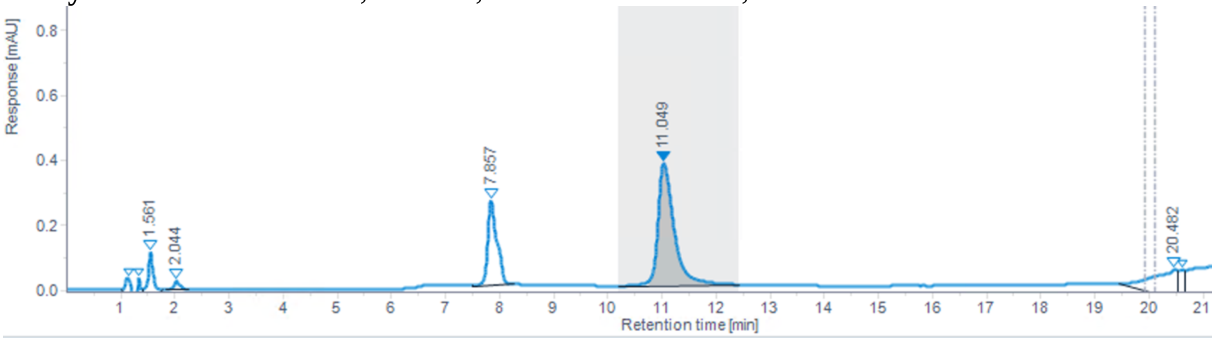

Injection Results

Peaks | Summary

#	Name	Signal description	RT (min)	Area (mAU·s)	Area%	Height (mAU)	Height%
5		DAD1B,Sig=280,4 Ref=off	7.857	309.625	9.989	26.195	18.36
6		DAD1B,Sig=280,4 Ref=off	11.049	782.351	25.239	37.572	26.33

Figure S28. RP-HPLC traces for **Table S15** (entry 1-9) showing impact of chloride salts on the solubility of **ATGC-1** in H_2O.

12. Substrate scope of hexa-T-tagged macrocycles (hexa-T-2´-7´)

Macrocycles **hexa-T-2´-7´** were synthesized as described in General Procedure 2.
RCM conditions: 400 mM (40,000 equiv) NaCl, 400 mM (40,000 equiv) $NHEt_3Cl$, 10 equiv **Ru-1**, pH 2.0, 70 °C, 60 min.

(a)

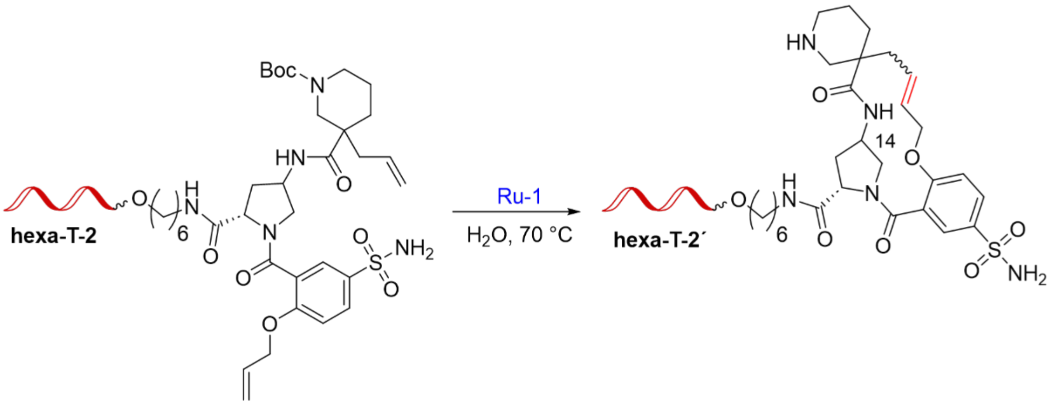

RP-HPLC chromatogram of hexa-T-2´ diene after RCM in H_2O
6.6 min: **hexa-T-2´**, Boc-off. 10.3 min: **hexa-T-2´**, Boc-on. 12.0 min: **hexa-T-2**, Boc-on. **60% in situ yield**.

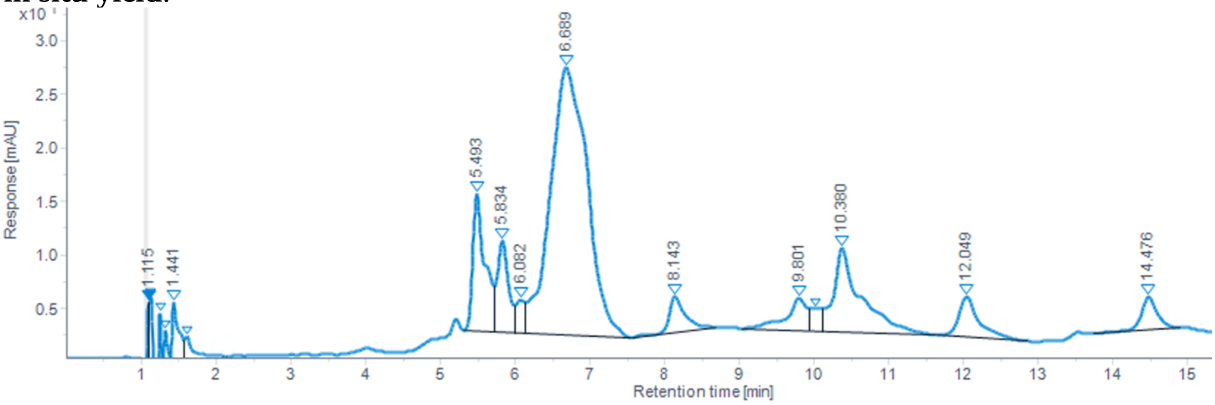

Full MALDI-MS: hexa-T-2´, Boc-off, calculated m/z 2517.

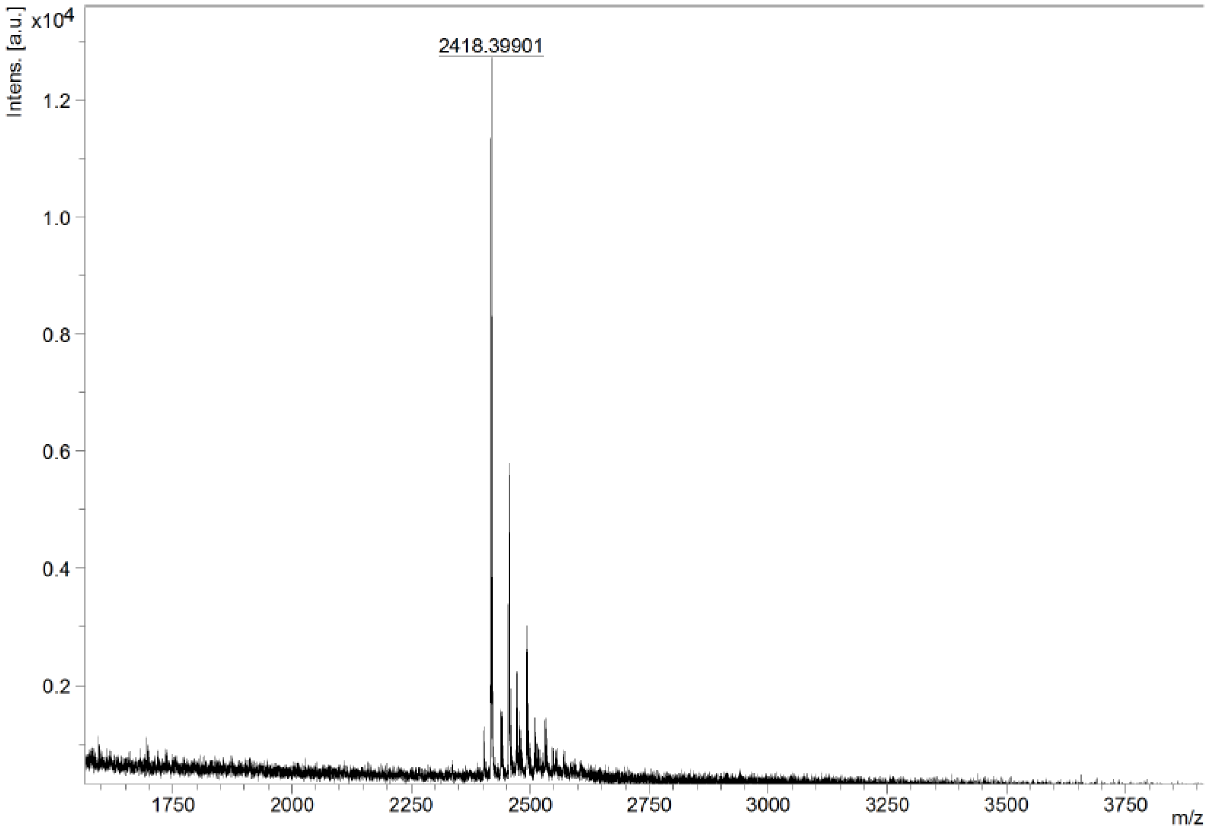

Zoomed-in MALDI-MS: hexa-T-2´, Boc-off, calculated m/z 2517.

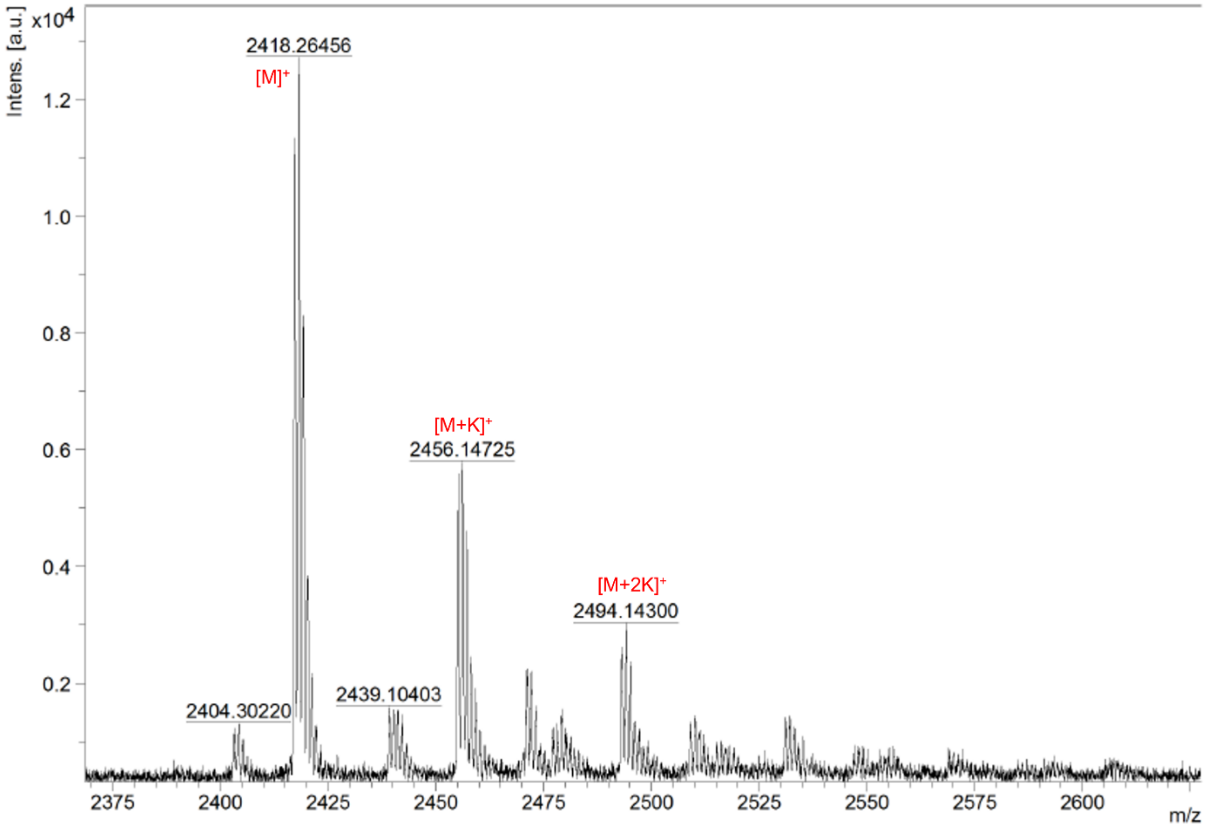

(b)

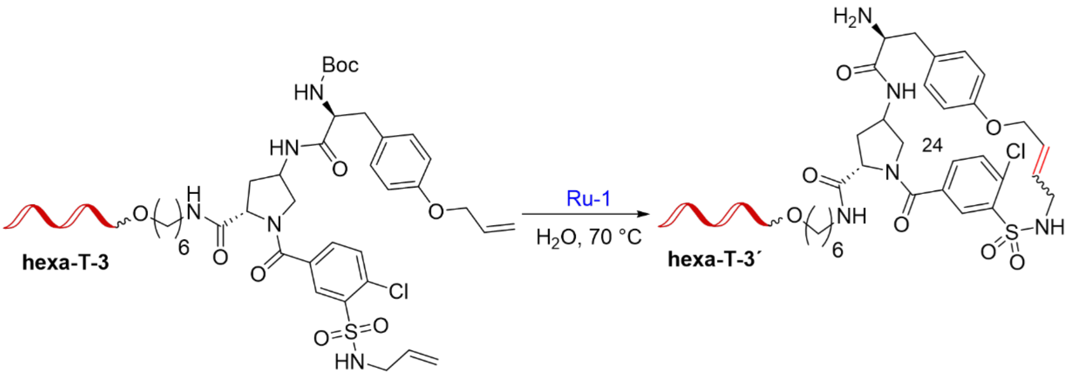

RP-HPLC chromatogram of hexa-T-3 diene after RCM in H_2O.
7.7 min and 8.0 min: **hexa-T-3´**. Boc-off. 9.9 min: **hexa-T-3**. Boc-off. **87% in situ yield.**

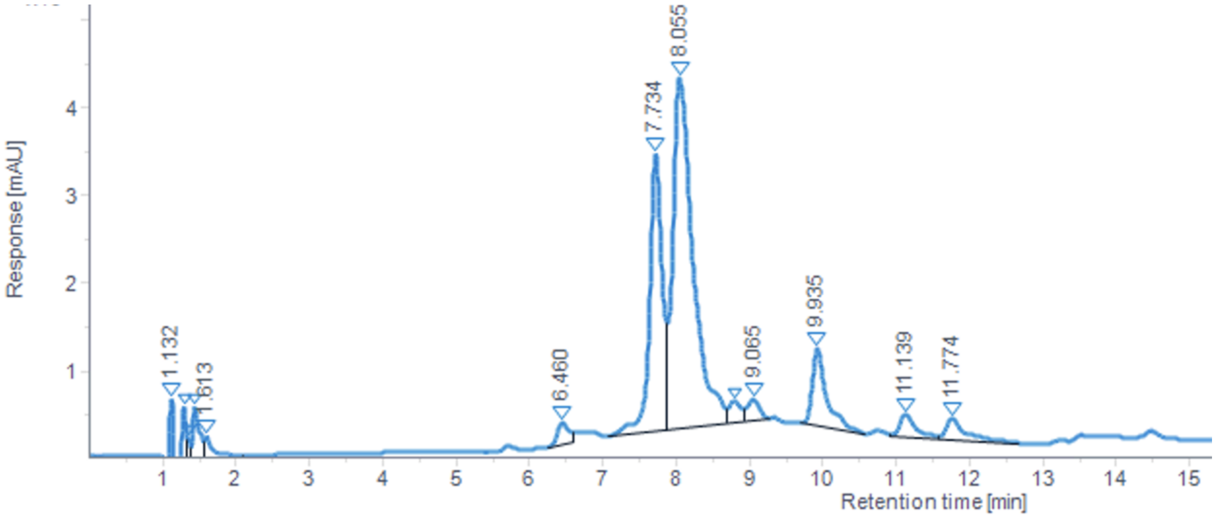

MALDI-MS: **hexa-T-3´**, Boc-off, calculated m/z 2487.

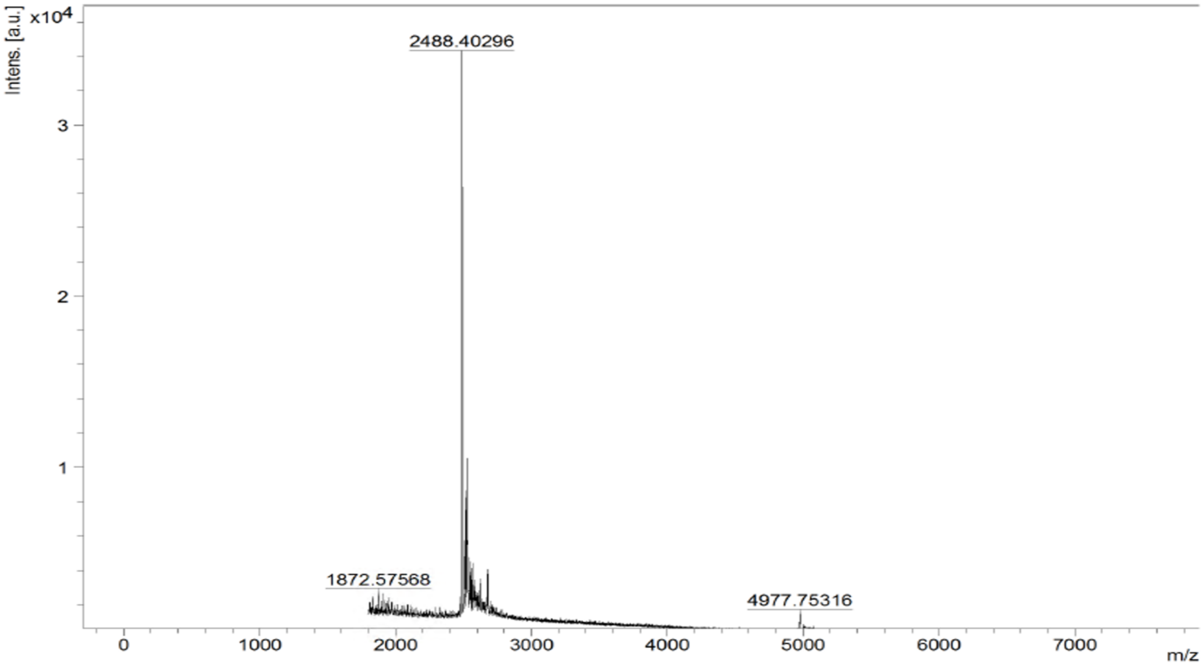

(c)

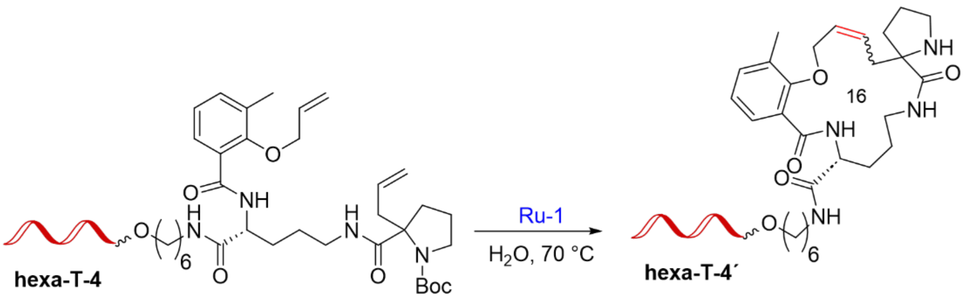

RP-HPLC chromatogram of hexa-T-4 diene after RCM in H_2O.
8.0 min and 8.6 min: **hexa-T-4´**, Boc-off. 11.1 min: **hexa-T-4**, Boc-off. **55% in situ yield**.

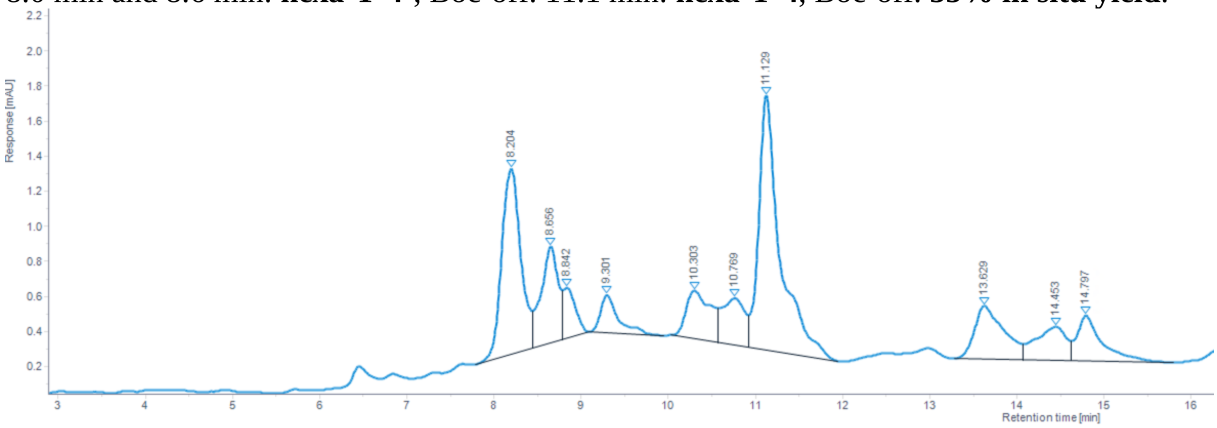

MALDI-MS: hexa-T-4´, Boc-off, calculated m/z 2339.

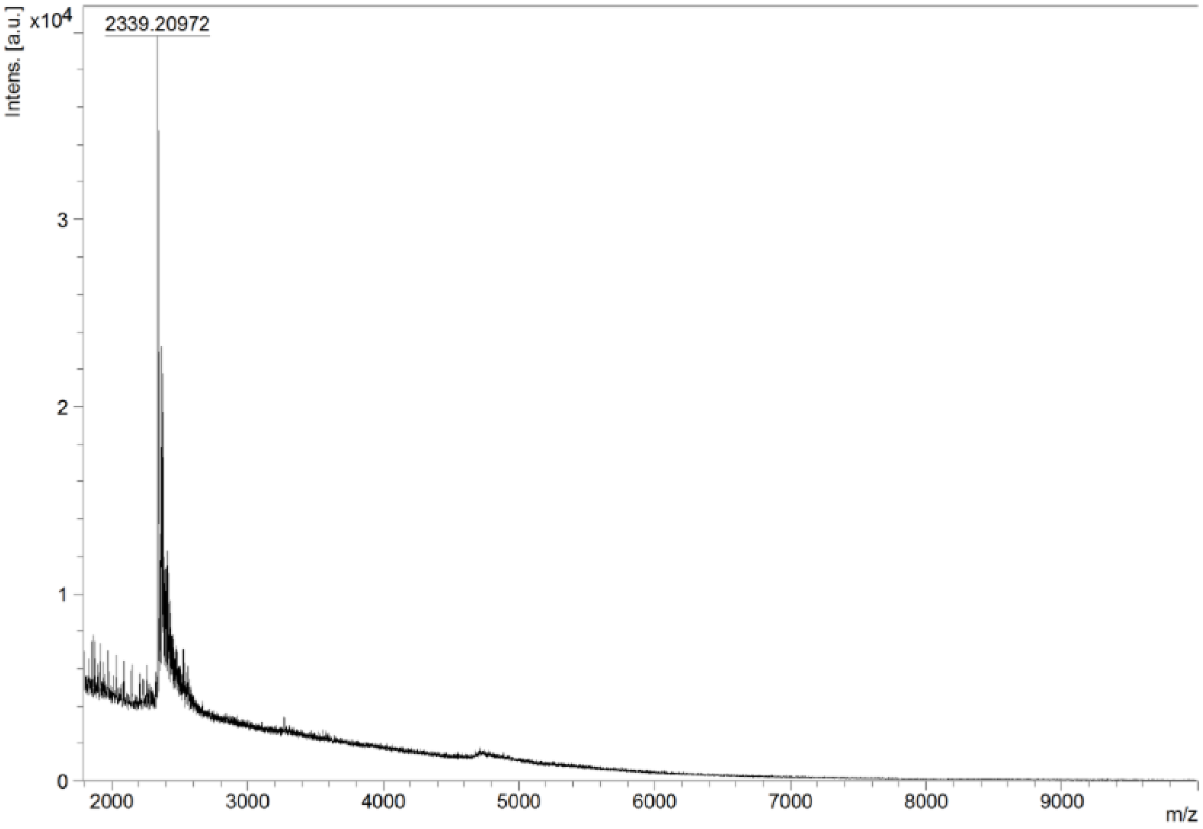

(d)

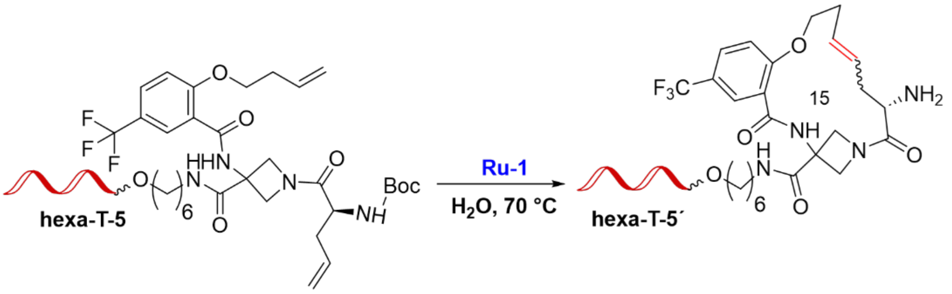

RP-HPLC chromatogram of hexa-T-5 diene after RCM in H$_2$O.

8.3 min and 8.7 min: **hexa-T-5´**, Boc-off. 11.7 min: **hexa-T-5**, Boc-off. **80% in situ yield**.

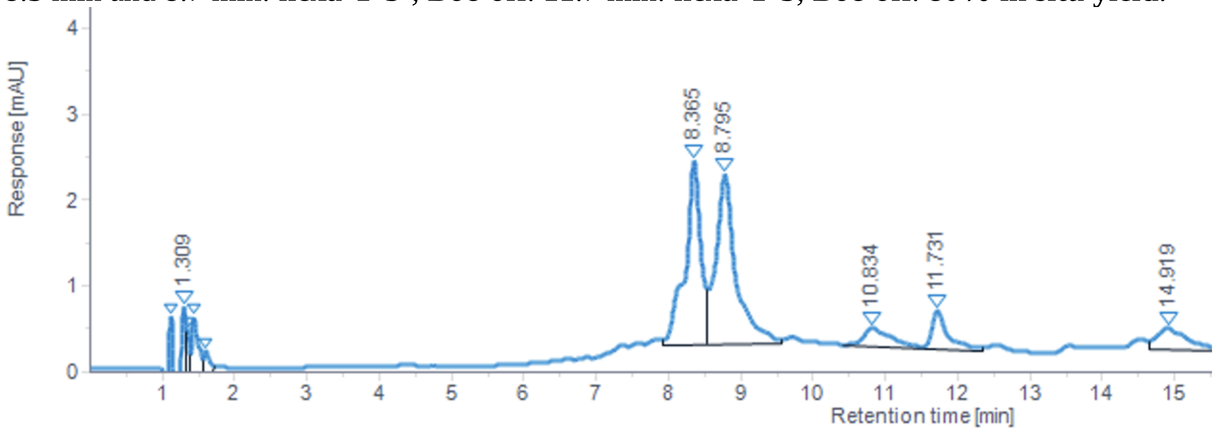

MALDI-MS: hexa-T-5´, Boc-off, calculated m/z 2304.

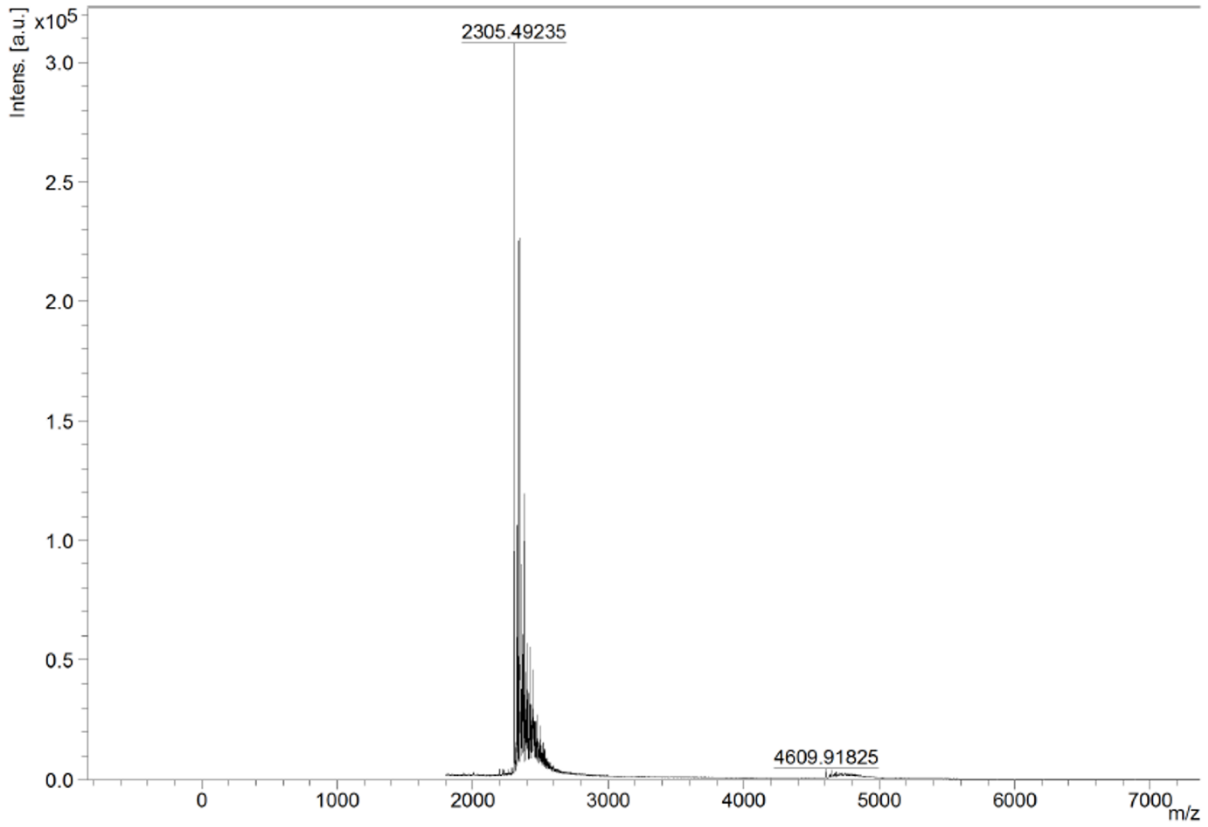

(e)

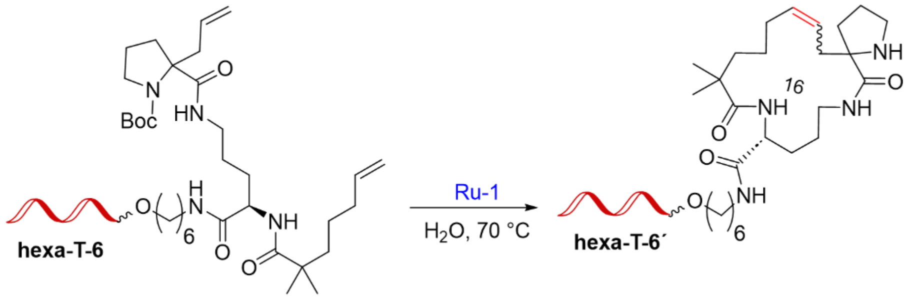

RP-HPLC chromatogram of hexa-T-6 diene after RCM in H_2O.
8.7 min: **hexa-T-6´**, Boc-off. **64% in situ yield**.

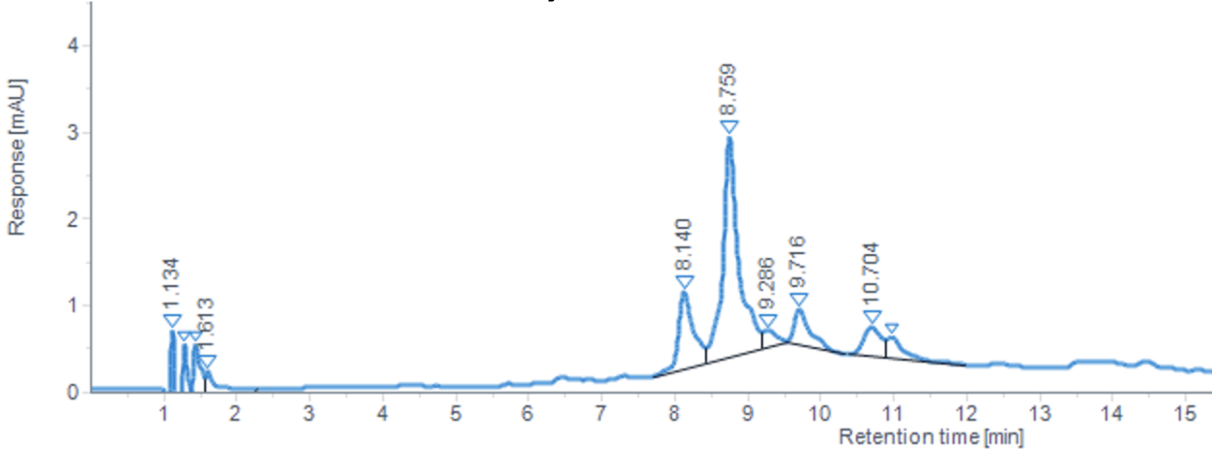

MALDI-MS: hexa-T-6´, Boc-off, calculated m/z 2338.

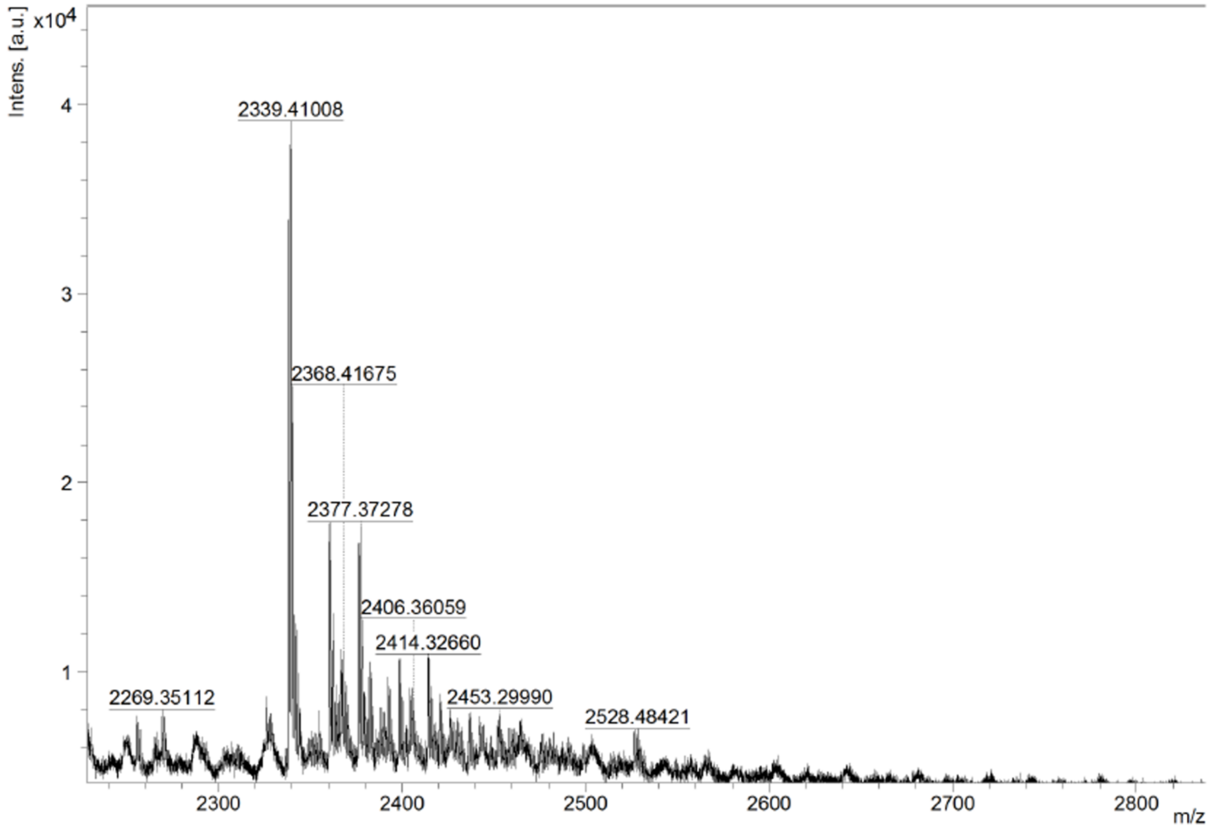

(f)

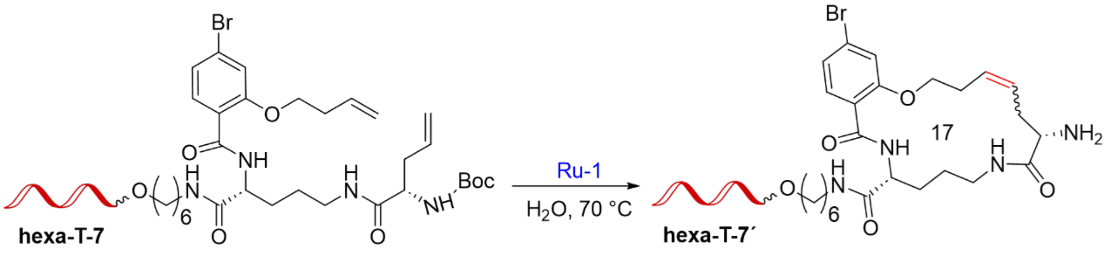

RP-HPLC chromatogram of hexa-T-7 diene after RCM in H_2O.
9.2 min and 9.6 min: **hexa-T-7´**, Boc-off. **95% in situ yield**.

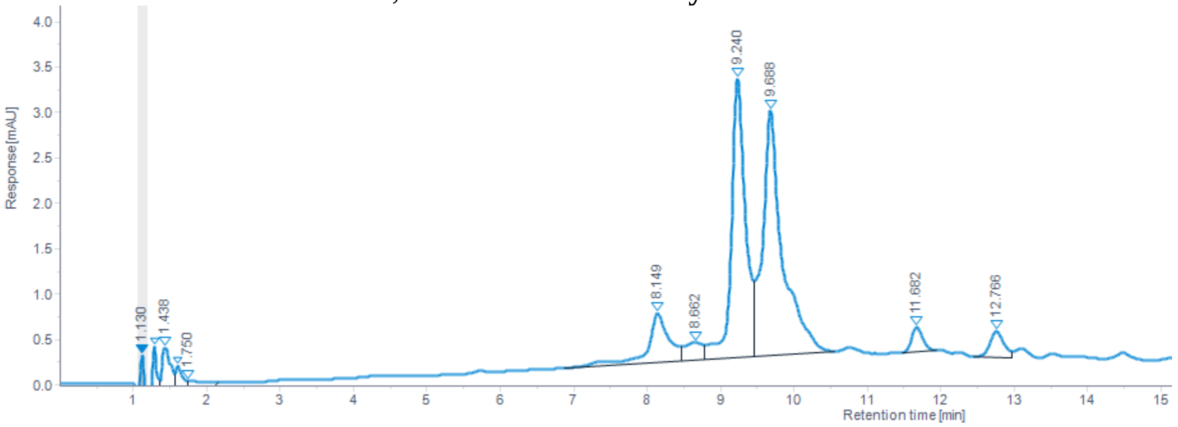

Full MALDI-MS: hexa-T-7´, Boc-off, calculated m/z 2379.

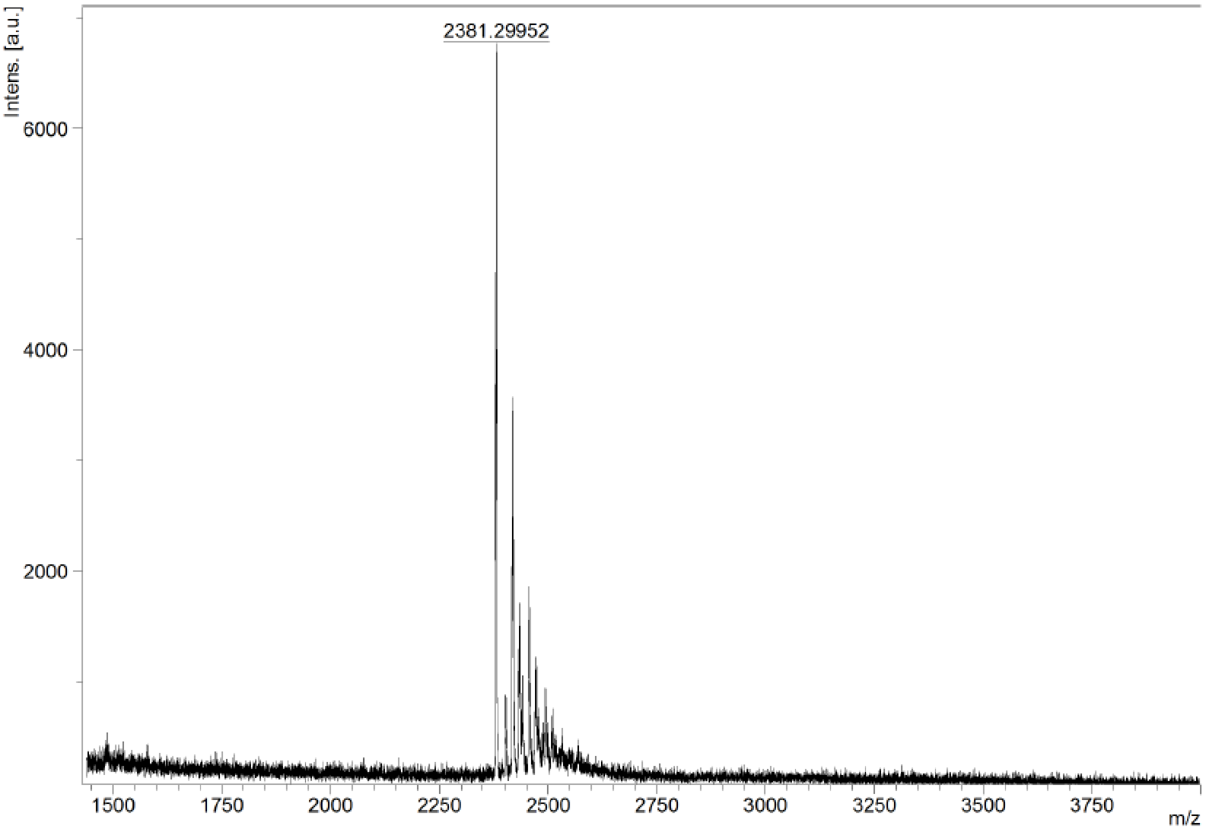

Zoomed-in MALDI-MS: hexa-T-7´, Boc-off, calculated m/z 2379.

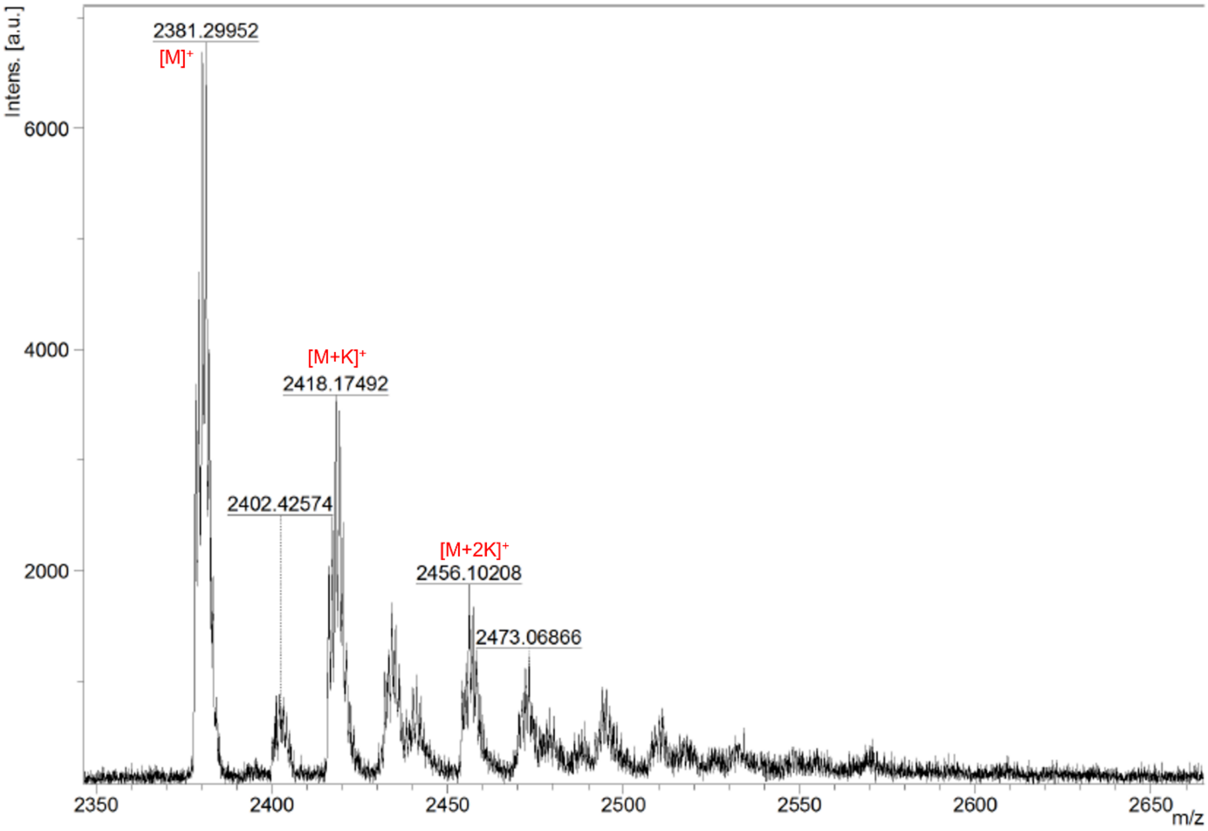

Figure S29. RP-HPLC and MALDI-MS spectra for substrate scope of hexa-T-tagged macrocycles (**hexa-T-2´-7´**).

13. RCM of a mixture of 4 hexa-T-tagged substrates to demonstrate DEL feasibility.

Synthesis of macrocycles **hexa-T-8´-11´** was performed as described in General Procedure 2. RCM conditions: 400 mM (40,000 equiv) NaCl, 400 mM (40,000 equiv) $NHEt_3Cl$, 10 equiv **Ru-1**, pH 5.0, 70 °C, 30 min.

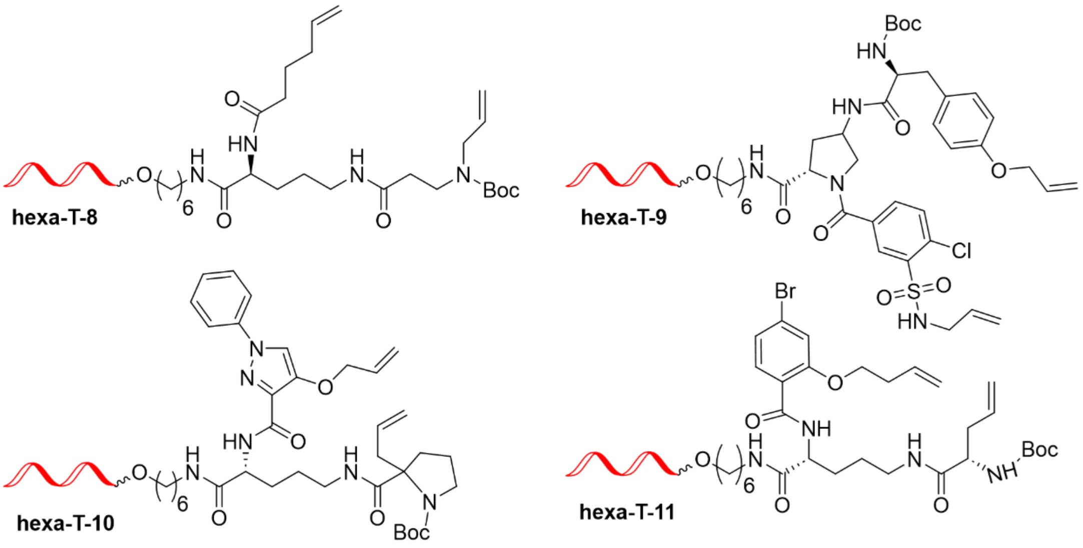

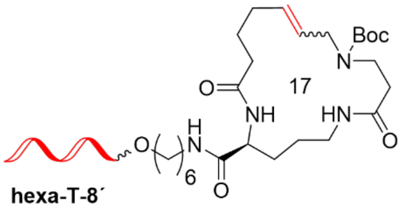

hexa-T-8´

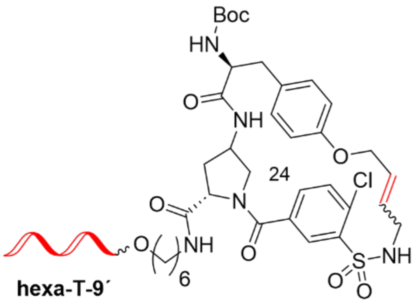

hexa-T-9´

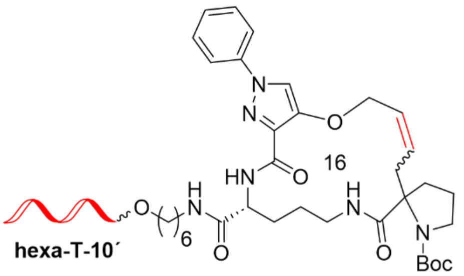

hexa-T-10´

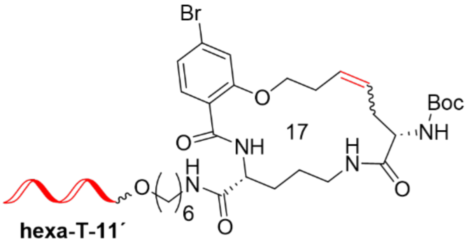

hexa-T-11´

RP-HPLC chromatogram of RCM reaction of mixture of four hexa-T-tagged diene substrates (hexa-T-8-11) to demonstrate DEL feasibility

10.3 and 10.4 min: **hexa-T-8´**, Boc-on, 11.2 min: **hexa-T-9´**, Boc-on, 12.8 and 13.2 min: **hexa-T-11´**, Boc-on, 13.0 min: **hexa-T-10´**, Boc-on.

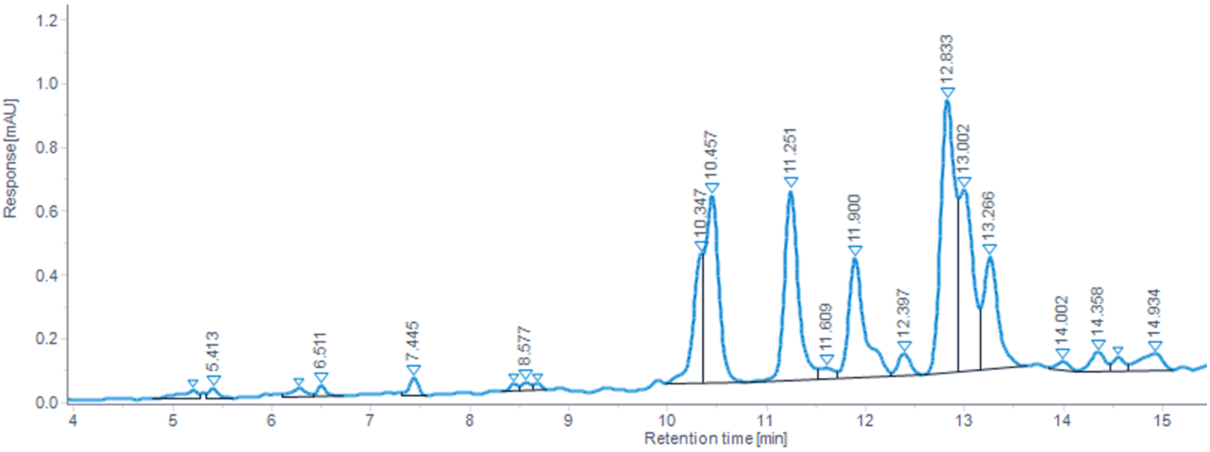

MALDI-MS: hexa-T-8´, Boc-on, calculated m/z 2335. **hexa-T-9´**, Boc-on, calculated m/z 2587. **hexa-T-10´**, Boc-on, calculated m/z 2492. **hexa-T-11´**, Boc-on, calculated m/z 2479.

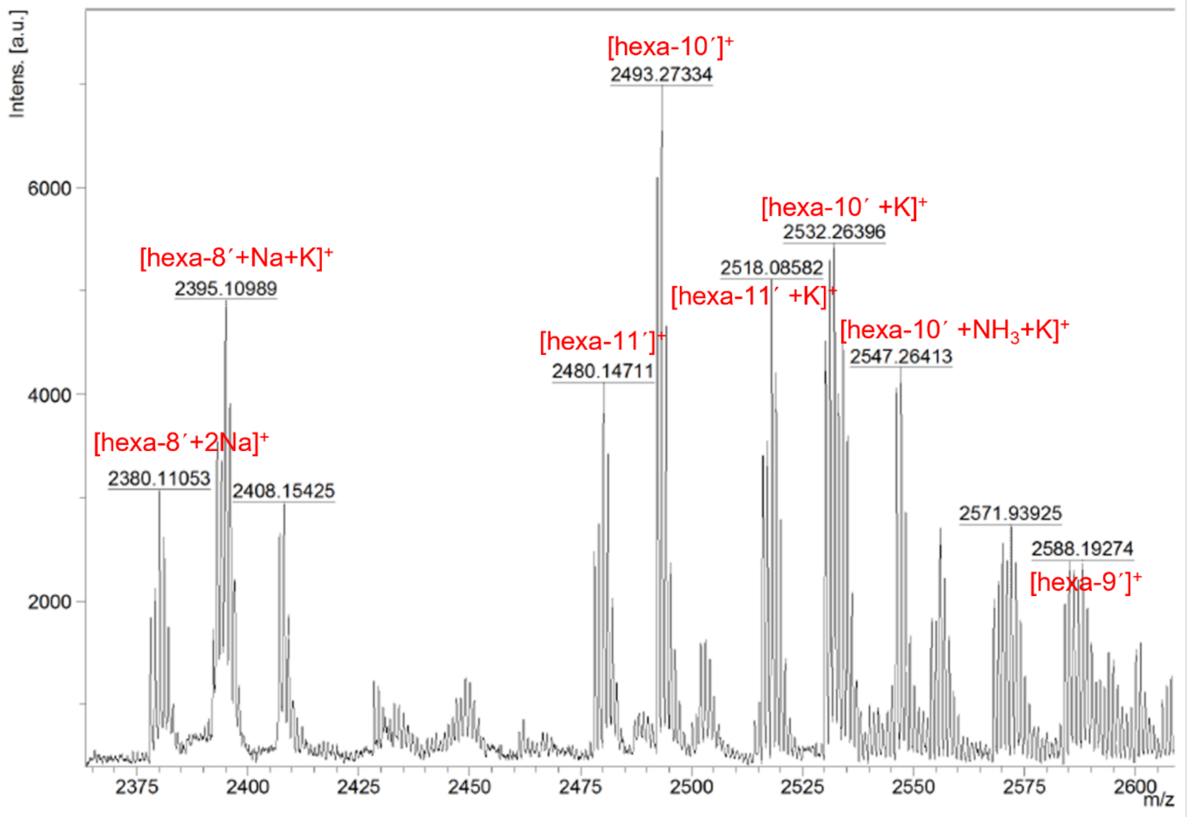

Figure S30. RP-HPLC and MALDI-MS spectra for RCM of mixture of 4 hexa-T-tagged substrates (**hexa-T-8-11**).

14. Optimizing conditions for RCM of csDNA-tagged dienes (7DeATC-1) in H_2O.

RCM reactions were performed as described in General Procedure 2

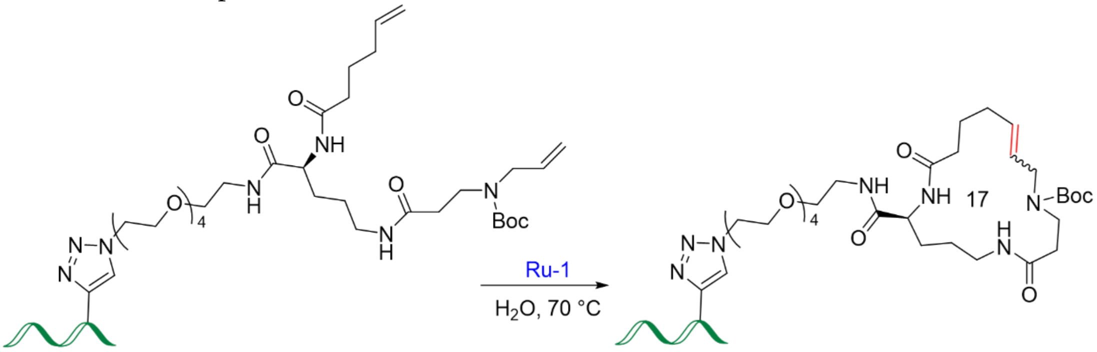

Table S16: Optimizing RCM reaction conditions with **7DeATC-1** in H_2O.

Entry	Additives (equiv)	pH	Time (min)	In situ yield (%)	Isolated yield (%)	DNA recovery (total DNA %)
1	40,000 NaCl 40,000 $NHEt_3Cl$	2	30	66	20	31
2	40,000 NaCl 80,000 $NHEt_3Cl$	2	30	62	33	54
3	80,000 NaCl 40,000 $NHEt_3Cl$	2	30	74	31	43
4	80,000 NaCl 80,000 $NHEt_3Cl$	2	30	46	27	59
5	100k NaCl 100k $NHEt_3Cl$	2	30	60	41	69
6	150k NaCl 150k $NHEt_3Cl$	2	30	41	28	69
**7**	**80,000 NaCl 80,000 $NHEt_3Cl$**	**5**	**30**	**62**	**50**	**81**
8	80,000 NaCl 80,000 $NHEt_3Cl$	5	60	92	38	41

RP-HPLC chromatograms for optimizing RCM reaction conditions with csDNA-tagged 7DeATC-1 in H_2O.

Entry 1: 5.1 min: **7DeATC-1´**, Boc-off, 6.3 min: **7DeATC-1**, Boc-off. 8.9 min: **7DeATC-1´**, Boc-on, 10.5 min: **7DeATC-1**, Boc-on.

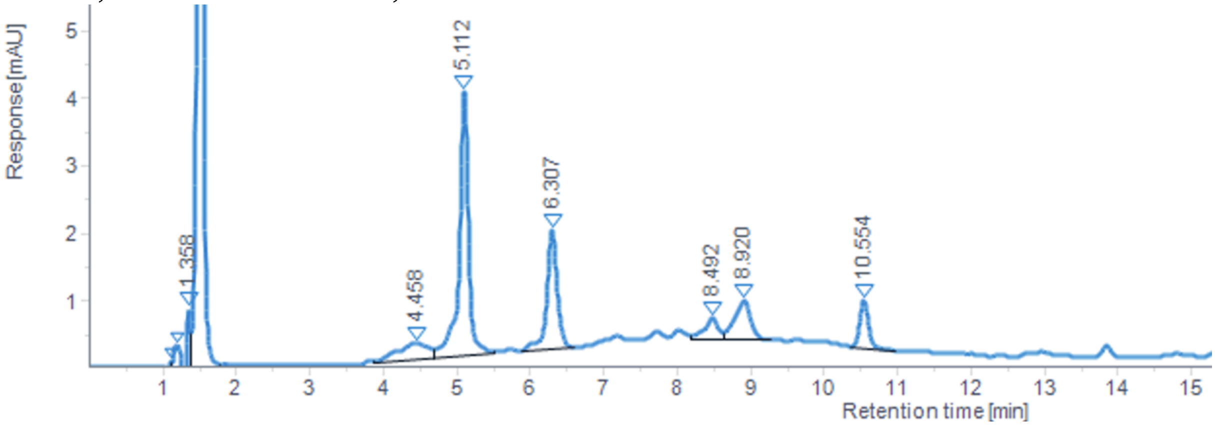

Entry 2: 5.1 min: **7DeATC-1´**, Boc-off, 6.3 min: **7DeATC-1**, Boc-off. 8.9 min: **7DeATC-1´**, Boc-on, 10.5 min: **7DeATC-1**, Boc-on.

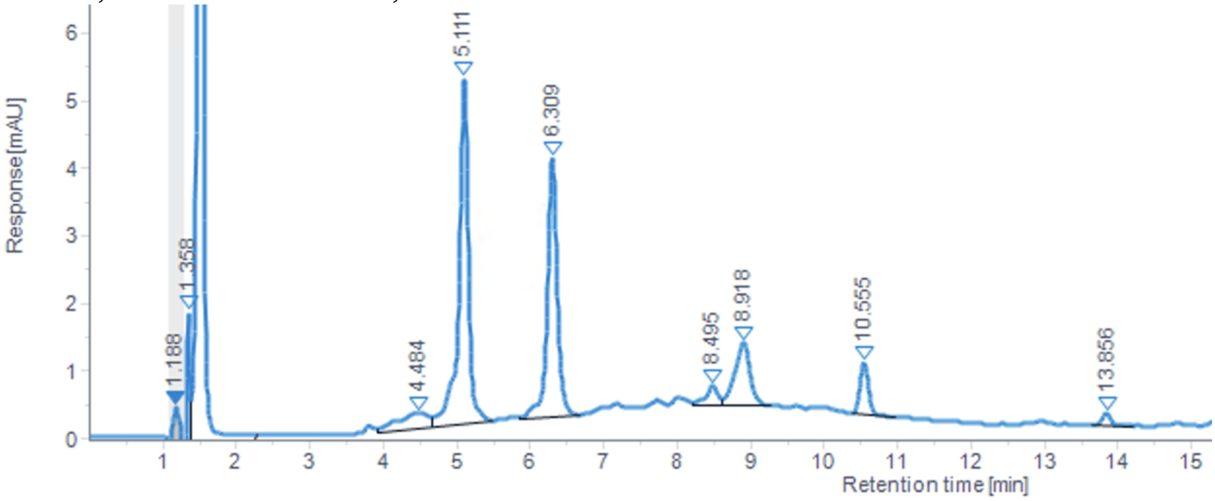

Entry 3: 5.1 min: **7DeATC-1´**, Boc-off, 6.3 min: **7DeATC-1**, Boc-off. 8.9 min: **7DeATC-1´**, Boc-on, 10.5 min: **7DeATC-1**, Boc-on.

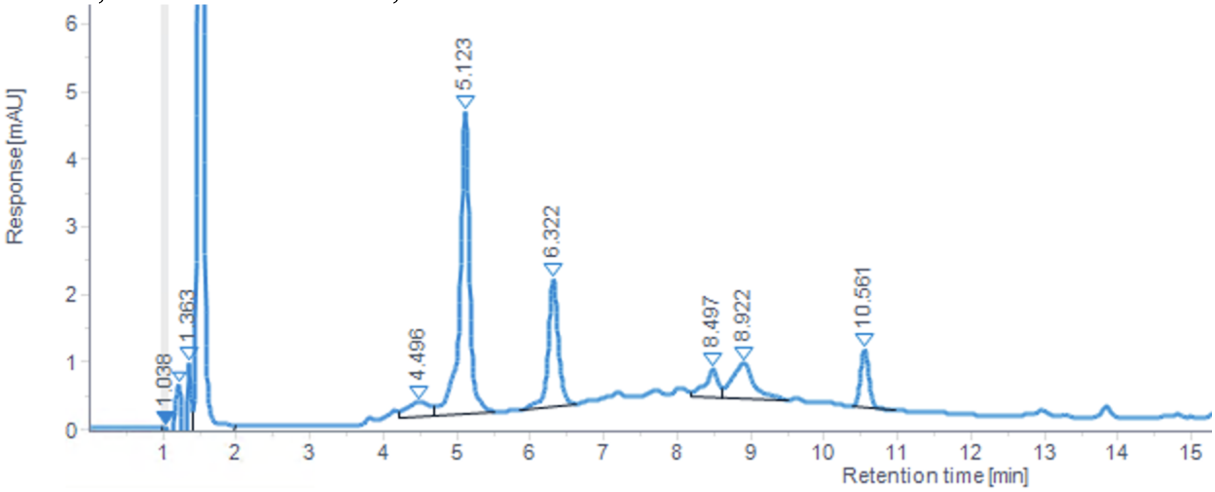

Entry 4: 5.1 min: **7DeATC-1´**, Boc-off, 6.3 min: **7DeATC-1**, Boc-off. 8.9 min: **7DeATC-1´**, Boc-on, 10.5 min: **7DeATC-1**, Boc-on.

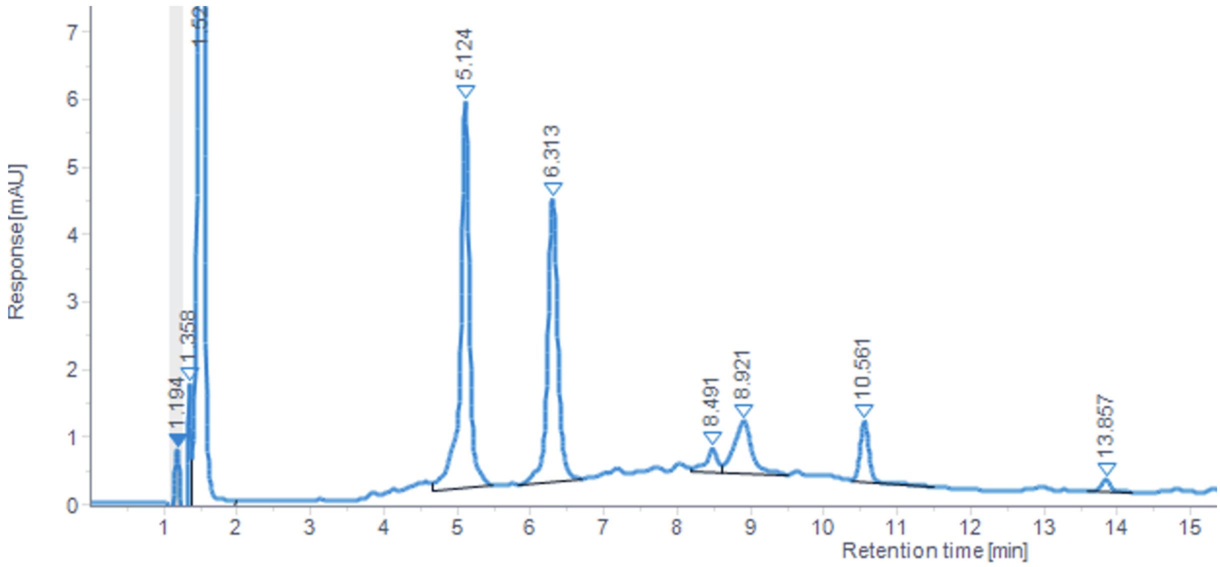

Entry 5: 5.3 min: **7DeATC-1´**, Boc-off, 6.5 min: **7DeATC-1**, Boc-off. 9.1 min: **7DeATC-1´**, Boc-on, 9.9 min: **7DeATC-1**, Boc-on.

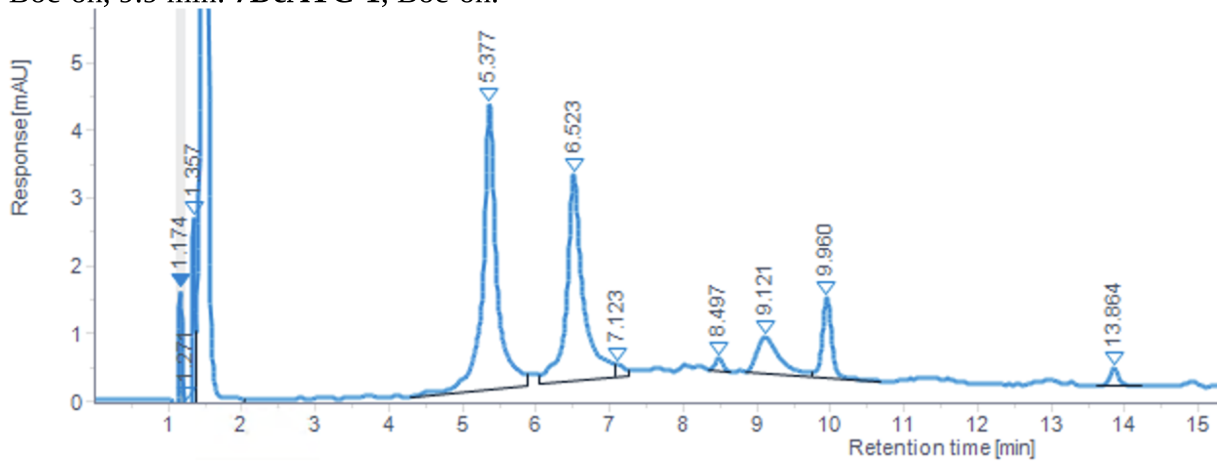

Entry 6: 5.4 min: **7DeATC-1´**, Boc-off, 6.5 min: **7DeATC-1**, Boc-off. 9.1 min: **7DeATC-1´**, Boc-on, 9.9 min: **7DeATC-1**, Boc-on.

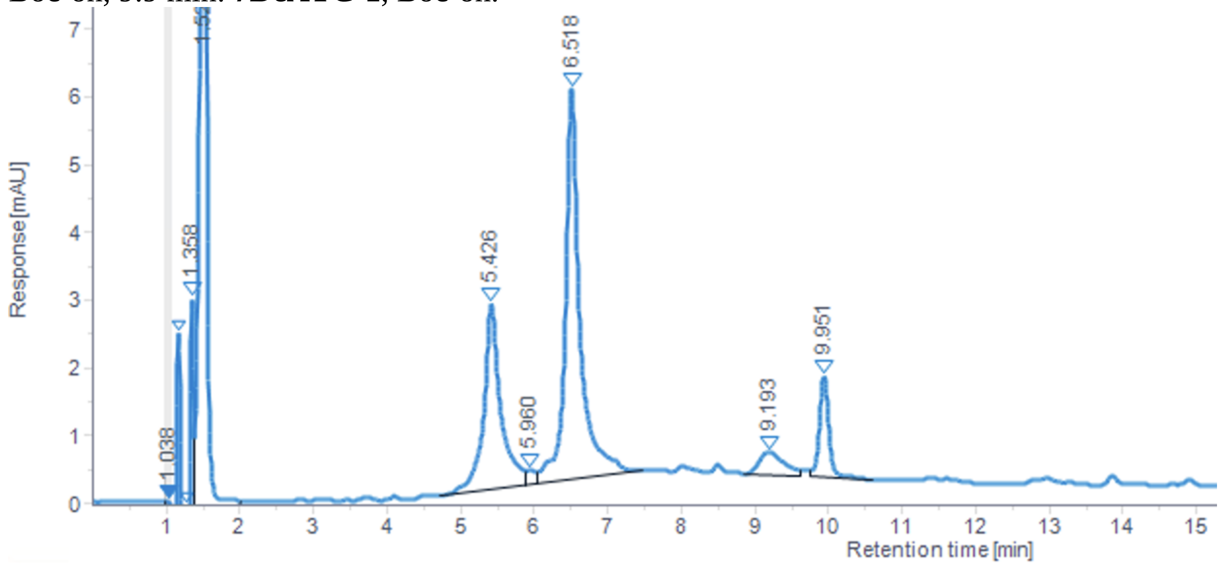

Entry 7: 5.5 min: **7DeATC-1´**, Boc-off, 6.5 min: **7DeATC-1**, Boc-off. 8.9 min: **7DeATC-1´**, Boc-on, 11.1 min: **7DeATC-1**, Boc-on.

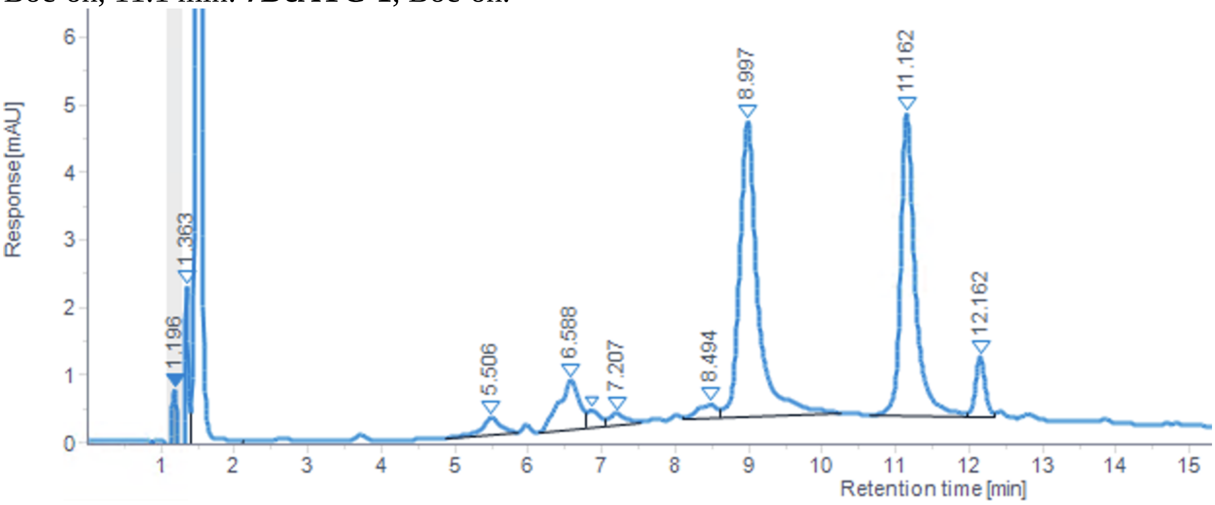

Entry 8: 5.4 min: **7DeATC-1´**, Boc-off, 6.6 min: **7DeATC-1**, Boc-off. 9.0 min: **7DeATC-1´**, Boc-on, 11.2 min: **7DeATC-1**, Boc-on.

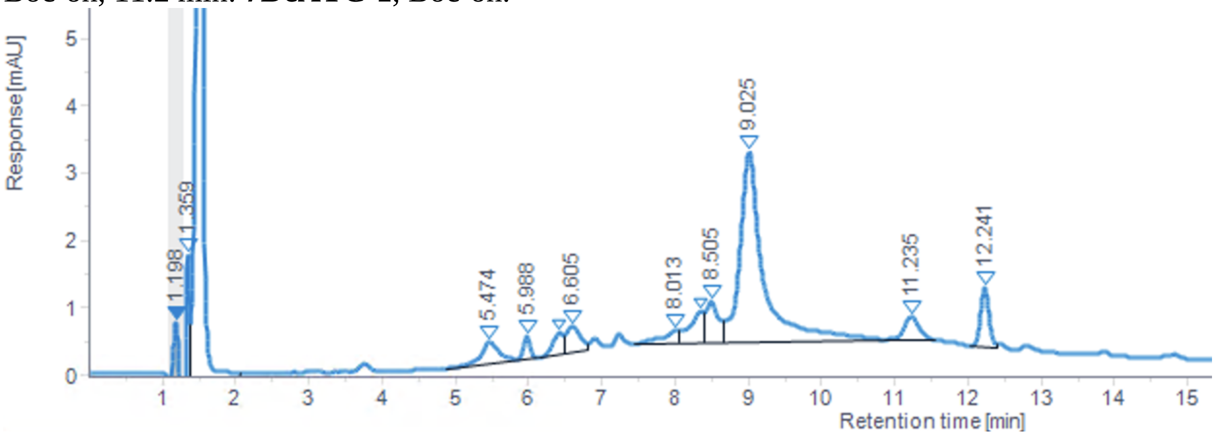

MALDI-MS: 7DeATC-1´, Boc-on, calculated m/z 5353, **7DeATC-1**, Boc-on, calculated m/z 5381.

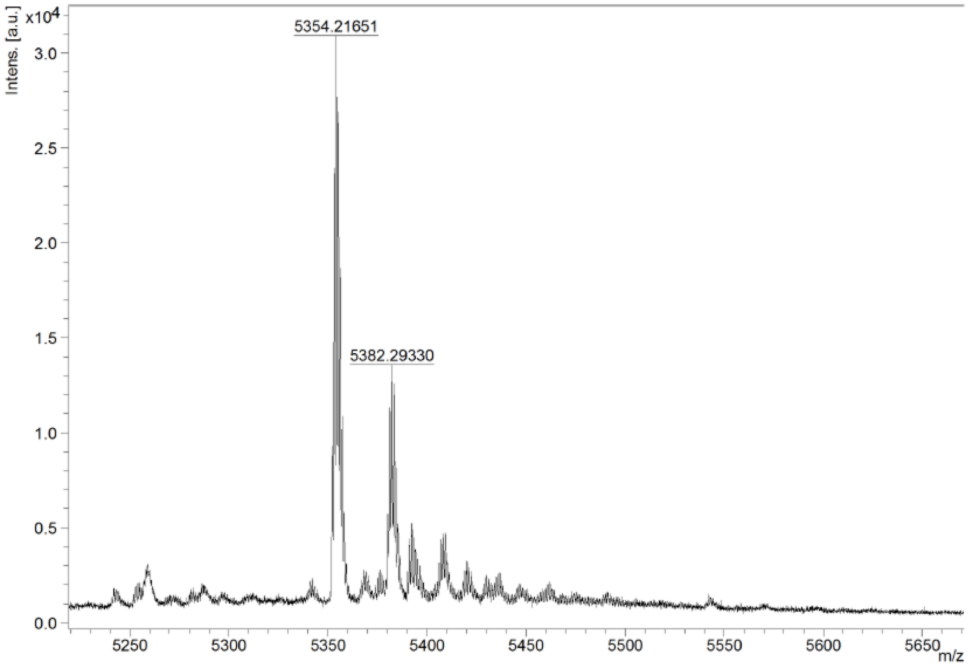

Figure S31. RP-HPLC traces for **Table S16** (entry 1-8), and MALDI-MS spectra for optimizing RCM reaction conditions with csDNA-tagged substrate **7DeATC-1** in H_2O.

15. Optimizing conditions for RCM of DNA-tagged substrates (ATGC-1) in H_2O

RCM reactions were performed as described in General Procedure 2 at pH 5.0.

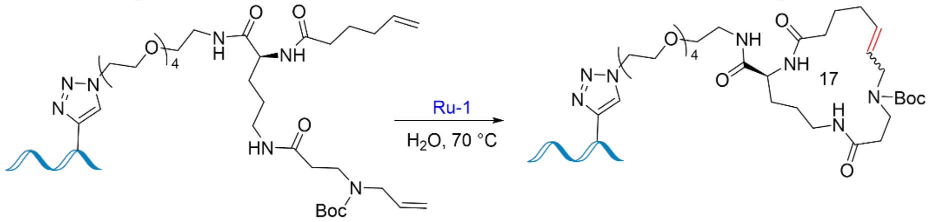

Table S17. Optimizing reaction conditions for RCM of **ATGC-1** in H_2O.

Entry	Additives (equiv)	T (°C)	Time (min)	In situ yield (%)	Isolated yield (%)	DNA recovery (total DNA %)
1	40,000 NaCl<br>40,000 $NHEt_3Cl$	70	30	21	10	46
2	40,000 NaCl<br>80,000 $NHEt_3Cl$	70	30	31	23	75
3	80,000 NaCl<br>40,000 $NHEt_3Cl$	70	30	50	24	48
4	80,000 NaCl<br>80,000 $NHEt_3Cl$	70	30	50	38	76
5	100k NaCl<br>100k $NHEt_3Cl$	70	30	61	45	73
**6**	**150k NaCl<br>150k $NHEt_3Cl$**	**70**	**30**	**58**	**45**	**78**
7	100k NaCl<br>100k $NHEt_3Cl$	70	60	75	30	40
8	80,000 NaCl<br>80,000 $NHEt_3Cl$	60	30	<5	<5	95
9	80,000 NaCl<br>80,000 $NHEt_3Cl$	60	60	<5	<5	83
10	80,000 NaCl<br>80,000 $NHEt_3Cl$	50	30	<5	<5	>95
11	80,000 NaCl<br>80,000 $NHEt_3Cl$	50	60	<5	<5	86
12	80,000 NaCl<br>80,000 $NHEt_3Cl$	40	30	<5	<5	>95
13	80,000 NaCl<br>80,000 $NHEt_3Cl$	40	60	<5	<5	94
14	80,000 NaCl<br>80,000 $NHEt_3Cl$	25	30	<5	<5	>95
15	80,000 NaCl<br>80,000 $NHEt_3Cl$	25	60	<5	<5	92
16	80,000 NaCl<br>80,000 $NHEt_3Cl$ Phosphate	70	30	<5	<5	88
17	80,000 NaCl<br>80,000 $NHEt_3Cl$ HEPES	70	30	5	5	>95
18	80,000 NaCl<br>80,000 $NHEt_3Cl$ MES	70	30	5	5	90

RP-HPLC chromatograms for RCM of DNA-tagged substrate (ATGC-1) in H_2O.

Entry 1: 8.8 min: **ATGC-1´**, Boc-on, 11.0 min: **ATGC-1**, Boc-on.

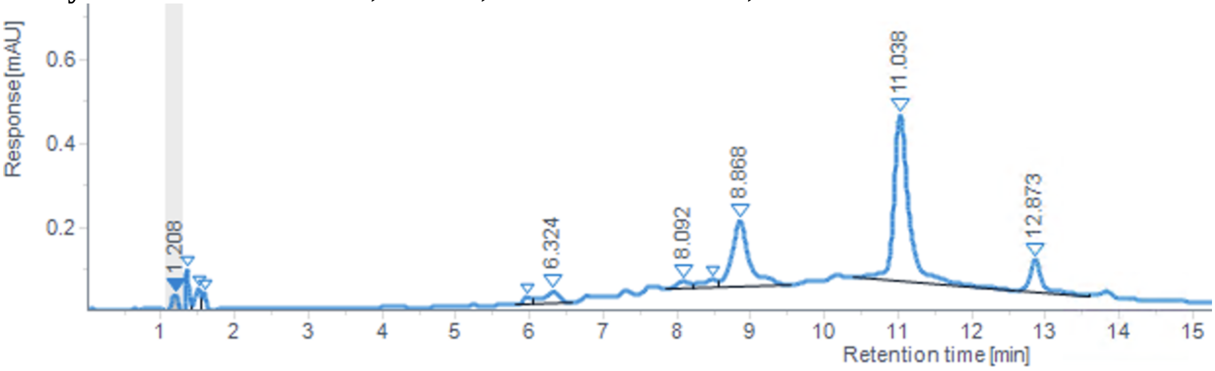

Entry 2: 8.8 min: **ATGC-1´**, Boc-on, 11.0 min: **ATGC-1**, Boc-on.

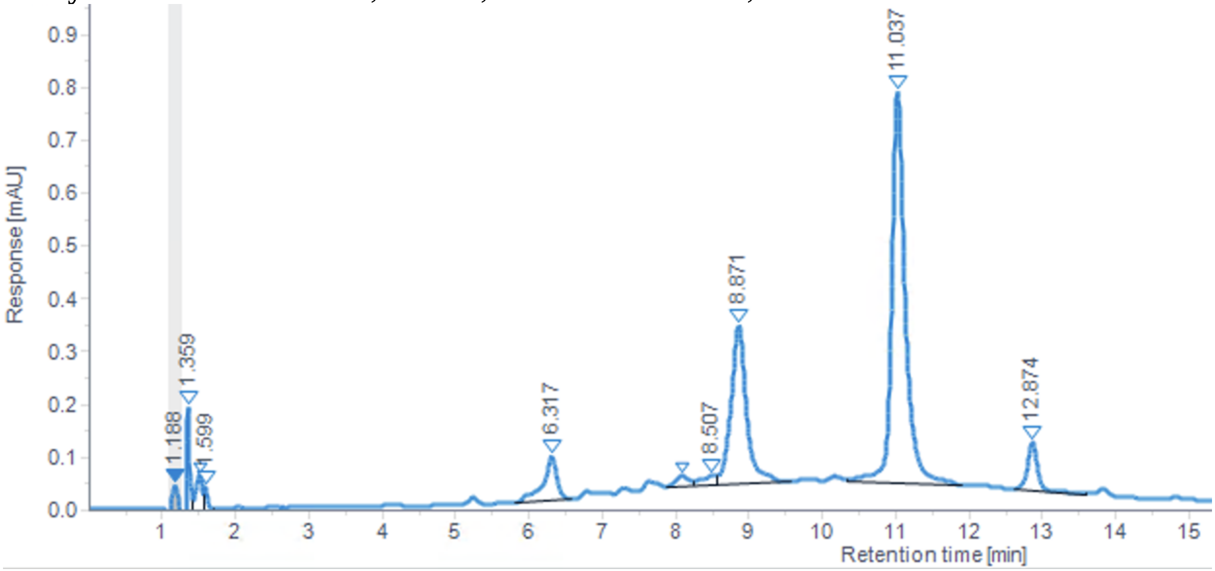

Entry 3: 8.8 min: **ATGC-1´**, Boc-on, 11.0 min: **ATGC-1**, Boc-on.

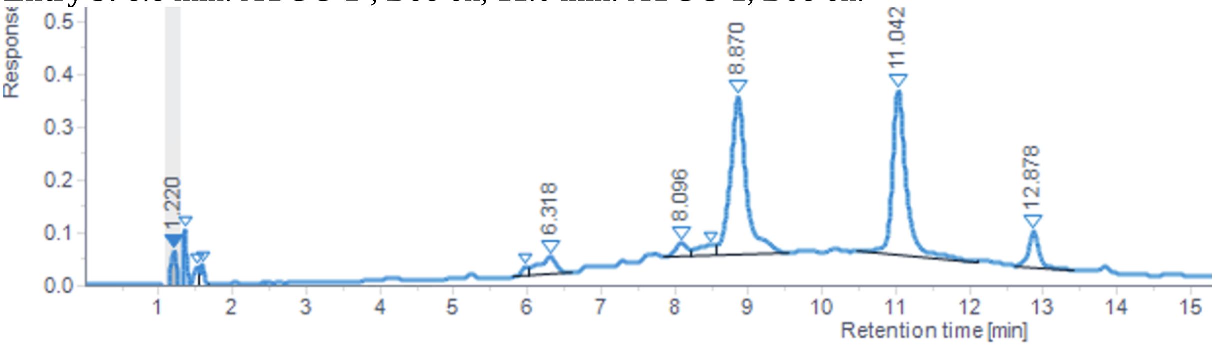

Entry 4: 8.8 min: **ATGC-1´**, Boc-on, 11.0 min: **ATGC-1**, Boc-on.

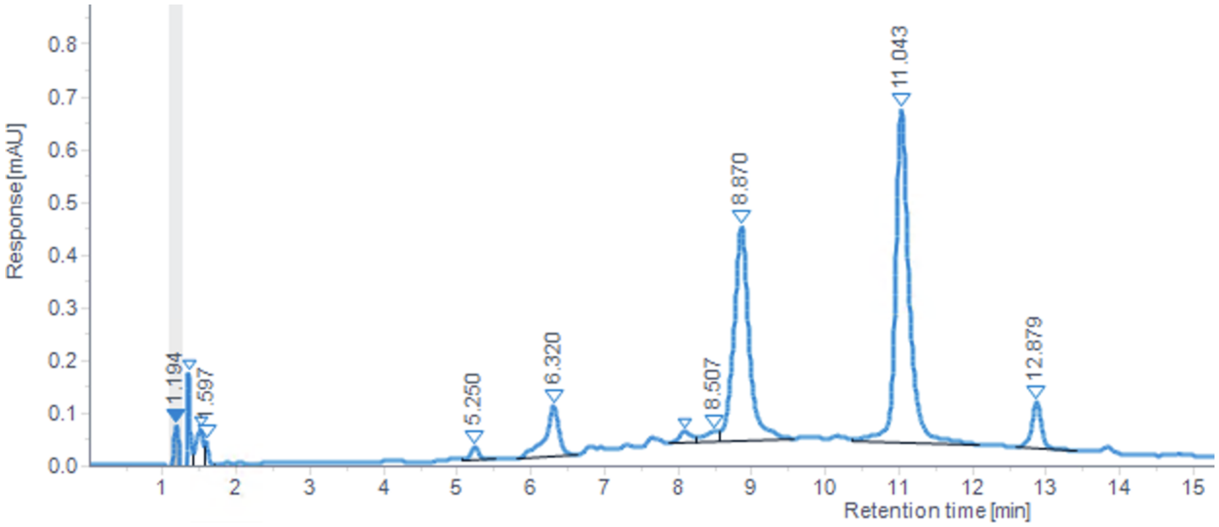

Entry 5: 8.9 min: **ATGC-1´**, Boc-on, 11.0 min: **ATGC-1**, Boc-on.

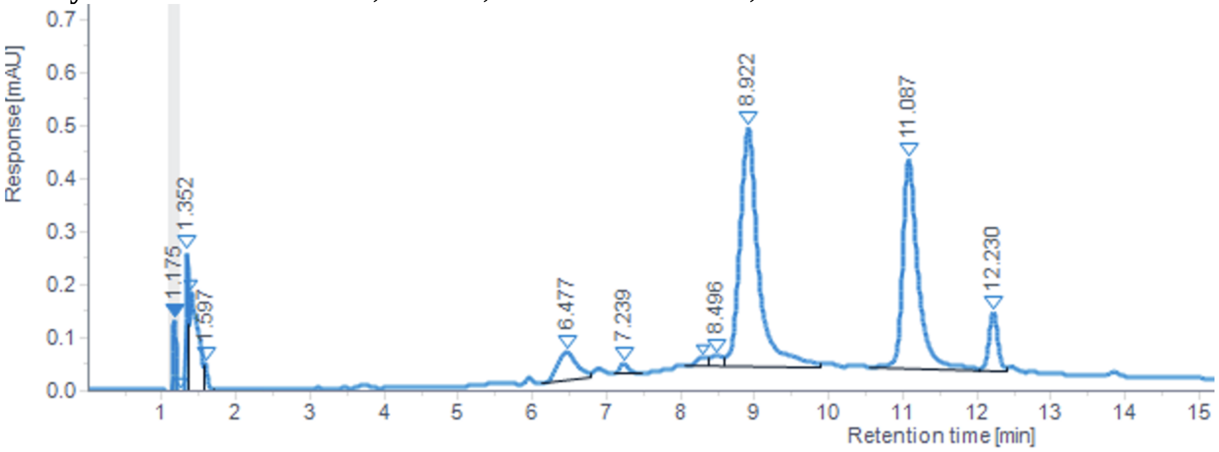

Entry 6: 8.9 min: **ATGC-1´**, Boc-on, 11.1 min: **ATGC-1**, Boc-on.

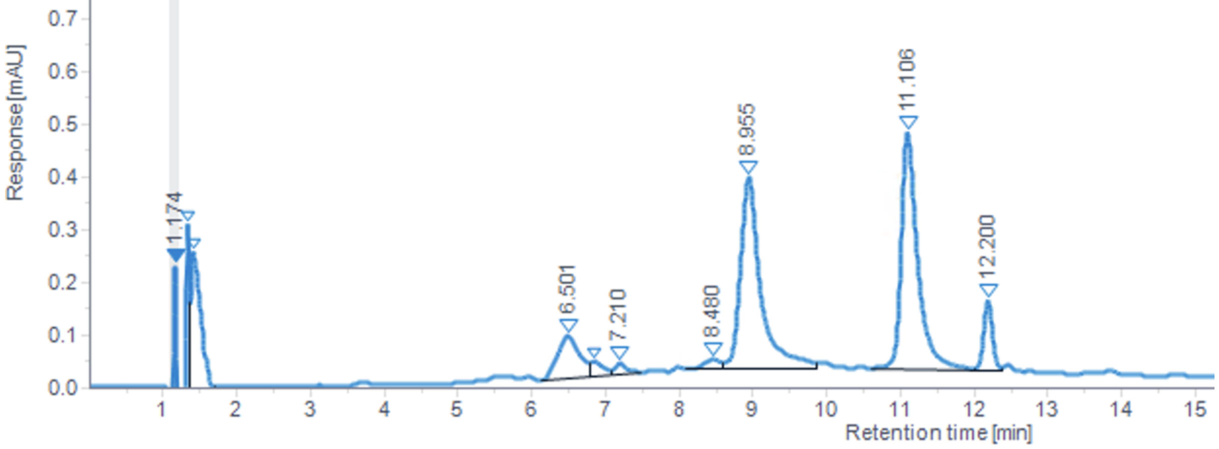

Entry 7: 9.0 min: **ATGC-1´**, Boc-on, 11.2 min: **ATGC-1**, Boc-on.

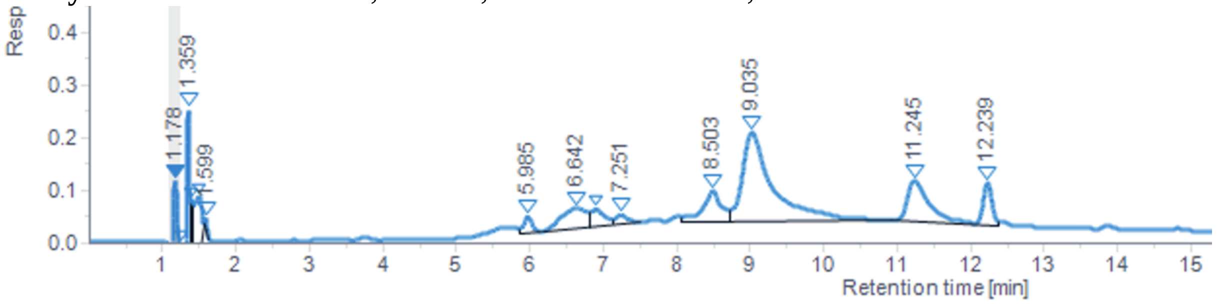

Entry 8: 11.1 min: **ATGC-1**, Boc-on.

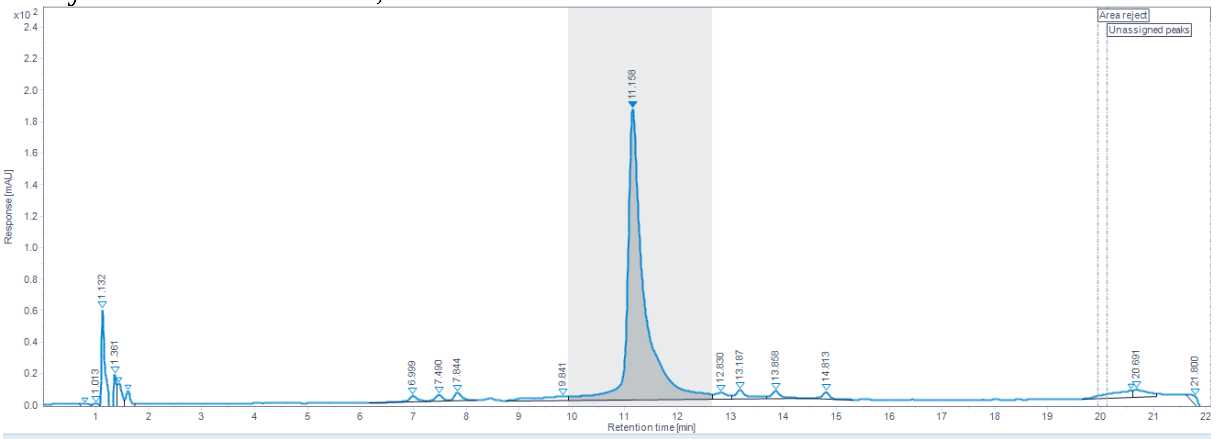

Injection Results

Peaks | Summary

#	Name	Signal description	RT (min)	Area (mAU·s)	Area%	Height (mAU)	Height%	Amount	Concentration	Start time (min)	End time (min)
7		DAD1A,Sig=260,4 Ref=off	6.999	49.593	0.747	3.850	0.85			6.180	7.257
8		DAD1A,Sig=260,4 Ref=off	7.490	45.337	0.683	4.182	0.92			7.257	7.691
9		DAD1A,Sig=260,4 Ref=off	7.844	55.861	0.842	5.436	1.20			7.691	8.207
10		DAD1A,Sig=260,4 Ref=off	9.841	111.644	1.682	2.587	0.57			8.767	9.939
11		DAD1A,Sig=260,4 Ref=off	11.158	3964.717	59.735	184.463	40.60			9.939	12.663

Entry 9: 11.1 min: **ATGC-1**, Boc-on.

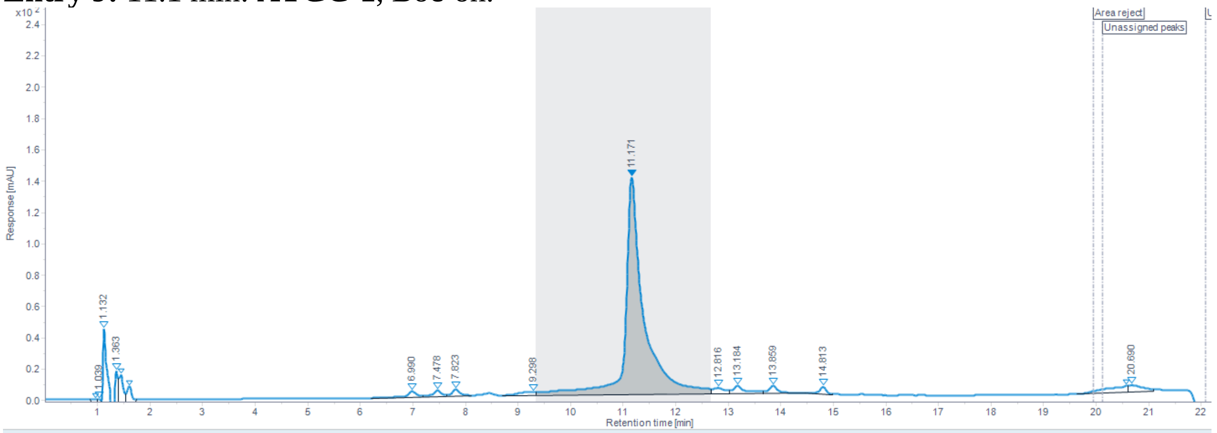

Injection Results

Peaks | Summary

#	Name	Signal description	RT (min)	Area (mAU·s)	Area%	Height (mAU)	Height%	Amount	Concentration	Start time (min)	End time (min)
7		DAD1A,Sig=260,4 Ref=off	6.990	43.637	0.764	3.715	0.98			6.207	7.206
8		DAD1A,Sig=260,4 Ref=off	7.478	40.591	0.711	3.779	0.99			7.206	7.658
9		DAD1A,Sig=260,4 Ref=off	7.823	40.469	0.709	4.137	1.09			7.658	8.115
10		DAD1A,Sig=260,4 Ref=off	9.298	42.605	0.746	1.827	0.48			8.714	9.347
11		DAD1A,Sig=260,4 Ref=off	11.171	3401.690	59.573	138.598	36.45			9.347	12.682

Entry 10: 11.1 min: **ATGC-1**, Boc-on.

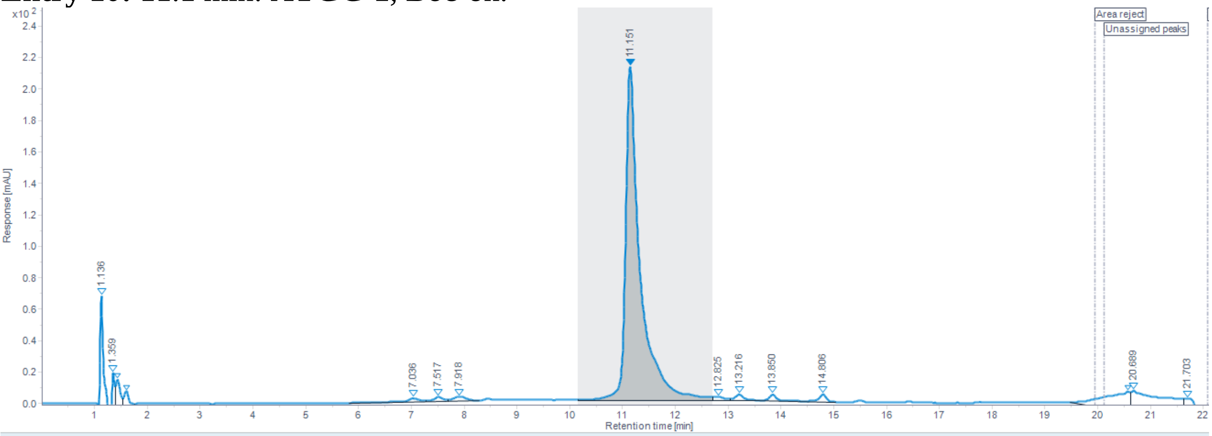

Injection Results

Peaks | Summary

#	Name	Signal description	RT (min)	Area (mAU·s)	Area%	Height (mAU)	Height%	Amount	Concentration	Start time (min)	End time (min)
5		DAD1A,Sig=260,4 Ref=off	7.036	37.498	0.516	2.196	0.47			5.836	7.295
6		DAD1A,Sig=260,4 Ref=off	7.517	34.159	0.470	2.744	0.59			7.295	7.702
7		DAD1A,Sig=260,4 Ref=off	7.918	44.658	0.614	2.752	0.59			7.702	8.300
8		DAD1A,Sig=260,4 Ref=off	11.151	4079.557	56.119	212.090	45.68			10.167	12.720

Entry 11: 11.1 min: **ATGC-1**, Boc-on.

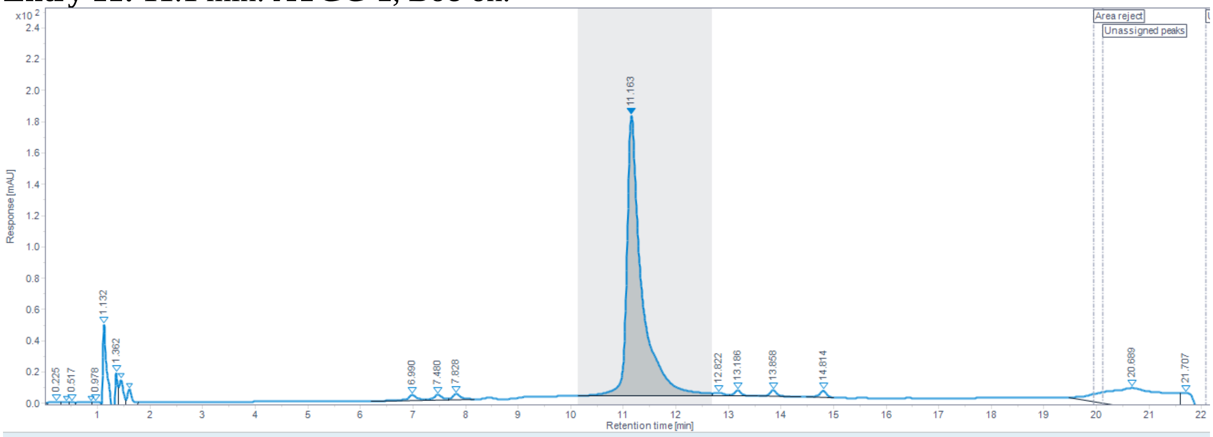

Injection Results

Peaks | Summary

#	Name	Signal description	RT (min)	Area (mAU·s)	Area%	Height (mAU)	Height%	Amount	Concentration	Start time (min)	End time (min)
9		DAD1A,Sig=260,4 Ref=off	1.615	160.141	2.037	20.413	4.02			1.548	1.785
10		DAD1A,Sig=260,4 Ref=off	6.990	51.856	0.660	3.640	0.72			6.210	7.253
11		DAD1A,Sig=260,4 Ref=off	7.480	43.329	0.551	3.690	0.73			7.253	7.678
12		DAD1A,Sig=260,4 Ref=off	7.828	46.439	0.591	3.961	0.78			7.678	8.179
13		DAD1A,Sig=260,4 Ref=off	11.163	3541.152	45.043	179.135	35.24			10.150	12.700

Entry 12: 11.1 min: **ATGC-1**, Boc-on.

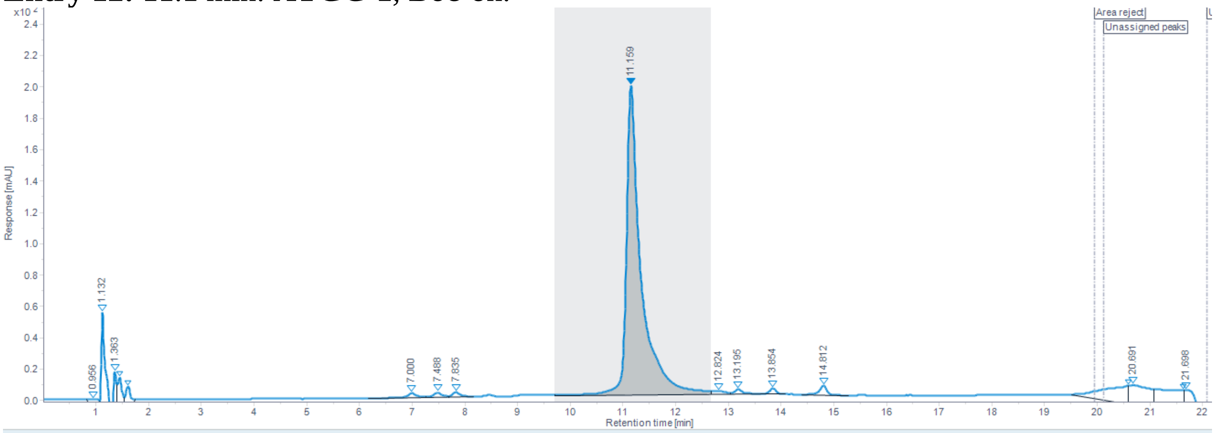

Injection Results

Peaks Summary

#	Name	Signal description	RT (min)	Area (mAU·s)	Area%	Height (mAU)	Height%	Amount	Concentration	Start time (min)	End time (min)
7		DAD1A,Sig=260,4 Ref=off	7.488	35.856	0.491	3.053	0.64			7.272	7.699
8		DAD1A,Sig=260,4 Ref=off	7.835	31.265	0.428	2.955	0.62			7.699	8.172
9		DAD1A,Sig=260,4 Ref=off	11.159	3883.745	53.199	197.090	41.45			9.722	12.679

Entry 13: 11.1 min: **ATGC-1**, Boc-on.

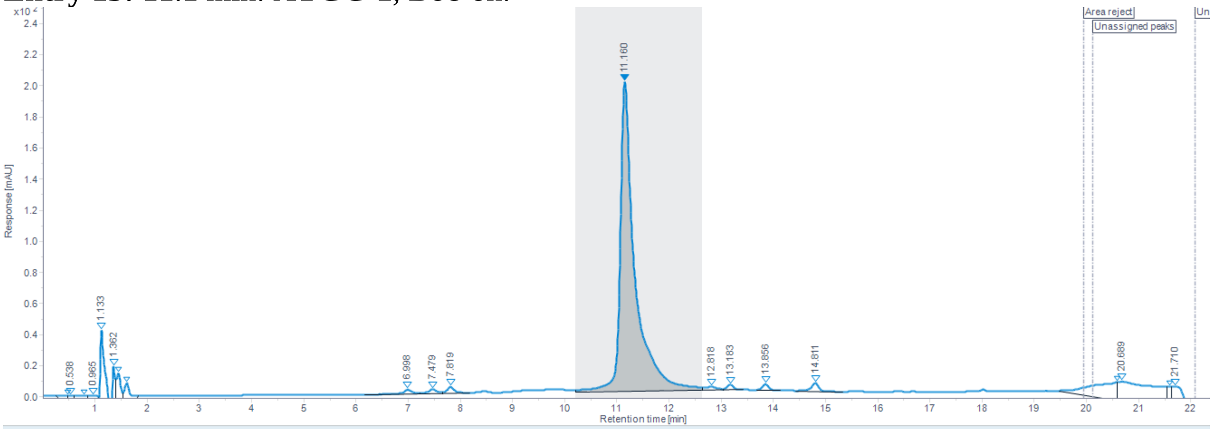

Injection Results

Peaks Summary

#	Name	Signal description	RT (min)	Area (mAU·s)	Area%	Height (mAU)	Height%	Amount	Concentration	Start time (min)	End time (min)
7		DAD1A,Sig=260,4 Ref=off	1.457	255.164	3.205	38.374	7.29			1.408	1.542
8		DAD1A,Sig=260,4 Ref=off	1.617	205.803	2.585	22.093	4.20			1.542	1.839
9		DAD1A,Sig=260,4 Ref=off	6.998	40.074	0.503	2.820	0.54			6.177	7.237
10		DAD1A,Sig=260,4 Ref=off	7.479	36.383	0.457	2.963	0.56			7.237	7.666
11		DAD1A,Sig=260,4 Ref=off	7.819	44.967	0.565	4.218	0.80			7.666	8.184
12		DAD1A,Sig=260,4 Ref=off	11.160	3896.878	48.943	198.893	37.80			10.225	12.643

Entry 14: 11.1 min: **ATGC-1**, Boc-on.

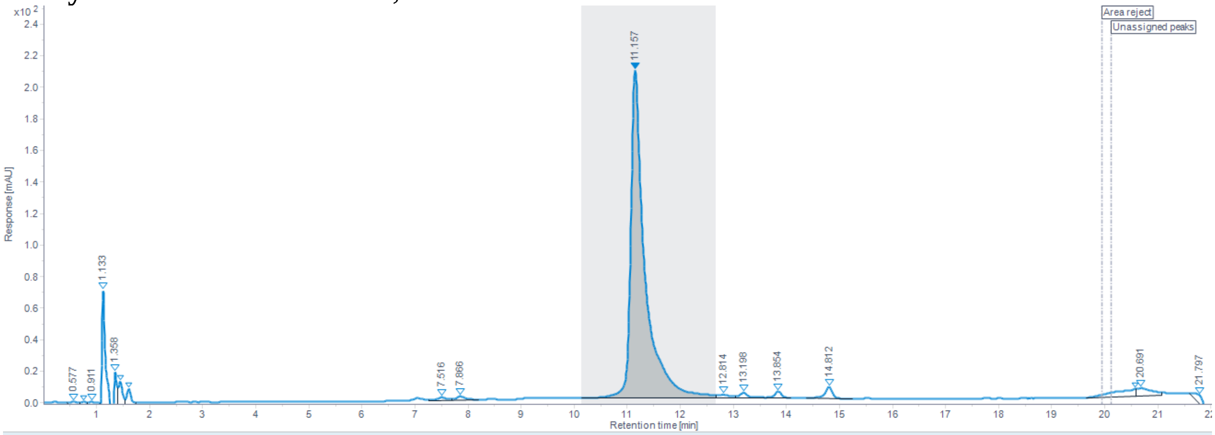

Injection Results

Peaks | Summary

#	Name	Signal description	RT (min)	Area (mAU·s)	Area%	Height (mAU)	Height%	Amount	Concentration	Start time (min)	End time (min)
7		DAD1A,Sig=260,4 Ref=off	1.616	130.067	2.002	18.169	3.72			1.548	1.750
8		DAD1A,Sig=260,4 Ref=off	7.516	24.066	0.370	1.792	0.37			7.271	7.702
9		DAD1A,Sig=260,4 Ref=off	7.866	26.413	0.406	2.176	0.45			7.702	8.199
10		DAD1A,Sig=260,4 Ref=off	11.157	3869.347	59.545	207.696	42.54			10.150	12.681

Entry 15: 11.1 min: **ATGC-1**, Boc-on.

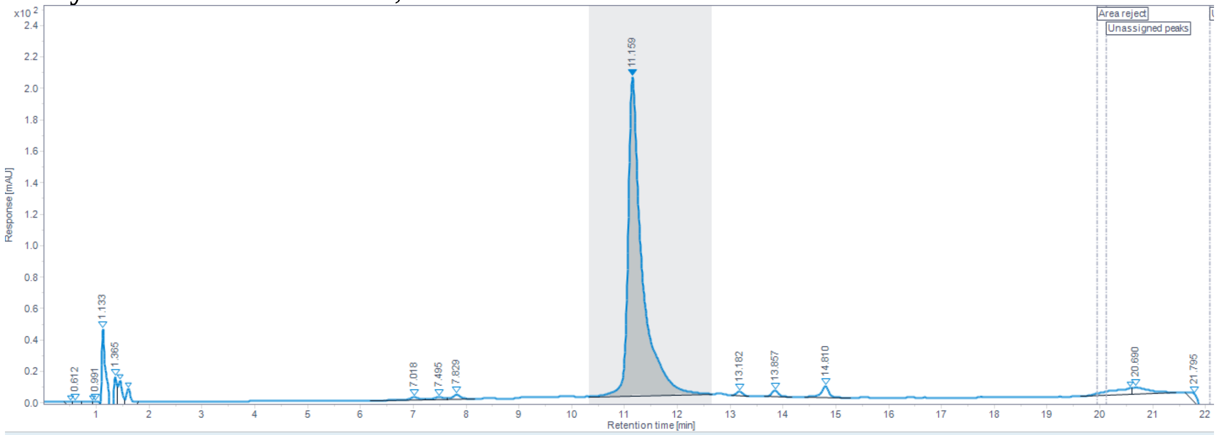

Injection Results

Peaks | Summary

#	Name	Signal description	RT (min)	Area (mAU·s)	Area%	Height (mAU)	Height%	Amount	Concentration	Start time (min)	End time (min)
7		DAD1A,Sig=260,4 Ref=off	1.456	235.322	3.683	35.826	7.53			1.411	1.547
8		DAD1A,Sig=260,4 Ref=off	1.616	162.828	2.549	20.164	4.24			1.547	1.798
9		DAD1A,Sig=260,4 Ref=off	7.018	30.567	0.478	1.886	0.40			6.195	7.226
10		DAD1A,Sig=260,4 Ref=off	7.495	28.596	0.448	1.973	0.41			7.226	7.658
11		DAD1A,Sig=260,4 Ref=off	7.829	38.339	0.600	3.086	0.65			7.658	8.164
12		DAD1A,Sig=260,4 Ref=off	11.159	3743.745	58.595	202.486	42.56			10.335	12.665

Entry 16: phosphate buffer, 9.0 min: **ATGC-1´**, Boc-on, 11.1 min: **ATGC-1**, Boc-on.

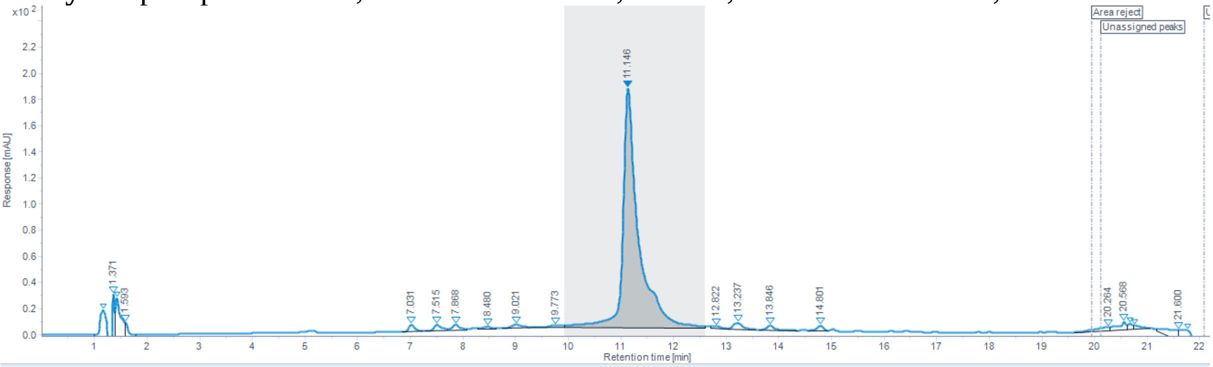

Injection Results

Peaks | Summary

#	Name	Signal description	RT (min)	Area (mAU·s)	Area%	Height (mAU)	Height%	Amount	Concentration	Start time (min)	End time (min)
7		DAD1A,Sig=260,4 Ref=off	7.868	42.741	0.682	4.241	0.97			7.728	8.092
8		DAD1A,Sig=260,4 Ref=off	8.480	21.104	0.337	1.860	0.43			8.285	8.636
9		DAD1A,Sig=260,4 Ref=off	9.021	42.033	0.671	2.587	0.59			8.753	9.483
10		DAD1A,Sig=260,4 Ref=off	9.773	28.185	0.450	1.745	0.40			9.483	9.941
11		DAD1A,Sig=260,4 Ref=off	11.146	3833.715	61.202	183.237	41.95			9.941	12.614
12		DAD1A,Sig=260,4 Ref=off	12.822	19.193	0.306	1.714	0.39			12.725	13.046

Entry 17: HEPES buffer, 9.0 min: **ATGC-1´**, Boc-on, 11.1 min: **ATGC-1**, Boc-on.

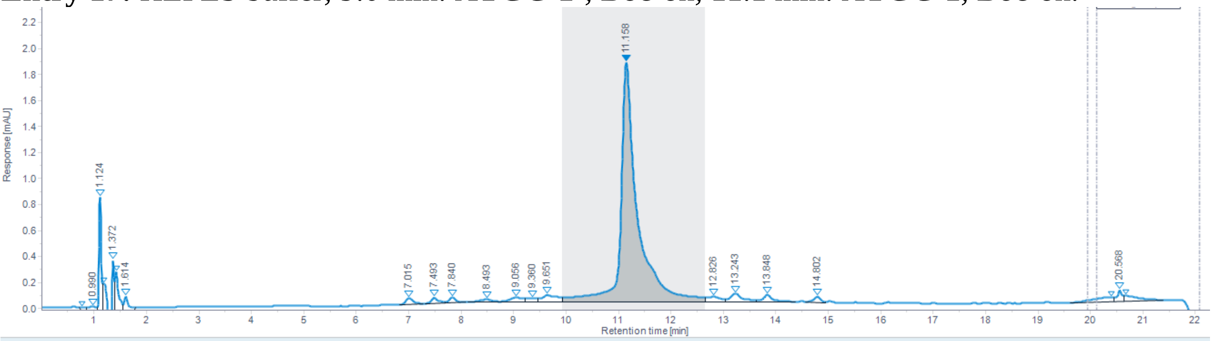

Injection Results

Peaks | Summary

#	Name	Signal description	RT (min)	Area (mAU·s)	Area%	Height (mAU)	Height%	Amount	Concentration	Start time (min)	End time (min)
12		DAD1A,Sig=260,4 Ref=off	9.056	51.819	0.735	3.121	0.53			8.745	9.228
13		DAD1A,Sig=260,4 Ref=off	9.360	35.980	0.510	2.549	0.44			9.228	9.474
14		DAD1A,Sig=260,4 Ref=off	9.651	104.002	1.474	5.036	0.86			9.474	9.943
15		DAD1A,Sig=260,4 Ref=off	11.158	4068.298	57.675	184.091	31.48			9.943	12.669
16		DAD1A,Sig=260,4 Ref=off	12.826	69.765	0.989	3.869	0.66			12.669	13.053

Entry 18: MES buffer, 9.0 min: **ATGC-1´**, Boc-on, 11.1 min: **ATGC-1**, Boc-on.

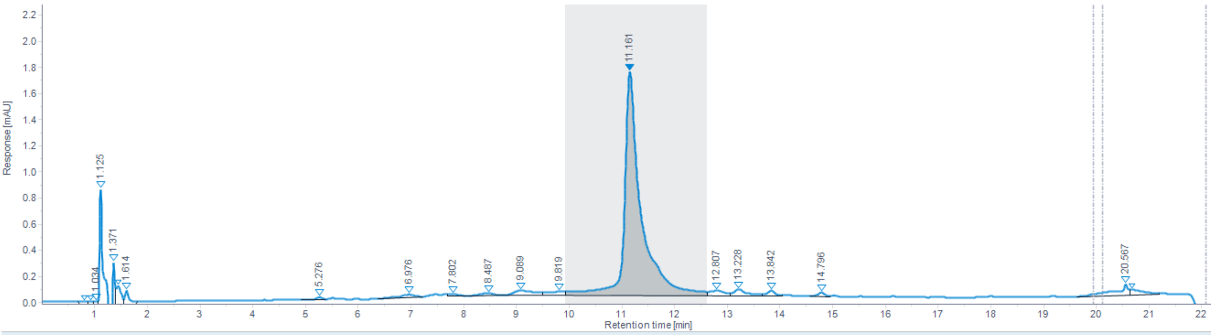

Injection Results

Peaks | Summary

#	Name	Signal description	RT (min)	Area (mAU·s)	Area%	Height (mAU)	Height%	Amount	Concentration	Start time (min)	End time (min)
12		DAD1A,Sig=260,4 Ref=off	9.089	101.456	1.526	3.585	0.71			8.757	9.514
13		DAD1A,Sig=260,4 Ref=off	9.819	65.537	0.986	2.950	0.59			9.514	9.938
14		DAD1A,Sig=260,4 Ref=off	11.161	3799.237	57.143	171.107	34.10			9.938	12.630
15		DAD1A,Sig=260,4 Ref=off	12.807	80.194	1.206	4.051	0.81			12.630	13.064

MALDI-MS: ATGC-1´, Boc-on, calculated m/z 5435, **ATGC-1**, Boc-on, calculated m/z 5463.

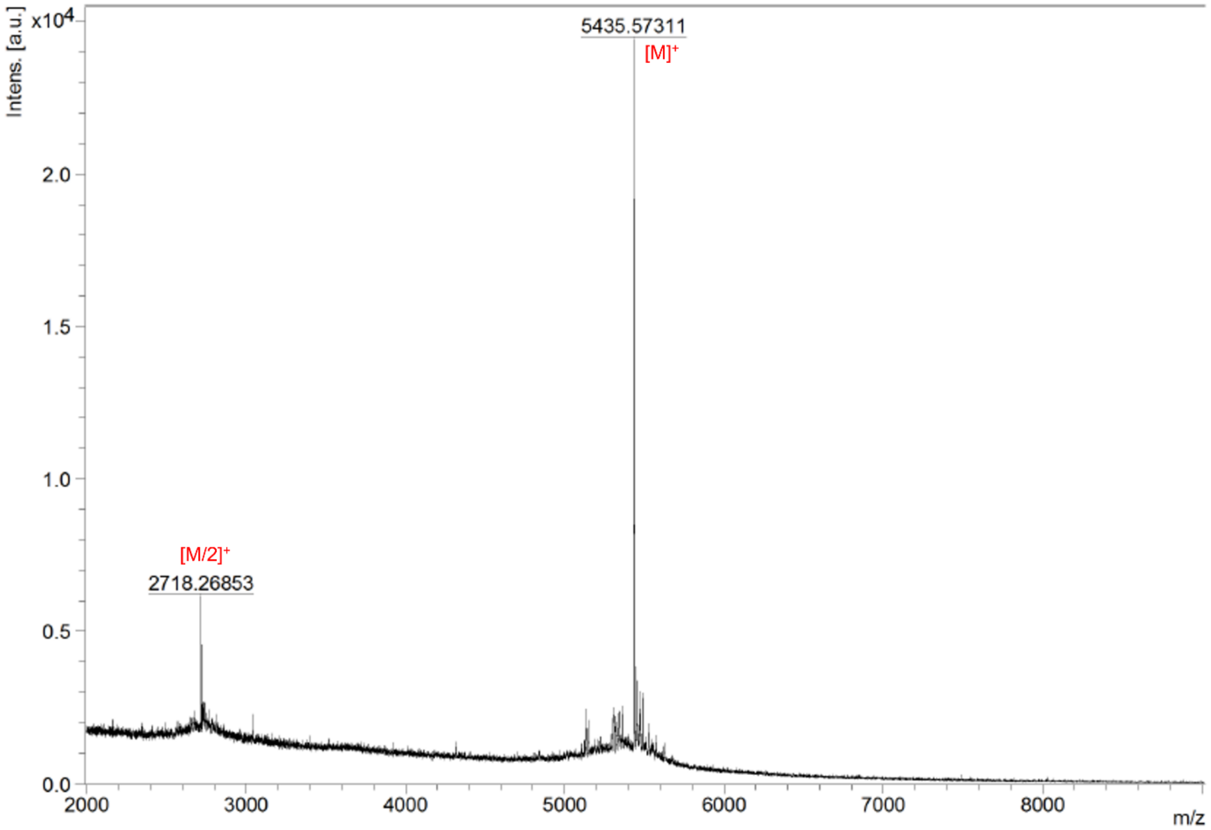

Figure S32. RP-HPLC traces for **Table S17** (entry 1-18), and MALDI-MS spectra for optimizing RCM reaction conditions with native DNA-tagged substrate **ATGC-1** in H_2O.

16. Synthesis of RNA-tagged macrocycle (purine25-AUGC-1´) via RCM in H_2O

RCM of **purine25-AUGC-1´** was performed as described in General Procedure 2. RCM conditions: 800 mM (80,000 equiv) NaCl, 800 mM (80,000 equiv) $NHEt_3Cl$, 10 equiv **Ru-1**, pH 5.0, 70 °C, 30 min.

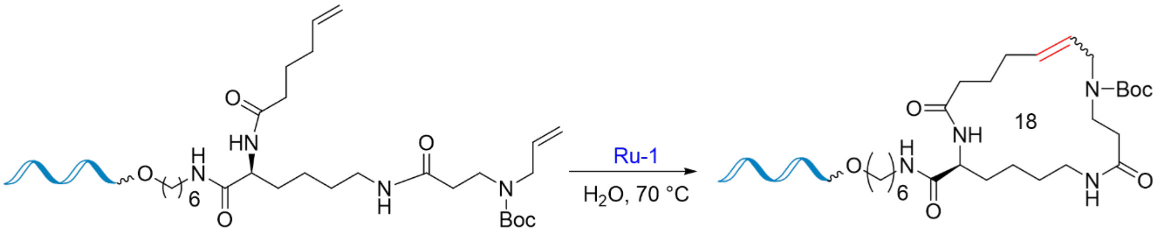

RP-HPLC chromatogram of RNA-tagged diene (purine25-AUGC-1) and its RCM in H_2O. RCM in H_2O: 8.8 min: **purine25-AUGC-1´**, Boc-on, 11.1 min: **purine25-AUGC-1**, Boc-on. **60% in situ yield**, **30% isolated yield**.

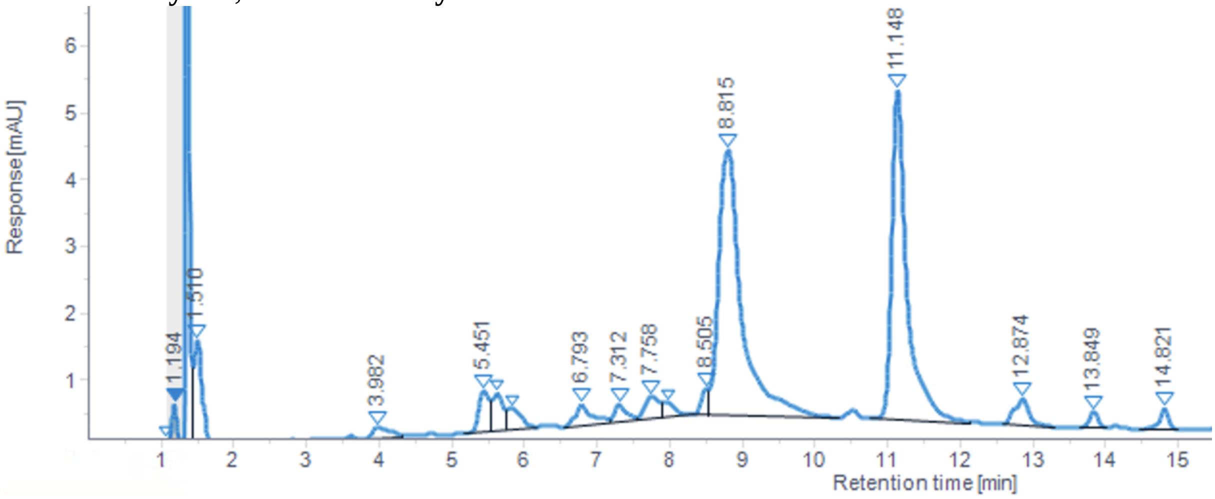

RCM in phosphate buffer: 9.1 min: **purine25-AUGC-1´**, Boc-on, 11.2 min: **purine25-AUGC-1**, Boc-on. **5% in situ yield**, **>95% total RNA recovery**.

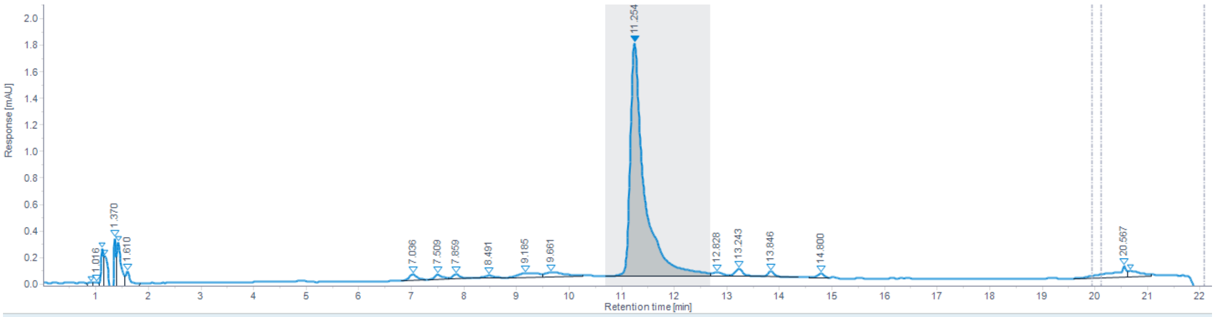

Injection Results

Peaks | Summary

#	Name	Signal description	RT (min)	Area (mAU·s)	Area%	Height (mAU)	Height%	Amount	Concentration	Start time (min)	End time (min)
10		DAD1A,Sig=260,4 Ref=off	7.859	33.047	0.556	3.515	0.70			7.717	8.161
11		DAD1A,Sig=260,4 Ref=off	8.491	26.263	0.442	1.959	0.39			8.166	8.815
12		DAD1A,Sig=260,4 Ref=off	9.185	83.884	1.410	3.015	0.60			8.826	9.508
13		DAD1A,Sig=260,4 Ref=off	9.661	85.945	1.445	3.492	0.69			9.508	10.275
14		DAD1A,Sig=260,4 Ref=off	11.254	3283.367	55.202	175.400	34.74			10.700	12.695
15		DAD1A,Sig=260,4 Ref=off	12.828	45.553	0.766	3.054	0.60			12.695	13.062

RCM in HEPES buffer: 9.6 min: **purine25-AUGC-1´**, Boc-on, 11.2 min: **purine25-AUGC-1**, Boc-on. <**5% in situ yield**, **83% total RNA recovery**.

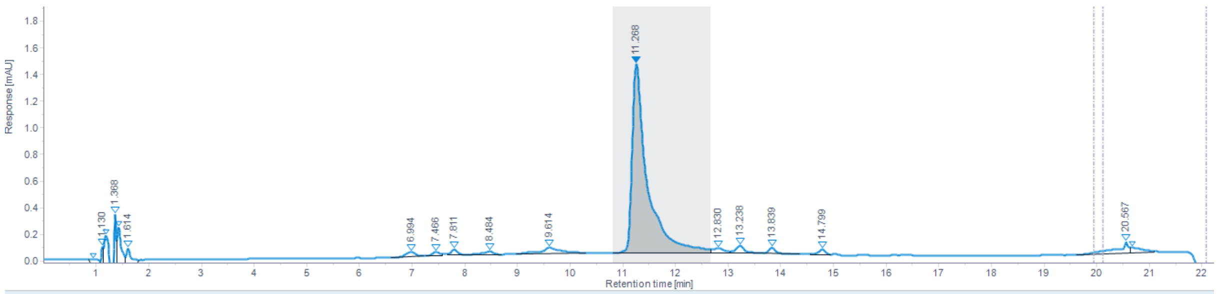

Injection Results

Peaks Summary

#	Name	Signal description	RT (min)	Area (mAU·s)	Area%	Height (mAU)	Height%	Amount	Concentration	Start time (min)	End time (min)
9		DAD1A,Sig=260,4 Ref=off	7.811	35.496	0.675	4.034	0.95			7.684	8.048
10		DAD1A,Sig=260,4 Ref=off	8.484	33.705	0.641	2.450	0.58			8.048	8.705
11		DAD1A,Sig=260,4 Ref=off	9.614	108.931	2.071	4.446	1.05			8.987	10.298
12		DAD1A,Sig=260,4 Ref=off	11.268	2874.298	54.652	141.874	33.46			10.820	12.691
13		DAD1A,Sig=260,4 Ref=off	12.830	55.345	1.052	3.705	0.87			12.691	13.047

RCM in MES buffer: 9.8 min: **purine25-AUGC-1´**, Boc-on, 11.2 min: **purine25-AUGC-1**, Boc-on. <**5% in situ yield**, **84% total RNA recovery**.

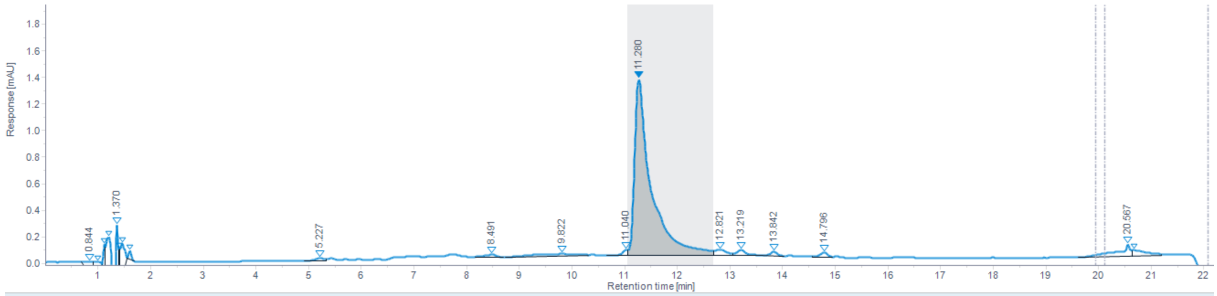

Injection Results

Peaks Summary

#	Name	Signal description	RT (min)	Area (mAU·s)	Area%	Height (mAU)	Height%	Amount	Concentration	Start time (min)	End time (min)
9		DAD1A,Sig=260,4 Ref=off	5.227	19.555	0.384	1.813	0.44			4.924	5.337
10		DAD1A,Sig=260,4 Ref=off	8.491	23.581	0.464	2.008	0.48			8.164	8.717
11		DAD1A,Sig=260,4 Ref=off	9.822	96.009	1.887	1.752	0.42			8.750	10.328
12		DAD1A,Sig=260,4 Ref=off	11.040	26.355	0.518	3.986	0.96			10.670	11.066
13		DAD1A,Sig=260,4 Ref=off	11.280	2906.169	57.134	132.443	31.94			11.066	12.696
14		DAD1A,Sig=260,4 Ref=off	12.821	67.488	1.327	4.377	1.06			12.696	13.072

Full MALDI-MS: purine25-AUGC-1´, Boc-on: calculated m/z 5541, **purine25-AUGC-1**, Boc-on: calculated m/z 5569.

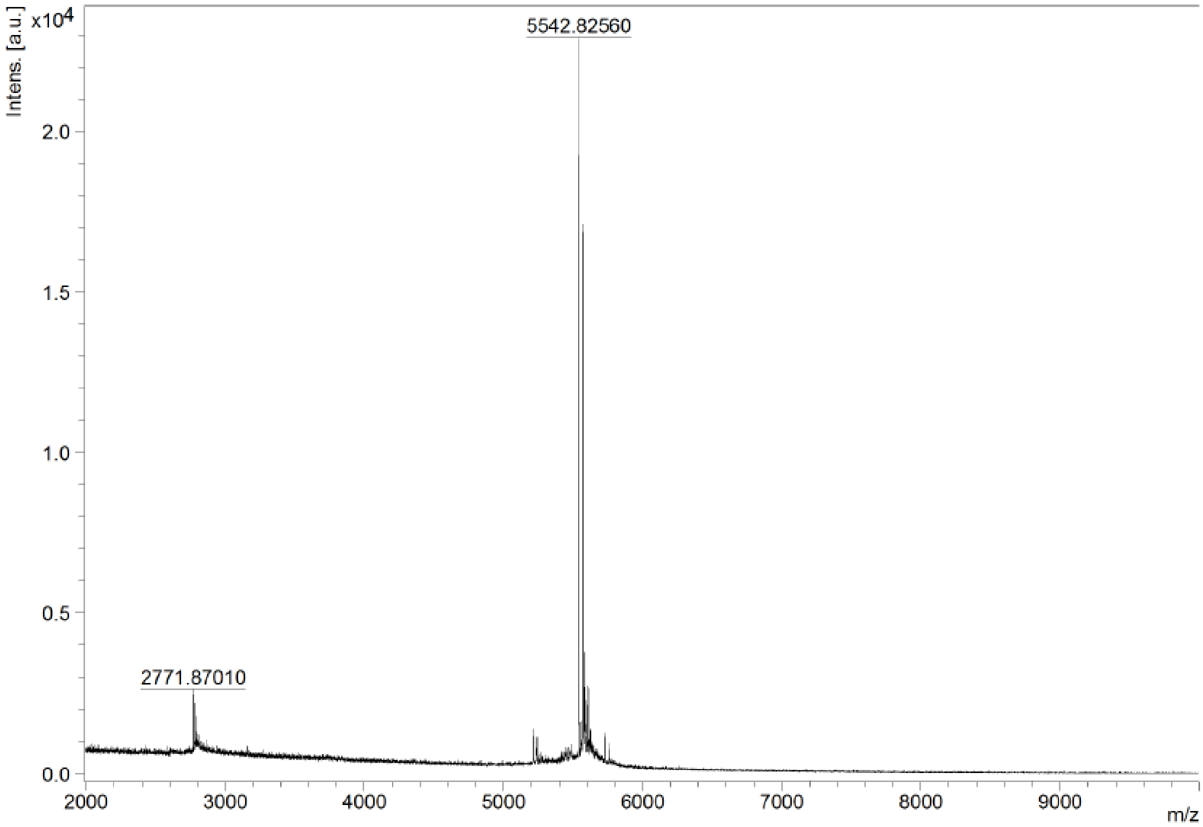

Zoomed-in MALDI-MS: purine25-AUGC-1´, Boc-on: calculated m/z 5541, **purine25-AUGC-1**, Boc-on: calculated m/z 5569.

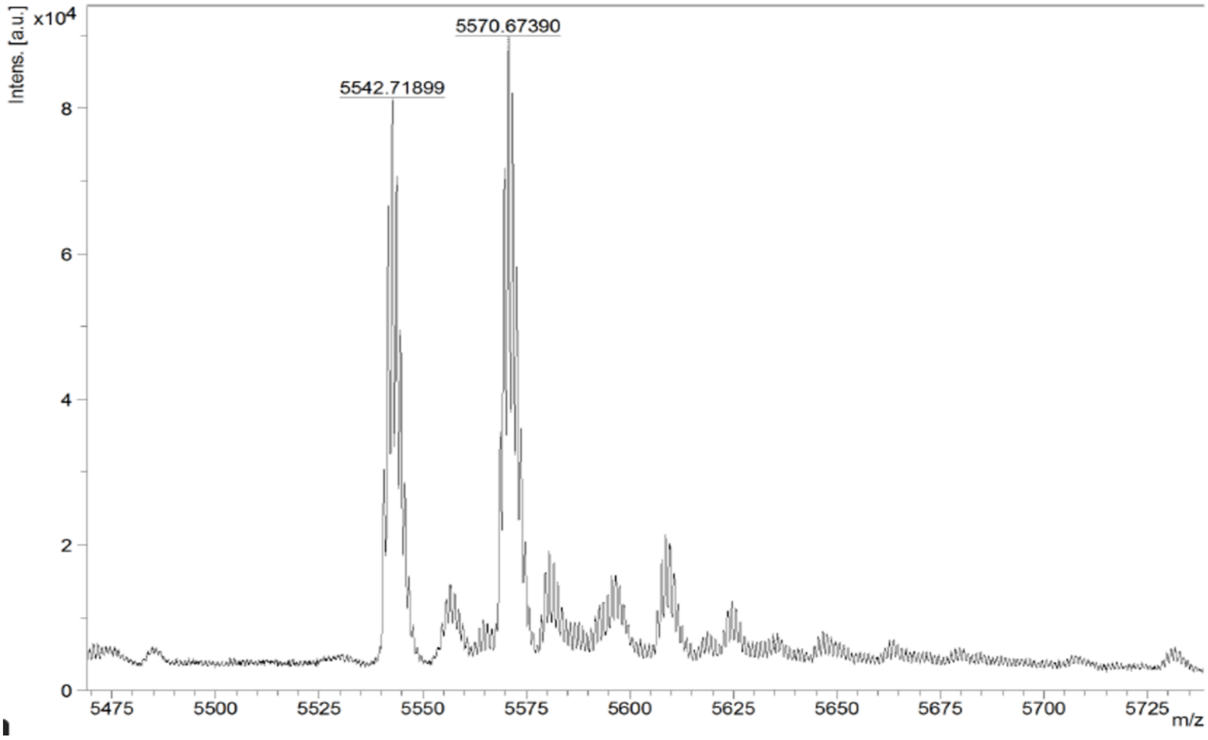

17. Synthesis of 2'-O-methyl-RNA-tagged macrocycle via RCM in H_2O

The synthesis of **2´-OMe AUGC-1´** was performed as described in General Procedure 2. RCM conditions: 800 mM (80,000 equiv) NaCl, 800 mM (80,000 equiv) $NHEt_3Cl$, 10 equiv **Ru-1**, pH 5.0, 70 °C, 30 min.

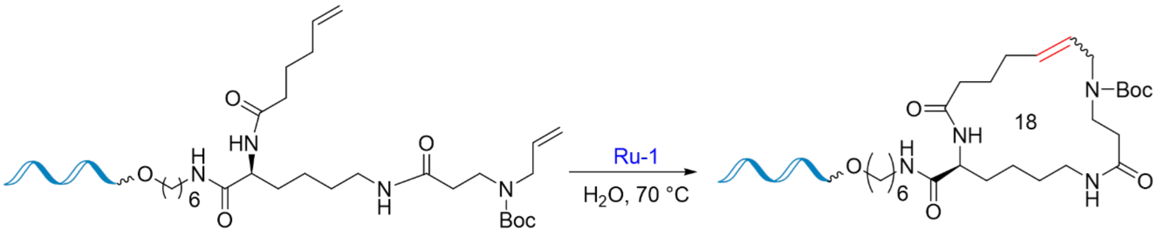

RP-HPLC chromatogram of 2´-OMe RNA-tagged substrate (2´-OMe AUGC-1) in H_2O.

RCM in H_2O: 9.5 min: **2´-OMe AUGC-1´**, Boc-on, 11.5 min: **2´-OMe AUGC-1**, Boc-on. **72% in situ yield**, **35% isolated yield**.

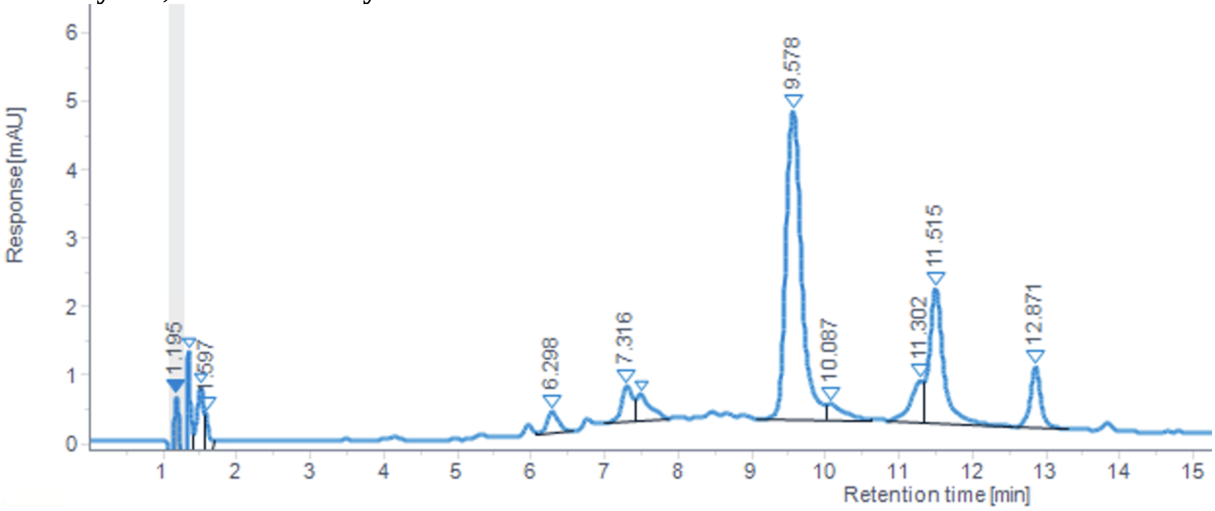

RCM in phosphate buffer: 9.7 min: **2´-OMe AUGC-1´**, Boc-on, 11.6 min: **2´-OMe AUGC-1**, Boc-on. **5% in situ yield**, **>95% total RNA recovery**.

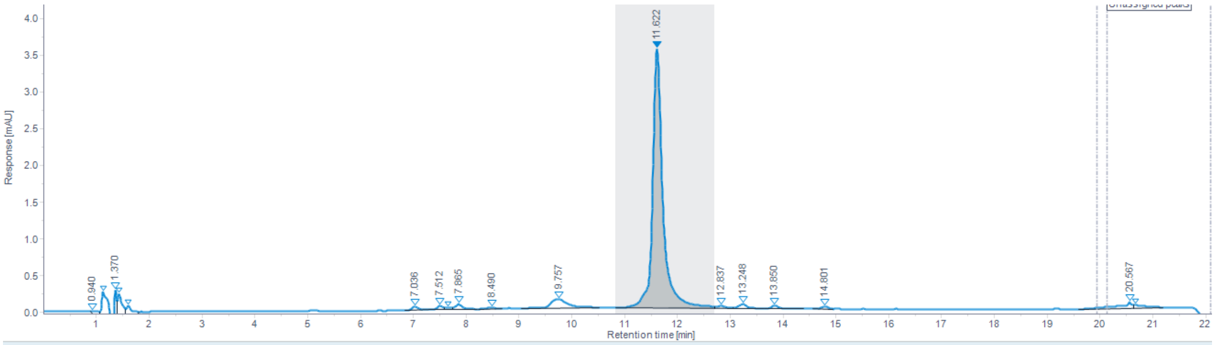

Injection Results

Peaks | Summary

#	Name	Signal description	RT (min)	Area (mAU·s)	Area%	Height (mAU)	Height%	Amount	Concentration	Start time (min)	End time (min)
9		DAD1A,Sig=260,4 Ref=off	7.865	65.735	0.883	6.722	1.09			7.752	8.224
10		DAD1A,Sig=260,4 Ref=off	8.490	19.109	0.257	1.855	0.30			8.243	8.682
11		DAD1A,Sig=260,4 Ref=off	9.757	263.089	3.532	11.769	1.92			9.056	10.534
12		DAD1A,Sig=260,4 Ref=off	11.622	4819.354	64.704	352.467	57.37			10.841	12.720
13		DAD1A,Sig=260,4 Ref=off	12.837	39.613	0.532	3.027	0.49			12.720	13.065

RCM in HEPES buffer: 9.7 min: **2´-OMe AUGC-1´**, Boc-on, 11.6 min: **2´-OMe AUGC-1**, Boc-on. **5% in situ yield**, **94% total RNA recovery**.

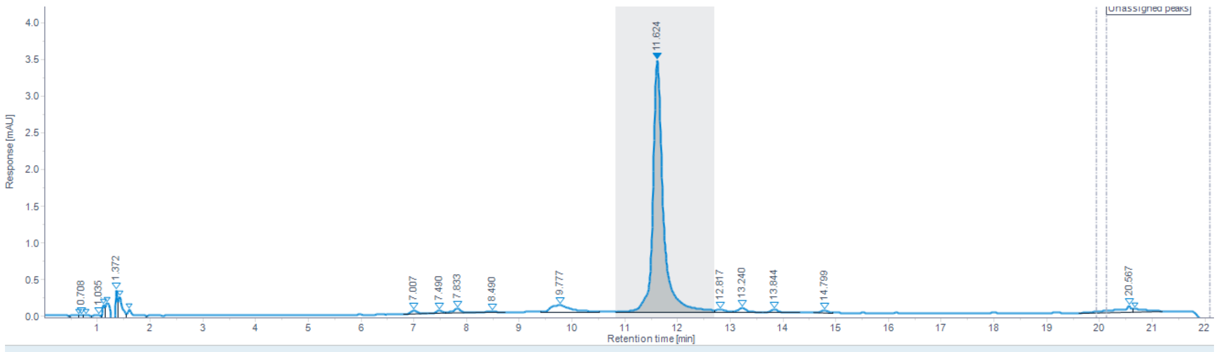

Injection Results

Peaks | Summary

#	Name	Signal description	RT (min)	Area (mAU·s)	Area%	Height (mAU)	Height%	Amount	Concentration	Start time (min)	End time (min)
11		DAD1A,Sig=260,4 Ref=off	7.490	33.992	0.428	4.301	0.60			7.287	7.609
12		DAD1A,Sig=260,4 Ref=off	7.833	69.900	0.880	6.428	0.90			7.609	8.182
13		DAD1A,Sig=260,4 Ref=off	8.490	23.613	0.297	1.978	0.28			8.187	8.733
14		DAD1A,Sig=260,4 Ref=off	9.777	265.402	3.340	9.470	1.33			9.415	10.522
15		DAD1A,Sig=260,4 Ref=off	11.624	4710.654	59.281	343.215	48.03			10.844	12.709
16		DAD1A,Sig=260,4 Ref=off	12.817	47.610	0.599	3.401	0.48			12.709	13.057

RCM in MES buffer: 9.8 min: **2´-OMe AUGC-1´**, Boc-on, 11.6 min: **2´-OMe AUGC-1**, Boc-on. **<5% in situ yield**, **90% total RNA recovery**.

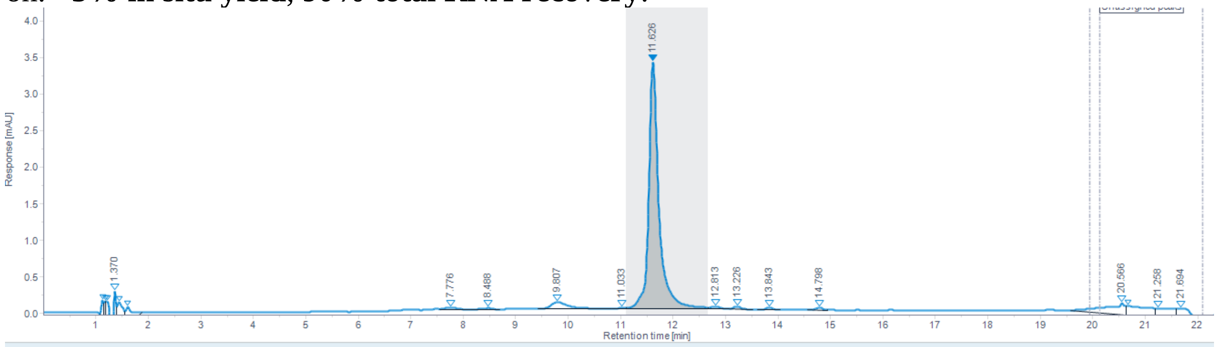

Injection Results

Peaks | Summary

#	Name	Signal description	RT (min)	Area (mAU·s)	Area%	Height (mAU)	Height%	Amount	Concentration	Start time (min)	End time (min)
7		DAD1A,Sig=260,4 Ref=off	7.776	51.452	0.641	2.982	0.45			7.561	8.156
8		DAD1A,Sig=260,4 Ref=off	8.488	21.151	0.264	1.786	0.27			8.156	8.713
9		DAD1A,Sig=260,4 Ref=off	9.807	220.618	2.750	9.259	1.40			9.427	10.840
10		DAD1A,Sig=260,4 Ref=off	11.033	18.387	0.229	2.144	0.32			10.847	11.120
11		DAD1A,Sig=260,4 Ref=off	11.626	4619.735	57.577	336.940	50.96			11.120	12.683
12		DAD1A,Sig=260,4 Ref=off	12.813	39.060	0.487	2.921	0.44			12.683	13.072

Full MALDI-MS: 2´-OMe AUGC-1´, Boc-on: calculated m/z 5766, **2´-OMe AUGC-1**, Boc-on: calculated m/z 5794.

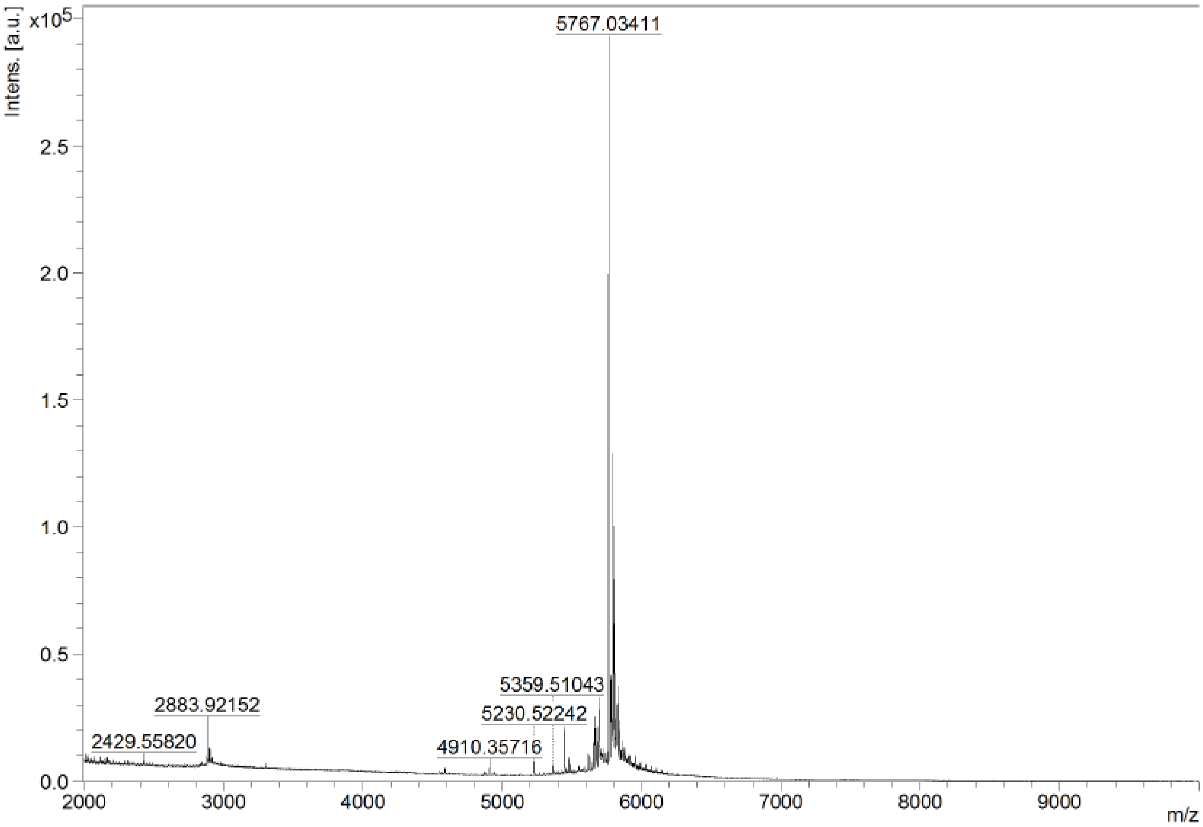

Zoomed-in MALDI-MS: 2´-OMe AUGC-1´, Boc-on: calculated m/z 5766, **2´-OMe AUGC-1**, Boc-on: calculated m/z 5794.

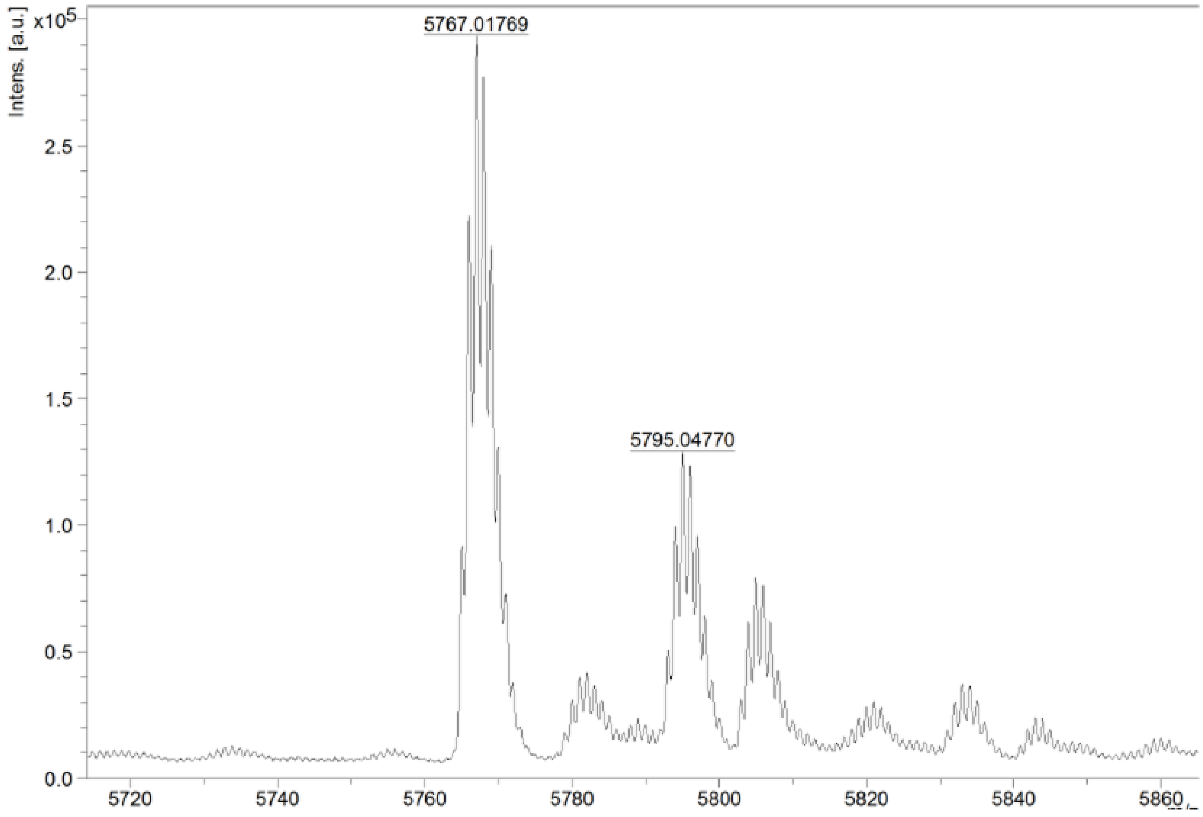

Figure S33. RP-HPLC traces and MALDI-MS spectra for RCM on native RNA **(purine25-AUGC-1**) and 2´-OMe RNA (**2´-OMe AUGC-1**) in H_2O.

18. Tolerance to different purine content in DNA/RNA oligomers and dsDNA headpiece in optimized RCM conditions in water

All RCM reactions were carried out with 2 nmol of DNA or RNA oligomers in **DNA LoBind® Eppendorf 96 deep well plates**. The aqueous solutions pH 5.0 were prepared by acidifying Millipore H_2O with a 1 M HCl solution. Stock solutions of 4 M NaCl and 4 M $NHEt_3Cl$ and 1.2 mg/mL **Ru-1** were prepared at pH 5.0 H_2O, respectively. Prior to RCM reactions, all stock solutions were filtered via sterile Millex™-GV Filter Unit (pore size 0.22 µm, 33 mm diameter, PVDF membrane). 5 µL oligonucleotide, 40 µL 4 M NaCl and 40 µL 4 M $NHEt_3Cl$ solutions, 10 µL **Ru-1** solution and pH 5.0 H_2O were added to the plates to a final volume of 100 µL. The solution was flushed with argon, sealed with Eppendorf heat sealing foil and shaken on an Eppendorf thermocycler at 70 °C for 30 min. The reactions were quenched by adding SnatchCat (1,4-bis(3-isocyanopropyl) piperazine; 10 equiv vs Ru) and shaking at 37 °C for 30 min. After the RCM reactions, ethanol precipitation was performed. 10% v/v 3M sodium acetate buffer (pH 5.2) was added into the reaction solutions, after addition of 3.5 volumes of cold EtOH, the solution was left at -80 °C overnight. DNA/RNA was recovered as pellet by centrifugation (2,250 × g, -10 °C, 1 h), the supernatant discarded, and the pellet was washed twice using cold 70% EtOH and centrifuged (2,250 × g, -10 °C, 20 min). The recovered pellet was dried under reduced pressure in a SpeedVac vacuum concentrator. The pellet was redissolved in nuclease-free water and analyzed by MALDI-MS and analytical RP-HPLC. The RCM reactions were repeated one more time and purified via semi-preparative RP-HPLC to determine the recoveries. RCM conditions: 800 mM (80,000 equiv) NaCl, 800 mM (80,000 equiv) $NHEt_3Cl$, 10 equiv **Ru-1**, pH 5.0, 70 °C, 30 min.

(a) 16mer native DNA with 25% purine content (purine25-ATGC-1)

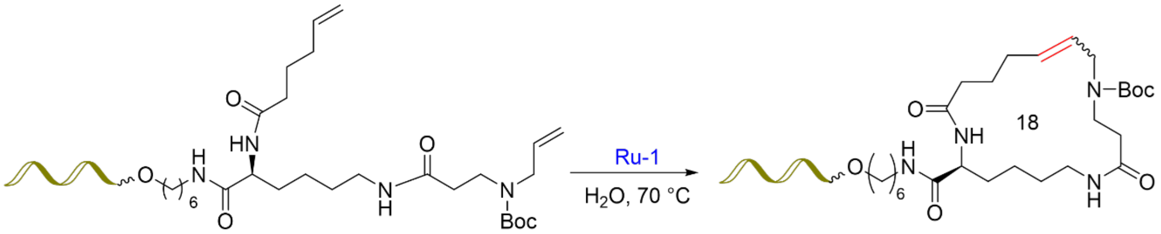

purine25-ATGC-1 **purine25-ATGC-1´**

RP-HPLC chromatogram of **purine25-ATGC-1** after RCM in H_2O
9.0 min: **purine25-ATGC-1´**, 10.9 min: **purine25-ATGC-1**. 95% in situ yield, 1.34 nmol DNA-macrocycle product (isolated yield, 67%).

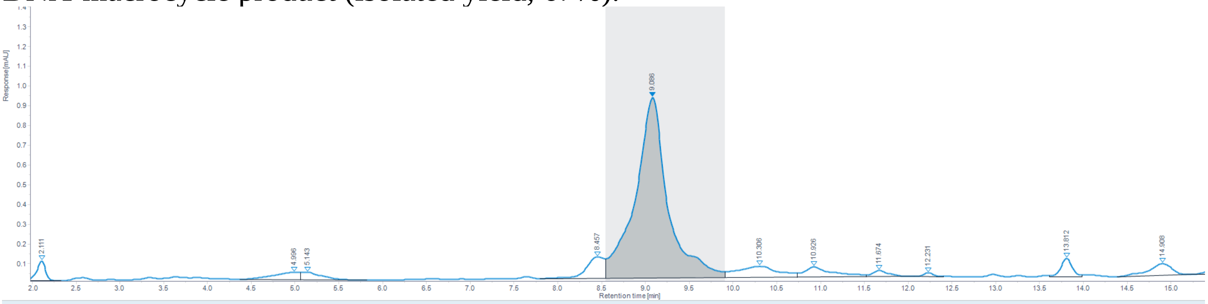

Injection Results

Peaks Summary

#	Name	Signal description	RT (min)	Area (mAU·s)	Area%	Height (mAU)	Height%	Amount	Concentration	Start time (min)	End time (min)
7		DAD1A,Sig=260,4 Ref=off	4.996	79.104	0.755	3.732	0.27			4.374	5.063
8		DAD1A,Sig=260,4 Ref=off	5.143	64.581	0.617	3.890	0.29			5.063	5.826
9		DAD1A,Sig=260,4 Ref=off	8.457	155.025	1.480	10.913	0.80			7.800	8.548
10		DAD1A,Sig=260,4 Ref=off	9.086	2362.179	22.557	91.553	6.71			8.548	9.911
11		DAD1A,Sig=260,4 Ref=off	10.306	184.156	1.759	5.627	0.41			9.911	10.734
12		DAD1A,Sig=260,4 Ref=off	10.926	118.506	1.132	5.198	0.38			10.734	11.526
13		DAD1A,Sig=260,4 Ref=off	11.674	41.161	0.393	3.211	0.24			11.526	12.072
14		DAD1A,Sig=260,4 Ref=off	12.231	14.772	0.141	1.900	0.14			12.073	12.408

Full MALDI-MS: **purine25-ATGC-1´**, Boc-on, calculated m/z 5322.

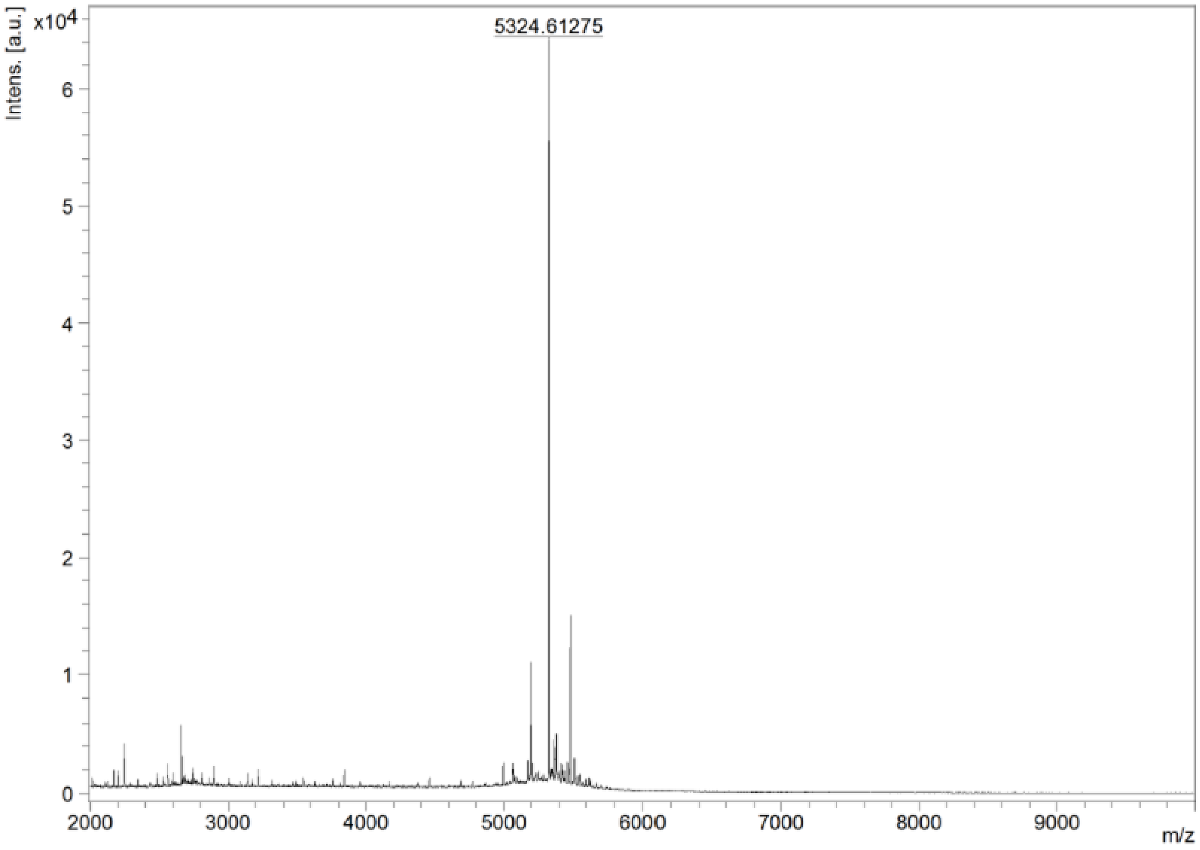

(b) 16mer native DNA with 25% purine content (purine25-ATGC-2)

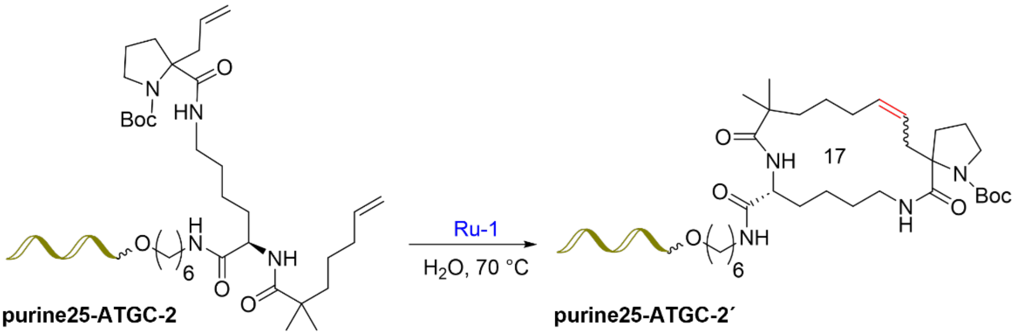

purine25-ATGC-2 **purine25-ATGC-2´**

RP-HPLC chromatogram of **purine25-ATGC-2** after RCM in H_2O
12.1 min: **purine25-ATGC-2´**, 14.0 min: **purine25-ATGC-2**. 66% in situ yield, 1.06 nmol DNA-macrocycle product (isolated yield, 53%)

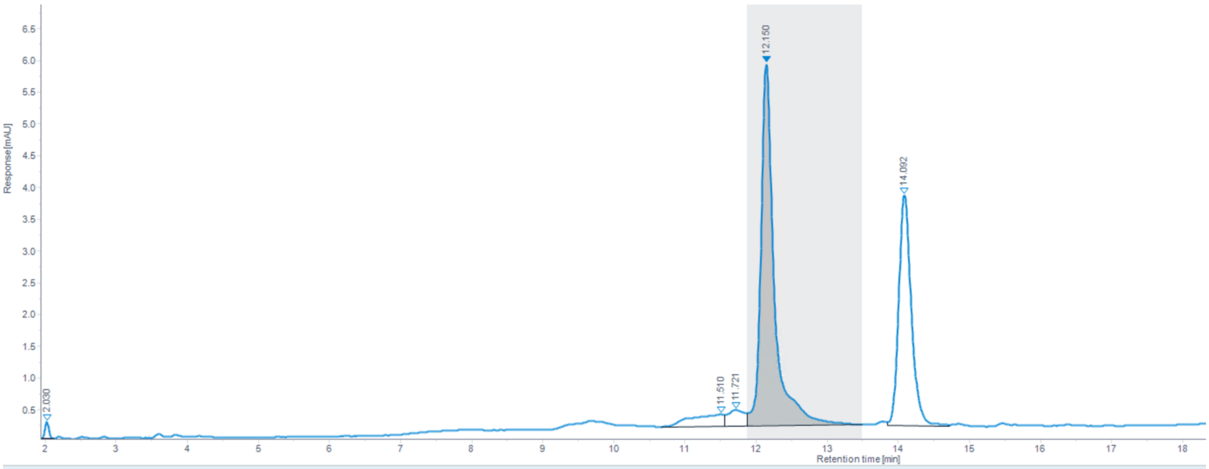

Injection Results

Peaks | Summary

#	Name	Signal description	RT (min)	Area (mAU·s)	Area%	Height (mAU)	Height%	Amount	Concentration	Start time (min)	End time (min)
1		DAD1A,Sig=260,4 Ref=off	0.800	50.284	1.804	6.342	3.46			0.547	0.803
2		DAD1A,Sig=260,4 Ref=off	0.998	156.360	5.609	11.419	6.23			0.803	1.069
3		DAD1A,Sig=260,4 Ref=off	1.201	181.145	6.498	22.458	12.25			1.069	1.260
4		DAD1A,Sig=260,4 Ref=off	1.311	40.275	1.445	12.939	7.06			1.267	1.362
5		DAD1A,Sig=260,4 Ref=off	2.030	10.425	0.374	2.634	1.44			1.946	2.127
6		DAD1A,Sig=260,4 Ref=off	11.510	59.914	2.149	1.860	1.01			10.667	11.561
7		DAD1A,Sig=260,4 Ref=off	11.721	41.441	1.487	2.531	1.38			11.561	11.878
8		DAD1A,Sig=260,4 Ref=off	12.150	747.353	26.810	56.989	31.10			11.878	13.499
9		DAD1A,Sig=260,4 Ref=off	14.092	445.728	15.990	36.296	19.81			13.854	14.730

MALDI-MS analysis of **purine25-ATGC-2´**, Boc-on, calculated m/z 5612.

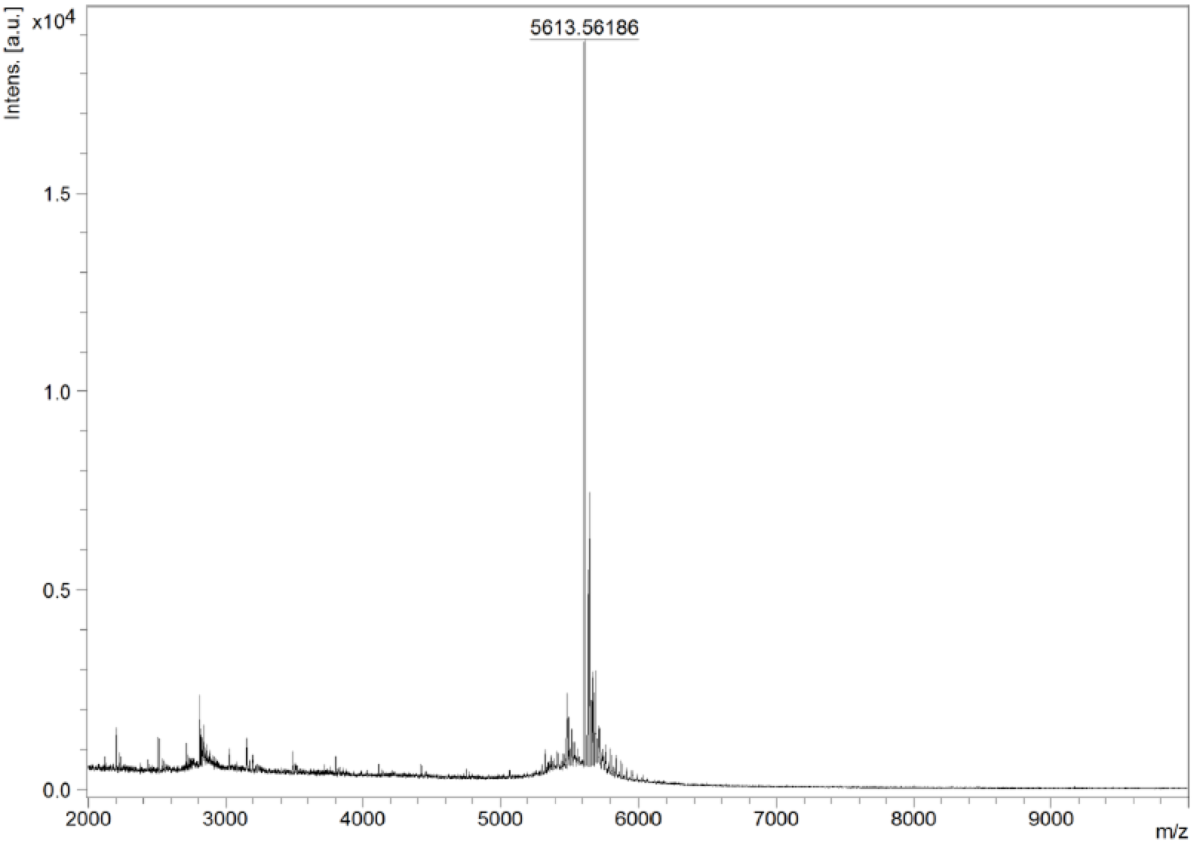

(c) 16mer native DNA with 75% purine content (purine75-ATGC-1)

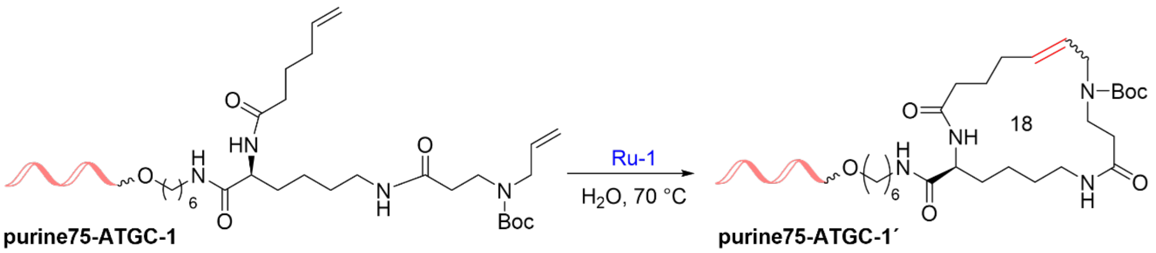

RP-HPLC chromatogram of **purine75-ATGC-1** after RCM in H_2O

9.6 min: **purine75-ATGC-1´**, 11.3 min: **purine75-ATGC-1**. 76% in situ yield, 1.36 nmol DNA-macrocycle product (isolated yield, 68%).

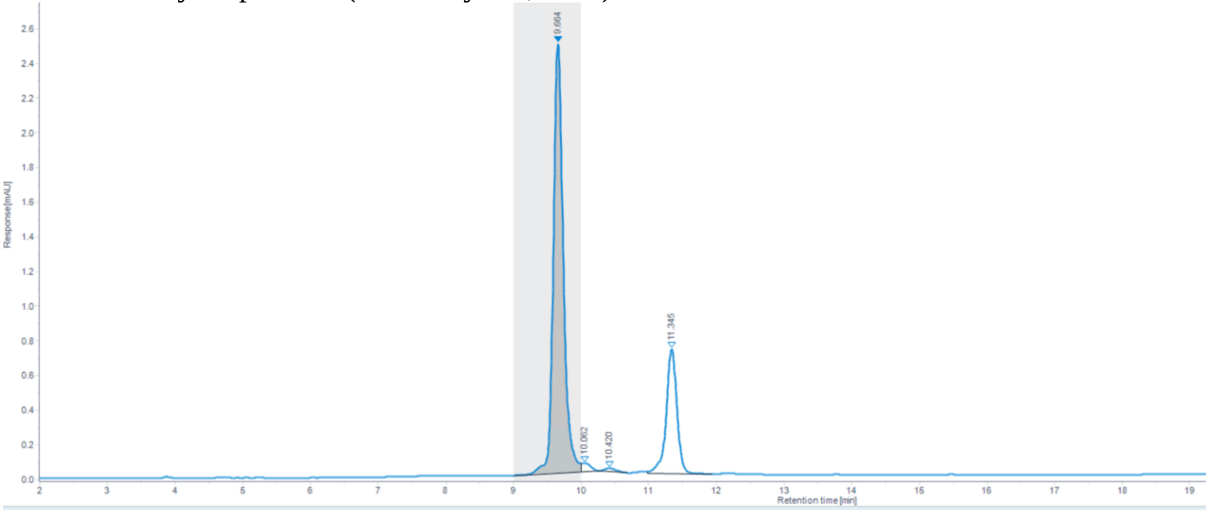

Injection Results

Peaks | Summary

#	Name	Signal description	RT (min)	Area (mAU·s)	Area%	Height (mAU)	Height%	Amount	Concentration	Start time (min)	End time (min)
1		DAD1A,Sig=260,4 Ref=off	1.198	106.075	2.193	17.497	4.27			1.060	1.260
2		DAD1A,Sig=260,4 Ref=off	1.313	71.854	1.486	18.812	4.59			1.267	1.357
3		DAD1A,Sig=260,4 Ref=off	1.386	72.334	1.495	12.654	3.09			1.357	1.459
4		DAD1A,Sig=260,4 Ref=off	1.586	92.931	1.921	6.430	1.57			1.459	1.756
5		DAD1A,Sig=260,4 Ref=off	9.664	2545.674	52.631	247.970	60.54			9.013	10.006
6		DAD1A,Sig=260,4 Ref=off	10.062	45.057	0.932	4.889	1.19			10.006	10.273
7		DAD1A,Sig=260,4 Ref=off	10.420	22.301	0.461	1.899	0.46			10.273	10.680
8		DAD1A,Sig=260,4 Ref=off	11.345	791.807	16.370	71.945	17.56			10.990	11.940
9		DAD1A,Sig=260,4 Ref=off	20.652	222.538	4.601	3.892	0.95			19.600	21.502

Full MALDI-MS: **purine75-ATGC-1´**, Boc-on, calculated m/z 5454.

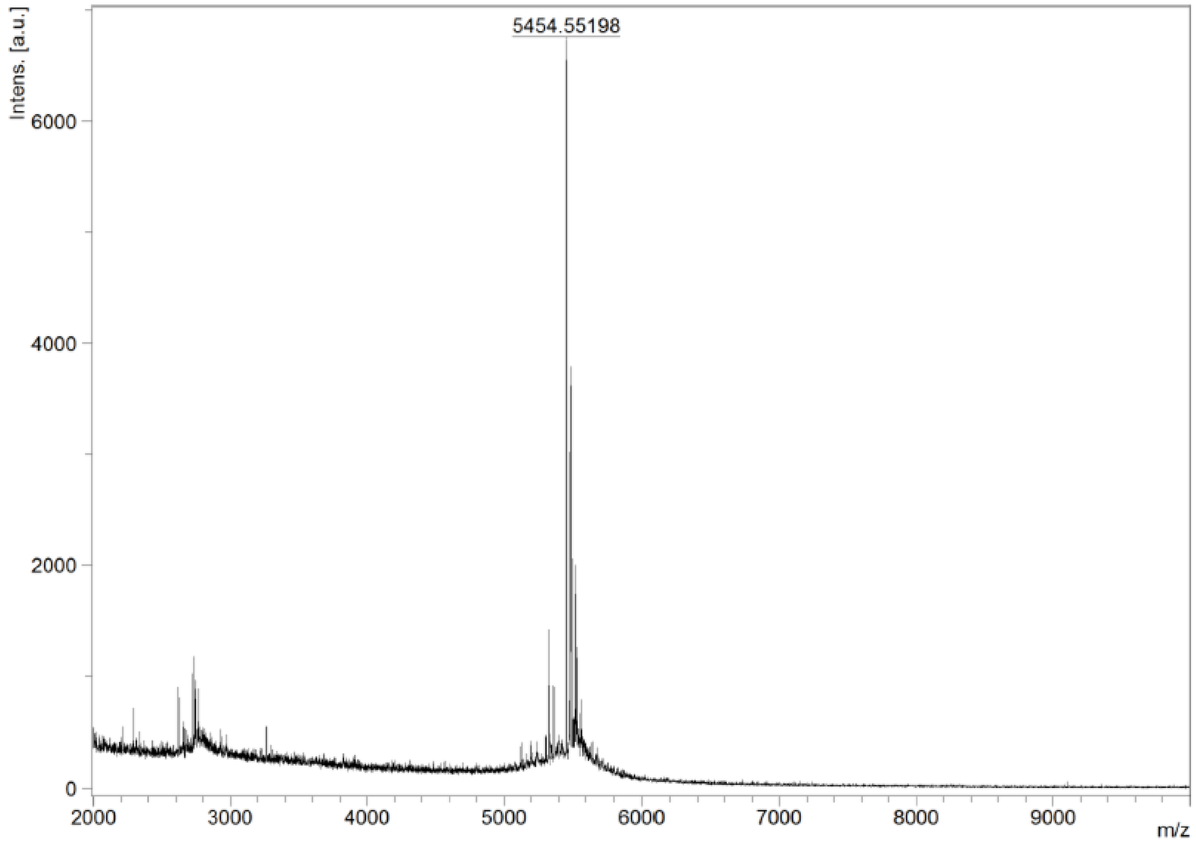

Zoomed-in MALDI-MS of **purine75-ATGC-1´**, Boc-on, calculated m/z 5454. **purine75-ATGC-**1, Boc-on, calculated m/z 5482.

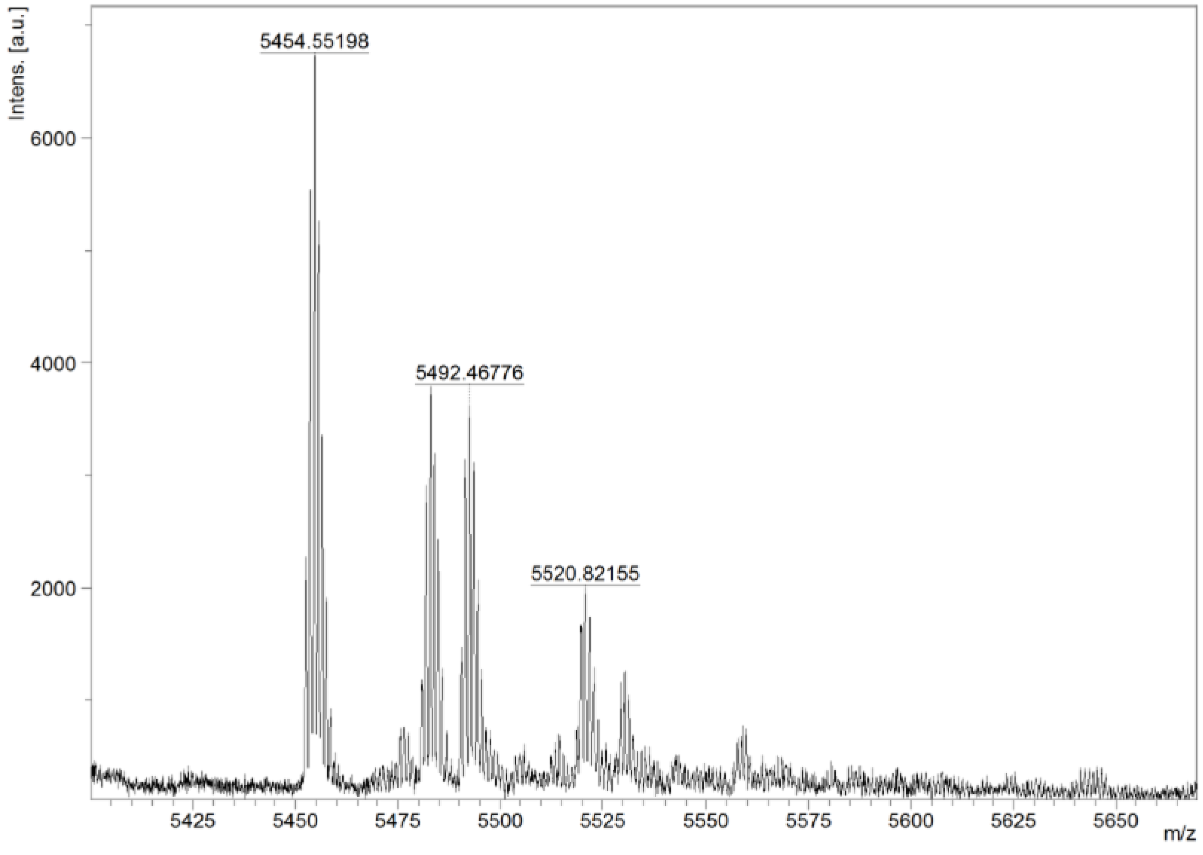

(c) 16mer native DNA with 75% purine content (purine75-ATGC-2)

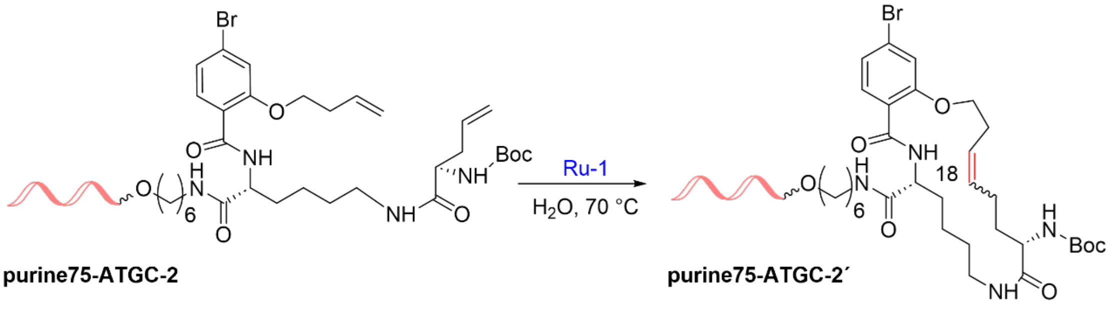

RP-HPLC chromatogram of **purine75-ATGC-2** after RCM in H_2O
12.1 min: **purine75-ATGC-2´**, 13.9 min: **purine75-ATGC-2**. 74% in situ yield, 1.22 nmol DNA-macrocycle product (isolated yield, 61%).

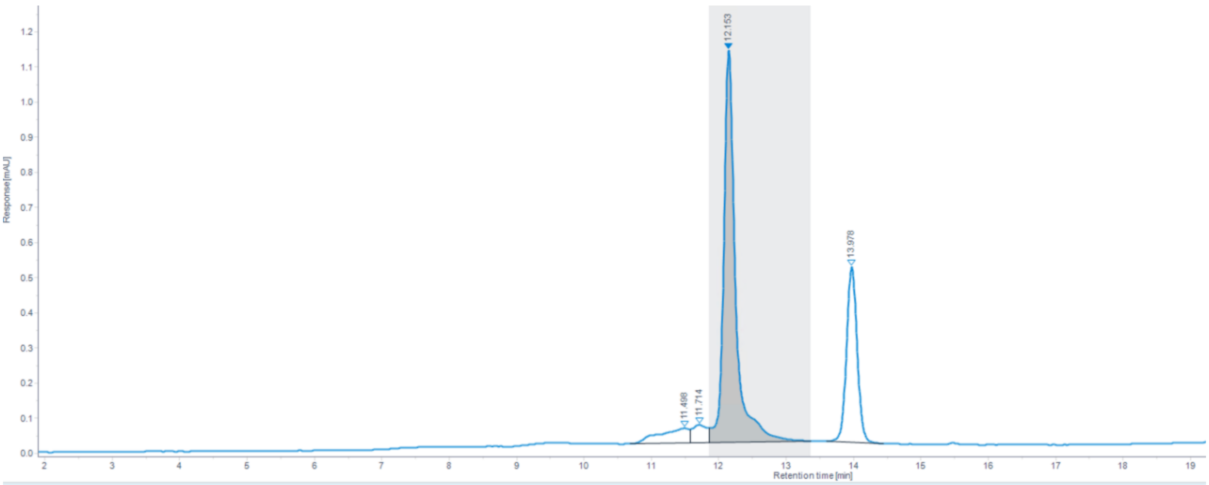

Injection Results

Peaks Summary

#	Name	Signal description	RT (min)	Area (mAU·s)	Area%	Height (mAU)	Height%	Amount	Concentration	Start time (min)	End time (min)
1		DAD1A,Sig=260,4 Ref=off	0.890	19.541	0.383	3.039	1.02			0.815	0.942
2		DAD1A,Sig=260,4 Ref=off	1.192	217.717	4.267	21.052	7.10			0.942	1.259
3		DAD1A,Sig=260,4 Ref=off	1.309	172.429	3.379	20.453	6.90			1.261	1.463
4		DAD1A,Sig=260,4 Ref=off	1.533	113.554	2.225	11.021	3.72			1.463	1.643
5		DAD1A,Sig=260,4 Ref=off	1.746	57.538	1.128	5.796	1.95			1.643	1.792
6		DAD1A,Sig=260,4 Ref=off	1.853	20.794	0.408	3.179	1.07			1.792	1.895
7		DAD1A,Sig=260,4 Ref=off	11.498	129.959	2.547	4.229	1.43			10.688	11.578
8		DAD1A,Sig=260,4 Ref=off	11.714	79.346	1.555	5.150	1.74			11.578	11.869
9		DAD1A,Sig=260,4 Ref=off	12.153	1369.160	26.833	111.744	37.69			11.869	13.376
10		DAD1A,Sig=260,4 Ref=off	13.978	546.707	10.715	49.886	16.83			13.608	14.455
11		DAD1A,Sig=260,4 Ref=off	20.555	449.148	8.803	13.073	4.41			19.388	20.561

Full MALDI-MS of **purine75-ATGC-2´**, Boc-on, calculated m/z 5596.

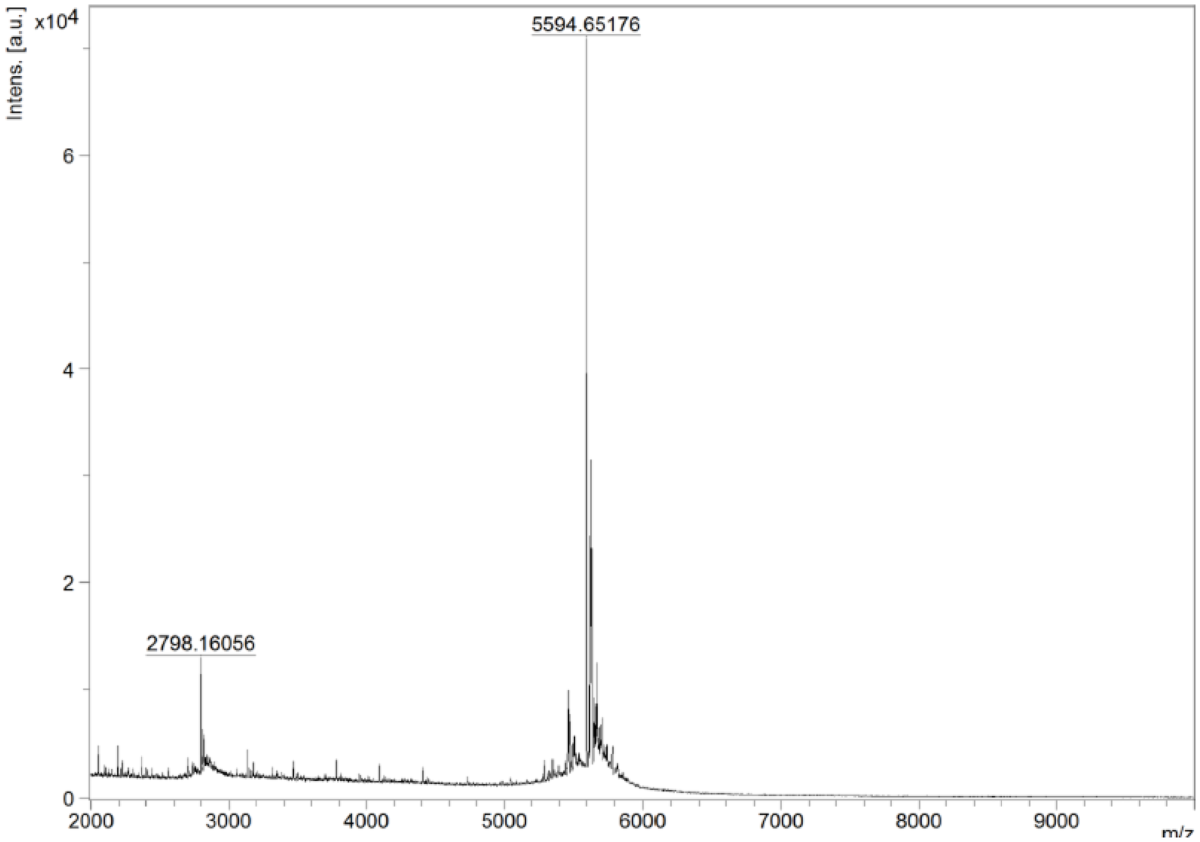

Zoomed-in MALDI-MS: **purine75-ATGC-2´**, Boc-on, calculated m/z 5596. **purine75-ATGC-2,** Boc-on, calculated m/z 5624.

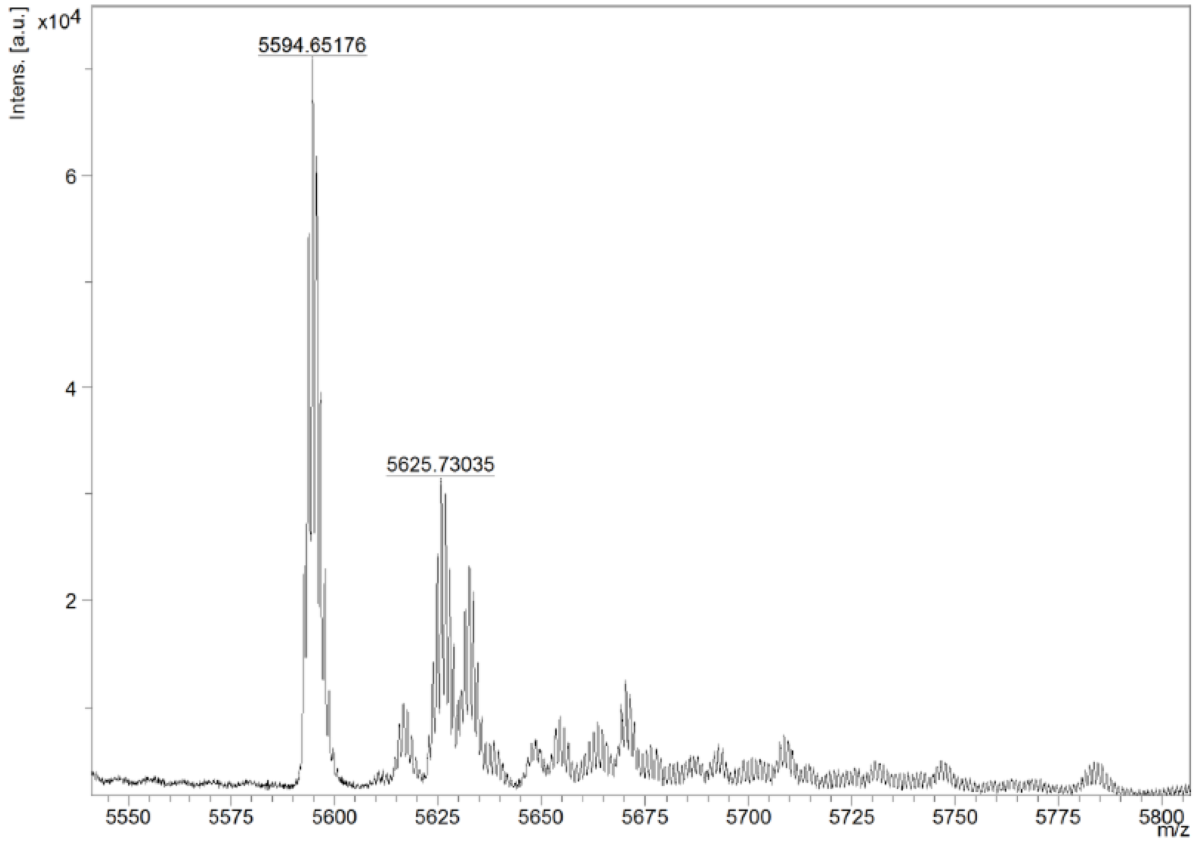

(d) dsDNA headpiece (headpiece-1)

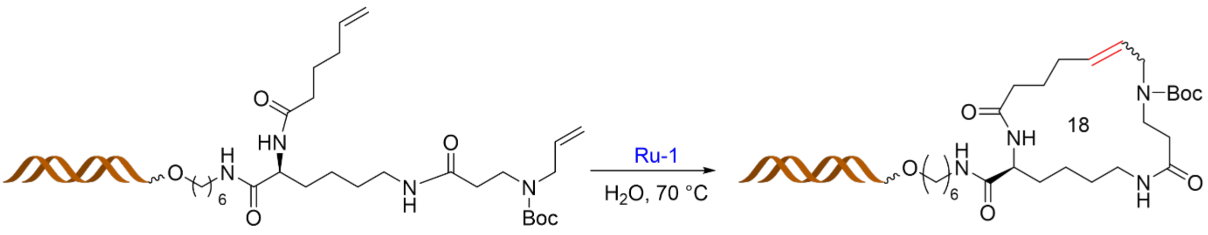

headpiece-1 **headpiece-1´**

RP-HPLC chromatogram of **headpiece-1** after RCM in H_2O
8.6 min: **headpiece-1´**, 10.3 min: **headpiece-1**. 90% in situ yield, 1.64 nmol DNA-macrocycle product (isolated yield, 82%).

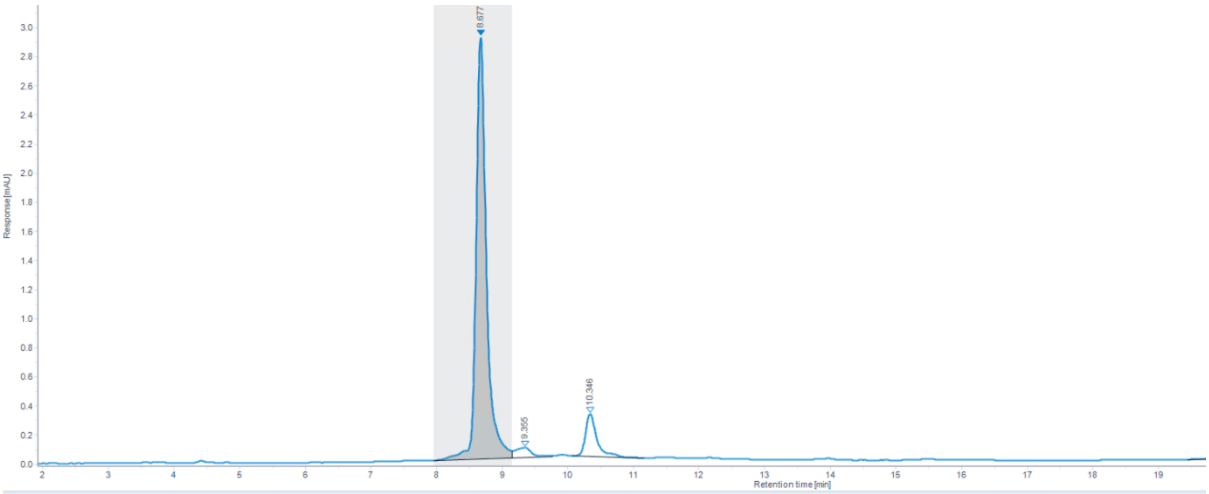

Injection Results

Peaks Summary

#	Name	Signal description	RT (min)	Area (mAU·s)	Area%	Height (mAU)	Height%	Amount	Concentration	Start time (min)	End time (min)
1		DAD1A,Sig=260,4 Ref=off	0.960	32.453	0.642	3.551	0.88			0.888	1.038
2		DAD1A,Sig=260,4 Ref=off	1.183	167.385	3.312	19.702	4.86			1.038	1.260
3		DAD1A,Sig=260,4 Ref=off	1.310	83.185	1.646	18.520	4.57			1.261	1.375
4		DAD1A,Sig=260,4 Ref=off	1.430	53.241	1.053	7.150	1.76			1.375	1.543
5		DAD1A,Sig=260,4 Ref=off	8.677	3188.187	63.085	290.001	71.55			7.968	9.159
6		DAD1A,Sig=260,4 Ref=off	9.355	116.372	2.303	6.796	1.68			9.159	9.774
7		DAD1A,Sig=260,4 Ref=off	10.346	350.639	6.938	29.208	7.21			10.061	11.168
8		DAD1A,Sig=260,4 Ref=off	20.530	125.074	2.475	3.881	0.96			19.448	20.556

Full MALDI-MS: **headpiece-1´**, Boc-on, calculated m/z 5344.

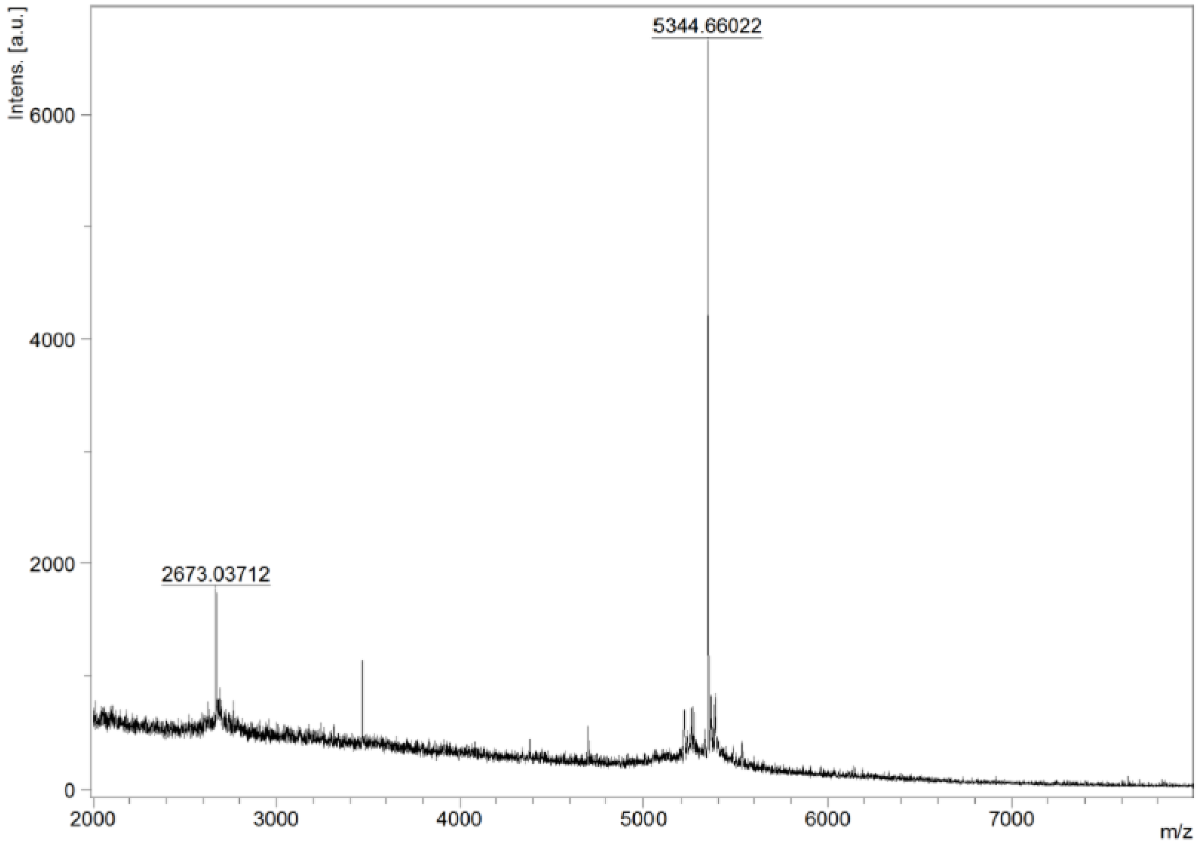

(e) dsDNA headpiece (headpiece-2)

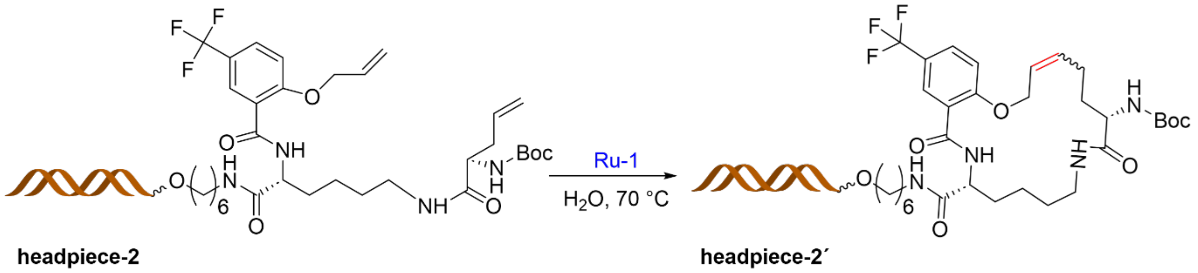

RP-HPLC chromatogram of **headpiece-2** after RCM in H_2O
9.6 min: **headpiece-2´**, 12.1 min: **headpiece-2**. 87% in situ yield, 1.34 nmol DNA-macrocycle product (isolated yield, 67%).

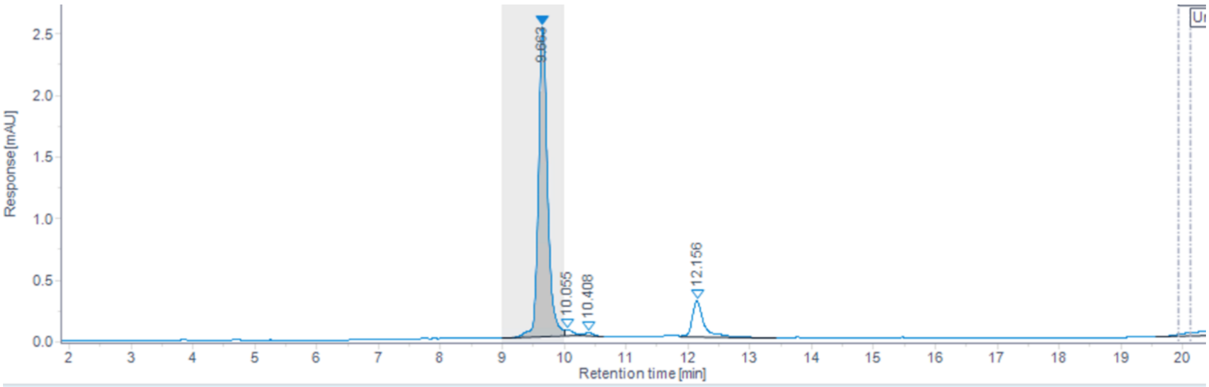

Injection Results

Peaks | Summary

#	Name	Signal description	RT (min)	Area (mAU·s)	Area%	Height (mAU)	Height%	Amount
5		DAD1A,Sig=260,4 Ref=off	1.419	28.836	0.629	4.596	1.24	
6		DAD1A,Sig=260,4 Ref=off	9.663	2594.112	56.575	251.311	67.95	
7		DAD1A,Sig=260,4 Ref=off	10.055	41.231	0.899	4.658	1.26	
8		DAD1A,Sig=260,4 Ref=off	10.408	21.535	0.470	2.123	0.57	
9		DAD1A,Sig=260,4 Ref=off	12.156	385.499	8.407	29.213	7.90	
10		DAD1A,Sig=260,4 Ref=off	20.647	223.023	4.864	3.940	1.07	

Full MALDI-MS: **headpiece-2´**, Boc-on, calculated m/z 5461.

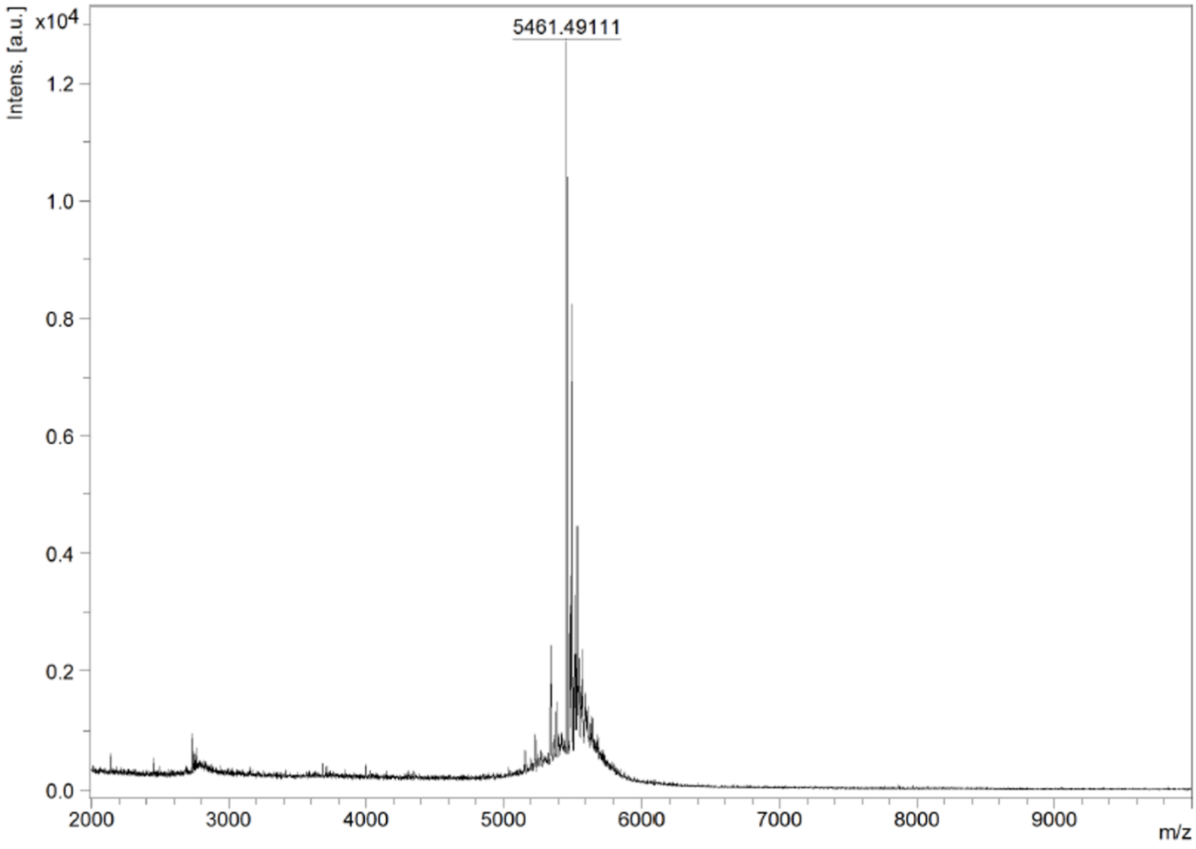

Zoomed-in MALDI-MS: **headpiece-2**, Boc-on, calculated m/z 5461. **headpiece-2,** Boc-on, calculated m/z 5489.

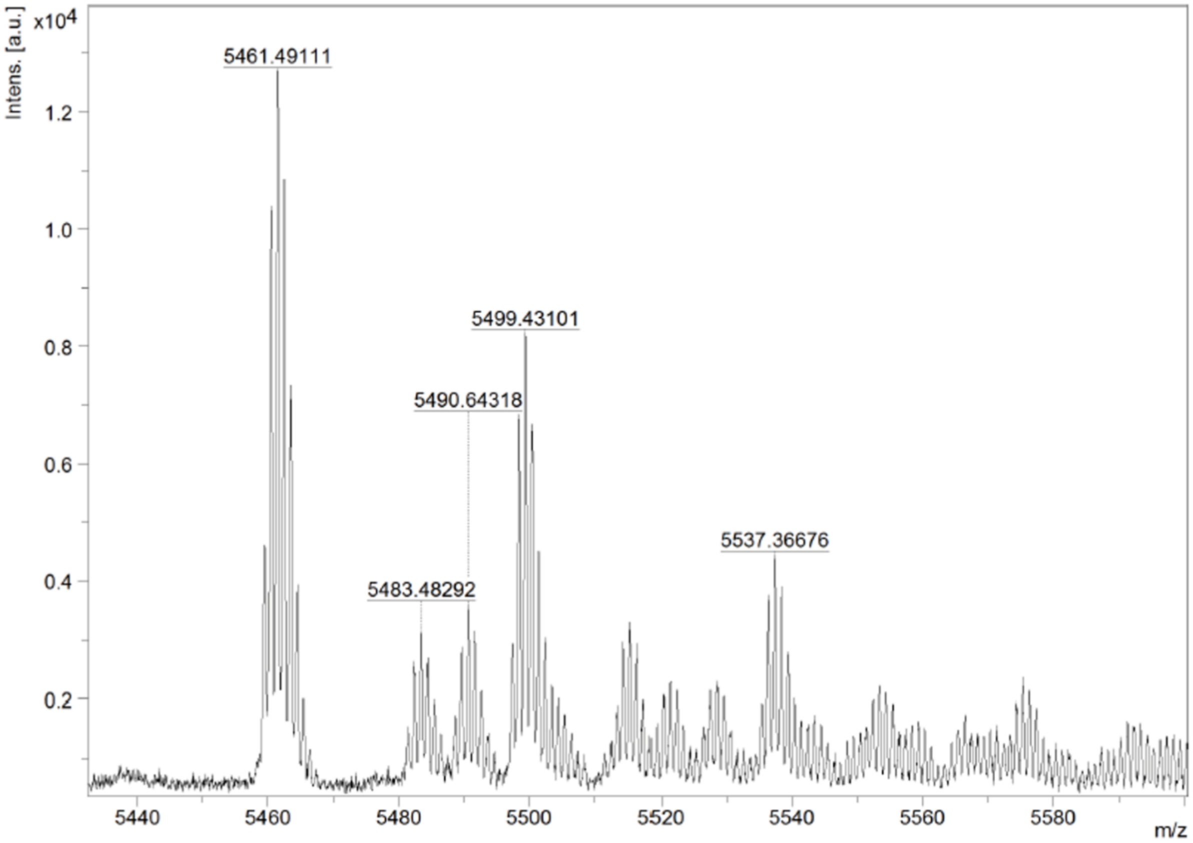

(f) dsDNA headpiece (headpiece-3)

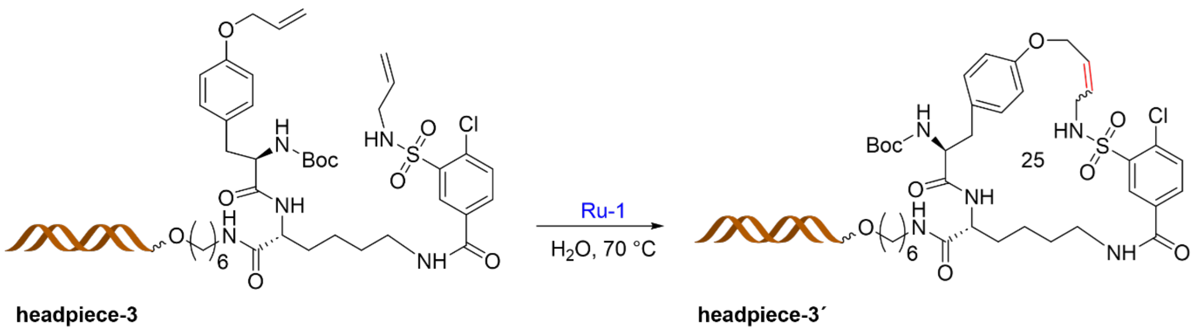

RP-HPLC chromatogram of **headpiece-3** after RCM in H_2O
9.0 min: **headpiece-3´**, 11.6 min: **headpiece-3**. 96% in situ yield, 835 pmol DNA-macrocycle product (isolated yield, 42%)

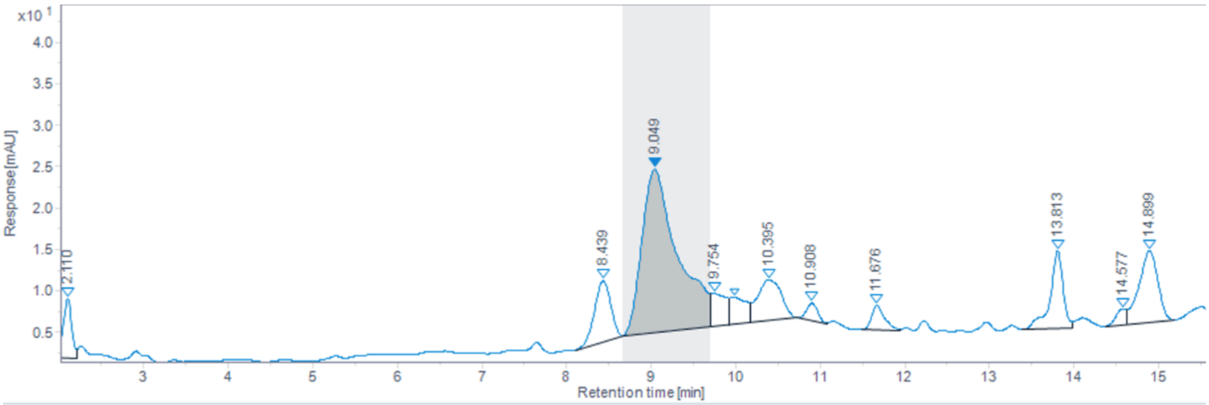

Injection Results

Peaks Summary

#	Name	Signal description	RT (min)	Area (mAU·s)	Area%	Height (mAU)	Height%	Amount
8		DAD1A,Sig=260,4 Ref=off	8.439	97.674	1.288	7.339	0.56	
9		DAD1A,Sig=260,4 Ref=off	9.049	581.081	7.661	19.701	1.51	
10		DAD1A,Sig=260,4 Ref=off	9.754	46.865	0.618	3.960	0.30	
11		DAD1A,Sig=260,4 Ref=off	9.987	44.098	0.581	3.259	0.25	
12		DAD1A,Sig=260,4 Ref=off	10.395	97.184	1.281	4.971	0.38	
13		DAD1A,Sig=260,4 Ref=off	10.908	20.106	0.265	2.138	0.16	
14		DAD1A,Sig=260,4 Ref=off	11.676	31.648	0.417	2.997	0.23	

Full MALDI-MS: **headpiece-3´**, Boc-on, calculated m/z 5597.

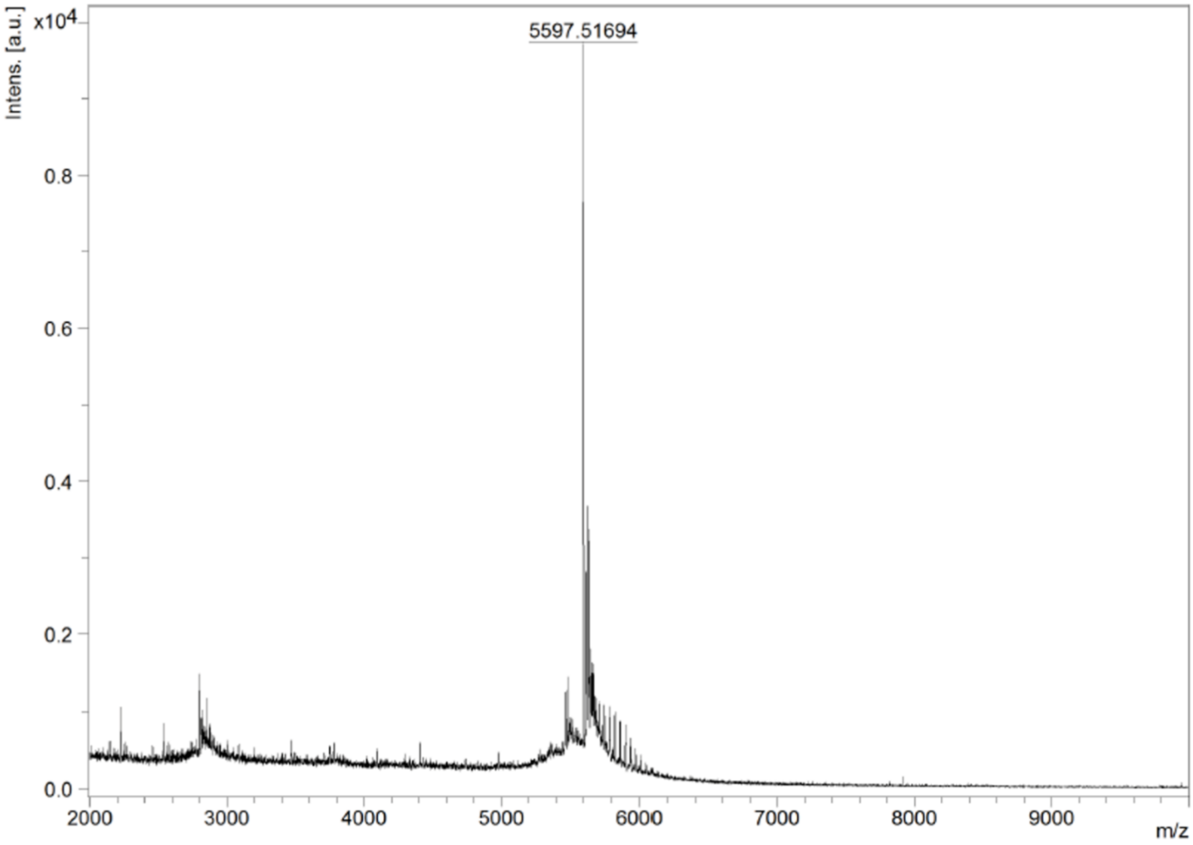

Zoomed-in MALDI-MS: **headpiece-3´**, Boc-on, calculated m/z 5597. **headpiece-3,** Boc-on, calculated m/z 5625.

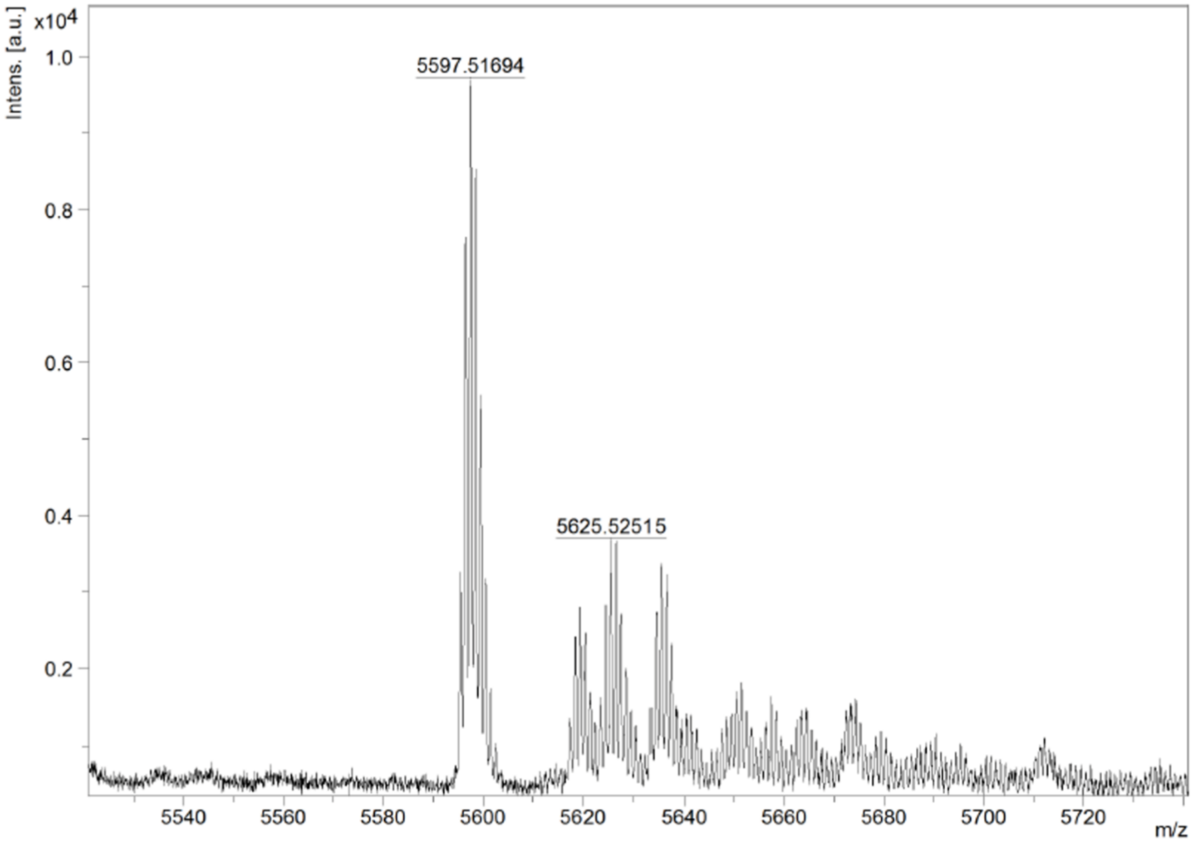

(g) 16mer native RNA with 50% purine content (AUGC-1)

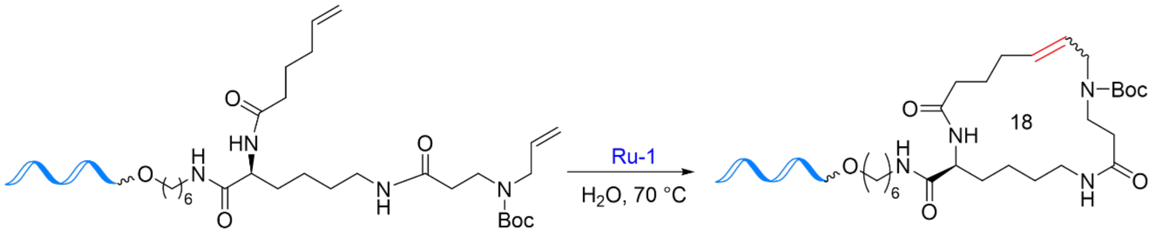

AUGC-1 **AUGC-1´**

RP-HPLC chromatogram of **AUGC-1** after RCM in H_2O
9.1 min: **AUGC-1´**, 11.2 min: **AUGC-1**. 63% in situ yield, 1.1 nmol RNA-macrocycle product (isolated yield, 55%).

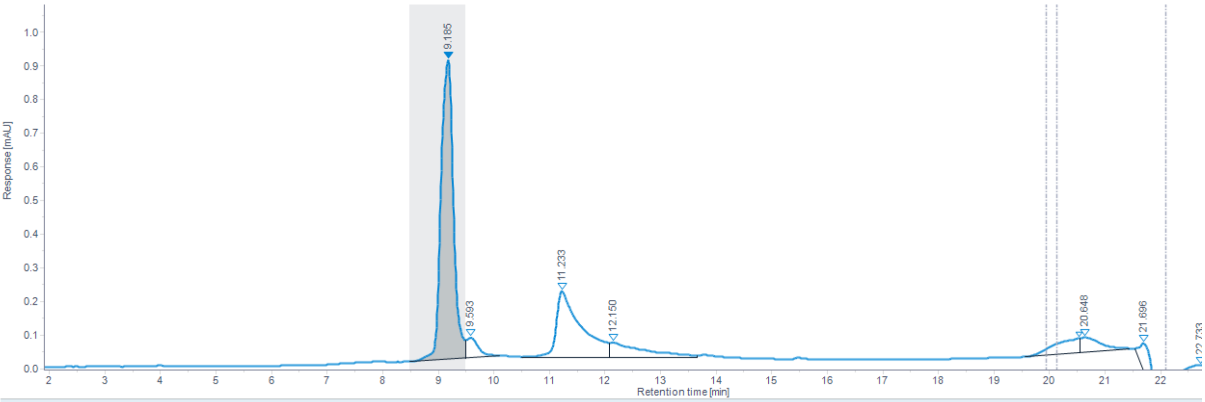

Injection Results

Peaks | Summary

#	Name	Signal description	RT (min)	Area (mAU·s)	Area%	Height (mAU)	Height%	Amount	Concentration	Start time (min)	End time (min)
1		DAD1A,Sig=260,4 Ref=off	1.189	100.786	2.799	17.163	8.72			1.068	1.255
2		DAD1A,Sig=260,4 Ref=off	1.311	70.852	1.968	18.189	9.24			1.261	1.358
3		DAD1A,Sig=260,4 Ref=off	1.412	56.329	1.564	8.873	4.51			1.358	1.463
4		DAD1A,Sig=260,4 Ref=off	1.530	18.265	0.507	2.136	1.09			1.463	1.568
5		DAD1A,Sig=260,4 Ref=off	9.185	1384.015	38.438	88.903	45.17			8.488	9.502
6		DAD1A,Sig=260,4 Ref=off	9.593	89.854	2.495	5.971	3.03			9.502	10.114
7		DAD1A,Sig=260,4 Ref=off	11.233	615.496	17.094	19.584	9.95			10.508	12.091
8		DAD1A,Sig=260,4 Ref=off	12.150	200.957	5.581	4.468	2.27			12.091	13.675

Full MALDI-MS: **AUGC-1´**, Boc-on, calculated m/z 5666.

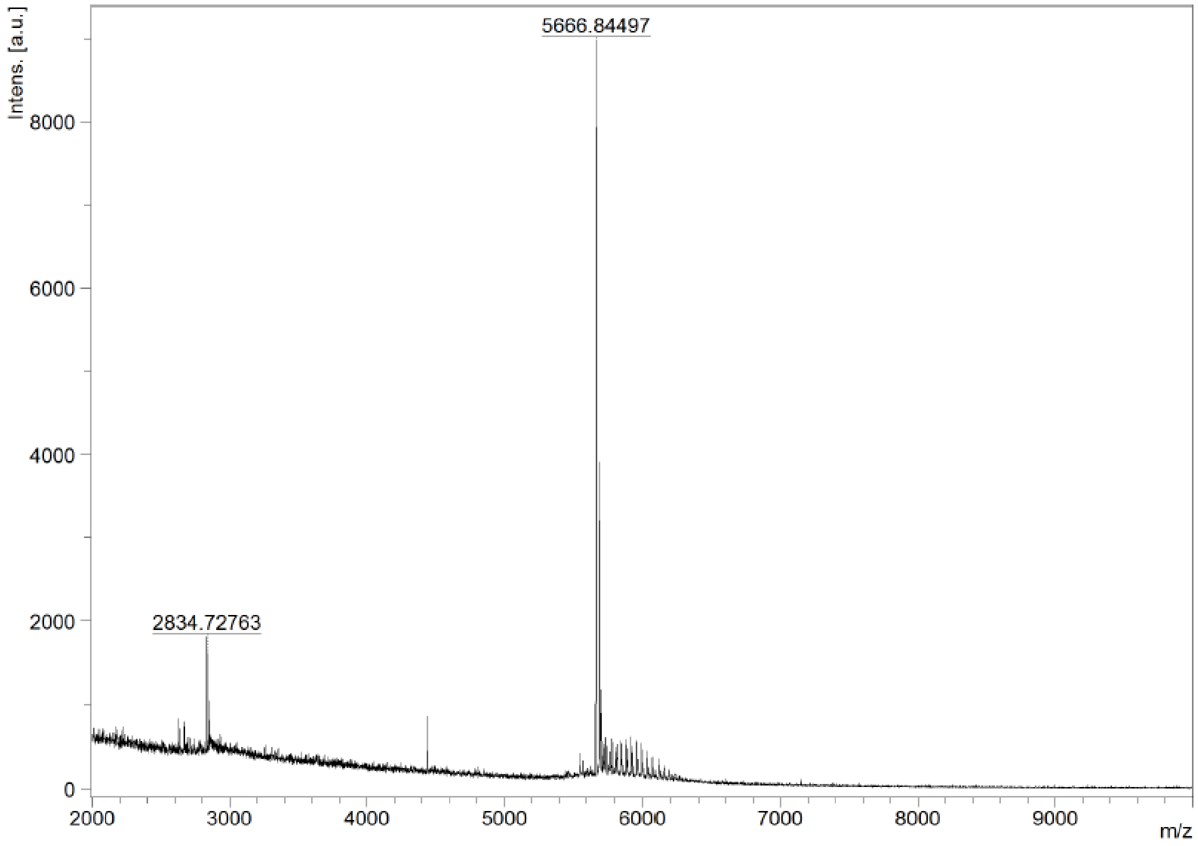

Zoomed-in MALDI-MS: **AUGC-1´**, Boc-on, calculated m/z 5666. **AUGC-1,** Boc-on, calculated m/z 5694.

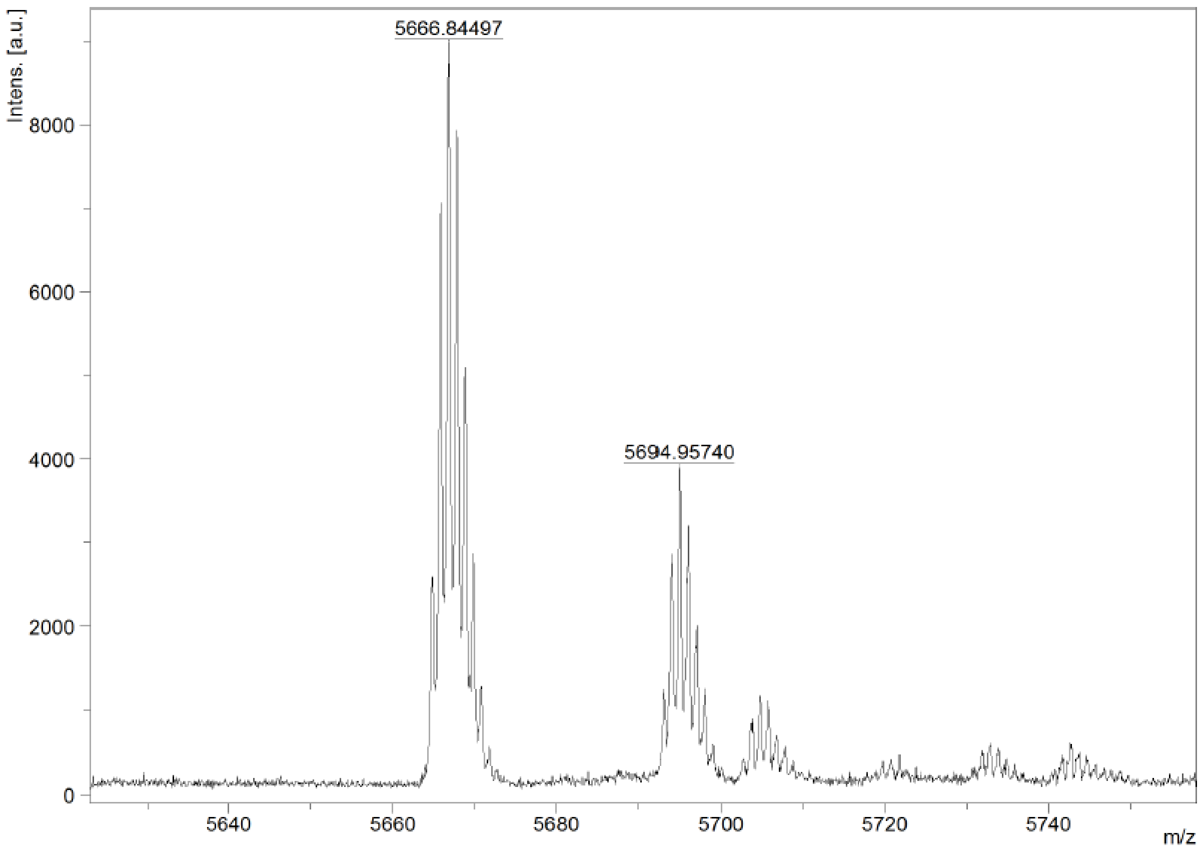

(h) 16mer native RNA with 75% purine content (purine75-AUGC-1)

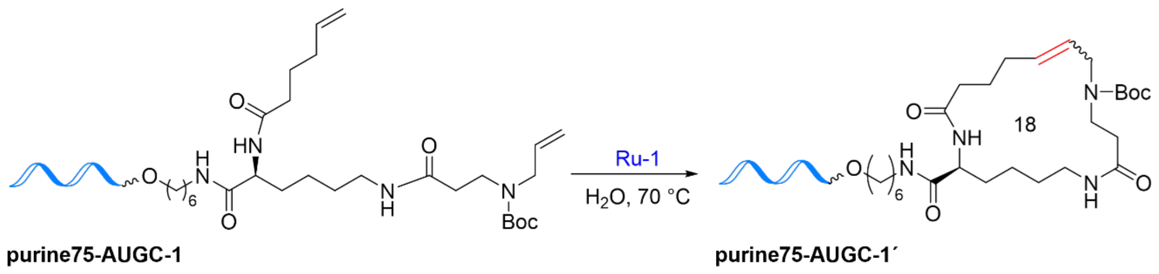

RP-HPLC chromatogram of **purine75-AUGC-1** after RCM in H_2O

9.1 min: **purine75-AUGC-1´**, 11.3 min: **purine75-AUGC-1**. 71% in situ yield, 1.16 nmol RNA-macrocycle product (isolated yield, 58%).

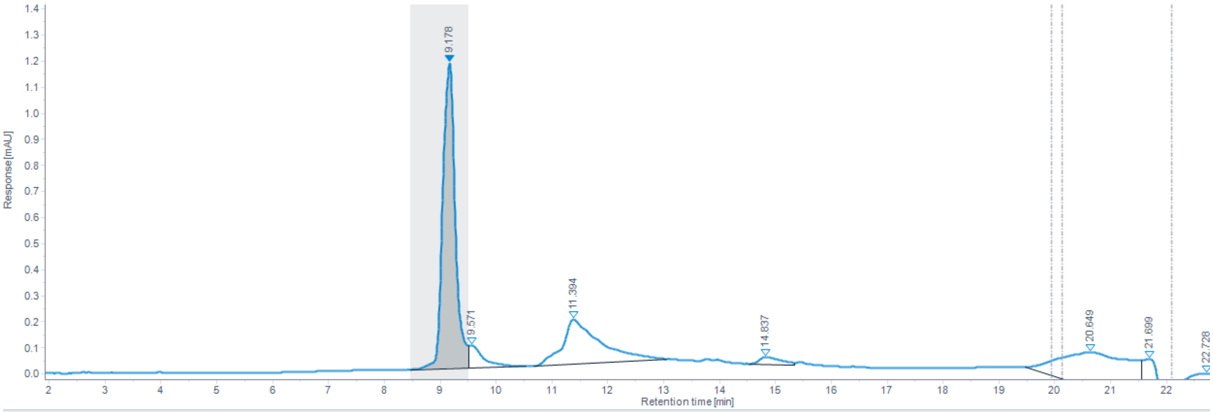

Injection Results

Peaks | Summary

#	Name	Signal description	RT (min)	Area (mAU·s)	Area%	Height (mAU)	Height%	Amount	Concentration	Start time (min)	End time (min)
3		DAD1A,Sig=260,4 Ref=off	1.220	83.549	1.434	24.591	8.15			1.198	1.280
4		DAD1A,Sig=260,4 Ref=off	1.327	91.266	1.566	24.276	8.04			1.282	1.372
5		DAD1A,Sig=260,4 Ref=off	1.425	115.017	1.974	15.498	5.14			1.372	1.503
6		DAD1A,Sig=260,4 Ref=off	1.529	32.537	0.558	10.308	3.42			1.503	1.555
7		DAD1A,Sig=260,4 Ref=off	1.608	57.773	0.991	6.951	2.30			1.555	1.763
8		DAD1A,Sig=260,4 Ref=off	9.178	1824.885	31.316	116.999	38.77			8.482	9.520
9		DAD1A,Sig=260,4 Ref=off	9.571	154.501	2.651	8.619	2.86			9.520	10.541
10		DAD1A,Sig=260,4 Ref=off	11.394	795.017	13.643	17.114	5.67			10.675	13.057
11		DAD1A,Sig=260,4 Ref=off	14.837	76.668	1.316	2.787	0.92			14.541	15.358

Full MALDI-MS: **purine75-AUGC-1´**, Boc-on, calculated m/z 5777.

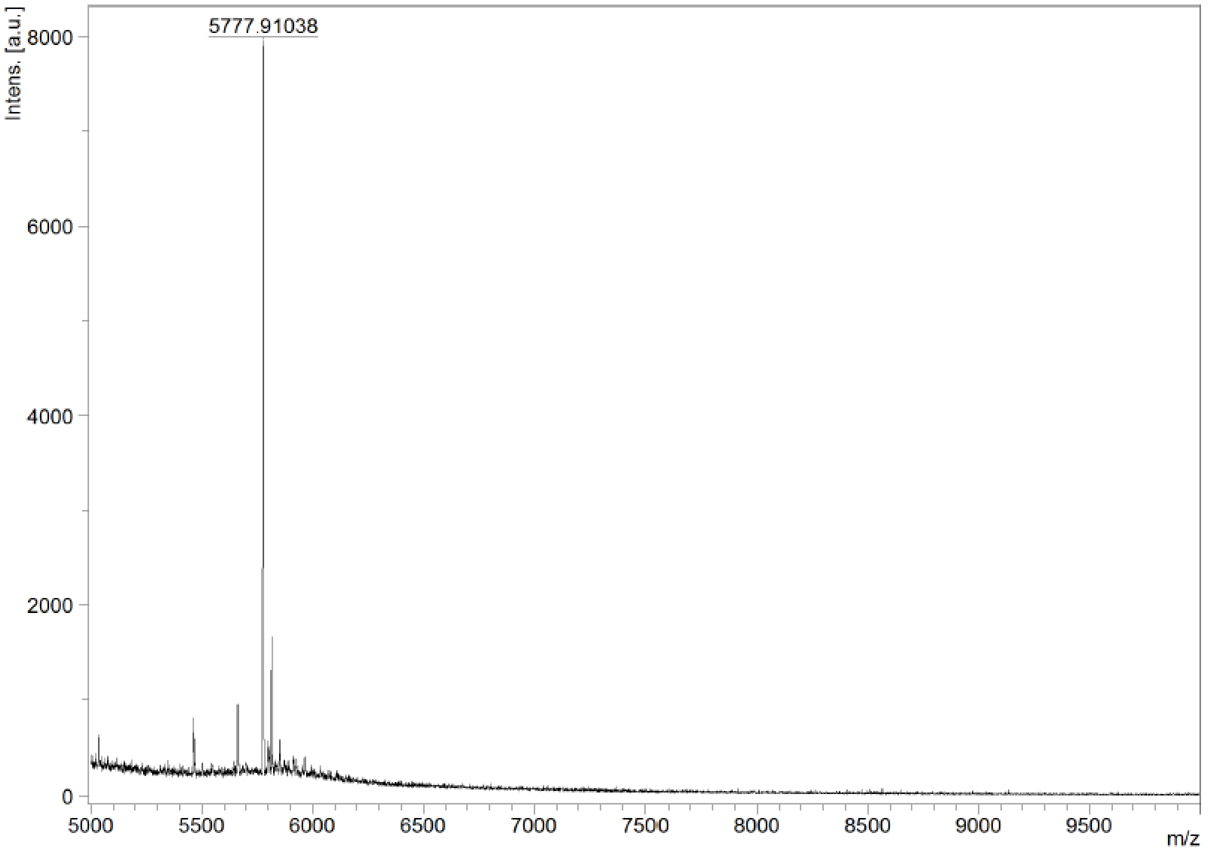

(i) 16mer native RNA with 75% purine content (purine75-AUGC-2)

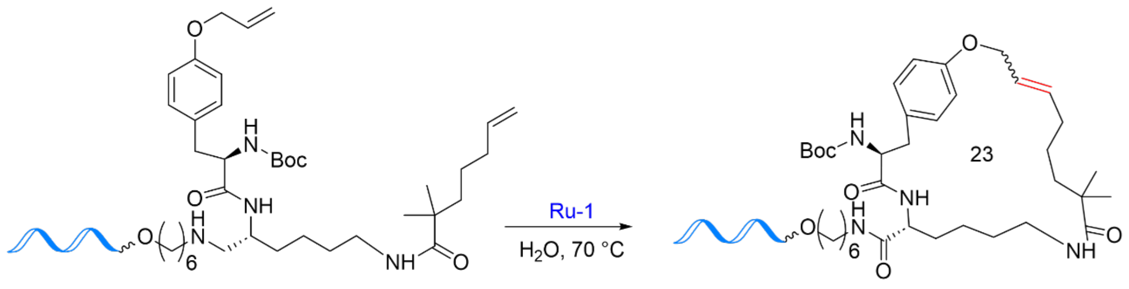

purine75-AUGC-2 **purine75-AUGC-2´**

RP-HPLC chromatogram of **purine75-AUGC-2** after RCM in H_2O
13.9 min: **purine75-AUGC-2´**, 15.1 min: **purine75-AUGC-2**. 84% in situ yield, 860 pmol RNA-macrocycle product (isolated yield, 43%).

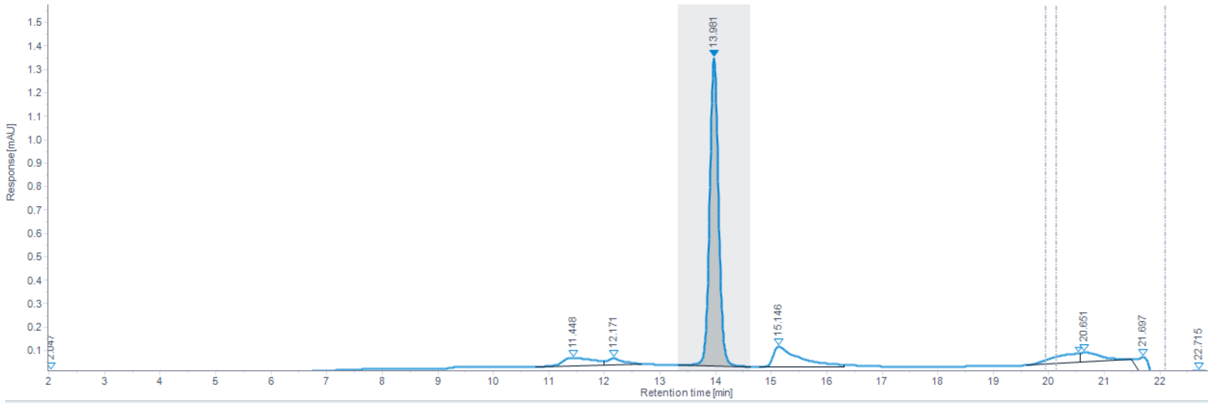

Injection Results

Peaks Summary

#	Name	Signal description	RT (min)	Area (mAU·s)	Area%	Height (mAU)	Height%	Amount	Concentration	Start time (min)	End time (min)
3		DAD1A,Sig=260,4 Ref=off	1.325	183.406	4.854	23.835	9.39			1.275	1.457
4		DAD1A,Sig=260,4 Ref=off	1.535	223.709	5.921	14.410	5.68			1.457	1.751
5		DAD1A,Sig=260,4 Ref=off	1.845	73.499	1.945	6.889	2.72			1.751	1.927
6		DAD1A,Sig=260,4 Ref=off	2.047	32.141	0.851	2.457	0.97			1.927	2.134
7		DAD1A,Sig=260,4 Ref=off	11.448	135.485	3.586	3.504	1.38			10.761	12.009
8		DAD1A,Sig=260,4 Ref=off	12.171	52.746	1.396	2.813	1.11			12.009	12.685
9		DAD1A,Sig=260,4 Ref=off	13.981	1467.507	38.841	131.397	51.79			13.335	14.641
10		DAD1A,Sig=260,4 Ref=off	15.146	280.301	7.419	8.558	3.37			14.788	16.318
11		DAD1A,Sig=260,4 Ref=off	20.537	130.542	3.455	3.998	1.58			19.595	20.567

Full MALDI-MS: **purine75-AUGC-2´**, Boc-on, calculated m/z 5911.

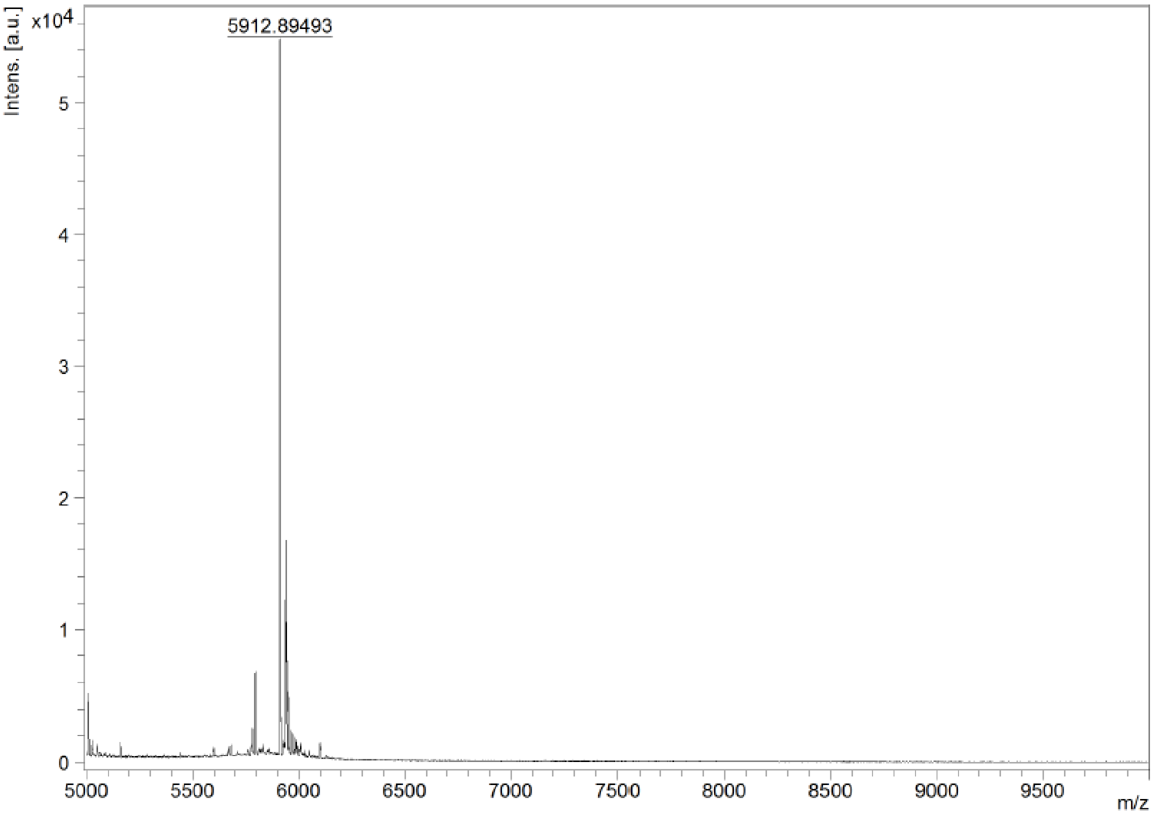

Zoomed-in MALDI-MS: **purine75-AUGC-2´**, Boc-on, calculated m/z 5911. **purine75-AUGC-2,** Boc-on, calculated m/z 5939.

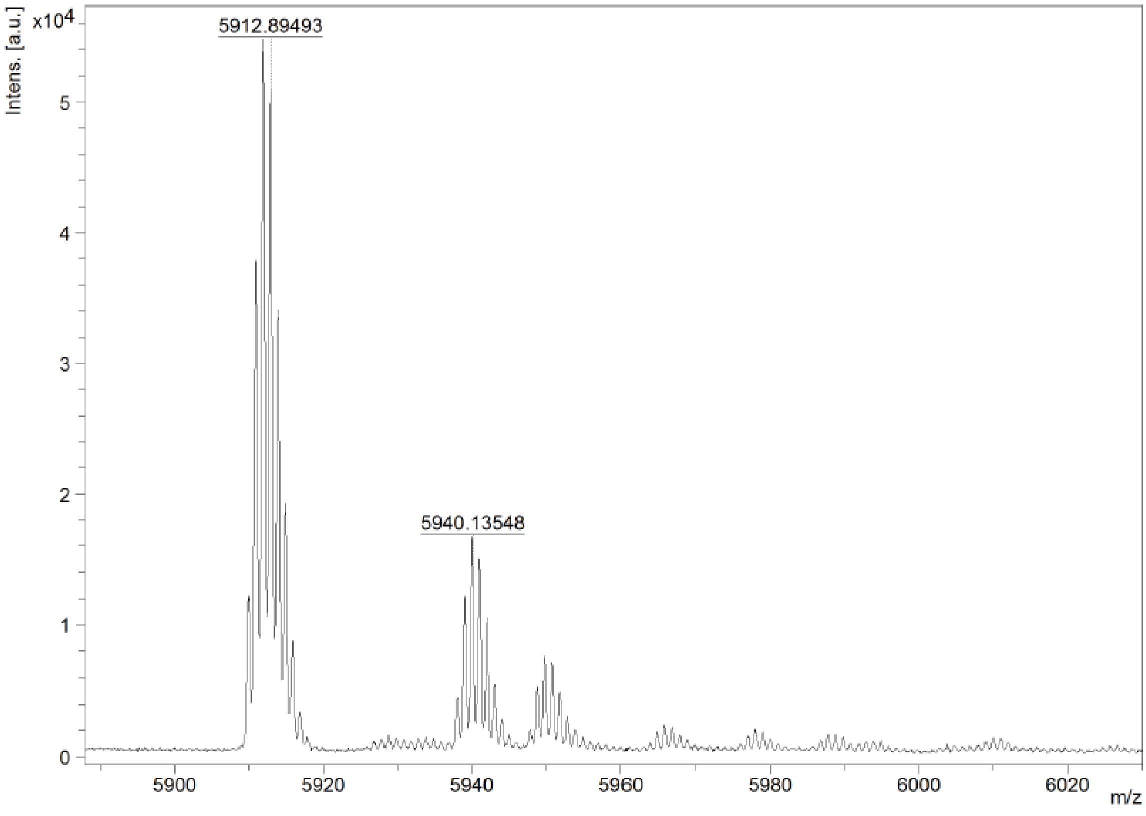

Figure S34. RP-HPLC traces and MALDI-MS spectra for RCM on dsDNA headpiece, DNA and RNA-tagged dienes with varied purine contents in H_2O.

Table S18: Sequences of DNA oligonucleotides for DNA ligation experiments.

DNA	Sequence (5´-3´)
Complementary strand to chemically modified DNA	(Phos)GATGGAGGAGAGCAGT
Complementary strand to native DNA	(Phos)GCGAATCGAGAGCAGT
Forward primer	ATGGCTGACCTGAGCTTCATACGGCATCAGAGC AGATTGTATCGACCCCTTTCTGACACCTGCCAC CAACGACAACAACTG
Complementary strand to forward primer	(Phos)TGTTGTCGTTGGTGGCAGGTGTCAGAAAG GGGTCGATACAATCTGCTCTGATGCCGTATGAA GCTCAGGTCAGCCAT
Reverse primer	ACCTTCCTCGTAACCCCACAGGTGTAGGAGAG GACATAGCGCACAAGCGACTGACAGCACCGTA TCGCCAAAATCACCGCCAGGT
Complementary strand to reverse primer	(Phos)GGCGGTGATTTTGGCGATACGGTGCTGTC AGTCGCTTGTGCGCTATGTCCTCTCCTACACCT GTGGGGTTACGAGGAAGGT
Complementary strand to hexa-T	AGGTGATGGAGGAGAGAAAAAA
Complementary strand	CTCTCCTCCATCACCT
Forward primer for sequencing	ATGGCTGACCTGAGCTTCATA
Reverse primer for sequencing	ACCTTCCTCGTAACCCCACA

Phos = 5´-phosphorylation

19. DNA damage assessment

After RCM, csDNA (**7DeATC-1´**) and native DNA (**ATGC-1´**) were 5´-phosphorylated as described in General Procedure 3. hexa-T (**hexa-T-1´**), **7DeATC-1´** and **ATGC-1´** were then ligated as described in General Procedure 4 based on the following ligation strategies (**Fig. S20**). Afterwards, the **7DeATC-1´** and **ATGC-1´** ligation products were purified using an E-Gel™ Power Snap Electrophoresis System. All DNAs before and after ligation were loaded into 12% polyacrylamide gel for analysis as described in General Procedure 5. The purified **7DeATC-1´** and **ATGC-1´** ligation products were sequenced by Sanger sequencing using TubeSeq Supreme.

(a)

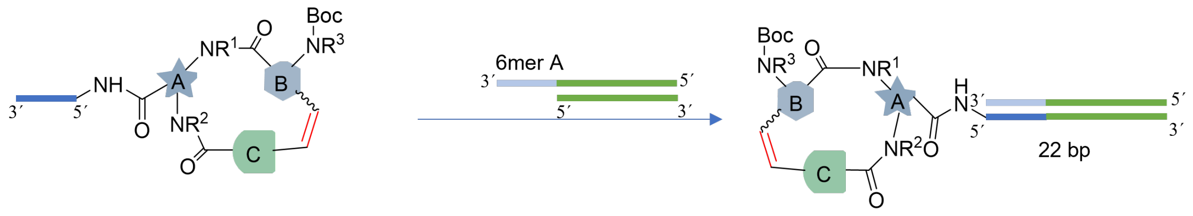

(b)

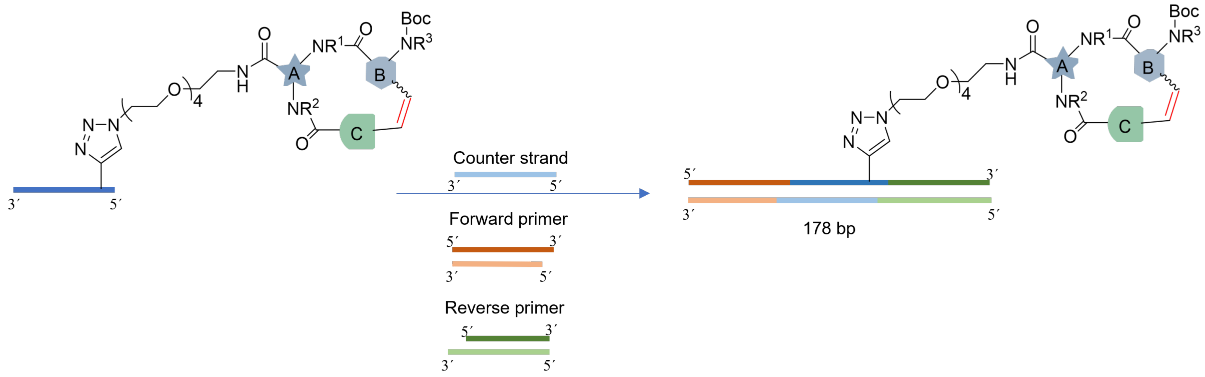

Figure S35. **Two ligation strategies. (a)** Ligation method for **hexa-T-1'**. **(b)** Ligation strategy for **7DeATC-1'** and **ATGC-1'**. DNA segments exposed to **Ru-1** are in dark blue. **hexa-T-1´**was ligated to a 16-bp dsDNA with a 6 nt hexa-A overhang complementary to the hexa-T sequence. **7DeATC-1′** and **ATGC-1´** were annealed to 16 nt complementary strands containing 4 nt overhangs, and ligated to two 81 bp long primer sequences.

20. Supplementary references

1. Sanford, M.S., Love J.A., Grubbs R.H. A Versatile Precursor for the Synthesis of New Ruthenium Olefin Metathesis Catalysts. *Organometallics* **20**, 5314–5318 (2001).
2. Higman, C.S., Nascimento D., Ireland B.J., Audorsch S., Bailey G.A., Fogg D.E. Chelate-Assisted Ring-Closing Metathesis: A Strategy for Accelerating Macrocyclization at Ambient Temperatures. *J. Am. Chem. Soc.* **140**, 1604−1607 (2018).
3. Blanco, C.O., Castellanos R.R., Fogg D.E. Anionic Olefin Metathesis Catalysts Enable Modification of Unprotected Biomolecules in Water. *ACS Catal.* **14**, 11147–11152 (2024).
4. Escudero, J., Bellosta V., Cossy J. Rhodium-Catalyzed Cyclization of *O*,ω-Unsaturated Alkoxyamines: Formation of Oxygen-Containing Heterocycles. *Angew. Chem., Int. Ed.* **57**, 574–578 (2018).
5. Blacquiere, J.M., Jurca T., Weiss J., Fogg D.E. Time as a Dimension in High-Throughput Homogeneous Catalysis. *Adv. Synth. Catal.* **350**, 2849–2855 (2008).
6. Garakani, T.M., Sauer D.F., Mertens M.A.S., Lazar J., Gehrmann J., Arlt M., Schiffels J., Schnakenberg U., Okuda J., Schwaneberg U. FhuA-Grubbs-Hoveyda Biohybrid Catalyst Embedded in a Polymer Film Enables Catalysis in Neat Substrates. *ACS Catal.* **10**, 10946–10953 (2020).
7. Lu, X., Fan L., Phelps C.B., Davie C.P., Donahue C.P. Ruthenium Promoted On-DNA Ring-Closing Metathesis and Cross-Metathesis. *Bioconjugate Chem.* **28**, 1625–1629 (2017).
8. Monty, O.B.C., Nyshadham P., Bohren K.M., Palaniappan M., Matzuk M.M., Young D.W., Simmons N. Homogeneous and Functional Group Tolerant Ring-Closing Metathesis for DNA-Encoded Chemical Libraries. *ACS Comb. Sci.* **22**, 80-88 (2020).
