# Supplementary material for: On-oligonucleotide olefin metathesis in water
Source: Nat Commun. 2026 May 12;17:6329. doi: 10.1038/s41467-026-72995-4 (PMC13377165; doi:10.1038/s41467-026-72995-4)
Supplement: Supplementary file 2 — Transparent Peer Review File [file 41467_2026_72995_MOESM2_ESM.pdf]

nature portfolio

Peer Review File

On-Oligonucleotide Olefin Metathesis in Water

Corresponding Author: Professor Andreas Brunschweiger

This file contains all reviewer reports in order by version, followed by all author rebuttals in order by version.

Version 0:

Reviewer comments:

Reviewer #1

(Remarks to the Author)
In their work “On-oligonucleotide olefin metathesis in water”, Zhang et al. reported the synthesis of on-DNA stapled peptides via ring-closing metathesis (RCM) using a fully water-soluble Ru-based catalyst in an entirely aqueous medium. This represents an important advance, as it demonstrates the feasibility of performing RCM directly on nucleic acid conjugates under water conditions. The catalyst, previously developed by the same group for aqueous alkene metathesis, was modified with a cyclic alkylamino carbene (CAAC) ligand. The CAAC motif enhances catalyst stability and suppresses undesired side reactions such as C=C bond migration, which had been problematic with earlier Ru–NHC catalysts. In addition, the catalyst bears a sulfonate substituent, providing excellent water solubility and improving DNA/RNA compatibility through electrostatic repulsion. Interestingly, the authors found that the reaction proceeds best under acidic conditions (pH 2–5), which preserve catalyst activity while maintaining nucleic acid integrity. Under optimized conditions, they achieved up to 60% conversion with ~80% DNA recovery within 30 minutes at 70 °C, with 10 eq. of Ru-catalyst, even in the presence of extremely high salt concentrations (80,000 eq. NaCl and 80,000 eq. $NHEt_3Cl$).

For the first time, this work applied RCM beyond DNA to RNA-conjugated substrates, which also underwent successful macrocyclization under these aqueous conditions. The study further confirmed the stability of both the Ru catalyst and the oligonucleotides in high-salt aqueous media. Although this is the first report of on-oligonucleotide alkene metathesis in a completely aqueous system with a previously reported Ru-Catalyst, the exclusive reliance on acidic conditions (pH 2–5) is interesting. Since oligonucleotides do not suffer from degradation under neutral or slightly basic pH, it would be important to test whether the reaction could also be performed under those conditions. The development of RCM conditions for on-DNA applications was first reported by Lu and (ref. 9) later by Young (ref. 10). The methodology reported in the current manuscript, while nicely developed, does not significantly expand the application of on-DNA RCM beyond these seminal papers. If it does, perhaps a side-by-side comparison should have been presented or the application of the technology to a full library and target selection. Therefore, the methodological advance in chemistry is viewed as incremental and to likely be of interest only to a specialized audience. Thus, due to limited novelty, the manuscript by Zhang et al. would not likely be of interest to the broad readership of Nature Communications and should not be accepted here. The manuscript is far more suited to a specialized chemistry journal such as Chemical Communications or Organic Letters.

Specific comments:
1. For assessing the stability of Ru-1 pre-catalyst, the authors reported results in the presence of native DNA at pH 5. What about stability in basic or buffered conditions, where oligonucleotides are known to be more stable? Prior work (ACS Comb. Sci. 2020, 22, 80–88) reported stability issues with this type of catalyst in basic pH. What is its long-term stability here?
2. While examining deamination of deoxyadenosine, ~43% deamination was observed with Ru-1 in the absence of ethylene. What is the mechanistic role of ethylene in this process? Moreover, in the DNA experiments, the reactions were performed without ethylene. Why was no deamination observed with native DNA containing a single dA residue? If the DNA had multiple dA residues, what would happen?
3. More DNA-degradation was observed in the optimized condition while kept for longer time, did the authors tried reaction with lower temp, or higher pH for longer time reaction for lower DNA degradation?
4. DNA backbone stability was measured using a 16-mer poly-thymidine strand at room temperature in 0.4 M NaCl. Thymidine appeared chemically stable under these conditions. However, what would be the backbone stability at 70 °C for poly-T, as well as for a mixed-sequence 16-mer DNA or RNA?
5. When applying the optimized conditions to RNA substrates, the recovery was only ~30%. Why is the recovery so low? Did the authors attempt any RNA-specific optimization?
Actionable Suggestions

1. pH/Buffer screen: Test pH 5–8 in phosphate, HEPES, and MES at matched ionic strength (±NaCl/NHEt3Cl). Report conversion, DNA/RNA recovery, and catalyst speciation.
2. Time/temperature minimization: Explore RT with longer times or brief to reduce nucleic acid damage.
3. Ethylene control set: Run on-DNA RCM ±ethylene (or alternate olefin scavengers) to directly test deamination suppression in the conjugate context.
4. Sequence scope: Evaluate A-rich vs A-poor sequences to probe deamination liability; include RNA with varied purine content.

Reviewer #2

(Remarks to the Author)
This manuscript applied the anionic, water-soluble ruthenium catalyst Ru-1 for the on-DNA RCM reaction. High compatibility is demonstrated with different oligonucleotide tags including DNA and RNA oligomers and a chemically-stabilized DNA congener. Synthetic useful conversions and recovery of the nucleic acid was achieved with this novel Ru catalyst. This method demonstrated the application of RCM for the encoded library synthesis. This manuscript is recommended to publish after the following questions are addressed.

1, The authors has demonstrated its application for the single strand oligo, whether this method could also applicable for the dsDNA, it's better for the authors also to explore this application.

2, It's better for the authors to explore whether this method is applicable for the plate format synthesis which is important for the encoded library synthesis.

3, It's better for the authors to optimize and explore a little more about the function of NaCl and ammonium salt for this reaction.

Reviewer #3

(Remarks to the Author)
The authors describe the synthesis of macrocyclic compounds anchored on short DNA or RNA fragments using ring-closing metathesis (RCM) carried out in aqueous media. This study paves the way toward the preparation of constructs applicable in targeted therapies, where nucleic acids can act as carriers for prodrugs or drugs.

The work is very well thought out and clearly required active collaboration between chemists and molecular biologists. The authors employed a well-known catalyst, AquaMet, as well as a second complex based on a CAAC ligand decorated with a sulfonate tag, recently introduced by the Fogg group. They then examined the stability of these complexes in the presence of nucleotides and their activity in a model RCM reaction, with Ru1 performing significantly better in both cases. Importantly, they also investigated the reverse relationship—namely, the stability of nucleotides and nucleic acid fragments in the presence of both catalysts. While the data for AquaMet appear to be missing in the first case (these should be added), both DNA and RNA fragments proved relatively stable in the presence of Ru1, especially when NaCl was included. Salt also had a positive, albeit weaker, effect on stability in the presence of AM.

Having established stability and optimized reaction conditions, the authors successfully obtained a series of macrocyclic compounds anchored on nucleic acid fragments, achieving satisfactory yields and DNA recovery (this term should be clarified for the reader).

This work is groundbreaking. Although it employs a very high catalyst loading (10 equiv. relative to substrate), which limits its immediate practical applications on a larger scale, it opens promising avenues for further discoveries.

Overall, I have very few concerns. I would only recommend clarifying some biological terminology (e.g., "DNA ligation") so that readers with limited expertise in molecular biology can also fully appreciate the significance of this study.

Version 1:

Reviewer comments:

Reviewer #2

(Remarks to the Author)
The authors have addressed all my previous concerns, current manuscript is recommended to publish at nature communications

Reviewer #4

(Remarks to the Author)
I read with attention and several times this manuscript, as well as the reports of the Referees and the point-to-point answers

by the authors. The authors have done a great job addressing all three reviewers' comments, adding new experiments and expanding the discussion to clarify key points. Overall, the authors have established an efficient RCM reaction for both DNA- and RNA-conjugated substrates using an anionic, water-soluble ruthenium catalyst. Given the high conversion and recovery achieved with DNA templates, and particularly as this represents the first successful application of the RCM to RNA modification, a long-standing challenge in nucleic acid chemistry, the prior on-DNA RCM methodology does not compromise the novelty or application potential of the present work. Although the reported RCM methodology will have great application potential in multidisciplinary fields, the manuscript's presentation is too specialized. To enhance accessibility for the broad readership of Nature Communications, the authors are encouraged to revise the text, particularly the Introduction and Summary sections, to clarify key concepts and explicitly articulate the broader implications of the findings. These revisions can be incorporated during the final production stage; no additional round of peer review is required.

Page 1, In the Abstract, a sentence explicitly underscoring the RCM for RNA modification could be added.

In the Introduction or Summary section, a concise yet comprehensive discussion of the application potential of the developed RCM reaction in RNA research should be incorporated.

Page 1, line 21: Several recent reviews (https://doi.org/10.1016/j.trechm.2025.08.008; https://doi.org/10.1016/j.apsb.2023.10.001; https://doi.org/10.1039/D5CC02904J) on DEL chemistry development could be included and discussed.

Page 1, lines 28–40: A concise yet comprehensive summary of recent advances in DNA-encoded macrocyclic libraries, and the key challenges currently impeding their construction and broader application, should be incorporated into the Introduction (https://doi.org/10.1039/D1CB00161B).

Page 2, line 53: In Fig. 1a, the therapeutic indications and clinical development stages of the depicted macrocyclic compounds could be explicitly annotated to enhance accessibility for the broad, interdisciplinary readership of Nature Communications.

Page 2, line 53: In Fig. 1d, RNA-compatible acidity could also be added.

Open Access This Peer Review File is licensed under a Creative Commons Attribution 4.0 International License, which permits use, sharing, adaptation, distribution and reproduction in any medium or format, as long as you give appropriate credit to the original author(s) and the source, provide a link to the Creative Commons license, and indicate if changes were made.
In cases where reviewers are anonymous, credit should be given to 'Anonymous Referee' and the source.
The images or other third party material in this Peer Review File are included in the article's Creative Commons license, unless indicated otherwise in a credit line to the material. If material is not included in the article's Creative Commons license and your intended use is not permitted by statutory regulation or exceeds the permitted use, you will need to obtain permission directly from the copyright holder.
To view a copy of this license, visit https://creativecommons.org/licenses/by/4.0/

REVIEWER 1: Comments. In their work “On-oligonucleotide olefin metathesis in water”, Zhang et al. reported the synthesis of on-DNA stapled peptides via ring-closing metathesis (RCM) using a fully water-soluble Ru-based catalyst in an entirely aqueous medium. This represents an important advance, as it demonstrates the feasibility of performing RCM directly on nucleic acid conjugates under water conditions. The catalyst, previously developed by the same group for aqueous alkene metathesis, was modified with a cyclic alkylamino carbene (CAAC) ligand. The CAAC motif enhances catalyst stability and suppresses undesired side reactions such as C=C bond migration, which had been problematic with earlier Ru–NHC catalysts. In addition, the catalyst bears a sulfonate substituent, providing excellent water solubility and improving DNA/RNA compatibility through electrostatic repulsion. Interestingly, the authors found that the reaction proceeds best under acidic conditions (pH 2–5), which preserve catalyst activity while maintaining nucleic acid integrity. Under optimized conditions, they achieved up to 60% conversion with ~80% DNA recovery within 30 minutes at 70 °C, with 10 eq. of Ru-catalyst, even in the presence of extremely high salt concentrations (80,000 eq. NaCl and 80,000 eq. NHEt3Cl).

For the first time, this work applied RCM beyond DNA to RNA-conjugated substrates, which also underwent successful macrocyclization under these aqueous conditions. The study further confirmed the stability of both the Ru catalyst and the oligonucleotides in high-salt aqueous media.

1-1. Although this is the first report of on-oligonucleotide alkene metathesis in a completely aqueous system with a previously reported Ru-Catalyst, the exclusive reliance on acidic conditions (pH 2–5) is interesting. Since oligonucleotides do not suffer from degradation under neutral or slightly basic pH, it would be important to test whether the reaction could also be performed under those conditions.

Reply. Use of neutral or basic pH is precluded by catalyst instability. RCM in water is a race between metathesis and decomposition, and the catalysts do not survive long enough under these conditions to effect cyclization. High chloride concentrations aid in retarding water-induced catalyst degradation, but are insufficient for challenging metathesis reactions. Slightly acidic conditions are necessary to stabilize the dichlororuthenium complexes against decomposition into metathesis-inactive hydroxide complexes.
Action 1-1. We add this context, with the important qualifier that *DNA-compatible*, weakly acidic conditions suffice to preserve catalyst integrity, as demonstrated in our recent speciation study (JACS 2025; ref 20).

1-2. The development of RCM conditions for on-DNA applications was first reported by Lu and (ref. 9) later by Young (ref. 10). The methodology reported in the current manuscript, while nicely developed, does not significantly expand the application of on-DNA RCM beyond these seminal papers. If it does, perhaps a side-by-side comparison should have been presented or the application of the technology to a full library and target selection. Therefore, the methodological advance in chemistry is viewed as incremental and to likely be of interest only to a specialized audience. Thus, due to limited novelty, the manuscript by Zhang et al. would not likely be of interest to the broad readership of Nature Communications and should not be accepted here. The manuscript is far more suited to a specialized chemistry journal such as Chemical Communications or Organic Letters.

Reply. We respectfully disagree. As the first report of metathesis of dienes appended to either RNA or ss-DNA, this work significantly expands the scope of oligonucleotide metathesis, while also greatly improving the practicality of such methodologies for ds-DNA.
Action 1-2. We illustrate the superiority of the new methodology by undertaking the suggested side-by-side comparison with the literature methods, using **hexa-T**, **ATGC-1**, **7DeATC-1**, **AUGC-1** and **2'-OMe AUGC-1**. The experimental data, with in situ and isolated yields, and HPLC and MALDI spectra, are added to the SI. We note in the text the following specific advances:

(a) **Superior yields**. Under the GSK conditions, the solution became biphasic after adding $MgCl_2$, and the HPLC chromatograms showed considerable decomposition, with neither starting materials nor the cyclic products. Under the Young/Simmons conditions, 32% isolated yield was achieved for **hexa-T-1'**, but only ca. 10% for **ATGC-1',** and **7DeATC-1',** and negligible oligonucleotide recovery for the RNA experiments, for which the products could be observed by MALDI-MS, but not by HPLC.

(b) **Superior library fidelity**. We now point out that double-bond isomerization leading to ring-contracted products is evident from the mass spectra in the Young/Simmons report (Supporting information, p. 29 ff; no supporting information with spectra was provided for the GSK publication), consistent with prior reports from pharma (new citations added). We also note that the CAAC catalyst **Ru-1** – in contrast with the Ru-NHC catalysts employed in the prior work – does not form isomerization-active species upon decomposition. That is, the choice of a CAAC carbene prevents formation of ring-contracted products, an advance critical for library fidelity and integrity.

(c) **Electrostatic protection of DNA**. We now point out more explicitly that anionic catalyst **Ru-1**, uniquely, incorporates electrostatic repulsion to inhibit docking of degraded Ru species at the oligonucleotide backbone of DNA or RNA. No such protection is present in prior catalysts. Because all of the Ru catalysts form cationic species in water, their degradation products are expected to bind to DNA, triggering reactions that compromise barcode integrity. Indeed, the GSK study utilizing neutral **GIII** noted that DNA decomposition occurred, although no sequencing data were provided in either report. In short, the prior work – although unquestionably groundbreaking – was limited by the nature of the catalysts accessible at the time.

Specific comments:
1-3. For assessing the stability of Ru-1 pre-catalyst, the authors reported results in the presence of native DNA at pH 5. What about stability in basic or buffered conditions, where oligonucleotides are known to be more stable? Prior work (ACS Comb. Sci. 2020, 22, 80−88) reported stability issues with this type of catalyst in basic pH. What is its long-term stability here?

Action 1-3. As noted in Response 1-1, use of basic pH causes immediate decomposition (ref 20). We now make this more explicit, and note that we chose acidic conditions and an ammonium chloride buffer for their compatibility with ruthenium metathesis catalysts.

1-4. While examining deamination of deoxyadenosine, ~43% deamination was observed with Ru-1 in the *absence of ethylene. What is the mechanistic role of ethylene in this process*? Moreover, in the DNA experiments, the reactions were performed without ethylene.

Action 1-4. We now state more clearly that an ethylene atmosphere was added in these model reactions to deliberately generate the most reactive ruthenium species present during metathesis. We also note that these experiments are much more aggressive than would be attained in the DNA experiments, where the proportion of ethylene is 10% relative to **Ru-1** (given 10 equiv Ru per diene). Finally, we comment that the absence of deamination or depurination in these model experiments implies that these side-reactions are irrelevant under DEL conditions, where the ethylene concentration is limited not only by stoichiometry, but by the low solubility of ethylene at elevated temperatures (see new ref. 30).

1-5. Why was no deamination observed with native DNA containing a single dA residue? If the DNA had multiple dA residues, what would happen?

Reply. The absence of deamination is consistent with the inference in Response 1.4. Nevertheless, to assess the impact of multiple dA residues, we further investigated the impact of DNA oligomers with high versus low purine content.
Action 1-5. Experiments with **purine75-ATGC** and **purine25-ATGC** delivered in situ yields of 66–95% with 53–68% isolated yields of the DNA-macrocycle products (**Fig. 7a**). Mass spectrometric analysis revealed no depurination or ring-contracted side-products (**Supplementary Fig. S34**). See also Response 2-1.

1-6. More DNA-degradation was observed in the optimized condition while kept for longer time, did the authors tried reaction with lower temp, or higher pH for longer time reaction for lower DNA degradation?

Reply. Higher pH is precluded by catalyst degradation, as explained in Action 1-1. We examined the impact of temperature as suggested, via RCM on native DNA **ATGC-1** at 60 °C, 50 °C, 40 °C and 25 °C for 30 min and 60 min. No macrocycle products could be detected by HPLC analysis.
Action 1-6. We expand on our discussion of optimization strategies to note that at temperatures below 70 °C, degradation out-competes RCM. These data are added to the Supplementary Information (Table S17, entries 8-15). We also cite our original report which commented on the low yields obtained at RT for **Ru-1.**

1-7. DNA backbone stability was measured using a 16-mer oligo-thymidine strand at room temperature in 0.4 M NaCl. Thymidine appeared chemically stable under these conditions. However, what would be the backbone stability at 70 °C for poly-T, as well as for a mixed-sequence 16-mer DNA or RNA?

Action 1-7. We conducted the suggested tests of nucleic acid stability. The protective effect of NaCl on nucleic acid integrity is reproduced in experiments in which mixed-sequence DNA and RNA 16mers were incubated with 10 eq of **Ru-1** at room temperature and at 70°C. In contrast, **AM** caused massive nucleic acid degradation under the same conditions (**Supplementary Tables S5-S9, Figs. S12-S21**).

1-8. When applying the optimized conditions to RNA substrates, the recovery was only ~30%. Why is the recovery so low? Did the authors attempt any RNA-specific optimization?

Reply. Given the notorious difficulties in working with RNA, the fact that this is the first report of on-RNA metathesis, and a rare example of transition-metal catalysis on RNA, we respectfully suggest that the yield of 30% should be recognized as a breakthrough. Nevertheless, we carried out further experiments with RNA sequences containing higher proportions of purines. Unexpectedly, these give higher yields, perhaps because they are precipitated more readily, which expand the scope with respect to RNA substrate scope and product recovery.

Action 1-8. We add and discuss experiments on RNA oligomers with equal proportions of each nucleobase **(AUGC**), and with purine-rich or purine-poor RNA (**purine75-AUGC** and **purine25-AUGC**, respectively). In situ yields were 60–84%, while isolated yields of the RNA-macrocycle product ranged from 30% (**purine25-AUGC**) to 43-58% yield (**Fig. 7b, Supplementary Fig. S33**). No depurination was detected (**Supplementary Fig. S34**).

Actionable Suggestions *[Author note: these suggestions expand on those in the comments above]*
1-9. pH/Buffer screen: Test pH 5–8 in phosphate, HEPES, and MES at matched ionic strength (±NaCl/$NHEt_3Cl$). Report conversion, DNA/RNA recovery, and catalyst speciation.
Action 1-9a. We add a section explaining in more detail the choice of buffer and cite the Young/Simmons precedent for the protective effect of ammonium chloride, as well as the negative impact of phosphate, HEPES and MES buffers.
Action 1-9b. We confirmed the negative impact of the suggested phosphate, HEPES and MES buffers by carrying out RCM on diene-appended native DNA (**ATGC-1**), **hexa-T-1**, native RNA **(purine25-AUGC-1**) and **2´-OMe-AUGC-1**. Even under the optimized conditions (0.8 M each of NaCl and NEt_3·HCl at pH 5.0; 70 °C for 30 min), in situ yields were <10%. We add a comment to this effect in the text, and add the data in Supplementary Table S12, entry 10-12 (**hexa-T-1**); in Table S17, entry 16-18 (**ATGC-1**), in Supplementary p. S115-S116 (**purine25-AUGC-1**) and S118-S119 (**2'-OMe-AUGC-1**).

- Time/temperature minimization: Explore RT with longer times or brief to reduce nucleic acid damage. Done: see Action 1-6.
- Ethylene control set: Run on-DNA RCM ± ethylene (or alternate olefin scavengers) to directly test deamination suppression in the conjugate context. Done: see Actions 1-4, 1-5.
- Sequence scope: Evaluate A-rich vs A-poor sequences to probe deamination liability; include RNA with varied purine content. Done: see Action 1-8.

Reviewer 2: Comments. This manuscript applied the anionic, water-soluble ruthenium catalyst Ru-1 for the on-DNA RCM reaction. High compatibility is demonstrated with different oligonucleotide tags including DNA and RNA oligomers and a chemically-stabilized DNA congener. Synthetic useful conversions and recovery of the nucleic acid was achieved with this novel Ru catalyst. This method demonstrated the application of RCM for the encoded library synthesis. This manuscript is recommended to publish after the following questions are addressed.

2-1. The authors has demonstrated its application for the single strand oligo, whether this method could also applicable for the dsDNA, it’s better for the authors also to explore this application.

Action 2-1. We followed this suggestion and now demonstrate that macrocyclization indeed succeeds with dsDNA conjugates. Three such substrates were examined: in situ yields are excellent (87-96%), and isolated yields of the dsDNA-macrocycle products were 42–82% (**Fig. 7a**), with no MS evidence of depurination or ring-contracted side products (**Supplementary Fig. S34**).

2-2. It’s better for the authors to explore whether this method is applicable for the plate format synthesis which is important for the encoded library synthesis.
Action 2-2. We conducted the suggested experiment in plate format. See Action 1-5.

2-3. It’s better for the authors to optimize and explore a little more about the function of NaCl and ammonium salt for this reaction.

Action 2-3. We now comment on the critical role of chloride salts established in our 2025 JACS paper (see Action 1-1). We also discuss the Young-Simmons finding concerning the superior performance of the ammonium salt buffer, and our experiments involving optimization of the NaCl–NEt_3•HCl system.

Reviewer #3 (Remarks to the Author):
The authors describe the synthesis of macrocyclic compounds anchored on short DNA or RNA fragments using ring-closing metathesis (RCM) carried out in aqueous media. This study paves the way toward the

preparation of constructs applicable in targeted therapies, where nucleic acids can act as carriers for prodrugs or drugs.
The work is very well thought out and clearly required active collaboration between chemists and molecular biologists. The authors employed a well-known catalyst, AquaMet, as well as a second complex based on a CAAC ligand decorated with a sulfonate tag, recently introduced by the Fogg group. They then examined the stability of these complexes in the presence of nucleotides and their activity in a model RCM reaction, with Ru1 performing significantly better in both cases. Importantly, they also investigated the reverse relationship—namely, the stability of nucleotides and nucleic acid fragments in the presence of both catalysts.

3-1. While the data for AquaMet appear to be missing in the first case (these should be added), both DNA and RNA fragments proved relatively stable in the presence of Ru1, especially when NaCl was included. Salt also had a positive, albeit weaker, effect on stability in the presence of AM.

Action 3-1. The AquaMet (**AM**) data were in fact included: we now state more clearly that the instability of **AM** to DNA is shown in Fig. 2, and the reciprocal instability of DNA in the presence of **AM** in Fig 5.

3-2. Having established stability and optimized reaction conditions, the authors successfully obtained a series of macrocyclic compounds anchored on nucleic acid fragments, achieving satisfactory yields and DNA recovery (this term should be clarified for the reader).

Reply. The referee has identified unintended ambiguities arising from differences in the standard nomenclature in chemical biology, vs catalytic chemistry. We now clarify our usage.
Action 3-2. We replace "conversion" with "in situ yield", and define this in the text as the fraction of starting material that is converted to product. Similarly, we note that "isolated yields" of the modified oligonucleotide reflect both in situ yields and losses on isolation via chromatography. Lastly, we define total DNA/RNA recovery as the sum of macrocycle product and unconsumed starting material isolated after HPLC.

3-3. This work is groundbreaking. Although it employs a very high catalyst loading (10 equiv. relative to substrate), which limits its immediate practical applications on a larger scale, it opens promising avenues for further discoveries. Overall, I have very few concerns. I would only recommend clarifying some biological terminology (e.g., “DNA ligation”) so that readers with limited expertise in molecular biology can also fully appreciate the significance of this study.
Action 3-3. We thank the referee for the assessment, and have sought to identify and explain terms as they arise.

REVIEWER 2: Comments. The authors have addressed all my previous concerns, current manuscript is recommended to publish at nature communications

Reply. We sincerely thank the reviewer for the careful evaluation of our manuscript and for the positive recommendation.

REVIEWER 4: Comments. I read with attention and several times this manuscript, as well as the reports of the Referees and the point-to-point answers by the authors. The authors have done a great job addressing all three reviewers' comments, adding new experiments and expanding the discussion to clarify key points. Overall, the authors have established an efficient RCM reaction for both DNA- and RNA-conjugated substrates using an anionic, water-soluble ruthenium catalyst. Given the high conversion and recovery achieved with DNA templates, and particularly as this represents the first successful application of the RCM to RNA modification, a long-standing challenge in nucleic acid chemistry, the prior on-DNA RCM methodology does not compromise the novelty or application potential of the present work. Although the reported RCM methodology will have great application potential in multidisciplinary fields, the manuscript's presentation is too specialized. To enhance accessibility for the broad readership of Nature Communications, the authors are encouraged to revise the text, particularly the Introduction and Summary sections, to clarify key concepts and explicitly articulate the broader implications of the findings. These revisions can be incorporated during the final production stage; no additional round of peer review is required.

Reply. We thank the reviewer for the enthusiastic comments and useful suggestions

1. Page 1, In the Abstract, a sentence explicitly underscoring the RCM for RNA modification could be added.

Action 1: We added the following sentence to the manuscript: "This advance paves the way for uptake of ring-closing metathesis in water for encoded-library technologies, and for broader applications of nucleic acids, potentially including RNA-oligonucleotide drugs."

2. In the Introduction or Summary section, a concise yet comprehensive discussion of the application potential of the developed RCM reaction in RNA research should be incorporated.

Action 2a: We added the following sentence to the Introduction: "Importantly, [these capabilities] also offer new opportunities to design structurally modulated RNA oligonucleotides via olefin linkages. Today, the chemistry toolbox for structural modulation of RNA oligomers is limited to head-to-tail cyclization via enzymatic ligation of the phosphate backbone, and Cu-promoted click chemistry." (references cited: https://pmc.ncbi.nlm.nih.gov/articles/PMC8670866/ and https://pubmed.ncbi.nlm.nih.gov/34508350/)

Action 2b: We added the following statement to the Conclusions: [These advances] also open the door to broader applications of nucleic acids, potentially including novel DNA- and RNA-oligonucleotide drugs.

3. Page 1, line 21: Several recent reviews (https://doi.org/10.1016/j.trechm.2025.08.008; https://doi.org/10.1016/j.apsb.2023.10.001; https://doi.org/10.1039/D5CC02904J) on DEL chemistry development could be included and discussed.

Page 1, lines 28–40: A concise yet comprehensive summary of recent advances in DNA-encoded macrocyclic libraries, and the key challenges currently impeding their construction and broader application, should be incorporated into the Introduction (https://doi.org/10.1039/D1CB00161B).

Action 3: We addressed both comments early in the manuscript. In the Introduction, we now state: "DEL researchers are increasingly seeking ways to access libraries of DNA-encoded macrocycles beyond those available via the established methodologies of head-to-tail lactam formation and Cu-promoted 'click' chemistry (alkyne-azide cycloaddition). Other approaches such as thioether formation are slowly being adopted. In general, however, current synthetic strategies typically produce polar macrocycles that would require extensive optimization for bioavailability, a prerequisite to reach intracellular targets, for example." (*all references proposed by the reviewer cited*)

4. Page 2, line 53: In Fig. 1a, the therapeutic indications and clinical development stages of the depicted macrocyclic compounds could be explicitly annotated to enhance accessibility for the broad, interdisciplinary readership of Nature Communications.

Action 4: We annotated the drugs in the Figure legend: "Pacritinib, a drug used to treat myelofibrosis, Paritaprevir, a drug used to treat Hepatitis C infection, MK-0616, a clinical candidate for the treatment of hypercholesterolemia."

5. Page 2, line 53: In Fig. 1d, RNA-compatible acidity could also be added.

Action 5: We added “RNA-compatible acidity” in the equation for Figure 1d.

Other changes: Minor improvements in wording were made. We added an acknowledgement of early contributions by an undergraduate student on the degradation of AquaMet.
